# Characteristics and Prognostic Analysis of 55 Patients With Pulmonary Sarcomatoid Carcinoma

**DOI:** 10.3389/fonc.2022.833486

**Published:** 2022-05-03

**Authors:** Jiachun Sun, Zhiyi Jiang, Tanyou Shan, Ruina Yang, Dejiu Kong, Junshuai Rui, Xinyang Li, Guoqiang Kong, Baoping Chang

**Affiliations:** ^1^ Henan Key Laboratory of Cancer Epigenetics, Cancer Hospital, The First Affiliated Hospital, College of Clinical Medicine, Medical College of Henan University of Science and Technology, Luoyang, China; ^2^ Medical College, Henan University of Science and Technology, Luoyang, China

**Keywords:** pulmonary sarcomatoid carcinoma, clinical characteristics, cathological characteristics, treatment, prognosis

## Abstract

Pulmonary sarcomatoid carcinoma (PSC) is a rare and aggressive subtype of non-small-cell lung cancer (NSCLC). Here, we present information on the clinicopathologic characteristics and clinical outcomes of this type of cancer. Clinicopathologic data from 55 patients treated at a single cancer center from January 2011 to December 2018 were retrospectively analyzed. The patients were mostly male (76.4%), with a median age of 66 years and a history of smoking (54.5%). Most had symptoms, and about 60% presented with locally advanced or metastatic disease at diagnosis. Of the 55 cases, 21 were diagnosed by surgical resection. Pleomorphic cancer was the most common subtype (58.1%). With a median follow-up period of 13.2 months, the average survival time of the patients was 16.1 months, and the median survival time was 12 months. The overall survival rates for 1, 2, and 3 years were 52.7%, 18.2%, and 9.1%, respectively. Univariate analysis showed that prognosis of the patients was influenced by tumor size, T stage, metastatic status, and surgery (*p* < 0.05). Multivariate analysis showed that T stage (*p* = 0.034) was an independent prognostic factor. There are few reports on the natural history of PSC, and its clinicopathological characteristics remain unclear. Herein, a retrospective review 55 individuals with PSC found that T stage was an independent predictor of survival. Surgical resection was associated with better prognosis.

## Introduction

Pulmonary sarcomatoid carcinoma (PSC) is a rare type of non-small-cell lung cancer (NSCLC). The incidence of PSC is extremely low, accounting for about 0.1 to 0.5% of pulmonary malignancies (1, 2). PSC was defined as “a variant of squamous cell carcinoma” in 1981 and renamed as “carcinomas with pleomorphic, sarcomatoid or sarcomatous elements” in 1999. In 2004, the World Health Organization (WHO) classified PSC as a collective category of malignant lung epithelial cell tumors based on pathological and morphological characteristics. Until 2015, both the nomenclature and diagnostic criteria of PSC under the WHO tumor classification remained basically unchanged (3).

According to the literature, PSC is the name for a group of metastatic tumors derived from the same original epithelial cells after undergoing an epithelial-mesenchymal transition. The tumors have both epithelial and mesenchymal features (4). With continuing progress in histopathological and molecular diagnostic techniques, in-depth research on PSC has increased. However, knowledge of PSC is still limited due to its rarity. Targeted therapy plays an important role in the treatment of NSCLC. Several studies detected EGFR, MET, and BRAF mutations in PSC tissue samples (5). Some data suggested that targeted therapy for these mutations achieved good curative effect (5). Pan-cancer analysis revealed that PSC was among those tumor types with the most evidence of TMB and LF, indicating likely a high neoantigen burden and a T-cell-inflamed tumor microenvironment (TME). Considering the similar TME of the epithelial and sarcomatoid components from the same patient demonstrated by fluorescent multiplex IHC, the two components could both have a favorable response to immunotherapy. In addition, cases of PSC with dramatic response to immune checkpoint inhibitors were reported (6, 7).

In the present study, 55 PSC patients treated between 2011 and 2018 at a single center were selected as retrospective subjects. In addition to summarizing clinical features, such as age, gender, pathological type, and staging, we investigated the clinical diagnosis, treatment and prognosis, as well as stage characteristics of the tumors. We also examined current relevant diagnoses and treatments with the goal of establishing a clinical foundation for subsequent investigations.

## Material and Methods

### Ethical Approval

All experimental research was performed with the approval of the ethics committee of Medical Ethics Committee The First Affiliated Hospital of Henan University of Science and Technology. The IRB approval number is 2021-03-B055. Research carried out on humans was in compliance with the Helsinki Declaration.

### Patients and Tissue Samples

The study involved 55 patients with PSC who were treated at the First Affiliated Hospital of Henan University of Science between January 2011 and December 2018. All specimens from these patients were confirmed by two experienced pathologists according to the 2004 WHO classification of PSC. The diagnosis was confirmed when at least 10% of the tumor contained a sarcomatoid component consisting of spindle or pleomorphic giant cells, or both (1).

### Clinical Data

Of the 55 cases, 4 were spindle cell carcinoma, 2 were giant cell carcinoma, 17 were carcinosarcoma, and the remaining cases were not diagnosed as a specific subtype of sarcomatoid carcinoma. The male to female ratio was 3.2:1 with 42 male patients and 13 females. Patient age ranged from 31–86 years with a mean of 66 years. Twenty-five patients had no history of smoking, 6 had a smoking history of less than 30 years, and 24 had a smoking history of 30 years plus. Symptoms of cough, expectoration, chest pain, back pain, and bloody sputum were found in all but 9 asymptomatic individuals. In the latter group, physical examination aided diagnosis. With respect to location, there were 8 tumors in the left upper lobe of the lung, 10 in the left lower lobe, 2 in the right upper lobe, 10 in the mid-lobe, and 5 in the right lower lobe. The remaining cases had multiple lesions. According to the international TNM standard of staging of lung cancer, the stage of the 55 resections or biopsy pathologic results were as follows: 1 cases of Phase Ia, 21 cases of Phase II (3 were IIa and 18 were IIb), 12 cases of Phase IIIa, and 21 cases of Phase IV.

### Follow-up

All patients were followed with outpatient visits, telephone call, or follow-up letter, through December 30, 2018. Duration of the follow-up was between one and 86 months, with the median follow-up period being 13.2 months. At the end of the observation period, 49 patients had died, and 6 patients were alive.

### Statistical Methods

Data were entered, analyzed, and processed using SPSS 22.0 statistical software. Data was computed, and the survival curve was created using the Kaplan-Meier method. The log rank method was used for univariate analysis of prognostic factors, and the Cox model was used for multivariate analysis. The inspection level was α = 0.05.

### Pathological Results

Eight cases were confirmed by fiberoptic bronchoscopy (16 cases in total, positive rate 50%). Computed tomography-guided lung tumor biopsy confirmed 26 cases (34 cases in total, positive rate 76.47%). The remaining 21 cases were not correctly diagnosed before the pathological analysis of surgical specimens. Tumor size was calculated according to the maximum diameter. In this study, the tumor sizes ranged from 2.9 –11.4 cm with an average size of 6.5 cm, including 30 cases with a tumor size ≤ 6.5 cm and 25 cases with size > 6.5 cm. Of the 55 cases, one had neuroendocrine differentiation and one case was large cell neuroendocrine carcinoma that expressed CD56.

### Histologic and Immunohistochemistry Findings

**Figure 1:**
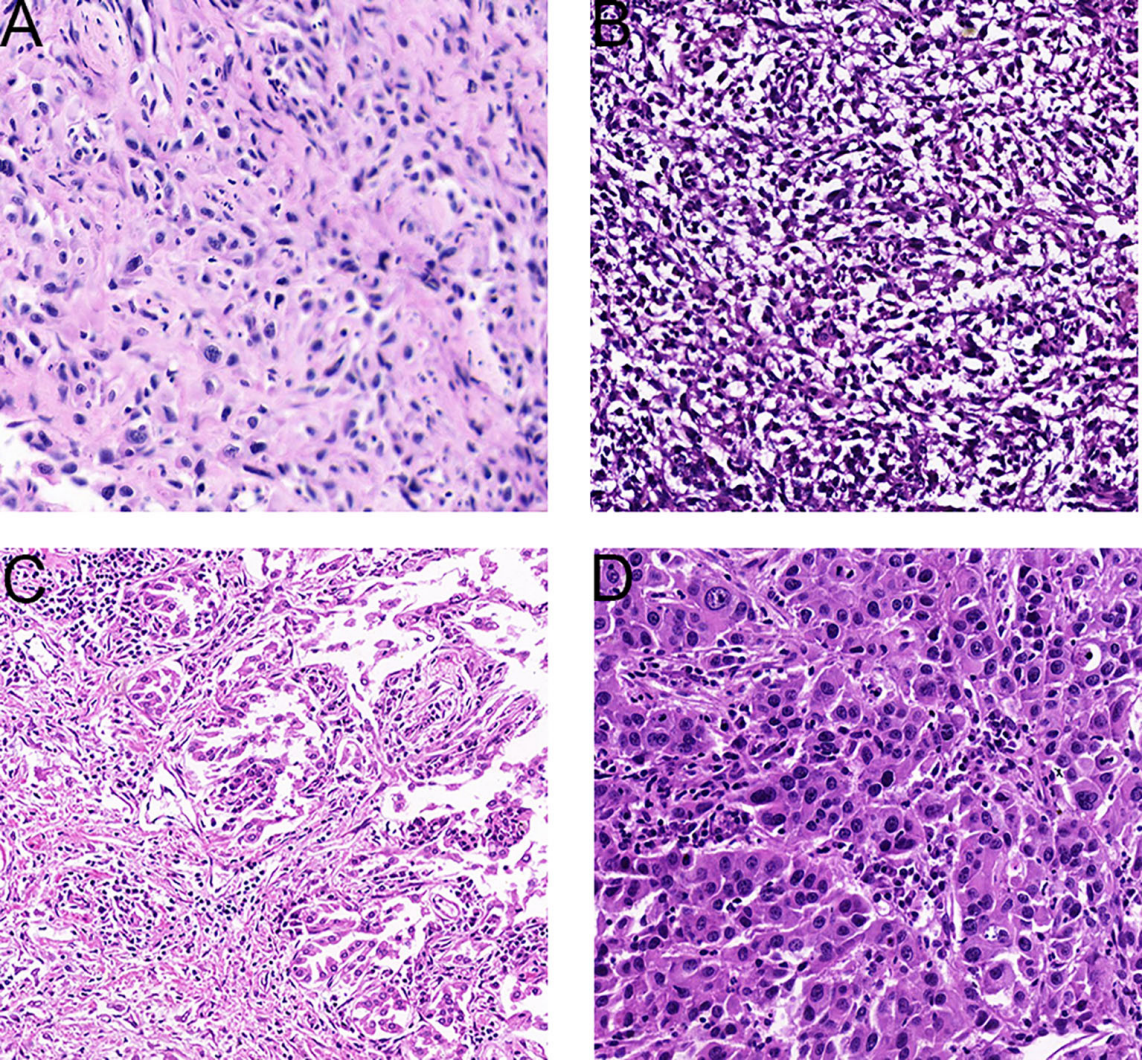
Representative tissue histology of PSC tumors. **(A)**, Spindle cell carcinoma (HE× 200). **(B)**, Carcinosarcoma (HE×200). **(C)**, Pleomorphic carcinoma (HE×200). **(D)**, Giant cell carcinoma (HE×200).

Spindle cell carcinoma is characterized by malignant spindle cells with deeply dyed nuclei and irregular nucleoli (8, 9) **Figure 1A**.

For carcinosarcoma, in addition to obvious epithelial components, there are heterologous sarcomatoid components, such as malignant cartilage or bone, or rhabdomyoma (8, 9) **Figure 1B**. In some of the patients enrolled in this study, carcinosarcoma presented as a mixture of squamous cell carcinoma and sarcoma.

Pleomorphic cancer is composed of malignant giant cells and/or spindle cells and/or epithelial components such as squamous cell carcinoma or adenocarcinoma (8, 9) **Figure 1C**.

Giant cells appear in the aggregate tumor cells, usually lacking adenoid or squamous differentiation. In the tumors studied herein, cells were typically spindle type or elongated, existing singly, in loose clusters, or as many pieces. Nuclei were single, large, and spindle-shaped. Neoplastic cells exhibited high nuclear-to-cytoplasmic ratios and prominent nucleoli, and mitotic figures were prominent. At the same time, multinucleated tumor giant cells and mucous matrix were observed. When the epithelial component is not obvious morphologically, it can be difficult to distinguish spindle cell or giant cell carcinoma from spindle cell sarcoma or pleomorphic sarcoma by cell morphology alone. Immunocytochemical staining is an aid to distinguish these classifications **Figure 1D**.

**Figure 2:**
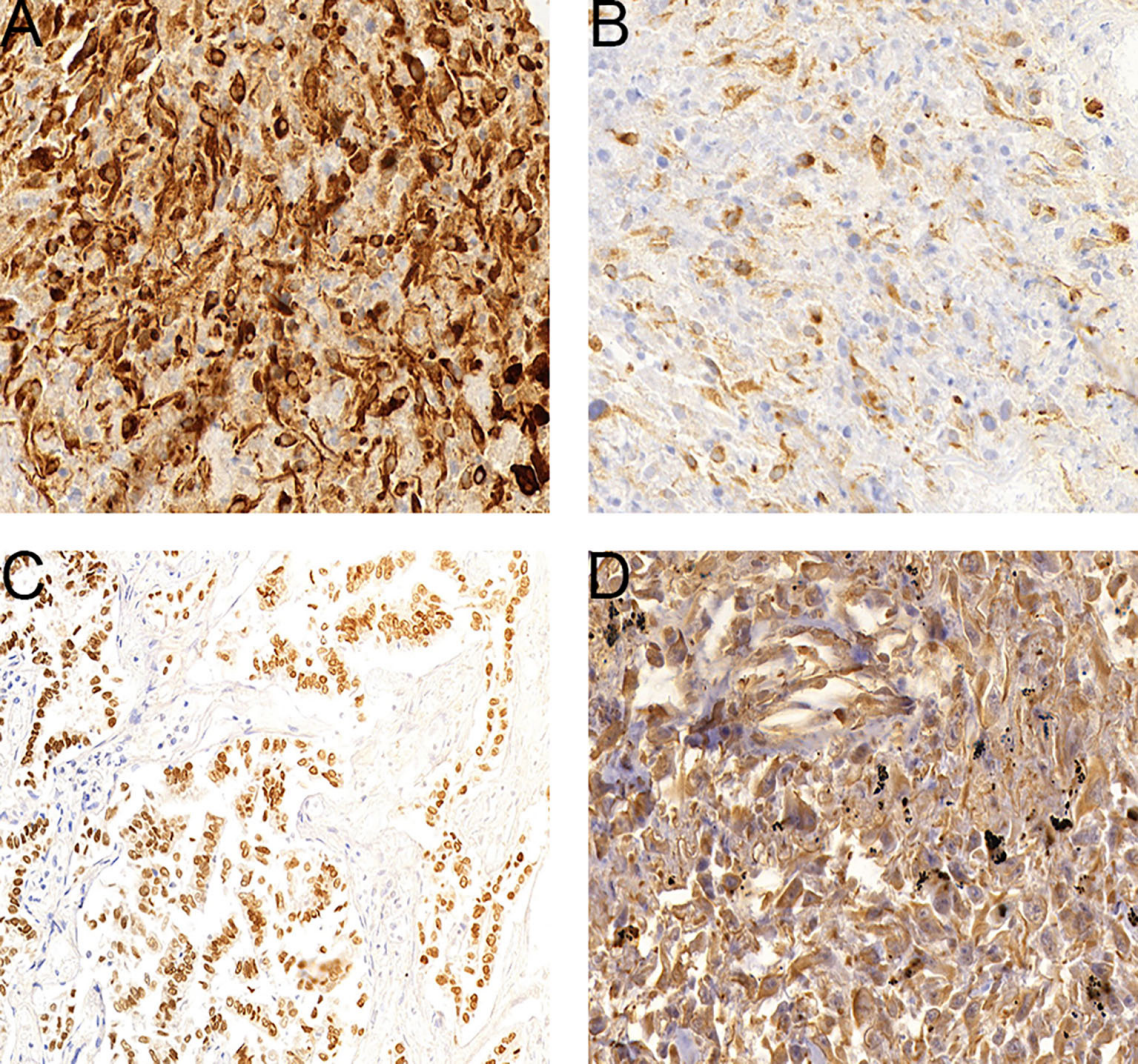
Expression levels of select genes in PSC tumors. **(A)**, CK-positive staining cells in a sarcomatoid carcinoma. **(B)**, CK7-positive staining cells in a sarcomatoid carcinoma. **(C)**, TTF-1-positive staining cells in a sarcomatoid carcinoma. **(D)**, Vim-positive staining cells in a sarcomatoid carcinoma.

Immunohistochemistry was performed with antibodies including Vim, CK, P63, S-100, Ki67, CK7, EMA, TTF-1, CK5/6 and Syn, using the streptavidin-peroxidase method. CK and Vim showed the highest expression and was noted in 94.5% (52/55) and 98.2% (54/55) of tumors, respectively (**Figure 2**). EMA was expressed in 58.3% (7/12) of tumors. TTF-1, P63, CK5/6, and CK7 were expressed in 63.6% (35/55), 27.8% (9/38), 22.4% (11/49) and 45.5% (25/55) of tumors, respectively. S-100 and Syn expression was noted least often being found in 18.2% (4/22) and 20.0% (3/15) of tumors, respectively.

### Treatment Methods

Twenty-one patients underwent ipsilateral lobectomy or pneumonectomy, combined with mediastinal lymph node dissection. Following tumor resection, ten patients received chemotherapy including gemcitabine, vinorelbine, paclitaxel, docetaxel, and cisplatin.

### Survival Analysis

**Figure 3:**
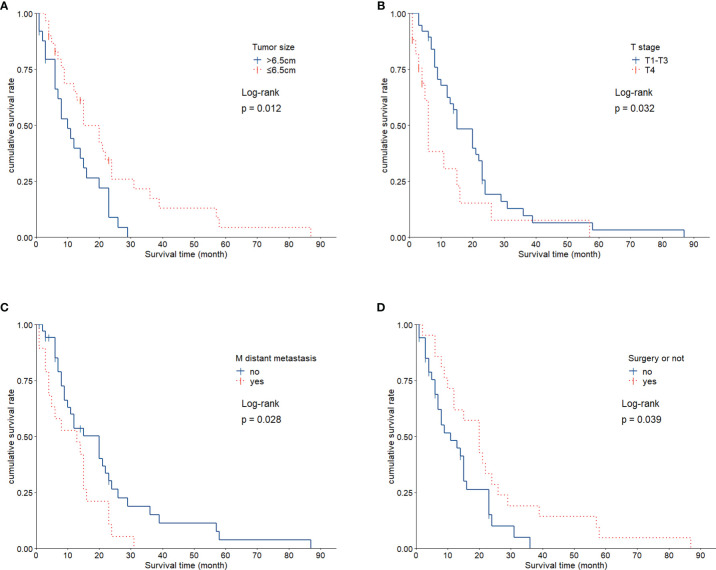
Kaplan-Meier Survival Curves for Each Factor. **(A)**, Patients with small tumor size (≤ 6.5 cm) exhibited significantly longer survival than did patients with larger tumors (*p* = 0.012). **(B)**, Statistically significant difference in survival between individuals with T1+3 and T4 (*p* = 0.032). **(C)**, Survival was significantly different based on M stage (*p* = 0.028). **(D)**, Patients showed significant differences in survival as related to surgical treatment (*p* = 0.039).

The average survival time of the patients was 16.1 months, and the median survival time was 12 months (95% confidence interval, 11.8–20.4 months). The overall survival rates for 1, 2, and 3 years were 52.7%, 18.2%, and 9.1%, respectively. We analyzed the influence of gender, age, tumor size, smoking history, TNM stage, and treatment factors on prognosis. Univariate analysis (**Figures 3A**–**D**) showed that tumor size, T stage, metastatic status, and surgery had a significant influence on the prognosis of patients (*p* < 0.05, **Table 1**). In addition, multivariate analysis showed that T stage (*p* = 0.034) was an independent prognostic factor (**Table 2**).

**Table 1:** Clinicopathological Characteristics and Survival Analysis of 55 Patients with Pulmonary Sarcomatoid Carcinoma (PSC).

Characters	Case Numbers	Median Survival Time (Months) (95% CI)	3-Year Survival Rate (%)	*X* ^2^	*p*
**Gender**					
Female	13	12.000 (4.954–19.046)	0.077	0.120	0.729
Male	42	15.000 (11.589–18.411)	0.097		
**Age**					
≤65 y	24	11.000 (6.618–15.382)	0.049	0.510	0.475
>65 y	31	15.000 (8.932–21.068)	0.125		
**Tumor Size**					
≤6.5 cm	30	15.000 (8.168–21.832)	0.173	6.361	0.012
>6.5 cm	25	10.000 (5.375–14.625)	0.000		
**Smoking History**					
No	25	15.000 (9.317–20.683)	0.051	0.012	0.912
Yes	30	13.000 (8.828–17.172)	0.125		
**T Stage**					
T1–3	38	15.000 (9.303–20.697)	0.096	4.614	0.032
T4	17	6.000 (4.879–7.121)	0.076		
**N Lymph Node Metastasis**					
No	20	12.000 (7.782–16.218)	0.000	1.583	0.208
Yes	35	16.000 (8.774–23.226)	0.158		
**M Metastasis**					
No	36	20.000 (12.132–27.868)	0.151	4.799	0.028
Yes	19	13.000 (1.625–24.375)	0.000		
**Surgery**					
Yes	21	20.000 (12.592–27.408)	0.190	4.281	0.039
No	34	11.000 (2.366–19.634)	0.000		
**Chemotherapy**					
Yes	30	15.000 (12.540–17.460)	0.144	1.351	0.245
No	25	12.000 (1.984–22.016)	0.045		
**Postoperative Chemotherapy**					
Yes	10	20.000 (10.703–29.297)	0.300	1.971	0.160
No	11	15.000 (2.053–27.947)	0.091		

**Table 2:** Multivariate Cox Model Analysis of Effects on Pulmonary Sarcomatoid Carcinoma (PSC) Prognosis.

Variable	B	Wald	RR	95% *CI*	*p*
**Gender**	−0.255	0.268	0.775	0.296–2.032	0.605
**Age**	−0.252	0.528	0.777	0.394–1.533	0.467
**Tumor Size**	0.531	1.435	1.700	0.713–4.051	0.578
**Smoking History**	0.210	0.309	1.234	0.588–2.588	0.231
**T Stage**	0.836	4.500	2.307	1.066–4.996	0.034
**N Lymph Node Metastasis**	−0.472	2.119	0.624	0.330–1.178	0.145
**M Metastasis**	0.284	0.326	1.329	0.501–3.524	0.568
**Surgery**	0.733	2.326	2.081	0.812–5.334	0.127
**Chemotherapy**	0.376	1.131	1.456	0.728–2.911	0.288

## Discussion

A large-scale retrospective analysis of pulmonary sarcomatoid carcinoma conducted by Xiao et al., *via* the SEER database, found that the proportion of pulmonary sarcomatoid carcinoma was only 0.5% of lung cancer worldwide (10).In this current study, the 55 patients with pulmonary sarcomatoid carcinoma accounted for only 0.16% of the patients diagnosed with lung cancer during the same period, which was slightly lower than that reported by Li et al. (11–14). Our hospital may be located in the central and western regions of China, with relatively limited tumor diagnosis and treatment ability. In addition, This study lasted for 9 years, and some patients with pulmonary sarcomatoid carcinoma were not diagnosed; thus, they were not included in our study. PSC is more common in elderly men aged 65–75 years, especially those with a history of heavy smoking. The ratio of male to female patients is about 1.5:1 (8, 15). Our study enrolled 42 male and 13 female patients (3.2:1) ranging from 31–86 years. The median age was 66 years, and more than half of the subjects had a long history of smoking (54.5%), 27 of whom had smoked for more than 10 years. This suggests that smoking may be associated with the incidence of PSC. However, our sample showed a lack of statistical significance (p = 0.912) regarding the influence of smoking history on prognosis. Such an outcome is in accord with previous research results, indicating that smoking history has no significant influence on the overall survival of PSC patients.

Despite the variety of tumor types classified as PSC, it is still considered an epithelial carcinoma based electron microscopy and immunohistochemistry demonstration of concurrent carcinoma and sarcomatoid components in a given lesion, and that the sarcomatoid components may have evolved from the carcinoma (4). Considering that primary PSC is usually poorly differentiated and heterogeneous, relevant pathological patterns should be identified using a combination of pathomorphological and immunohistochemical analyses. Preoperative pathological diagnoses, such as sputum cytology, bronchoscopic biopsy, and CT-guided percutaneous needle lung biopsy, are often non definitive due to insufficiency of tissue. Moreover, intraoperative frozen section examinations are frequently inconsistent with postoperative paraffin section examinations (15, 16). In this study, 61.8% (34/55) of patients were diagnosed with pulmonary malignant tumor before operation, but only 3 cases were suspected to be spindle cell carcinoma. These findings emphasize the need to obtain bulk tissues for analysis.

According to the 2015 WHO classification of tumors, there are 5 subgroups of PSC: spindle cell carcinoma, giant cell carcinoma, carcinosarcoma, pleomorphic carcinoma, and pulmonary blastoma (8). Although clinical characteristics and imaging can suggest PSC, histopathological and immunohistochemical examinations are necessary for definitive diagnosis. There are two major categories of immunohistochemical indicators for PSC used currently: epithelial and mesenchymal biomarkers. Epithelial biomarkers include cytokeratin (CK), epithelial membrane antigen (EMA), pan-cytokeratin antibody (AE1/AE3), thyroid transcription factor (TTF-1), carcinoembryonic antigen (CEA), cytokeratin 7 (CK7), p40, and cytokeratin 5/6 (CK5/6). Mesenchymal biomarkers include vimentin and desmin. Herein, CK (94.5%; 52/55) and vimentin (98.2%; 54/55) were found expressed in the majority of tumors. These findings suggest that sarcomatoid components of PSC arise from transformed epithelial carcinoma.

PSC patients can also display mutations of EGFR of K-RAS genes, or EML4-ALK gene fusion. Indeed, 28.1% of PSC patients tested positive for the EGFR mutation, and 3% tested positive for the K-RAS mutation (2, 17). In our study, eleven patients were tested for EGFR mutation and one patient was found to be positive. These patients received Gefitinib, but no obvious efficacy was observed. The K-RAS gene mutation occurs about 38% more frequently than the EGFR mutation (18). However, mutations of K-RAS were not reported in our cohort. In individuals diagnosed with PSC, a major site of EGFR or K-RAS gene mutations is the epidermatoid tissue (19). Interestingly, PD-L1 expression positively correlated with KRAS mutation status (REF (20)). PD-L1 expression was noted in 44.4% and 12.0% of mutant and wild-type KRAS genes, respectively (21). Therapies targeting several genes, including EGFR-TKI (13, 22, 23), ALK-TKIs (24), MET-TKIs (25), antiangiogenic therapy (26, 27) and immune-checkpoint inhibitors (21, 28), were tested in patients with PSC, although given the rarity of the tumor type, large scale studies of any particular therapy may never be completed.

Treatments of PSC involves a multidisciplinary approach that integrates surgery as a primary treatment with the adjuvant chemoradiotherapy. Chemoradiotherapy is based on existing protocols for other types of NSCLC. Postoperative adjuvant chemotherapy improved patient survival rate, but the efficacy of adjuvant chemotherapy remains controversial (3, 12–15, 23, 29). In one report, 10 of the 21 patients who underwent operative treatment received adjuvant chemotherapy in the third or fourth post-operative week. No survival advantage was noted (p = 0.160); this is consistent other data (13, 14). and may be due to small cohort size and differences in tumor stage. Several studies suggest that late-stage PSC is minimally responsive to chemotherapy. Thirty patients with PSC received chemotherapy with no benefit (p = 0.245).

We found that tumor T staging was linked to survival rate, while lymphatic metastasis had no significant effect on prognosis. Two critical factors affecting T staging are tumor size, and the relationship between tumor tissues and the tissue of adjacent organs. Tumor size correlates with the components of sarcomatoid carcinoma. In particular, there may be fewer sarcomatoid components in smaller tumors (2).This may account for the improved prognosis of patients with small sarcomatoid carcinoma tumors. Tumor invasions of the mediastinum, heart, esophagus, or trachea are mostly found in T4 tumors. Prognosis of individuals with T4 tumors is very poor. In our study, the 3-year survival rate of T4 tumor bearing patients was 9.1%, which is lower than other reports (11, 12, 14, 23).Variation in the degree of surgical resection may contribute to these differences in survival.

The current study has several limitations. First, the cohort of 55 individuals is rather small. Moreover, the cohort does not reflect all epidemiologic characteristics of PSC. Second, diagnostic and therapeutic approaches changed during the study time. For example, analysis of EGFR gene mutations is a recent assay. Thus, uniform analysis of genetic variability was not possible in the study cohort. Consequently, gene mutations of PSC patients with obvious heterogeneity cannot be determined. Finally, the number of patients who received radiotherapy was so small that it could not be statistically analyzed. Therefore, the impact of radiotherapy on PSC treatment outcomes could not be determined.

In summary, PSC includes highly heterogeneous types of pulmonary malignancy. Characteristics of PSC include low incidence, difficult diagnosis, high malignancy grade, high probability of recurrence and metastasis, and minimal chemoradiotherapy sensitivity. Currently, tumor resection is the most effective therapy. Analysis of biomarkers should be performed as part of the diagnosis and treatment of PSC. Targeted therapy and immunotherapy play a role in the treatment of PSC.

## Data Availability Statement

The original contributions presented in the study are included in the article/**Supplementary Material**. Further inquiries can be directed to the corresponding authors.

## Ethics Statement

The studies involving human participants were reviewed and approved by the ethics committee of Medical Ethics Committee The First Affiliated Hospital of Henan University of Science and Technology. Written informed consent for participation was not required for this study in accordance with the national legislation and the institutional requirements.

## Author Contributions

BC, GK, and JS designed and carried out the experiments. ZJ and TS analyzed the data. ZJ followed-up, BC and JS wrote the manuscript. RY, DK, JR, and XL: Clinical data collected. BC provided supervision and guidance. The authors read and approved the final manuscript.

## Funding

This study was supported by the National Science Foundation of China (81872500).

## Conflict of Interest

The authors declare that the research was conducted in the absence of any commercial or financial relationships that could be construed as a potential conflict of interest.

## Publisher’s Note

All claims expressed in this article are solely those of the authors and do not necessarily represent those of their affiliated organizations, or those of the publisher, the editors and the reviewers. Any product that may be evaluated in this article, or claim that may be made by its manufacturer, is not guaranteed or endorsed by the publisher.
