# Supplementary figures and images for: Characteristics and Prognostic Analysis of 55 Patients With Pulmonary Sarcomatoid Carcinoma
Source: Front Oncol. 2022 May 3;12:833486. doi: 10.3389/fonc.2022.833486 (PMC9113756; doi:10.3389/fonc.2022.833486)

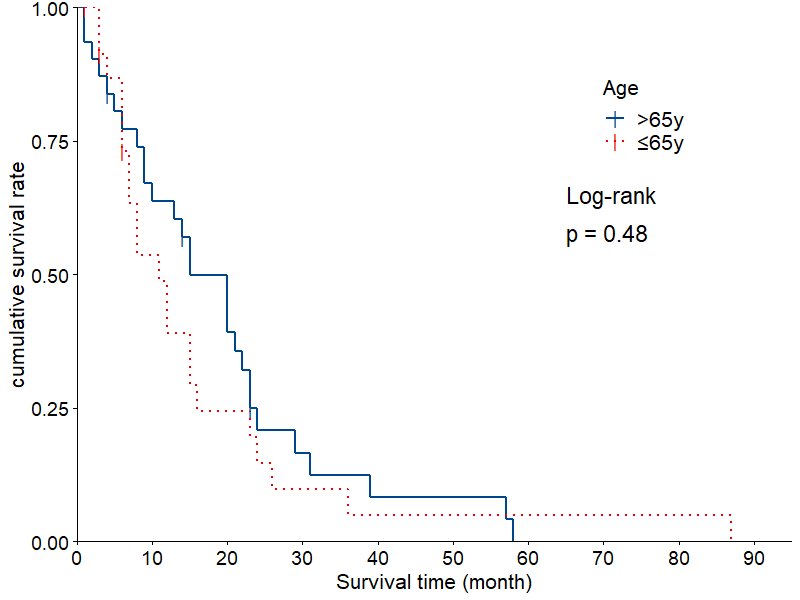

Supplement: Supplementary file 1 [file Image_1.png]

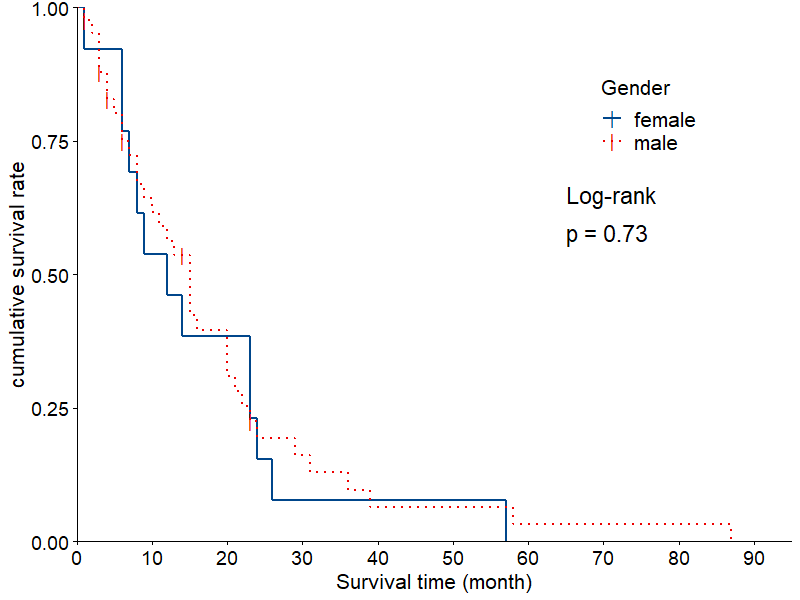

Supplement: Supplementary file 2 [file Image_2.png]

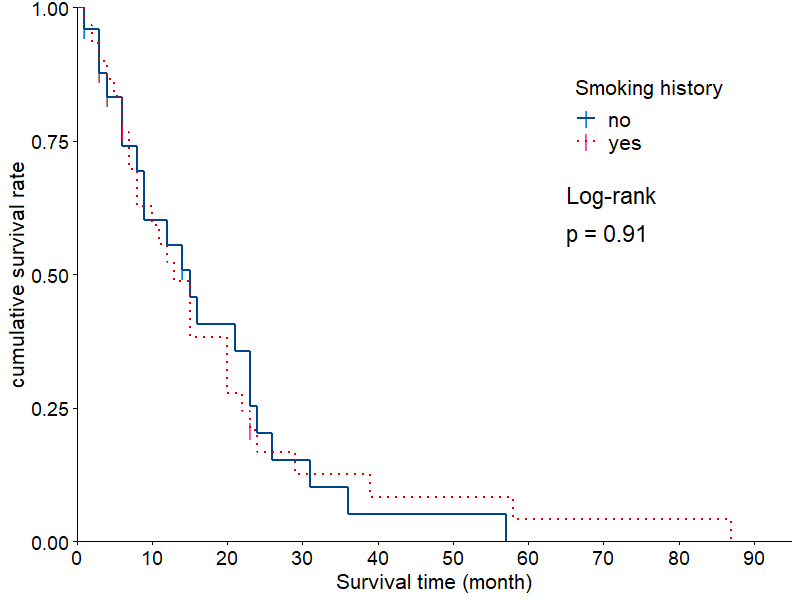

Supplement: Supplementary file 3 [file Image_3.png]

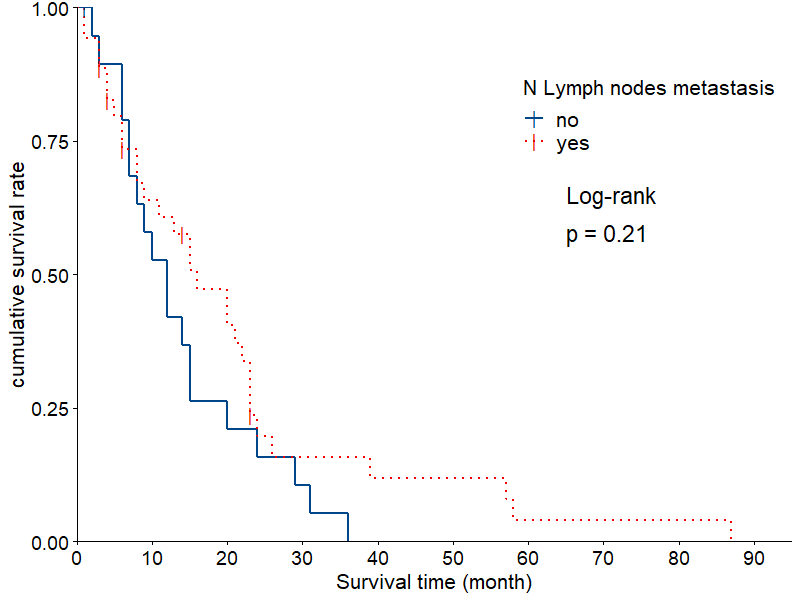

Supplement: Supplementary file 4 [file Image_4.png]

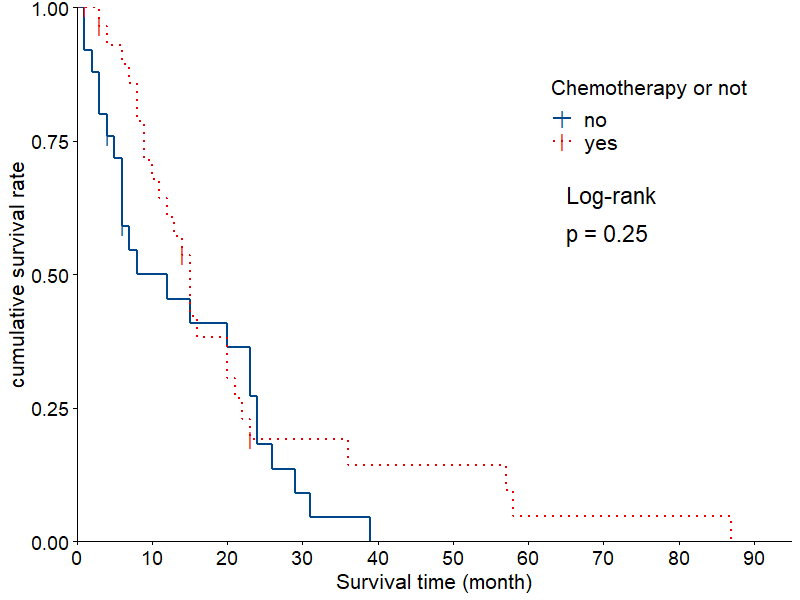

Supplement: Supplementary file 5 [file Image_5.png]

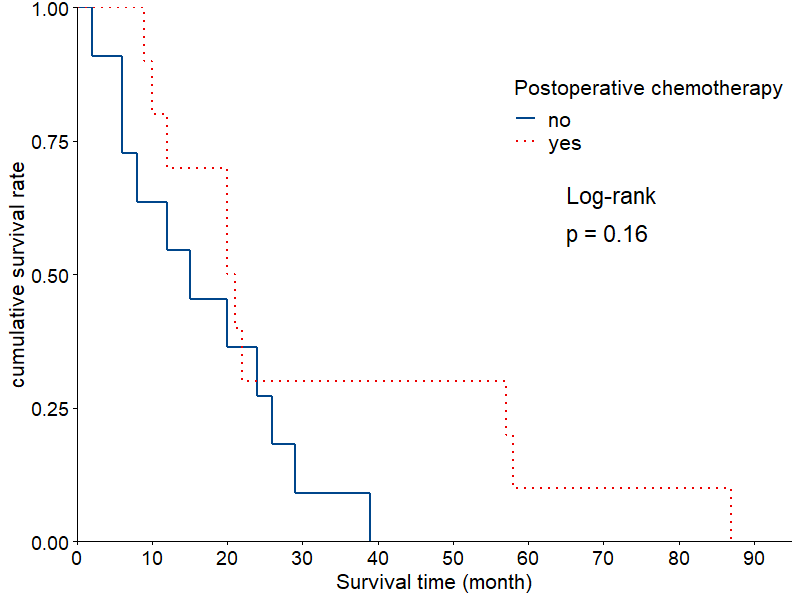

Supplement: Supplementary file 6 [file Image_6.png]

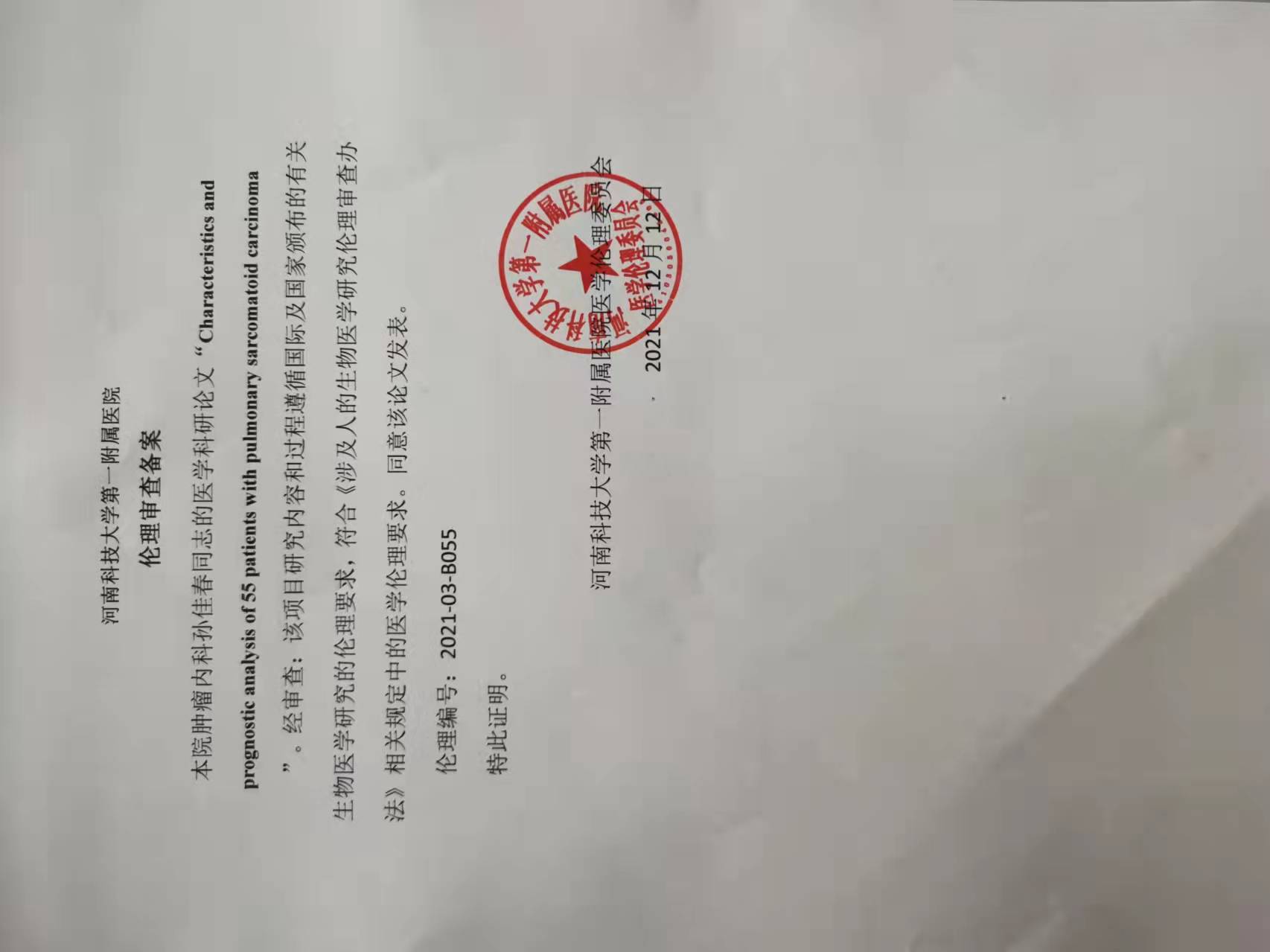

Supplement: Supplementary file 7 [file Image_7.jpeg]

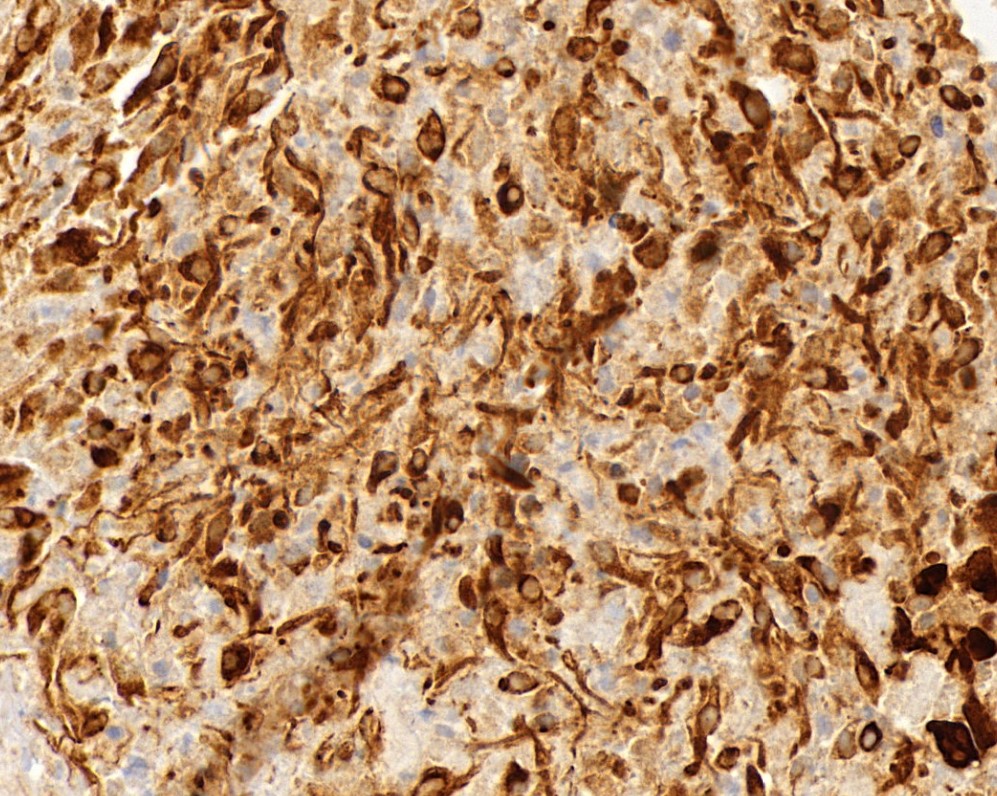


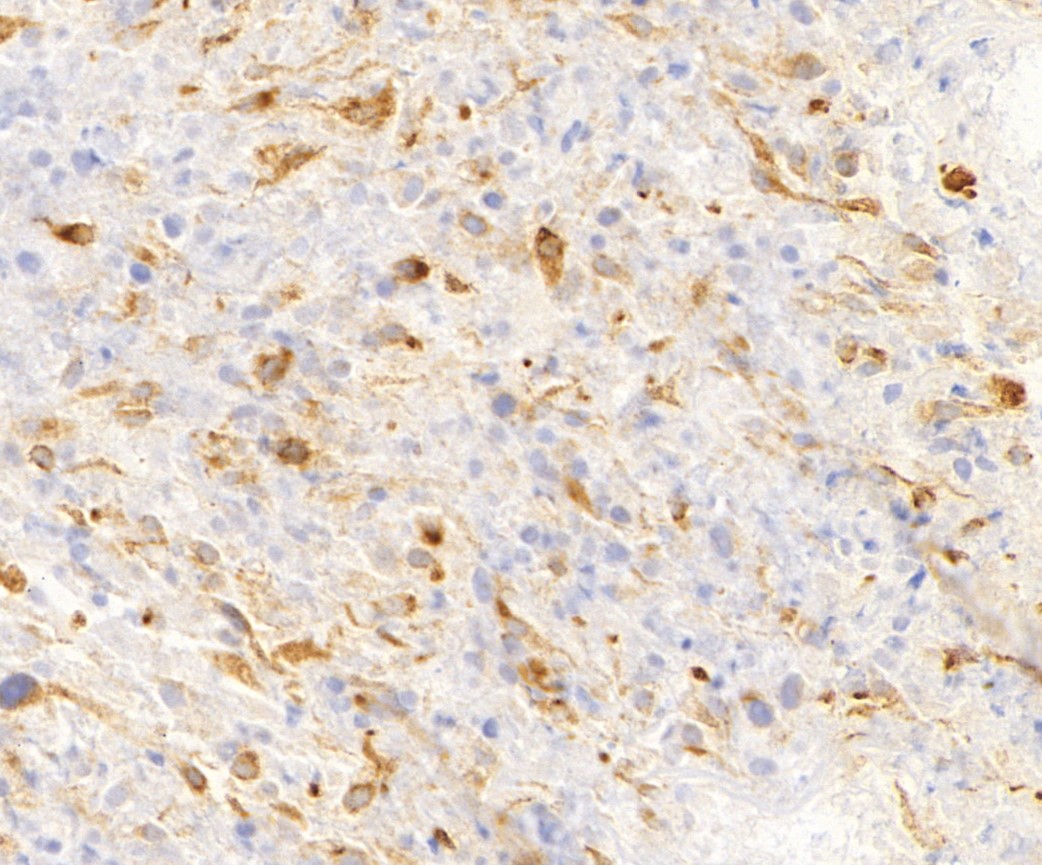


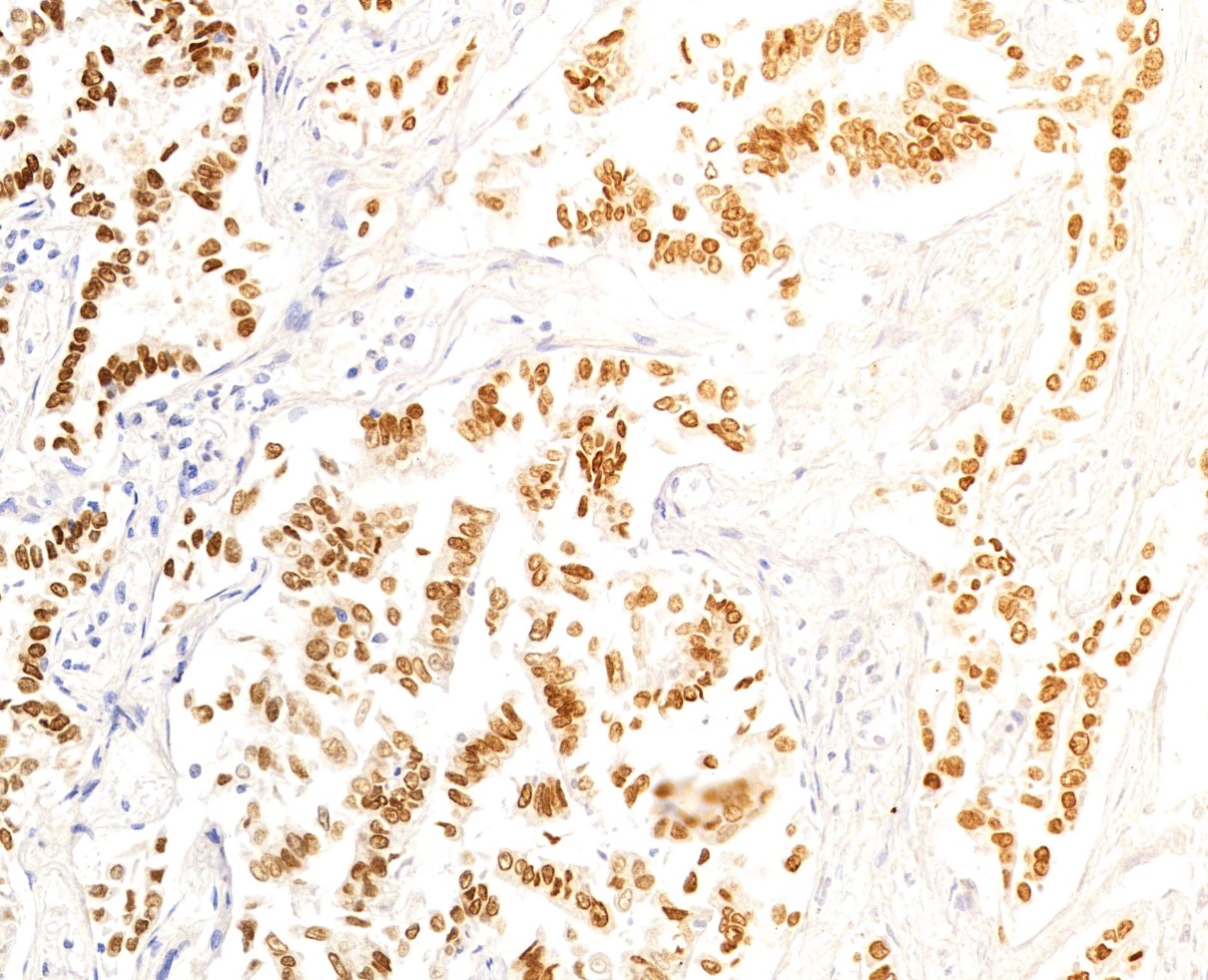


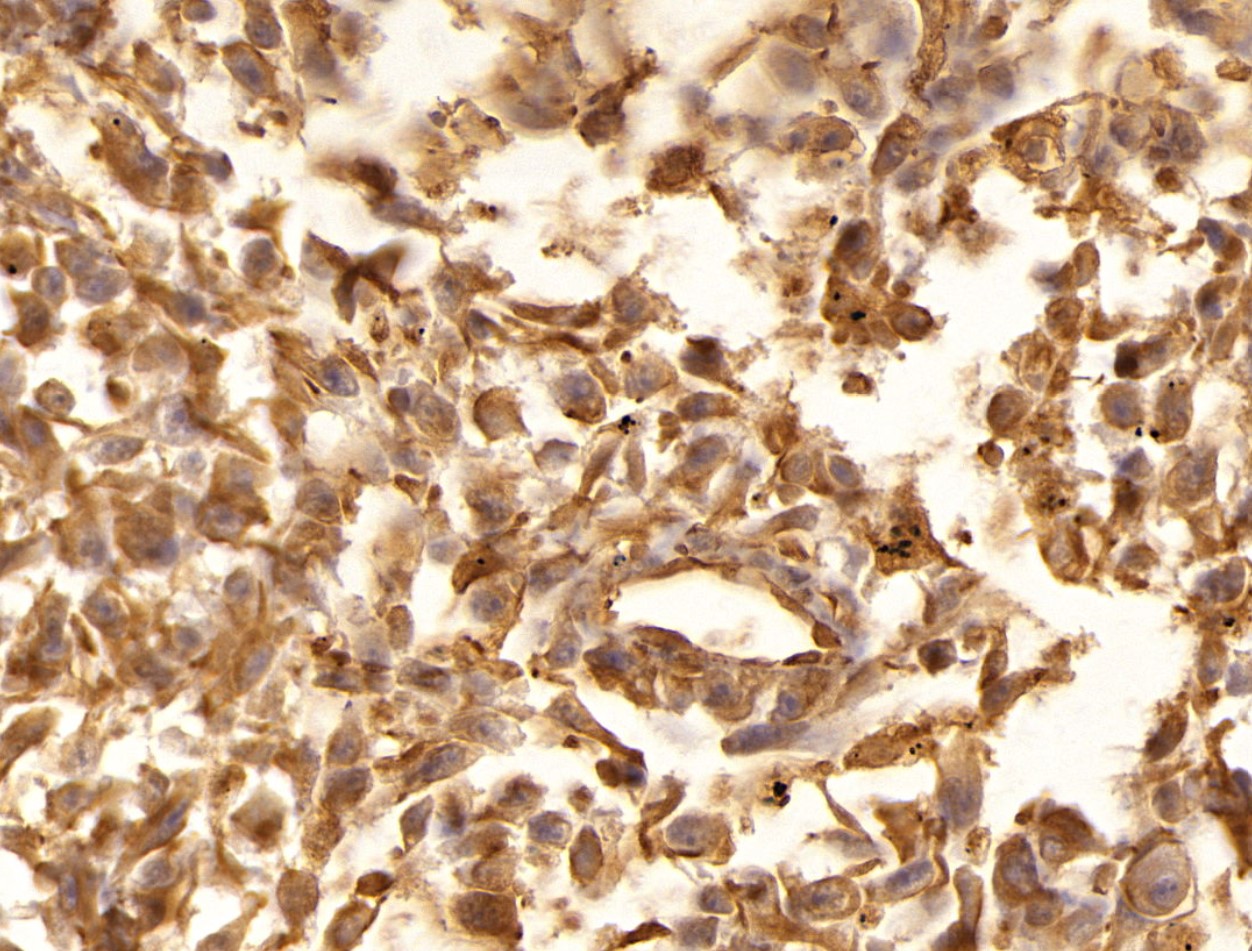

Supplement: Supplementary file 9 [file Table_2.docx]

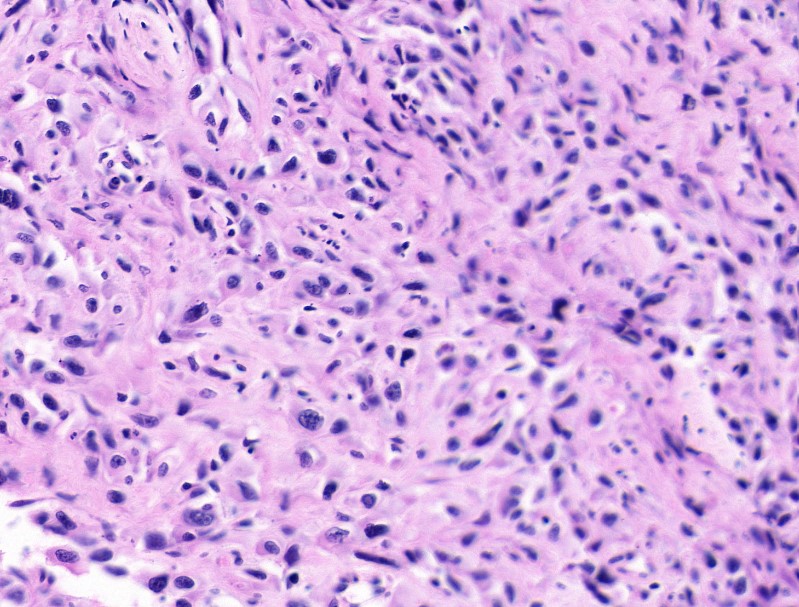


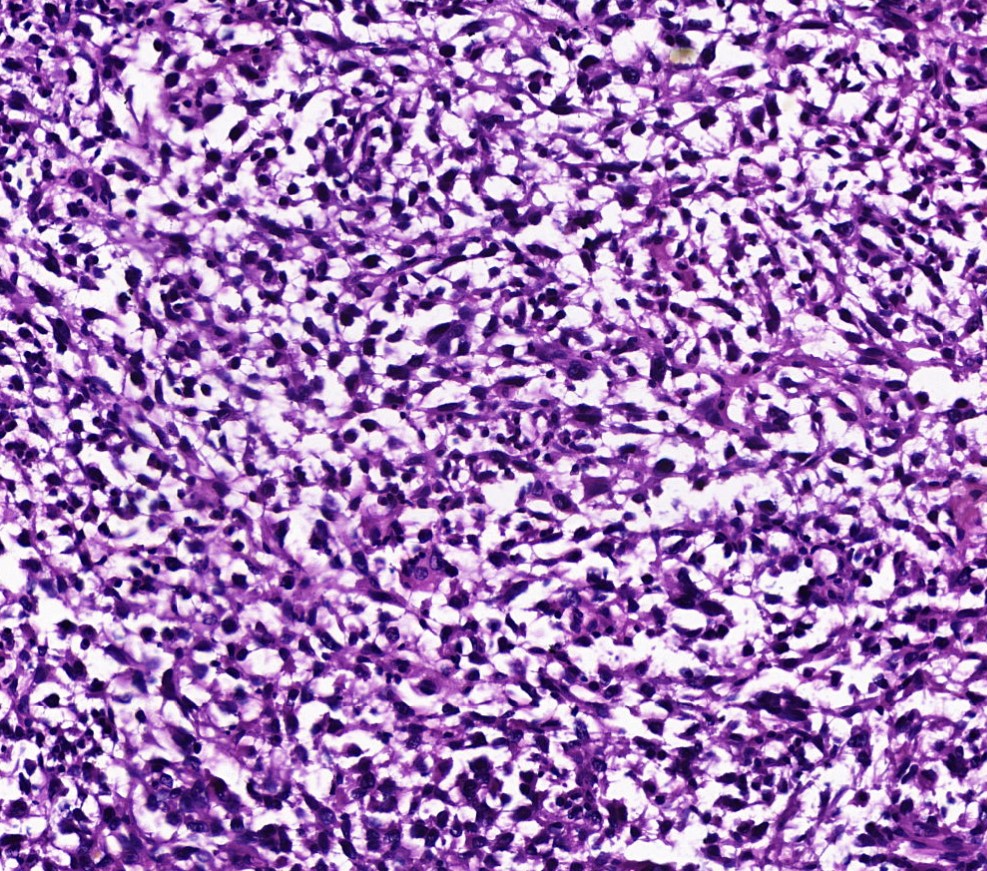

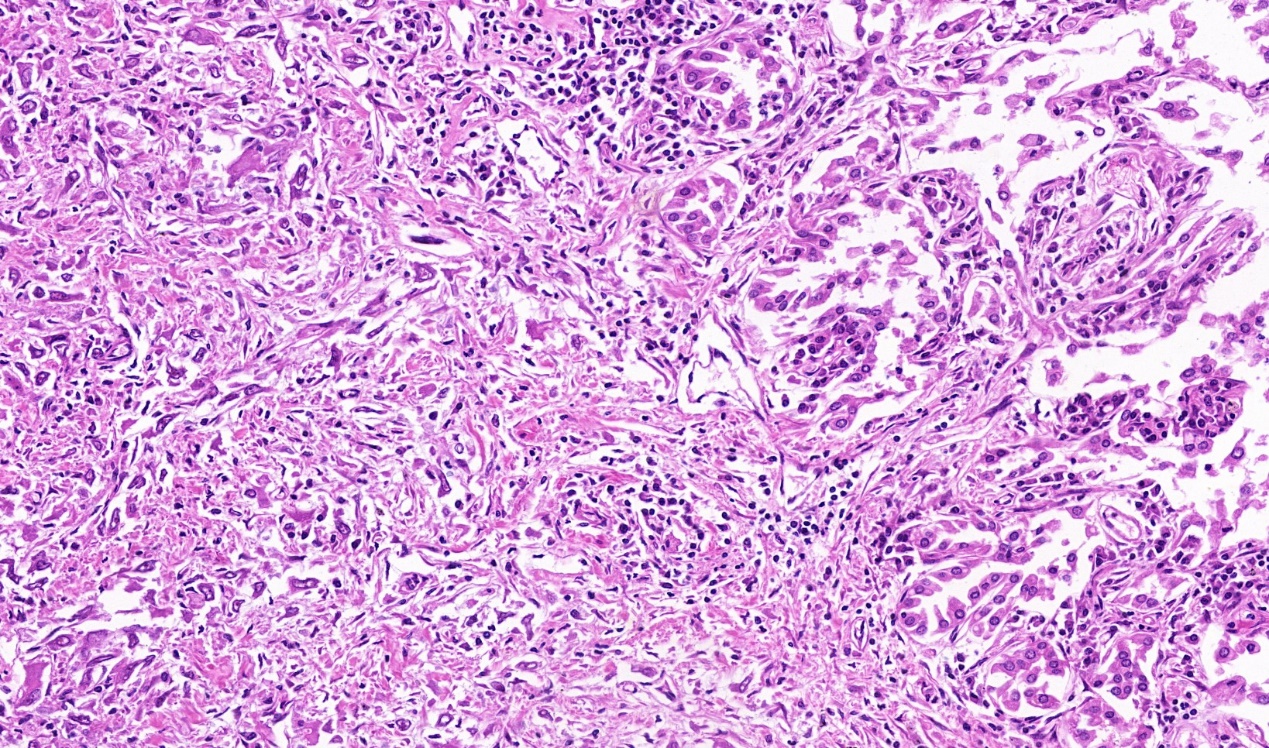


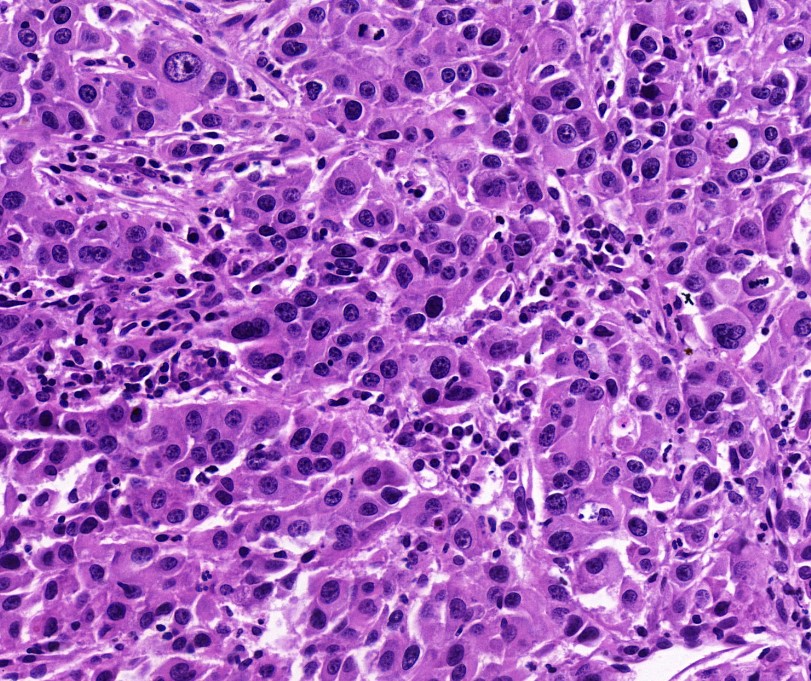

Supplement: Supplementary file 10 [file Table_3.docx]
