# Supplementary material for: Characteristics and Prognostic Analysis of 55 Patients With Pulmonary Sarcomatoid Carcinoma
Source: Front Oncol. 2022 May 3;12:833486. doi: 10.3389/fonc.2022.833486 (PMC9113756; doi:10.3389/fonc.2022.833486)
Supplement: Supplementary file 8 [file Table_1.doc]

KM time BY sex
  /STATUS=status(1)
  /PRINT TABLE MEAN
  /PLOT SURVIVAL
  /TEST LOGRANK
  /COMPARE OVERALL POOLED.


Kaplan-Meier


附注	
创建的输出	19-JUN-2020 20:28:37	
注释		
输入	数据	E:\医学\肿瘤内科\孙佳春老师的肠癌统计数据\modified\data_modified 赋值.sav	
	活动的数据集	数据集1	
	过滤器	<none>	
	权重	<none>	
	拆分文件	<none>	
	工作数据文件中的 N 行	55	
缺失值处理	缺失的定义	用户自定义缺失值被视为缺失。	
	使用的个案	对于分析中的所有变量而言，统计量以带有有效数据的所有个案为基础。	
语法	KM time BY sex
  /STATUS=status(1)
  /PRINT TABLE MEAN
  /PLOT SURVIVAL
  /TEST LOGRANK
  /COMPARE OVERALL POOLED.	
资源	处理器时间	00:00:00.41	
	已用时间	00:00:00.41	


[数据集1] E:\医学\肿瘤内科\孙佳春老师的肠癌统计数据\modified\data_modified 赋值.sav


个案处理摘要	
性别	总数	事件数	删失	
			N	百分比	
女性	13	13	0	0.0%	
男性	42	36	6	14.3%	
整体	55	49	6	10.9%	


生存表	
性别	时间	状态	此时生存的累积比例	累积事件数	剩余个案数	
			估计	标准误			
女性	1	1.000	死亡	.923	.074	1	12	
	2	6.000	死亡	.	.	2	11	
	3	6.000	死亡	.769	.117	3	10	
	4	7.000	死亡	.692	.128	4	9	
	5	8.000	死亡	.615	.135	5	8	
	6	9.000	死亡	.538	.138	6	7	
	7	12.000	死亡	.462	.138	7	6	
	8	14.000	死亡	.385	.135	8	5	
	9	23.000	死亡	.	.	9	4	
	10	23.000	死亡	.231	.117	10	3	
	11	24.000	死亡	.154	.100	11	2	
	12	26.000	死亡	.077	.074	12	1	
	13	57.000	死亡	.000	.000	13	0	
男性	1	1.000	死亡	.976	.024	1	41	
	2	1.000	删失	.	.	1	40	
	3	2.000	死亡	.952	.033	2	39	
	4	3.000	死亡	.	.	3	38	
	5	3.000	死亡	.	.	4	37	
	6	3.000	死亡	.879	.051	5	36	
	7	3.000	删失	.	.	5	35	
	8	4.000	死亡	.	.	6	34	
	9	4.000	死亡	.828	.059	7	33	
	10	4.000	删失	.	.	7	32	
	11	5.000	死亡	.802	.063	8	31	
	12	6.000	死亡	.	.	9	30	
	13	6.000	死亡	.751	.068	10	29	
	14	6.000	删失	.	.	10	28	
	15	7.000	死亡	.724	.071	11	27	
	16	8.000	死亡	.	.	12	26	
	17	8.000	死亡	.670	.075	13	25	
	18	9.000	死亡	.643	.077	14	24	
	19	10.000	死亡	.617	.078	15	23	
	20	11.000	死亡	.590	.079	16	22	
	21	12.000	死亡	.563	.080	17	21	
	22	13.000	死亡	.536	.081	18	20	
	23	14.000	删失	.	.	18	19	
	24	15.000	死亡	.	.	19	18	
	25	15.000	死亡	.	.	20	17	
	26	15.000	死亡	.	.	21	16	
	27	15.000	死亡	.423	.081	22	15	
	28	16.000	死亡	.395	.080	23	14	
	29	20.000	死亡	.	.	24	13	
	30	20.000	死亡	.	.	25	12	
	31	20.000	死亡	.310	.077	26	11	
	32	21.000	死亡	.282	.075	27	10	
	33	22.000	死亡	.254	.072	28	9	
	34	23.000	死亡	.226	.070	29	8	
	35	23.000	删失	.	.	29	7	
	36	24.000	死亡	.194	.067	30	6	
	37	29.000	死亡	.161	.063	31	5	
	38	31.000	死亡	.129	.058	32	4	
	39	36.000	死亡	.097	.052	33	3	
	40	39.000	死亡	.065	.043	34	2	
	41	58.000	死亡	.032	.031	35	1	
	42	87.000	死亡	.000	.000	36	0	


生存表的均值和中位数	
性别	均值a	中位数	
	估计	标准误	95% 置信区间	估计	标准误	95% 置信区间	
			下限	上限			下限	上限	
女性	16.615	4.064	8.649	24.582	12.000	3.595	4.954	19.046	
男性	18.530	3.008	12.634	24.427	15.000	1.740	11.589	18.411	
整体	17.983	2.422	13.235	22.731	14.000	1.724	10.620	17.380	

a. 如果估计值已删失，那么它将限制为最长的生存时间。	


整体比较	
	卡方	df	Sig.	
Log Rank (Mantel-Cox)	.120	1	.729	

为 性别 的不同水平检验生存分布等同性。	

Väø/e]ïeýÒ[ßü®¯²X+[5yyyjpéÒ%ãgÝH8e½´9ÊUË§×ë-,,Tµe×_»r¾ýÎ?PVk®¬µµµ%%%åååiiiáG1òY7>¢ÎÊ-È©¾¬N§³¹¹Yÿ:«"Y<sæSUÊJY°¬ZA>,ÍóûýËºàë¬êµX)kqq±*ëðð°LvCÊÚ××wìØ1µ9??¦RÖPýJÕ-,[[ÓSÛÏö¥øæYÊ`­URZZZÚÕÕ¥²·eËhjÆøY7@ ªªj``àùçw»Ý£££RVNOOë?<ÇçóÉ¿¥mkkÛ»wooo/e¥¬ËºëçVUVSVª¬1ÝpvvÖåri»/i&&&:::´³1~Ö×ëë455]¾|YnAæ¾ýýýsÁwÊªÏTUofÕóx<¢¬5~ß`Êà²e¥¬5Lå­¼ñÞUÊà5óÆ²¡¬zÀ#,«<ÅþÜ¨ÞrªWTT¤U#òSVÊ².¢¬¹¹¹*¨j vYÒ_a#òSVÊ²ÆHËäP***ôû	sD~ÊJY¿¬Sþãûo§ (þÿ«nÌÉÉ	³êÞÀù)+e°.Êúÿ?¥_ÿÛùyréËþWôÏCìv»zyµ»»[­áü²`kðâ¶ûý~mÇ¥Ý»w«Ass³Û9ÈOY)+ÊYµÔMLL¨#%	§ÓYWWþB,Gä§¬eDq``@¹Ôçóu#òSVÊ²ÆÂf³©^z½ÞÑÑQýôç8"?e¥¬(ëKïåYqvv¶££C²ª5RÖäææJõWãü²Xïee¥¬@Y)+e¥¬(+(ëõþîß¾ëWõ­ß¿­ÿØo)+ÊÊºho¼wußµ#j²¦¶=/¾âþ;Ê²².ÏÖàWýâç(+ÊÊJYPVPVÊ²²²¬Ý#>Ê²².?Ê²²RV² ¬ ¬åõz-K\[[þ¢öööÍ7«e@YAYfµZív»ª««333õ%$$Ê@NWUY·¶¦KÿIÑÖ7y^@Y±ZÊ*ùTcBòùäOÏ?GWÆ2(..~êA²^&µóßì·Wµüç7¾ðç+</ ¬X-e.F«ÅñññòSÇ#kÿþA%%%7n|_h(¦¬(+VQYM&66Íú¶oß®>^»cÇÕ³5² ¬X½eMLLjk°cÎRV5²¬¬,Í&9µZ­údêóùdÐÓÓ#óWÊ²².Ìår%%%L&Åâv»?üV²;wîHPe¶*§2¦¬(+(ë£¬(+(+eÊJY)+e@YAY)+ÊÊJYPVPÖuç_ýõåêFg¿,?m¿ÍÊÊúP²[ËÿðåÓÕ7dIËz'õ¡ß<÷ªß¡Æee¥¬(+(ëj*ëßýÎ;ee]íÊùö;ÿ@YPVPVÊ²²®²~§§²øæ9Y¶¶¦Ë"%µîk<A ¬ ¬KÑøoo¼êwÈ"Yýë¹2mEÆ<A ¬ ¬E¿o0e@YAY)+ÊÊºÊúÃÛ¯º<ee]~ee¥¬(+(+e@YAY)+PVÊJY)+ÊÊúHËí9.Ë:¿YòÎ?òd²RVÊúPv~ß5ä¥êMâÊÊJY)ëCÑÈ/q+O(+e¥¬ee¥¬(+(+eÊJY)+e@YAY)+ÊÊJYPVPVÊJYPVPVÊ²²®æ²º»ÞåÇ7Ú¾â*âÉe¥¬õ¡|åÅ7^(»*Ë-þþ3ÿüu,PVÊJYÌYÿªá<Y ¬²RV² ¬ ¬(+e¥¬ee¥¬(+(«Êº³ék·ïúÕrw|'²RÖ¥ûç_Ú¾ïÚYdðªßÁÊJY)ëÒ¹»Þ¡ìªß<GYPVÊJY)+ÊÊJYPVPVÊ²RVÊ²²RV² ¬ ¬² ¬ ¬+VÖcÝåW×G-< ¬²>TY/þú×¿í9.ËÖÖtAPVÊJYª¬ze¥¬² ¬ ¬ee¥¬(+(+e¥¬(+(+e@YAY)+ÊÊJY)+ÊJYðe6<ûì³Â455ÉEgÏ¥¬ú²VÝ²©¥ñßÞàÙe¥¬Ë¡Ær¶··W»èÒ¥Kë°¬¿¸9õº¶ü´ý¶Zñ×¯¨¬fï»vg²F.«h:sæ³³³µ9«ÃáXeÕ;]£ÑÙ²Ò5ä¸òl²RVÊJYPV¬|Y+**d0;;«ß2,êëë''')+e@Y)ëâHSkjjâââ;FGGõ¯¶Fçõz-|mJJJ[[þ¢ééé³ÙüÄO´··SVë¢¬jðùóçe'ãÉm,_nµZív»ª««333õ9sFnG²úäOÊ×^íä6nÜHYPV§¬ããã2w4&%%µ´´¨1¾å&!!A5X&»ÉÉÉúd2ñ=úôç*ó]Ê²Â8eU-	zÖÔÔÈär÷îÝ2y³²2+++úK#ÕÙÊÊÊøøx¹Íîîn¶ ¬Xe©jEE<,Ün·Ú¡Éãñ¸¶Jëêê¢¹ÉdÒÆf³9ä¢Ë/Ë ¯¯Oæ¦eÅzÙéäÉò°É«VÖééé¢¢"ì.111¨­Á2¹h¾é¬Êºµ5½øæ9YÞ.õ¾ÿÏ,eeý###òxî¹çîÝ»×ØØ¨öfjjjR^¹rEÎÖÖÖF¹¬¬,Í&9µZ­úòóóÕ×ú|¾;w©¬Ã÷G^õ;ÔòÌ_SYÊÊ:×ÛÛ Ç§>õ)ýu.033åFWRRÉd²X,j²øªÝ 222d¶Úßßo¤²êÉ´²QÖÕ² ¬0ZYeJZ__/´´4ÊJYPV<ÔÖàõî¯×+kîÜ¹³iÓ¦k*®:ö!e¥¬(+eÉää¤<&Ô"îÝ»²?°­ªª¢¬e¥¬MIIY©ÚX½¥ÕãñÈigg§¾¬555² ¬uaê¨.]ÇÄÀÀ@Èü²RV².éËÔO)+e@YñPeu»Ý999êàûrª>?.%%E¿SGGer·K·¶¦ï»vDò,PV¬ß²ªpÊ©ßïW¯°j[µ+x<ÊåÃ÷Gnßõ«å7¿(§<Ëë´¬f³9+++âà¼¼¼ááá¡¡¡þ¦PV=¶RV²buppP4MHY].mnnVÍÉÉ¡¬e¥¬ ÊªR*¹q½~þºcÇÊJYPVÊ«ññqõ:kOOúúúôú|¾ÄPVF+koo¯<&úûû].WÄ+466RÖÅuøþkÈÃÓ@Y±Ëúhµ¬ÕlÏqnÊÊJY)+Ê+ëÌÌÌÔÔ×ÔÔ|ÌÕ_Öo»õú~6ß"¾Ùé§¬eeýÓéÇDee¥(«ÞûÜûÍÀh¥ðt6©ÝÚq±÷¿ÆS@Y±^Ê*øøx5¶Ûíú²Êt².ms±~ÎÊÊuTÖÙÙYy@$&&6ÍæCÉYu°~ÊJYPVÊ«ºº:ý×ÔÔX,¶SVsK>"¿ÌM)ë²Uÿ~VÊPV¬²ÊC¡¢¢B¿&¤¬ê£Ñ)ëÊªGYÊuTÖ©©©®®® ýë¬2SíÑ)+e@Y)k¬ffffggÙLYPV,OY£l¦¬e¥¬² ¬XÅeZÑý(+ÊuvvvóæÍÛ¶mkjj:$cÇÅÇÇkkÔÁ"Vî`Leqæ¬Ãj(+e(+ÖõÖ`ÊJYPV,[YzzäÁQSSò:+e¥¬(+evõªÕjÇ'´Yív»¬IKK£¬e¥¬1íÄ¤#B~°C¶SV².õ7lØ¼y3¯³RVËPVÃ!ÎÎÎçôo¹9qâe¥¬(+e]Ü6ayL>|Xá¹àG·Êú²²2ÊJYPVÊº2+ÇDKK¶ÆçóÉ³gÏ²5² ¬uqjjjªªªä1ÑÜÜ¬­looWµI-e¥¬(+eéåÕÄÄDµûÔtPYY¬±Z­2V3ÚÊJYPVÊÍààà;´=v°C­®hM)+Êï,óçÏkg)+e@Yñ°eÕï¯DY)+Êå)ëàà Ýn/,,5Ò<ÊJYPVÊºÄ²666ªñèè¨-((øx¾iõ[ç®ïûÛ&é«~)­tÛêß¥¬eÅ:-ë#d²vûdÎ²H½ä¤¬eÅº+kggçóAÖÕ××Ë¥¨¨².»ë½Ê®RV²bÝ5bzzZ½µ²²²RV².ÏçÓõz½òikk³333² ¬uqû.Ùl¶ÎÎN³Ù<÷ÑQøeÎÚÓÓ#uáêêjÊJYPVÊkY/^¼¨µµµõõõ»víêíí³!¦¬e¥¬õòåËÚYµãÒ=Ô¯² ¬ueíëë;zô¨¬)..Vk(+e@Y)ë¢ËÚÝÝív»eàt:eÎºwï^¨¾RVÊ²RÖEUªúª¶?þøã2Pq² ¬u)[z«ªªjjjÔETæ¬e¥¬«<,LUëêê<(gä¬:Ó+Wd²RV²ÆÄétêÏIÍY½^/e¥¬(+e]ÖCYÞ.æÍ/J_eñðýÊÊJY^VïûïÈU-[[Óoßõó4PV¶¬êÈÀ---ò°RÛål~~>e]²êí»v²ß7¸%HãããrÚÔÔ¤Êò,e¥¬(+e]X__:b°feeuuu©ÜîÜ¹­Áe¥¬ãv»ezùòåÒÒRõ6rZSS#§h:ö¬v	ÊJYPVÊúØó¡¡¡Aê³Zdìp8d¼iÓ¦-[¶0g¥¬(+ezmµ¢¢"77×d2¥¦¦ÊøâÅê­±löz½%...%%E^zõ".e¥¬eñË:::*þþ~¿	i¤LRå¬¿ÊÊ¼¼¼è7bµZív»ª««333Ã÷=Þ¶möóûý7$ÿÙl¦¬(+Ö|Y¥£ÚSí¾¤Z«MdïÝ»'§çÏ~;			jC.-//I°öËÉÉÙæ±Ç£¬(+0g²JPä1ñøã744È@½¥U_NÝnwô8©©©êÝ±jÍþð÷ô«_ýj]ÍY?ÿæ^õ;Xß<ak©¬ÓÓÓr*³R«.^¼h2ÔEò@ÉÈÈXðF´/ÒHýEòåªÓ¼ÎªäþâäWof/¸ìjÿLpyÂ(+ÖØLjB¹eËÇ£¡Ö766j0]bbb P[eüÀ7ô Êº(2g¾òPV¬±²*êÎÕ¥µêð'NÓöööè_e³Ùd §V«5òwÆ²ë¤¬¶óçÏoÚ´i||644TUU©c«Ýel2îÝ»7ß-¸¤¤$¹ÅbÑ^y­Ï²¦e½Þèì_òò=góÖ¿~<£«¥¬@Àét¦¦¦NMM©5½½½RGuÌYWî6jY30ú­s×OWßXòRøÃº=ÿòÍ(W8ôóË?ã ¬X[!£õá-¸5øá78 ¬ ¬²²RV²²RVÊ²²RV²RV²²RVÊ²RVÊJYPVPVÊJYÊÊJY)+@YAY)+e@YAY)+e(+(+e¥¬ee¥¬e¥¬² ¬ ¬ ¬ee¥¬ ¬²RVÊ²²RVÊPVPVÊJYÊÊJY)+ÊÊJYPVPVÊJYÊÊJY)+ÊJY)+e@YAY×YwµINÕ206HYÊÊJYÎ÷Aß¾kGdÚ*ËÖÖôª[6ÊPVPVÊº<$« ¬ ¬²²RV² ¬ ¬²²RV²RVÊ²²²²RV²²RÖ5¡úkkú¾kGd:Ò!e(+(+e]¢ÆGoßõ«å7¿(§ ¬ ¬uyÈ´²²RV²²RVÊ²RVÊJYPVPVÊJYÊÊJY)+ÊJY)+e@YAYAYÊÊJYWÌÖÖôâçªnÙ¾ýõåj~ ee¥¬å;?Q¶^ñó¯ð(+(+e]?¾ÑöWßàçPVPVÊJYÊÊJY)+ÊJY)+e@YAYAYÊÊJYßjÚÙô5~ee¥¬Ëã»?JmÉàçPVPVÊº<ÎºíÛ~ú´¬×Õb«	@YAY)ë¢ùÇ~ëòÈR|óÜÖÖt[,/4^øu.~8ee¥¬ÖàoÎöE²*Ë®[eÁ¦¿çPVPVÊºt2m¾ªqî¿	@YAY)+e(+(+e]ÞúýÛ ¬ ¬u9RV²²RÖåGYÊÊJY)+@YAY#ñz½%...%%¥­­MÛíÞ¶meË¹e¥¬ee]ÕjµÛí2¨®®ÎÌÌÔ_ôÔSOuvvÊ ¦¦æé§¦¬ ¬ ¬KHHA HNNïjñññrúâ/þ§îsI- ¬ ¬.Fëy<£GÊàêÕ«/?¨¢¢bãÆüYRV²²~Èd2ic³Ù~	«Õ:99ÉÖà-ëyã«¯ú]Þxï*?=²bu5111¨­Á2¹txx8''gddWÔ·ê3Ço[Ôû[[Óùé««¬YYY6Mr*sSýEíííöìvFùrÊº,.Õ¾#Ëb¿êö]ÿ¾kGøé««¬.+))Éd2Y,·Ûýá·|%''oÐ¡¬ ¬ ¬+²RV²²RVÊ²RVÊJY)+@YAYAYÊÊJY)+@YAY)+e@YAYAYÊÊJY)+@YAY)«qËºµ5½ê-|¾?ÂO ¬ ¬uq>UÉ­D*@YAY)ëòØwíe(+(ëz/kZÖë_.ø~Ù¤©ðte(+(+môîýß,Îþo´SV²²by¸»Þ¡l)lNYÊÊÊPVPVÊJYÊÊJY)+ÊJY)+e¥¬eee(+(+e¥¬ee¥¬5ÔÖÖô×¿í9¾¨EzÜàoæÇÊÊJY)k¨îkÈ³ØEâZuËÆ²RÖå¡æÏ²RVÊPVPVPV²²RVÊPVPVÊJY)+@Y)+ee(+(+(+@YAY)ëªÖíÊ-~²Ëã7£_.øe(+(+(+@YAY)ëº/kõ-ûÖÖô×Ì·È¥?º]Ïï²RÖÜ»×e)¾yI-(+(+e¥¬l.(+e¥¬ ¬eee(+(+e¥¬ ¬²RVÊPVPV,­¬iY¯®¾±J¯Ôeõ|?K[¾ý-<º@Y)+e]Æþý~£³õ,Î*YVÕ·´å²«W] ¬²­ÁËCâ*3W~ ¬²²RVPVPV®¬Å7ÏE?NÓê_~ä¼QüýíìÝñ1~³ ¬²âÑøÑíúè^KZë¡ÍYÚ_õ;øÍ²RVÊ,ÏÖ`SVPVÊJYÊÊÊPVPVPVÊÊÊÊJYÊÊÊPVPVPVÊÊÊÊJYÊÊJY)+@YAYAYÊÊÊJY±îÊz¬»êò°,méñQVPVÊÊú²^üõ+®=Ûse	üè¶¶¦SVPVÊÊÊ§È-Ûwýû®¡¬ ¬²RVÊJY)+@Y)+ee(+e¥¬ ¬²²RVPV²RVÊÊJYAYAYAYcàõz-K\[[[QVPVÊJYAY#³Z­v»]ÕÕÕ1^DYAY)+ee,!!avvV@ 999úE?þñ¿ñ #GlÜ¸gPVÊJYAY?qñ¢óçÏïPZZ³ÖnßÄÃÃû`|´ê²²F`2´±Ùlñ"¶0ÊJYSbbb P|eãEee,++Ëf³É@N­VkQV52Ëd2,Ûíþð[	>È"^DYPVPÖDYPVPVÊ²RV ¬ ¬@YAY)+ÊÊJY²²ee¥¬(+(+eÊJY)+PVPV ¬ ¬e¥¬²eeÊÊCYÍfó°QVÊºÜ»wOæ¬æ¥2Lqqqf#û%÷ÎlPüâÖî/nãÆ¼k-ùË?ùÉO!ÊjO?ýôw¿û]CÞ5ÍöéOÚ¨¿¸Ï|æ3?øÁy×Î9óÔSOò®9CÞ»ÂÂÂÝ»wPVÊJY)+e¥¬ ¬²RVÊJYAY)+e¥¬²²RVÊJY)+e¥¬ ¬²RVÊJYAY!yv¾qã!ïÏçûÞ÷¾gÔ_Õ»ï¾kÈ»æñx._¾lÈ»öç?ÿùÜ¹s~¿ß÷®½½½¶¶²@Y ¬«×ëµX,qqq)))mmm¸Gn·Û¶mr¶lÙ"÷Î÷±¥¥E;aîÝôôtNNÙl~â'ÚÛÛö´yófu_1îÝØØXrrr'ãýé²ÆÄjµÚívTWWgffà=õÔS2¨©©yúé§wgffä¿ZYsïÊÊÊÎ93;;+zòÉ'öKHHª­õ'ÿ?ÿ+èó~÷ôÊë¼<Í÷WÔÿ÷ÓâããwËËË+**´g4ÃÜ;ÖôööõÁ)ÿWªÿ7¬õ·ÿþþþ~YÃï±^@YçqlçèÑ£»2ãIMMg+íÍ0÷N¾ùÊÊJùÏ§»»Û`¿8¯×+wM~kr*LÃÜ;YÃï^@Y£1LÚØl6æ~MLLX­ÖÉÉIÝÇý3aîÜõ6¾¾>ùßÁ~qÛ·oW3rIì;sïôe¿GFze]@bbb n®±1îÔððpNNÎÈÈñîãéÞé¿y5¿1Ò/.|öf§/kø=2äÓ(ëÂ²²²l6ÛXE2É3À=jooß³gÏØØï£þÍ0÷.??_aÀçóíÜ¹Ó`¿8§ÊýAOOÌ_sïôe¿GüÓe]ËåJJJ2LÅívà%''ÌêwõÏh¹wããã2KMMíïï7Ø/îÎ;T¹wr*cÃÜ;YÃï!ÿô@Y ¬PV@Y ¬PV(+ ¬PV(+ ¬PV(+ ¬Xfff¦¦¦Ô¸¦¦FÿA"ÑõôôÈåKô+µZøA¿aCÄùõ¥¥¥+û÷¶aCZZZSSÓ¡ÈXVfggóx(+ð°N§D¥²²r.øqxb¾kvuue©IåO8¡ÅÉn·«h©ëÇÇÇïÚµKÖ×ÕÕÉúô5çÏÿWä"ù·:::2BúÒò(ü~Äÿ(ÈÊÝ»w¯Ü»woøJõ@YELà$j,iÔU¦³úkÎ©ñØØ|aEEEô[V'>99)ãK/ Ý v5Ï'Åõ2ýÕ_ªUôTãp8äÊG½¬YÙÙÙ)ë¥î<NÊÄDÒ%åHLLl***2Í%%%Úq¾Ã²~óæÍ6[_Vkfi7^]]-§E]?ÈYàÊØd2mß¾½>H¾½ýû÷Gép¹ÍS§NÍ÷ð­ÁáWîííeC1@YXIÆªªª´³?­sNe>÷>N'N¯¬/_ÖÏAÕU³ú²ê·*ËxË-±UÝÎÁ£$?âßð9«ñeÜ)â¢Êª¦¹ÑR½ZVV25Iç+Wd066¦©uÁ²>ûì³Ú5wíÚYå_«õ÷÷G¹×Ú$8²ªKSRRxÀX «!¯YURþU2+/liiÑÖø|>YsöìÙðÛ×ÏY%ujo)ËKYUU&³²²b/kØK³Ø÷@Y±~Ë:55¥íñ«UÆª%µµµ!é­ªªõÍÍÍÚÊöövµqXÔF,«k­Ü5??_®&ÿhôôÆø:kµ@Yé7êFßìp8Uf¤¦ÛdNYVV¦Þ*c5£íéé¯¬Z«V¨¬ò¿õ¾(,//_Ô5=¶PV T²JwìØ¡eÆãñhÛÕ[STM#NÕK°>/äÒÚ¬Þ$3Ñùþ'ñ]ªQÊªÞbòÝ ¬ÀC5$Bú¼E)«±³:ÎE5//O»æ±cÇb/ë¹ÚMP±¬ªÄ©©©<<Ê,CY§¦¦Â÷cÙ_)JY¯²rbbbûöí¦¤ÊòÊ®öm466¶´´¨l,÷E½r DµcsÄ£'F,«×ëõO?ý4²ÆlÞ¼yÛ¶mÚ<2©a|||qq±~¿r0&­¬2;,,,Toªý¸CPÄ©­fttT.üñÇc¿n·;ä_	ßM©»»»¢¢";;;âÿdeQQ²1ø¦¯6ÈR¼(Õ®¢?³.^¼ñ¢K.Å8@Y ¬²@Y ¬²@Y ¬²@Y ¬PV@Y ¬PVîÿ³èù©¸IEND®B`


KM time BY age
  /STATUS=status(1)
  /PRINT TABLE MEAN
  /PLOT SURVIVAL
  /TEST LOGRANK
  /COMPARE OVERALL POOLED.


Kaplan-Meier


附注	
创建的输出	19-JUN-2020 20:33:36	
注释		
输入	数据	E:\医学\肿瘤内科\孙佳春老师的肠癌统计数据\modified\data_modified 赋值.sav	
	活动的数据集	数据集1	
	过滤器	<none>	
	权重	<none>	
	拆分文件	<none>	
	工作数据文件中的 N 行	55	
缺失值处理	缺失的定义	用户自定义缺失值被视为缺失。	
	使用的个案	对于分析中的所有变量而言，统计量以带有有效数据的所有个案为基础。	
语法	KM time BY age
  /STATUS=status(1)
  /PRINT TABLE MEAN
  /PLOT SURVIVAL
  /TEST LOGRANK
  /COMPARE OVERALL POOLED.	
资源	处理器时间	00:00:00.34	
	已用时间	00:00:00.32	


[数据集1] E:\医学\肿瘤内科\孙佳春老师的肠癌统计数据\modified\data_modified 赋值.sav


个案处理摘要	
年龄	总数	事件数	删失	
			N	百分比	
≤65岁	24	21	3	12.5%	
>65岁	31	28	3	9.7%	
整体	55	49	6	10.9%	


生存表	
年龄	时间	状态	此时生存的累积比例	累积事件数	剩余个案数	
			估计	标准误			
≤65岁	1	1.000	删失	.	.	0	23	
	2	3.000	死亡	.	.	1	22	
	3	3.000	死亡	.913	.059	2	21	
	4	3.000	删失	.	.	2	20	
	5	4.000	死亡	.867	.071	3	19	
	6	6.000	死亡	.	.	4	18	
	7	6.000	死亡	.	.	5	17	
	8	6.000	死亡	.730	.094	6	16	
	9	6.000	删失	.	.	6	15	
	10	7.000	死亡	.	.	7	14	
	11	7.000	死亡	.633	.104	8	13	
	12	8.000	死亡	.	.	9	12	
	13	8.000	死亡	.536	.108	10	11	
	14	11.000	死亡	.487	.109	11	10	
	15	12.000	死亡	.	.	12	9	
	16	12.000	死亡	.390	.107	13	8	
	17	15.000	死亡	.	.	14	7	
	18	15.000	死亡	.292	.100	15	6	
	19	16.000	死亡	.243	.094	16	5	
	20	23.000	死亡	.195	.087	17	4	
	21	24.000	死亡	.146	.078	18	3	
	22	26.000	死亡	.097	.065	19	2	
	23	36.000	死亡	.049	.047	20	1	
	24	87.000	死亡	.000	.000	21	0	
>65岁	1	1.000	死亡	.	.	1	30	
	2	1.000	死亡	.935	.044	2	29	
	3	2.000	死亡	.903	.053	3	28	
	4	3.000	死亡	.871	.060	4	27	
	5	4.000	死亡	.839	.066	5	26	
	6	4.000	删失	.	.	5	25	
	7	5.000	死亡	.805	.071	6	24	
	8	6.000	死亡	.772	.076	7	23	
	9	8.000	死亡	.738	.080	8	22	
	10	9.000	死亡	.	.	9	21	
	11	9.000	死亡	.671	.085	10	20	
	12	10.000	死亡	.637	.087	11	19	
	13	13.000	死亡	.604	.089	12	18	
	14	14.000	死亡	.570	.090	13	17	
	15	14.000	删失	.	.	13	16	
	16	15.000	死亡	.	.	14	15	
	17	15.000	死亡	.499	.092	15	14	
	18	20.000	死亡	.	.	16	13	
	19	20.000	死亡	.	.	17	12	
	20	20.000	死亡	.392	.091	18	11	
	21	21.000	死亡	.356	.089	19	10	
	22	22.000	死亡	.321	.087	20	9	
	23	23.000	死亡	.	.	21	8	
	24	23.000	死亡	.250	.081	22	7	
	25	23.000	删失	.	.	22	6	
	26	24.000	死亡	.208	.077	23	5	
	27	29.000	死亡	.166	.072	24	4	
	28	31.000	死亡	.125	.065	25	3	
	29	39.000	死亡	.083	.055	26	2	
	30	57.000	死亡	.042	.040	27	1	
	31	58.000	死亡	.000	.000	28	0	


生存表的均值和中位数	
年龄	均值a	中位数	
	估计	标准误	95% 置信区间	估计	标准误	95% 置信区间	
			下限	上限			下限	上限	
≤65岁	16.215	4.066	8.245	24.185	11.000	2.235	6.618	15.382	
>65岁	19.115	2.887	13.456	24.775	15.000	3.096	8.932	21.068	
整体	17.983	2.422	13.235	22.731	14.000	1.724	10.620	17.380	

a. 如果估计值已删失，那么它将限制为最长的生存时间。	


整体比较	
	卡方	df	Sig.	
Log Rank (Mantel-Cox)	.510	1	.475	

为 年龄 的不同水平检验生存分布等同性。	

°|bø'²@Y ¬²@Y ¬²@Y ¬PV@Y ¬PV@Y§õÛóâ/.êþËþmÌÍÍiÏ_\,W'&&äjcc£,éïïç'PV`õêììÔòôéÓ*ë¿üå £££ú«===çYCÔ××Ë3ØíöÐ§MOOW3n·ûòåË2355%7näGPV`õ,-ieeeÝYßHâ¹sçäþ;wîÔ²¤¤DÊ¼ö¨@ úl1óº»»nå^¯W.[[[µqªÌTUUñS(+°zÉxQ¿5¨¬cccú;ÏÎÎjûiÕðE'&&NgllìÐÐPè­Gíp8Ô¶¸¸Xj*3íííjç³JøñãÇåêÀÀ?D²«é×iJÛ7»uëV5_WW§n]hü*7¥¥¥îÝ½xñbèýe`zçÎífffÞßôúõëA÷1®ô[¹2>|X.ËÍfsRRöjw1Ê¬"2TUodêCao°¦··WîYRR¢§9ö¬,Ë xïÙ³Gb©÷T¦2%qíèèËMrY]]->O.µçâò(+°ê¬åååAK¢)kèD###N§S¿6èÎ2úææfµÿV.+**äRë¤ª^Õ[¿rõX¯×ÛÖÖÖÞÞ.1«ê á(¿OxrÔ¸Sÿ¾iP±dà¨2DýéÚ«àBûeù®]»îÏÿ-M]]ßï%±±±ÚdÚW¬]¸jÅUßU=³eFBÎ ¬Àê°J´$TóBßgU±Ñ?ª««+'''ì`w¡7>µ²ª"ÊTuQíO*«èèè8xð ö¨uÏÚÚZÉ­zcÊ¬FyaÇ¬¡ÜAî/9GfÍSñ3Íõó:$Wµã~UU&§¦¦îÿéVI©ÌÄÇÇUÁÔÝÝ­v«?c­¬¬ÔxW½ËÏ ¬ÀÚÈ.TV)¥äP»saÌZSS¢_ráÂ-ÕjüªU»DW½çª-yY"äPV`m5ènê ¤(ËªöK/^¼«O£OÆ¬;v¨Aªv«=èxfXKeúÓ%U©®®nkkÓß!lYÕVêê­Yµ[X¿êÜO2ÃÏ ¬À*ÒÐÐö,¾©©©ÚÕN¡/«ÜÇápÈC~õ¦¬v2°§.,,;¨ó=Éý[[[å8~ü¸þyúúúôç ü«§æG	PV`Ux&mU>_Ø;Ë3èã ---òðÐ±2::*=/X²@Y ¬²@Y£ðûßÿÞ	k¢¬«ÅØØÉd²ÀZ¶Ðç.²>²~îsûß°QVÊJY²²ee¥¬(+(+eÊÊ² ¬Q*++kllp@  ¥¥¥úOÌÏÏ_è3!@Y`ýujj*55µ5Ä/¾X^^®î#Ýu8¹¹¹ÍÍÍêvíÚÕÔÔD )+PÖªy»Ý6²PZ§¯Âív_¼x±ººÏë¥¬@Yÿ¬­­­««K»¶¬CCCEEEàýû÷744=¶½½½¸¸ØãñË@FRV ¬411áóùúúú$¨jpAATfÊËËµdÎÍÍÍÎÎÊL]]TVfúûû[ZZ+**dfÏ=ò$²cÖ?òûýÇ¼7¸¶¶vdd$;;;HP%½ª¸2~c¢¬@Yÿl||üe=räÊvUæ§§§þ#)+PÖ?o$%N§s÷îÝ*yyyEEEjÞáp¤¥¥Éä^³^¼xQ²ª2¬eÄfee©û²cÖûÚ±¾Ç¬RÓºº:¹sAA*k¿ºtI;®uq®¾ëç×!÷GYV¿ß¯Þ[UeíîîÑm[[¤¬Ka»Â¯7u[Ö9r¤²²Òétîß¿_-t»ÝiiiÒZ¹ÆÊ*uLJJ]îõz-KlllJJJ;e@Y_½l]CwttLNNj_mùÙ³ghäZ*«ËåJNNûª²Z­Cfªªª²³³eæÓO?íØï½g6)+ÊÊúÀÞ½ûûûÃ¾ªâããÕ.@  µ9991!yæ%¿Tý7	jäéÍÛüÚ ¬Xcï³UÅÆÆÍ?ù1ëªkmýüÚ ¬0BYM&6/ù|*ï³RVÆ)kBBú3,¹yÊ²²>VYm6Ýn¿?¸Õj]é²îøñ·(+ÊcU]u¹&Éb±¸Ýî.ë¶«û(+ÊuÉ(+ÊÊJY²RVÊ² ¬K044¤fÕçÇi'>e¥¬RÖ#¯¶¿|öÝ°ÜTû·ù333999f³ù¹çëèè%###úSÚéÿ4CïøñãjÝnïîî¦²0NY3lWzúÆÂNç«uæ¿°Ð¥êVKKKËÊÊäªduÓ¦M÷çÏÎ/­½ÿîÝ»[çíÝ»WÍSJÊJYª¬Ýô× Ä5tùàà`qqqZZZmm­éííÕßA²ÚØØú@QyTWWZâ÷û'&&(%e¥¬ÖcYeÐ)½Ü¿ÿd¸©-­¨¨«Úµ»yóæÌÌLYÚ××§ÎÉÉÉíÛ··´´¨1kUUÕÌÌ¥¤¬Àz,«4<(CÕýL¦JJ©[·nèmÝ#G´eÙ³gcVÊJY¬±²Úß¸õ7ù¿XhPÖö÷>ùÚ+WCï=ú´´¸¸X5Rvý)ú>±Ûí²uµZ­Òæììl+¤¬À+ëïff?^h»÷»Ã©éÿ·ÐM­­­/_'N¨7}>_zzºÚì÷ûÕ533SÝßëõª®ÌHYsss9*²RVÜ½»té,ÈÊÊRïªª£=Orr²,ÙµkWè¡¿===c^PVÊ`ýõ1uuu¹Ã¡ÊJYPVPVÊ²²RV ¬ ¬eñSÖå/ëëþf5]ûÍ¯(+UUÖ·~uÏ·ÐôÉô§=ñSÖ§VÖÝgné/~ùWyPV«ª¬ò¿ÿ¯ÿmØInzåæÈø)ëS+«FÆ¬ÒWÊ`µu¡þqøßé­Póééé555@@»ñSVÊ².½¬§N±i~~¾Ïç»Ïø)+e@Y§¬Êìì¬U=-'â§¬««¬g:íõòkà	Õý/¿úÿ"5ë½oªùÞÞÞ 1+'â§¬««¬¶[_»ø>¿özYÿþßXhKøÉô§òXíV	¤þVNÄOY)+ö/'NÄOYWEY_íúþG£25÷ÍJß~¿û|ÃüþXse'â§¬+[Öÿõg/ä½%SíLy¸Q&á÷QËÊú¦¬(+(+e`²ötPQÖ5mW÷UÞ²Ëtô|]M³²xêeM¡¢¬kµ¬¾ÏúTVeú×W²ÚÛLY¬«²Fÿ¡7ýýý­­­ííí2#jù©S§¦§§î­ÿs ÊºÊªWtã¼a)+u%>ôÆétNMMååå]ºti||¼¾¾Þãñtvvj.ËívËÌ±cÇ³RVÊ`ÍU©þu%>ô&´¬òärYO8QWW'333Õ³åææÊ£(+e¥¬V¶¬|8êøyßBl"Ü*ÝB§¥¥©s0­ÄÞ<²¬Òéªª*5Ï²RVk²¬3éåþýû8 Ó/ûÞHåkÉ¸JV­V«ÜS+«|'òÔhCa¹ç#Gfgg)+e¥¬ÖÀÞ`h<xPª4õv©²ìz#O.cÖ@ aÌªåÓëõæçç«÷w³RVÊ`-U;¾÷ôéÓRÐââbÕÈeùÐ ÃÎ=+ú²¶µµÉHWÿ>«"Yg+++[CUÊJY°¬A^¾|ùþÊ|èÍ¹sçTYTYå©$ÃAeQòÉ'Õo²RVÊ`-µ¤¤äÀÃd@y>ô&TVVÊX¾Ûí²ÊtffæÈ#ÚÝ$äò-iWÛÛÛ÷ìÙt,e¥¬ð¤ËúÄÎnýÞ¨ÑmSSSMMÍääd~~¾vsssii©jygg§úcV=)úáÃµóQPVÊO¡¬ ¬)kÞ%Û®î;æyU¦Ô7lüþ ¬ ¬åÖèm×GMØÆ¶þÐi`ð.Û+]Öìk/*Êj²êIYÏT]2lW´+«lVzÊø)ëS(kèBý¸À*,+'â§¬e]Î²r"~ÊJY¬Ó²Nÿáwþnt¡I6Aný?³ÿWmÓÓÓkjjô_9?e¥¬ÖiYäÿï~¡I6Aný¡ÿ§j«Ø××wêÔ)æççû|¾û²RVì~ü½ÁR/)«zZNÄOY)+Êºô²öööY9?e¥¬(ëÒË.Ô¿ÏÊø)+e@YóØ`NÄOY)+ÊÆ+7Ï¬èæñSÖ'TÖöA×óïU5µýL2sâbÊàIÕ e½÷Û·ïúÕ$Aï7ÈôÚU'o¼FYPVPÖåÙüº¿¹èÆyÊ²²RVuÕõïn¿NYPVPÖåGYPVPVÊ²²RV²ee¥¬(+(+e@YAY)+PVÊº²¼^¯ÅbMIIioo×ßÔÑÑ¡>Anr¹ª¬Û®î¸Ê´ïç¯þíÊÕRV«Õêp8d¦ªª*;;[S||üàà ÌÈeRRÒê)ëí»~«þÍ/¾ú¶²]@Y±ZÊ*ùTµò¹iÓ&õÁr)ó2STT´ùa²µOñ÷á«EeÅ**«t1ì¼ÚQ'/8¹ôx<²¤¥¥å+..Þ°ae@YAY0LÚ¼ÙlÖß´cÇõéóØ´´´Õ³7² ¬X½eMHHjo°ÌG9¥¬(+(kx6Ín·Ë­VýM2Nõù|2ÓÓÓ#ãWÊ²²>ËåJLL4LÅív?øVæ_dwîÜ ÊhU.e² ¬ ¬+² ¬ ¬(+e¥¬ee¥¬(+(ë+ë»ZêòM¾ÏúØX ¬².ÚáÚïg^ýú1Ï«úigÇ_?ÿÎQ6(+e¥¬öSädÌ*ec²RVÊJYPVPVÊ²²RV ¬²RV² ¬ ¬ee¥¬ee¥¬(+(+e@YAY[Ö3öú)+ÊÊº<l/·¾vñý ïýæmW÷iSãÿú²RÖ¥U¯èÆù×ýÍl8PVÊJY#¼ûÑÀ¨L_Íó¥Ò·ßïþD&_ß0e@YAYâð+m/ä½%SíLy¸Q&¡¬(+(ëcÑLYPVPVÊ²²®¦²]|² ¬ ¬Ëfôî½±¹GYPVPÖåGYPVPVÊ²²RV²e¥¬² ¬ ¬ee¥¬(+(+e¥¬(+(+e@YAY)+ÊÊJY)+ÊJY)+e@YAY)+ÊÊJY²RVÊJYPVPVÊ²²ª¬/_ÿî1Ï«Wü-lAPVÊJY«¬T«Úvu[²RÖÇ*«e@Y)+e¥¬(+(+e@YAY)+ÊÊJY)+ÊÊJYPVPVÊ²²RVÊ²RVÊJYPVPVÊ²²®Ó²Þ¾ë<±(+(+e]DYçhI¥­@YAY)ë²V×ÞTÓ·ðWÊPVPVÊú½þCÕJß~!ï-Ê²RVÊº<Ü×?¸RV²RVÊ²²RV²RV².ð°Ý»wÑÔÔ$7;w²RV².®¬YYYj¾««K®öööj7UWWSVÊ²RÖÅU4Í+++ùcÇicÖææfÊJYPVÊJY)+Ê§WÖòòrÓïõõõSSS² ¬uq¤©N§366öäÉ@`ttTÿnkd^¯×b±ÈcSRRÚÛÛõ7ÍÌÌäääÍæç®££c=uÛÕßýÆ1Ï«Ýõ­âßgsPV¬Ç²644¨Â.ùÜÜ/..ÜFóp«Õêp8d¦ªª*;;[SiiiYY<duÓ¦M²ä7Þ8õ°¼¼¼6¦¬ÞOoº<2UÞ²KÜë±¬2v4&&&¶¶¶ªQþÉM||¼j°vô7É(6hàæÌû°ÔÔTï¦¬«Üë±¬ê¨%!CO§Ó)Ë]»vÉàU®ÊBÍùáÒÅ°óêjEEE\<gww÷zØLYÊÊúÇ½¸åååò²p»Ýê&Çãr¹dØ*9¬««üpÉ¤ÍÍæ jjjd¦¯¯OÆ¦eÅz9éÔ©Sò²Á«VÖ:()TBBB Pe>è¦³e1Ë:22"/ýû÷766ª£Ô­/_«µµµÁf³ÙívK«Õª¿éÄê±>/==² ¬0xYãããµ«òâøÂ¾ ¿ÏÅggg#<ËåJLL4LEíOV_uTVVVSSSûûû)+Êõ²7ø)¢¬(+VVÖ××ËË"##².WYöQÓîN²bÝínmmU]ãõzeÉ;w6nÜe§«ÎHYÅÞ_·ó¬lnÊõ8f×:AÄøøxÐñÀrµ²²²RV²>ÂÜÜJÕÀêOZ=éËêt:)kÚ]·ì<µáÿeÛÕÇ<¯ªéýO»ÙôÆ³ª¿C­®®×ÄÀÀ@Ðø²>NY¿víe­¬2óÓ®°é(+ÖËÞ`ujCõ§u¹öë.ºqþu3²b]Õívçää¨ïË¥úü¸ýL² ¬5Ú«S.ý~¿zUÛ3¬ÝÁãñPÖÅêñIP)+@Y±¾Êj6m6[Ø=À¹¹¹ÃÃÃCCC+ýMµ¬z ¬XeTMTVË%W[ZZTbsrr(+e@Y)ë"¨²ªJne¾¾¾^?~MKK£¬e¥¬ÑPï³öôôÈL__þVÏ·¢¯Ê²Âheííí×D¿ËåÆÆFÊJYPVÊº6PV²RV².lvvvzzZÍ;Î'üú ¬(+VÖ¶¶6yMTTTÈ|Ò<Ê*eÍ°]¸.4ýÛíeß§¬(+e÷°¸¸85ïp8ôeáìú,ëèÝ×SuíM+e@Y)k°¹¹9yA$$$4Í+((0ÍÅÅÅ«êdýë°¬Ë»»²ë¨¬uuuú6w:½ÁeÅý%_Æ¦² ¬X²ÊK¡¼¼$¨¬ê£Ñ)+e@Y)k´e¾~ýzÖ<ýû¬2/Ú'¢SVÊ²RÖhÍÎÎÎÍÍ±7² ¬X²FØLY)+ÊJY)+e@Y±Ë:==½¢Ç1QV)ëÜÜòöíÛÏ'OÆÅÅiKÔÉ"VîdLeqÆ¬Oàj(+e(+ÖõÞ`Êºeùúwy^¸.vryØ`k¸¬===òâp:Aï³RÖÇ,«4Râ*#×EMßýÆóïePV¬¥²jçPo©Z­Vyqjo²:YAY§¬K#Vé²Á(+ÖRYçæi=Ù!)+ÊJYúàäädÞg¥¬(+¡¬ÍÍÍò²èêêÚ?Oÿ'75Ôû73lW^ÈK&¹ú®²õÏûå5qäÈý	ïÏt«,/--¥¬a40ª¦ü3mý ¬ ¬È¨T^­­­ÚÏ'KÎ;ÇÞàh©ºFYÊJ(ëN§³²²R^---ÚÂµsXÔRVÊ²RÖ¨Þ^MHHP/IMwÌ«¯¯/--%V«UæÕ¶§§²RV²F288¦ìñx´ÊhuEkJY)+@Yaðcå5qáÂí*e¥¬(+·¬úã(+e@Y±<et8ùùù²DGY)+ÊJYXÖÆÆF5?::*WóòòÌ7MY)+@YaÀ²>E²*kWW×yÖÕ××Ë¥  ²RV².B =ÄÌÌú3ÖÊJYPVÊº>OuttÔëõÊK¤½½]®ÎÎÎRVÊ²RÖÅ»d·Û»ººÌfóý?_Æ¬===2£N )+ÊJY£-ë¥KÔLmmmýÎ;åj~~þäädÐ))+e@Y)ë#ÊZSS£]U.effª7_§¦¦(+e@Y)ëËÚ××wüøqYRTT¤PVÊ²RÖEµ»»ÛívËL[[Y÷ìÙ#3ª¯² ¬uÑeª¾ª½ÁÏ>û¬Ì¨¸ÊBÊJYPVÊº½ÁCCC½½½N§SÝ$AeÌJYPVÊº¸ªÉ«Áb±ÈPµ®®îÐ¡Cr5>>^®ªÓ0]¾|YHq)+e@Y)kTÚÚÚôWÎÇ¤Æ¬^¯²RV²®²²RV².@¸µµU^SSSCCCj°qâe¥¬(+e]ô±Á­ódfbbB.ÔIùÞ¥¬e¥¬Ö××§Î,6íúõë*·éééì¦¬(+e]·Û-#Ôõ6rét:åòàÁMóÎ;§G²RV²F¢>ö|hh(fú¬Öonnù7nÝº1+e@Y)k´Ô«ååå/¾ø¢ÉdJMMùK.©?lf·°×ëµX,±±±)))êóÒ¨7q)+e(+_ÖÑÑQy5ô÷÷«øMNNJ#e*WÕøUæææF~«Õêp8d¦ªª*;;;ôØãíÛ·k¯9¿ßíaòµÌf3e¥¬eÅ/«tTbªÃTkµìøø¸¸p!òóÄÇÇ«n HJJºõìÙ³2Ö^s9991!yæÊJYÊ#Y¥¬Ô¦¦&yM<ûì³2£þ¤U_.Ýnwä';/SSSÕ_Çª%¿ýío?Ø¯ýkÆ¬ ¬0HYgffäRF¥W¹téÉdR7åççË%++ëO¢=DH#õ7ÉÃ;;;U§yu	e=øî7nßõ«é³Q6^eÅ8I(·nÝêñxÔi"ÔòÆÆFíæ"KHHjo°Ì?ô=Ì¨e-©pK×?0-öi»G|Û®îþ£2ÉLå-;/²bUQp®Îh(­U§(,,ËÈµÙlv»]fäÒjµÿÎ=fµ×xø¶Jß^hÊ°]yÌA­d²k£¬¶.lÜ¸qbbB®644TVVªs«ÃeÞd2/ô.+11Qîc±X´7e^dÆ.ëØ]LYÊ5PÖ@ ÐÖÖ:==­ôööJÕ9"42fo²RV²Â"ÊJYÊÊJY)+ÊJY)+e@YAY)+e(+(+e¥¬ee¥¬ee¥¬(+(+e¥¬ee¥¬« ¬E7Îkgçg+PVPVÊúXeýûÛõú³óW²²RÖeû9+e(+(+e¥¬ee¥¬e¥¬² ¬ ¬²²RV²RVÊ²²RVÊPVPVÊJYÊÊJY)+ÊÊJYPVPVÊJYÊÊJYWIY·]Ý§MÿÉÃ ¬ ¬uy¸<Ç<¯²E(+(+e¥¬ee¥¬e¥¬² ¬ ¬²²RV²²RVÊ²²RVÊPVPVÊJYÊÊJYÑß÷¼·ªkoªiôî=ÊPVPVÊºt×´¬fØ®|40JYÊÊJY^)+@YAY)+e@Y)+e¥¬(+(+e¥¬ee¥¬ ¬²RVÊ²²RVÊPVPVÊJYÊÊJY)+ÊÊJYPVPVÊºttýû%¶hee¥¬ËSVÝÎ²²E(+(+e¥¬ee¥¬e¥¬Õeýé?ÿÎQ¶]Ý'SjûdæGÿÓÉÖ ¬ ¬uÑ>½×/ÓëþæÿÐyø½ß| ÓË×¿[yËÎÖ ¬ ¬ë®¬¶+ùg:ÏT]üéÄÿøÙ¾ÇKV)+@YAY×cY¯¾ëolë_)ÿïêþ]ÝqÊPVPÖu]ÖeÔî½õµØ)+@YAY)ëòp_ÿø¥Ò·)+@YAY)+e(+(+e¥¬(+e¥¬eee(+(+e¥¬ee¥¬eee(+(+e¥¬ee¥¬ee]×eÝvu_Ñó¾ã¯XÅ¿$@Y±ºÊêõz-KlllJJJ»þ&·Û½ûv¹iëÖ­r7ÊºreõÖ÷º¿y±ê1ÿeÅê*«Õju82SUU­¿ióæÍ]]]2ãt:·lÙBYW®¬Ksû®ÿùwò/	PV¬®²ÆÇÇÏÍÍÉL HJJZènqqqrùòË/ÿ«å+_A-¿ ¬ ¬HÃÎëy<ãÇËÌÛo¿ýÃoØ°_KÊPVPÖL&6o6Cï099iµZ§¦¦Ø¼ìeÍ°]y!ï-dæõ7oQV²ÂeMHHjo°ÌÝ:<<322Â±ÁËîÞ½ß~40ª¦3U×ªkoRV²ÂeµÙlv»]fäRÆ¦ú:::233¥NYd²)«ËåJLL4LÅív?øVæ_dIII1:²uÅQVÊPVPVÊJYPVÊJY)+e(+(+(+@YAY)+e(+(+e¥¬(+(+¿¬Û®îû18Mò¯PVPVÊº8Ã÷Fò>(	ýÜVÉ­DU²²RÖåñü;G)+@YAY)+e(+(+e¥¬ee¥¬e¥¬²RV²²²²>öú3lWB§ÿüò/(+@YAY±<Ü×?~©ômÊPVPVPV²²RVÊPVPVÊJY)+@YAYAYÊÊJY)+@YAY)ëº-ë¿;Û5ä¸.û4|o(+(+e]cV«ï´íê¾Ê[v~. ¬ ¬uÝuHV)+(+(+e¥¬ ¬²bÞ7?ùfqe(+(+ÇG£/ä½EYÊÊÊPVPVÊJY)+(+(+e¥¬ ¬ ¬ ¬ee¥¬O©¬Ï½I.Øôégã²ÖÇÜÍ°]aëäkU×Þ¤¬ ¬ ¬ËC²JYAYAY)+(+@YAYAYÊÊJY)+eee¥¬XÎ²VÝrl»ºoÙ?î	O²ßé©àÇÊJY)+~YïN­Ä§©?áIÝß¾ñ=~Ü ¬²âéÕ^÷7Ý8Ï²RVPVÊÊÊÊJYAYAY)+e¥¬ ¬ ¬²²²²RVPVPVÊJY)+(+(+ee¥¬ ¬²²RVPVPVPVÊÊÊJYñç²¾Tú¶ûúÇÆ.¼W÷­wÎ~5ÌÔsë7²bAovÜ~!ï-«±'ë«þ£óÛ_Í'0~¥-Ãv²²RV¬wì^.ÊÅ(+(+eemÞvuÄU¦¼J®ýæWüPVÊJY)+°tþ±¸ªé/~ùWrÉ¿	e¥¬²ËC­²RVÊJYÊJY)+(+@Y)+ee¥¬ ¬ ¬ ¬ ¬²²²RVPVÊÊJY)+(+e(+e¥¬²²RVPV²RVÊÊJYAYAYAY×ëµX,±±±)))íííQÞDYAY)+eeÏjµ:©ªªÊÎÎò&ÊÊJY)+(kxñññsss2"ßôüä;zôèØÊ²RVÊÊú@lllØù°7]¸paÏÃ222ä&¶2Xü-ÞOoòï°4£wïU×ç_²RÖåd2´y³ÙåMì`$².§@  vùÊ|7QV5<Íf·ÛeF.­Vk7QV5<Ëh2,Ûí~ð­Ì¿ÈÂÞDYPVPÖDYPVPVÊ²RV ¬ ¬@YAY)+ÊÊJY²²ee¥¬(+(+eÊJY)+PVPV ¬ ¬e¥¬²eeÊÊEYÍfóÇ°QVÊºËÕ¼T&)66ÖlD²^²vfâ·vp6l0äª=3oÉÿüç?O(«AlÙ²åµ×^3äªÙíö/~ñFýÁéK_úñlÈU+++Û¼y³!W-ÈÈ¬¹¹Ùk¿k×.BÊJY)+e¥¬²RVÊJY)+(+e¥¬²RVPVÊJY)+e¥¬²RVÊJY)+(ëS$[çk×®rÕ|>ß~ð£þà*++?üðCC®Çã©©©1äªýá8þ¼ßï7äÚuttÔÖÖPV(+uòz½%666%%¥½½Ýkäv»·oß.k´uëVY;C®ckk«v8Ã¬ÝÌÌLNNÙl~î¹ç:::ö5JNNVëâr¹±vcccIII6&ÆûÕeÕju82SUUm5Ú¼ysWWÌ8Î-[¶ogggå¿ZY³v¥¥¥eeesss¡M6ì?88(3r©j´Ö×Nþ ÿWÐæ7t·yeö^¶e÷çWÔÿ÷Óâââ·gÏ-//×¶hY;ÖôööõÅ)ÿW¹TÿoXëk·wïÞþþ~YC×ÈØPÖÅÆÆ7Çsüøq­£xRSSek¥mÑ³vòÍWTTÈ$<ÝÝÝûÁy½^Y5ù©É¥¼2³vú²®7/ ¬L&mÞl6f½&''­VëÔÔÁÖ1++«³³S¿E3ÌÚÉ¨?³éëëÿ=ì·cÇ5"Ä¦¥¥fíôe]#£n^@Y!!!!Üß]#óÆX©áááã­cÌÃ´vúo^oô½cíôe]#Cn^@YÍf³Ùíöûóç*AÖ¨££#33sllÌÀë¨ß¢fíN8¡Î0àóùÒÓÓöqª¬ÌôôôÈøÕ0k§/kèòWõÑWbb¢Éd²X,n·Ûk4ª3Þ:ê·hY»¬¬,Ï¥¦¦ö÷÷ìwçÎ	ª¬¼aÖN_ÖÐ52ä¯(+Ê(+ÊeÊeÊe«Àìììôô´w:ú¬§§Gî,Ñ/ÔêÑ/ú°ò%ËKJJVö÷-&&##£©©éðÈ¼,<vì¯²«­­M¢RQQqþãðÄB÷¼~ýzÖ<"é¢<°°°PÃáPÑR÷Û¹s§,¯««åAé%K.úUä&ùZÙóúÔòü~Øÿ(ÈÂ]»v.Ü³gOèBõ/²ÀIÕ¼¤Q_VÎêï97OÍÉËËË#?³ú8ñ©©)ohhºõâÅÚjwóù|R.Ã_ý­Ú£"QMss³ÜùøñãÑõàÁA»ººd¹Ô×	@Y¨Hº¤			MóÌfsqq±6@ç°,ONN~d³õe±¦möäUUUri±XÔý®ÌL¦;vÔÏooïÞ½GºAÜóôéÓc"top^^^èÙQPV Z±ÊÊJíªÄOëÜ#2Û?O§ÂÂÂÊZSS£ª1«þiõeÕïUù­[·F_Võ<ü°;~CÇ¬JØwPV |c¤*«æ9rdvvV_JõfjiiiÐÐP/_±±1ý[­,ëîÝ»µîÜ¹3ú²ÊW»õ÷÷GXkmMYÕ­)))¼`Ê<"«AïYURú(Ê[[[µ%>O;w.ôùõcVI:ZÊårESVUeIÍY#ìÄ^ÚUGÔ4Êõ[Öééií_ýû¬2¯ZR[[ÞÊÊJYÞÒÒ¢-ìèèP;µAmØ²ªÁ±vôÐÊYO8!w/9½Q¾Ïe­PVàýNÝÈTf¤¦;æÉ²´´TýqªÌ«mOOÏBeÔZµBeÿ¨¿ÛÉ³gÏ.jÌÍ[(+,BY¥iiiZf<¶¯XýiªiØ¡¡zÖçóÝºBÕ_ÉHt¡ÿIý+ÕeUbôÝ ¬Àc5(Bú¼E(«±³¶µµ-ª¬¹¹¹Ú=O<YïÏÿa®öTATæGFF¢,«*qjj*/²ËPÖéééÐãWPÖË/-Ü±cGØ³)©²½³«­­­j Íº¨÷N¨löÄ°eõz½²|Ë-¼6Ê<4&99yûöíÚ<2#CÃ¸¸¸¢¢"ýq="èdLZYet¯þ¨&ú/ö °C[Íèè¨Üúì³ÏF¿n·;è«¦ÔÝÝ]^^~ìØ±°ÿ¼ZÊD%ìÕDõz¤>xª]E¿f11.]SuuuCaÊ(+Ê(+Ê(+ÊeÊe¡þ?ÃÆHWQ*IEND®B`


KM time BY smoke
  /STATUS=status(1)
  /PRINT TABLE MEAN
  /PLOT SURVIVAL
  /TEST LOGRANK
  /COMPARE OVERALL POOLED.


Kaplan-Meier


附注	
创建的输出	19-JUN-2020 20:34:10	
注释		
输入	数据	E:\医学\肿瘤内科\孙佳春老师的肠癌统计数据\modified\data_modified 赋值.sav	
	活动的数据集	数据集1	
	过滤器	<none>	
	权重	<none>	
	拆分文件	<none>	
	工作数据文件中的 N 行	55	
缺失值处理	缺失的定义	用户自定义缺失值被视为缺失。	
	使用的个案	对于分析中的所有变量而言，统计量以带有有效数据的所有个案为基础。	
语法	KM time BY smoke
  /STATUS=status(1)
  /PRINT TABLE MEAN
  /PLOT SURVIVAL
  /TEST LOGRANK
  /COMPARE OVERALL POOLED.	
资源	处理器时间	00:00:00.28	
	已用时间	00:00:00.28	


[数据集1] E:\医学\肿瘤内科\孙佳春老师的肠癌统计数据\modified\data_modified 赋值.sav


个案处理摘要	
是否有吸烟史	总数	事件数	删失	
			N	百分比	
无吸烟史	25	21	4	16.0%	
有吸烟史	30	28	2	6.7%	
整体	55	49	6	10.9%	


生存表	
是否有吸烟史	时间	状态	此时生存的累积比例	累积事件数	剩余个案数	
			估计	标准误			
无吸烟史	1	1.000	死亡	.960	.039	1	24	
	2	1.000	删失	.	.	1	23	
	3	3.000	死亡	.	.	2	22	
	4	3.000	死亡	.877	.067	3	21	
	5	3.000	删失	.	.	3	20	
	6	4.000	死亡	.833	.076	4	19	
	7	4.000	删失	.	.	4	18	
	8	6.000	死亡	.	.	5	17	
	9	6.000	死亡	.740	.092	6	16	
	10	8.000	死亡	.694	.097	7	15	
	11	9.000	死亡	.	.	8	14	
	12	9.000	死亡	.601	.104	9	13	
	13	12.000	死亡	.555	.106	10	12	
	14	14.000	死亡	.509	.106	11	11	
	15	14.000	删失	.	.	11	10	
	16	15.000	死亡	.458	.107	12	9	
	17	16.000	死亡	.407	.107	13	8	
	18	21.000	死亡	.356	.105	14	7	
	19	23.000	死亡	.	.	15	6	
	20	23.000	死亡	.254	.096	16	5	
	21	24.000	死亡	.204	.090	17	4	
	22	26.000	死亡	.153	.080	18	3	
	23	31.000	死亡	.102	.068	19	2	
	24	36.000	死亡	.051	.049	20	1	
	25	57.000	死亡	.000	.000	21	0	
有吸烟史	1	1.000	死亡	.967	.033	1	29	
	2	2.000	死亡	.933	.046	2	28	
	3	3.000	死亡	.900	.055	3	27	
	4	4.000	死亡	.867	.062	4	26	
	5	5.000	死亡	.833	.068	5	25	
	6	6.000	死亡	.	.	6	24	
	7	6.000	死亡	.767	.077	7	23	
	8	6.000	删失	.	.	7	22	
	9	7.000	死亡	.	.	8	21	
	10	7.000	死亡	.697	.084	9	20	
	11	8.000	死亡	.	.	10	19	
	12	8.000	死亡	.627	.089	11	18	
	13	10.000	死亡	.592	.091	12	17	
	14	11.000	死亡	.558	.092	13	16	
	15	12.000	死亡	.523	.093	14	15	
	16	13.000	死亡	.488	.093	15	14	
	17	15.000	死亡	.	.	16	13	
	18	15.000	死亡	.	.	17	12	
	19	15.000	死亡	.383	.090	18	11	
	20	20.000	死亡	.	.	19	10	
	21	20.000	死亡	.	.	20	9	
	22	20.000	死亡	.279	.083	21	8	
	23	22.000	死亡	.244	.080	22	7	
	24	23.000	死亡	.209	.076	23	6	
	25	23.000	删失	.	.	23	5	
	26	24.000	死亡	.167	.071	24	4	
	27	29.000	死亡	.125	.065	25	3	
	28	39.000	死亡	.084	.055	26	2	
	29	58.000	死亡	.042	.040	27	1	
	30	87.000	死亡	.000	.000	28	0	


生存表的均值和中位数	
是否有吸烟史	均值a	中位数	
	估计	标准误	95% 置信区间	估计	标准误	95% 置信区间	
			下限	上限			下限	
无吸烟史	17.268	2.959	11.468	23.067	15.000	2.900	9.317	
有吸烟史	18.687	3.712	11.410	25.963	13.000	2.129	8.828	
整体	17.983	2.422	13.235	22.731	14.000	1.724	10.620	

生存表的均值和中位数	
是否有吸烟史	中位数a	
	95% 置信区间	
	上限	
无吸烟史	20.683	
有吸烟史	17.172	
整体	17.380	

a. 如果估计值已删失，那么它将限制为最长的生存时间。	


整体比较	
	卡方	df	Sig.	
Log Rank (Mantel-Cox)	.012	1	.912	

为 是否有吸烟史 的不同水平检验生存分布等同性。	

>º¾¾^h¤¦2ãv»ÕÆgðììl9;::Ê/ ¬ÀZúsPiÛf·mÛ¦æÔ¥+_å¢ÔÔÔð­»ÕÕÕáëËÀôêÕ«ÚÝ·oßõÐë¦5e+ýa®eÍcÇIÑe¹ÕjMJJÒnAm.@Y5DªêLh£lÖÉgÏUÍÓpAË©!Þû÷ïXê_=iOOÌ¤§§K»»c¹¨¥¥ENkjjätppPN·nÝªÝ¦ß @Y57`-++3,¥¬áMNN:NíõZÃÊ2úöövµýVN+**äTíë¤ª^ÕK¿²º®ßïw¹·[b,gÕNÂ1~(+pï¨q§þuSC±dà¨2f Þú¢6çê¯¸ÒvcY¾gÏë¡÷Ò455È¸¸8m¶¶6Y¢¶«$®ZqÕ÷ eU7%ãì	óK(+°¶¬-	Õ¾ð×YU,õ×êííÍÊÊ8Ø]éO­¬ª2U]TÛeÝÝÝGÑ¾5£ÖlllÜªÙw	 ¬ÀZ8fçñxdY_N%r2LQñ³Z­Í!G³Ú~¿ª*óóó×û:«¤TfâããeU0õ÷÷«ÍÅêm¬rïª`ùÝXÙÊ*¥j«É0×ä(cÖÚÚÚäädýòòr-ÕjüªU[G¢«^sMIIÑË¼,+òË(+°¾ËjXMícYÕÖ`)quuuNN>2<5Y5;wîTTíR)ºafXOe0¼µæËªÔÔÔ¸ýËªv°RÇrR/ÍªÍÂ2üUÇ~~_eÖGñMIIÑÎ®t°YeúúzYrôèÑð¯¢^ÕFñÐÁ²:Þ¬ßÙÙ)ßCvv¶þvõÇ ü«]XXàW	PV`M¸&¶*ÉW§ac¹zøÈXB¬ö@Y ¬PV@Y ¬1øõ¯íõQÖµbzzÚb±8`=[és@YïOYï÷~ïß`=£¬²eeÊÊJYPVPVÊ(+(+e@YoSaa¡~I0¸ºººë¡ÏhêèèèÔ)++xgÎÙLY`MÕçóµµµiÑjhh0Dqhh¨mÙÙÙUUU×Cs¤/ß®]»d¹vöìÙ³öìQwT^^.W4666ê¯;22¢ÿº²Pì"//¯»»[QÄATkÎÌÌPVÊ÷³¬R/)V¸sçÎ©¢fiiiyyYÍûýþ´´´·£"ûûûÓÓÓWúrê¢K.ÉTn9%%E-7|ø |E·Û-ß^:ªÊ?;;k(«|KÍÍÍªô«¨y¿NLLPVÊ÷º¬MMMÒ!ýHÑPVÍäääáÃ÷ïß¢|àîÎ;=ÏJª(JYU,ååTnPF±úÕZ[[e©¾6fu8ácVÉªvGýU[²RVX[cVettT2dTUkii).."V«¬¬LHHÐo1.((XVîÝ»WÎfeeUWWËÚ(SæeS§N©^666ÊMíÙ³Gn_¾=ÊJY`íµ··wxxX;;88¨?«¨WFôãE©¬8KJJ´Õ¤ÒHÉ[×DõeÛ±c<åËÂÝ»wk·mÛ&jj¯³ÊÂÌÌÌcV	pøÖ`É0e¥¬pOËÚ¹22ªñ¢ZíêÕ«ÚµRSSõ7"ã]5ãõz¥gÒiÉ~Ìj(äSæääH,[[[Ô¹¯¯O´<	´è÷`lÕï÷ËUó'OóGy²RV¸eUûÕÖÖJÆ¢ìp«¨í±Br¨fÎ;§½ÅerrR»jÌzäÈµ	ZÖ5e´ZYY)Ã_	¹~£±ZG?f³YdÆpRPùödÆn·ó~VÊ÷yk°"ÕkéééjÀ*I;~ü¸Úö«Q»Þâbx3)F³JÈµ]ÕþÀóóóÙÙÙLÃMÉTÆ¬2U_H®JKKÃÿ	QuÄå²À=-k___cÖË*K¢Y5UUUê]ïfddHËõÃPõ¶Ú!RhµeXûýþ·¦m¦¬îuY¥dÅÅÅj_¤K.mÛ¶-Êi¤¬ÃDÈØ4zYÕÖæýû÷ßÚÈÈÚÒëõzÏ;'ßÌìÜ¹³¾¾^cccjD+7(ß§þe`ÃjÇÇÇ%«2åÖvìØ!£áYbÊ·´nºuLJJ_.ÿìØl¶¸¸¸ääd·ÛMYlÌ²ÎÌÌÈ(P¿Éwtt´²²233Ón·KO>­Ë ·P]]¾qOOö¦JÃ7ó^øI.FªWgµ@Ë¨tjjª¿¿_[.ßUkd5õÇãq:aÃÁåöå¾4440f½cäg½uëÖ*yÄÈÿDjCDFFÌ|úé§Ã7zçw¬V+L¿55VUñññj?lùGj³²²6yànùÑü[ßÞuPøó@Ya×Y#>ªâââówuÌJYPV¼¬E|Þí×Y)+Ê5!!A½Ð-§2OYPVPÖÛ*«ÃáPû­ÉéJGë ¬(+(ëÍËªÎz<ÄÄDÅb³ÙVúèyÊ²²ÞIee¥¬@Y)+eÊÊJYlÀ²knn®^¸¥¥Eæ«ªªô+mxáÂYÓpSuuuêÃvôÂ²RVk½¬Çs?sáíøêGÚúOÏÌÌ4Ü:n¾ÌdggAµßïokkÓ¯&ëÈí477««úËBí¨¿.KåöðáÃíííê#í<5¥¬¿³ëë/ðç`Í5Íqy`x:ât±æçÿþ½ð«,--aaA+ëõÐûåôÜ¹súåËhUÖ©¯¯?ú´*«ZY_VuEýBYÓglô²Ê£6Ó÷LGÞþjÕõü©Xke]é¢×=cW5¯>FÎÉRÍ755=zT)Ô5==]N:ÔÐÐ `jUU$ÙëõÊµ®®N®~àÀââbYµÑ0e¥¬ÑÊê÷ÉTøþÅÓýøS°ËªªjFRWSS£T¢/ëÜÜÝnQìÌÌLiiéÕ«Wey (,,Të¨­ÁËËËê¬aÌjØ,_ÊJYÿmtìZ«+ &yÔ¾Pý®LOUï¿üãÚrY?ë¨¬IÝÀÀ¡¬óóój +eíèèPQTÕÔ¿L+ÃV­¬òLöìYCYe¹YfN<©LLLÈnô²¾÷Á'ç«®¨Iµg:eú³ÿ¯´WþìÜ²ÖÿÃ¿È¥)JYÝï|òåg»%ÇÁhxYUDe8[TT$hvww766êWËÌÌVeu:½½½gÎÑõzèCËe[PP@A)ë[ÕÌ+#íï_TóWÊàÞõWK?^XiþìWQçþ¯aIyy¹XõeTÛÕVßÙÙÙÊÊJõDúøRSkmm­tWªRÖúúz55UÈ-h»2²RVëikðªÈ(ÓårÉ¾¬---úu¤RÙtfdd¸Ýnu:77'yj»D>|X_Ö¡¡¡×;vìÂííí2Ì]\$¨õsþêUÊ`½Uí,ÏÚlöîÝê÷ûµÕ¦¦¦zzz´Ê%%%2ZØÚÚª]ETWWKåÆï^±døÔ©S2&¨Õ²XïcVPVÊ²²RV ¬ ¬ÀÆ)ëðð°á8û×9"?e¥¬(«òÁì?ülp¥éOÕjZ333KKKÕ|û¾ûä9#òSÖûSÖí]%®2üÁ©ê®7ø³pßË*ÏK'û¾qýà¼Zm9D½ßæØ±cá·Ãù)ë(ëG×F$®júÒëO<ïz?k¡¬+]ôêÄÏUJVËËËÕ°²¥¥¥££C-çüõ>UïBÊ`Ýµ±±QSéîînjj/+Gä§¬e½yYÝnw¿ÔQÁµ¤¤D©.åü²Ø(eõþâ/¼¼Ò½¬éï<¥æ¥mjOÝ(eåü² ¬/ÿÃO¿¿Ò3á'ÊuËÊÊô[µ²rD~ÊJY°5øVhe]^^v»ÝZY5²RVuN§v8p@*¨¿#òSVÊ²²RV ¬²RVk³¬Ã?ùPQVÊw¬¬iË²¡¬~púH×WG:lý××ùû°öËjø%77WâÞÖM PGXt»Ý2£í3uæÌÃÊ7öPV³õ¯¾û¢ÃU¨Î¿½ë ÿÖ~YÕAíè¾ÿY7²}ÑK.Iå6>_OOþørE¯×ñ¦¬f+«þSä(+õµ5xii)''Ç°ðÞÖMxY¥ÓêðÚ:2¤njjûöíSÁïðÊJY`ÕeïGSõ?^i²F¹T®«'''µ·Ò9u`!Ý:zô¨$óÞÖÍMË*cubÆ¬ÖÕPUÍH½jjj$ú§ÍüY7­­­²nP¾¢zWUn¼¢¢B½ô+öîÝ«fdÍãÇk÷²§¬Åßô¶¼6LY¬Ç­ÁºCYïñgÝÈ­ÉUå|¥1«O¿ß¯^ÓeÌjÚ²~ù×_¨~²Xwe¤ÉOJ^Ö»ýY7~KÛÍXUY+«Ëå¨ë_gU$«ccc%%%ë¨JY£q<ÓIY¬Ç²ËU_ÖÁÁÁë÷ã³nJKKUYUYÕèÖPV	ùéÓ§ÕØ777²RVXCeQ¦eF_Öý:÷æ³nd©¯Ü¾ëõNMMÉúòue5Rk«IõÕgE¾ýû÷>E²®ï²>ûÂ[O¿)<e:tòUû»3üý¸_eñèjß`ynÔÞc³wïÞ¼ÔÔTíkîÙgÝÈÕÛÚÚ$äêüü|uãáââb5toC½UÏçóI°ÇÇÇ)«IÊúÞxû>éhnÇéÞz¹õÇ2¥´üÿîWYAYy×PVÊJYoVÖ®¨é_üç÷²¬W&TÕemsúÇµ²>þÖ	d&ï½³<¸egÕ>C³#òSÖû^V½4Çe5óÊHáûy.°Ë:11¡033³´´TÍ···ïÛ·O39"?e¥¬(ë*Êº¢ÞosìØ±ð8"?e¥¬6JY~ó«O5µÒ$eréÿ^ú?§GÉjyy¹ªTKKKGGZÎù)+e°QÊúâÈËßþÊJ5Ê¥ßyIÿÜØØØ¨JÉdD^VÈOY)+¶ßÛíîïï:j/¸H¨Ô¥²RVue¶©=u£#òSVÊ²®ú]7eeeú­ÁZY9"?e¥¬(ëmuyyÙívkeÕpD~ÊJYlè²>ûÁùU==:N5#í<pà¡R²RV½¬ ¬(+e¥¬aÞûàóUWÔ$eu<Ó)Ó4~éåÌ]¶wü¿ç5&£c×Z]5IY_¨~W¦Õ¾êÖÞp]é±ìç]/ñ¼²²ÞúÖ`½'Z)+ÊÊJYPVPÖû]Ö¿ûöee½»(+ÊÊJYPVPVÊ²²RV ¬²RVë¹¬~¿ßf³ÅÅÅ%''kVº»»·nÝª.Ò÷² ¬ ¬+²ÛíÚ'ÿeddè/9MJJ¢¬(+(ëÍI>¯>ÃÏÏ~xbbâzèSpe^f¹,A-e@YAY?']8¯6oÙ²EprêóùdIGGÇ7nTTT´yófÊ²²~Îb±hóV«UÑÎ;ÕGõJbSSSÙ²²ÞT[e>Æá,e@YAY#s8uuu2#§v»]Sef``@Æ¯ee½9Çh±Xl6×ëýü[	=È®^½*AÑªÊ<e@YAYï:Ê²²RV ¬²RVõØÛ~ò¿¹þçõÎØÝqì+®¯ó¼²²RVuÍõÒ¿¼TùaLÛ»þû®ÿéN¦ç~x'õvËÚuTUæy@YAYo~ß`Ê²²RVu-õ/¾qÑu² ¬ ¬wÆùª+­®e@YAY)+ÊÊJYPVPVÊ²RVÊ²²Þ_»øvãòÓÅoÊ$eU3_ùk÷Åý<Y ¬²®ÚOF§¼«IÊªfj?¸òd²RVÊz[´­ÁW¹òd²RVÊJYPVPVÊ²²RV ¬²RV² ¬ ¬ee¥¬ee¥¬(+(+e@YAY)+PVÊJY)+ÊÊJYPVPVÊ²²RÖHeü­2íì:öýÿÊÊJY)ëmûèÚLåï4éåLõ)èOæ½öö»?åe¥¬õÖ½1êµ¿ùúôcÏºZ]8PVÊJYogÜéNÍ¯ºBYPVÊJY)+ÊÊJYPVPVÊ²RVÊ²²RV² ¬ ¬² ¬ ¬ee¥¬(+(+eÊJY)+e@YAY)+ÊÊJYPVPVÊJYPVPVÊ²²RV²RV²RVÊ²²RV²e¥¬² ¬ ¬÷º¬Û»¾2Ò.Ó_÷Åç]/©ùAPVÊJYWíÃ©òÞ;[øþEh)Ìì*Vó[APVÊJYo~k0e@Y)+e¥¬(+îmYå±wïÞcaÚÚÚä¢ÒÒRÊJYPVÊºº²¦§§«ùÞÞ^9;44¤]TSSCY)+ÊJYWWVÑRRR"óÚµ½½²RV²RVÊ²âþµ¬¬Lfõ[Essóüü<e¥¬(+e]iªÓé;út0Ò¿Úßï·ÙlrÝääd·Û­¿hqq1++Ëjµ~ñ_ìîî¦¬(+6DY[ZZÔáòòrÏÉÉù¢¢"Ém,W·Ûíõõõ2SUU¡¿¨¸¸¸¤¤DnG²úðÃËïÿûgn·yóæµüó?þgoãòy¯É$eU3úI.£wg²²~nvvvnnNÆÆÄÄÄÎÎNµ0Æ·ÜÄÇÇ«Ë`7))IbßóçÏ?v£ï®å?©kýdtJMRVm^òÏ÷p`a²!Êú;j¯%!CO§Ó)Ë=öÈàUÎÊBÃýêÒÅóêlEEÅ-[ä6ûûû×éÖ`½[9d?ÊJYo CÕ²²2yXx½^µCÏçóx<2l655E¿ºÅbÑæ­V«á¢ÚÚZ±©9Êª |ºÿÂ¥y² ¬5²3gÎÈÃB¯ZYO:%óÂ%$$Aµ5Xæ­4]§emý××ÕÞH¼ýUÊ²RV£ÉÉIy@:thff¦µµUíÍÔÖÖ¦.mhh³QnÁápÔÕÕÉÚívýE¹¹¹êº»ví2AY5|+ÊJY#×ÎÎÍÍÉã_ø~êêê¥¥¥(7âñx-ÍfSÛÕÀWí.£Õ@ @YPVl­Á÷e@Ya¶²Ê´¹¹Yiii² ¬¸­­ÁêÝ5~¿_zõÁLOO·¨âªcRVÊ²RÖÌÏÏËcB bffÆ°?°­¬¬¤¬e¥¬7±¼¼,£Rµ°zK«ÏçÓÞÞ^YN'e¥¬(+e½9õ>ÔyLÆ¯² ¬õ®¢ÞxJY)+ÊÛ*«×ëÍÊÊRßSõùqÉÉÉú=zzz(+e@Y)k¬V	§¨WXµ-ÃÚ>²RV²ÞÕju8·çääLLLßíozýu×ÁÇß:!Ì]æ ¬ØèeSMc(«Çã³*±YYY5Üg¿üì£k#jÚw9w×ÙRÃÇ¡ï?Ñ¾§²b#îÁ¤ÊªR*¹ùææfýø555²Fñ×?,­¸lø8ôVWà©¢nnÊXÖÙÙYõ:ëÀÀÌë/¼«µðý¯´zû>~ºøMnÊXÖ¡¡!yLÇqÖÖVÊJYPVÊº>PV²RV²®liiiaaAÍ;Îüø ¬(+ÌVVË%O¡¬eÅõÛ9nð-[Ô|½¾¬2¥¬e¥¬«°¼¼,¶S§NY­Ö¢¢¢cÇÉYu°~ÊJYPVÊ«¦¦&ý;NÍÆÖ`Ê²âú-_Æ¦² ¬¸eBYY~¡¬ê£Ñ)kt§û/lï:¨M]¡¬eÅÆ-ëÂÂB___zþuVSíÑ)kë*kwô«oðtPVlÄÙ|gËúrë|§²b#5ÊÖ`ÊJYPVÊJY)+Ê5»ºÉÊºïæ¸>ïÕ!zÊuyyyëÖ­;vìhkk;"3§OÞ²eKaa¡¶D,âîÉ¬cVý¾Áç«®´º<õæ³Þ7ÕPVÊPVlè­Á² ¬¸ceÓé4¼ÎJYo¡¬þ|FâJYÊUVíê%U»Ý.íEÖúúzYFYW[V=ÊPVl².h?Ø![)+ÊJYoõÊ6mÝº×YoÓco<ñÊH»gÜ§ò¿Óô²«§²bµ½½]½½½Bôo¹)(( ¬±;ùî/_ÉÏô=§¶w|ÞõO=eÅF)«úðóãÇë |=ôÑ­²¼¸¸²Þ¦'Z)+@Y±Ê*£RyLtvvjKeIii)[)+ÊJYWÇétVVVÊc¢££C[ØÝÝ­6kZÊJYPVÊÓË«			j÷%©éÎæææââbYb·Ûe^h(+e@Y)k4ccc©©©ÚÁ>O;Ø¡VïjM)+Êï,òòrí,e¥¬(+n·¬úý(+e@YqgÊ:66V__/K¤y² ¬õËÚÚÚªæ§¦¦äl^^Þ½ù¦)+Êõ>Úe=üÊ×ìßªjuÔ4ýÏx(+L[ÖÞÞÞÃ!ö577ËåÔ©Sõöp=ü;ß:_uE¦4ÇåNñ4PV¶¬Á`0ü@êm¬õö¾ñv5ÿdÞk ¬0sYõg§¦¦ü~¿<DÜn·]ZZ¢¬e¥¬«Ûw©®®®··×jµ^ÿíQøeÌ:00 3êÂUUU² ¬5Ö²^ºtIÍ466677ïÞ½hhHÎæççÏÍÍ)LY)+ÊJYoRÖÚÚZí¬Úqiß¾êÅ×ùùyÊJYPVÊzeÎÎÎ%j	e¥¬(+e]uYûûû½^¯Ì¸³îß¿_fT_)+e@Y)ëªË*UU[zè!Qq² ¬õV¶UVV:Nu1+e@Y)ëêª&Í&CÕ¦¦¦£GÊÙøøx9«ÃÔÐÐ K¤¸² ¬5&.KÖp<&5fõûý² ¬u ¬(+(+e¥¬(+e]:2pgg§<,æççÇÇÇÕ6a9KY)+ÊJYW½opgÌÌÎÎÊi[[:(¿á%XÊJYPVÊzsÃÃÃêÁrêp8úúúTnwíÚÅÖ`Ê²RÖÕñz½2B­­­=ö¬úL9u:rzäÈ¶ÒÒRí8² ¬5õ±çãããBÔgµÊ|»Ì?øàÛ¶mcÌzËæ¸¾ç|ÕUM_»øö½#<yë`k°zmµ¬¬ìäÉ%%%Eæ/]º¤ÞØËfa¿ßo³ÙâââÕç¥¨q)«ïz¤ÕXí$#ÝÆxò(+ÖzY§¦¦äÑTüæææ¤2H³jü*srr¢ßÝn¯¯¯ªªªðwìØ¡=æFFF®ÜH¾ÕjÝ8e½5UÊPV¬õ²JGµ!¦ÚIµVÈÎÌÌÈiyyyôÛWÜ`0d¸ôÂ2ÖsYYYÂ<ðÀ²f³JY%¨mmmòxè¡ZZZdF½¥U_N½^oô8/ÆÆÆRRRÔ»cÕ_þòÞèÇ?þ1cVÊPV¤¬r*£R«ÌtÉb±¨òóóå~ÓÑ®"¤úäê===ªÓ¼ÎJYÊ±PnÛ¶Íçó©ÃD¨å­­­ÚÌEÕÖ`¿áºe¥¬eùËª¨O8WG4ÖªÃGÈiwwwôë:ºº:S»Ýù;cÌJYÊRV	[yyù>8;;+g[ZZ*++Õ1Õî¾2o±XfffVºÇ(ëØl6íEYÃ²RV²Âüe.+%%eaaA-:ªcDhdÌ:66v÷¾éPÖgú¾¾½ëà-LGÞþ*e(+ÖåÖàûh#õÖxÆ¾ç(+@YAY)+e(+(+e¥¬(+(+e@YAY)+e(+(+e¥¬(+e¥¬eee(+(+e¥¬e¥¬²®¯²Ö6¤9.OùÌëü ÊÊJYW]Ö¼?]ü&?(²²RVÊPVPVÊJYPVÊJY)+ÊÊÊPVPVÊJYÊÊJY)+ÊÊJY)+@YAY)+e(+(+e¥¬(+(+e@YAY)+e(+(+e¥¬(+e¥¬ee¥¬ ¬ ¬²²RV²RV²²RVÊPVPVÊJYPVÊJY)+ÊÊÊPVPVÊJYÊJY)+e¥¬(+(+e¥¬ee¥¬ ¬ ¬² ¬ ¬æ+ëö®ï_4Lyïøl²²®äóöðIrûÑµÊPVPVÊzg<þÖ	ÊPVPVÊJYÊÊJY)+ÊJY)+e@YAY)+e(+(+e¥¬(+e¥¬eeÅeMs¸ÊôdÞkÿÔý?²²RÖ[/ëÔµÏ$®j¸Ö4~À ¬ ¬õÖËª'Y¥¬ee¥¬ ¬ ¬õ~ÛÞuðOßøséëco<qòÝ3 ¬ ¬õ¶|òÉU¦WFÚ¿|%²²Þúp¥¬ee¥¬ ¬ ¬² ¬²RV ¬ ¬²²RVÿ_çOÿùÏs(+@YAY)ëQhÚýÏvÊPVPVÊJYÊÊJY×ÏN©£þí_üûð;?O¦¯7ÿãßï]~8ee¥¬«öÒÕ<þÖ	¶w)ÅX&9ÞVÂ ¬0CYý~¿ÍfKNNv»Ýú¼^ï;ä¢mÛ¶ÉjõÎÒï|òËeâgPV¡¬v»½¾¾^fªªª222ô=òÈ#½½½2ãt:ôQÊz÷ÊúÔ«ZVæ¸lÌ@Y±ÎÊ¿¼¼,3Á`0))i¥Õ¶lÙ"§Ï<óÌ¿»ÑüÉÈ ?ËÛ,kåu2Vðöütñü ÊuVVébÄy=Ï-3o¾ùæ·nTVV¶yófþ,oÁµÙéQV²ÂleµX,Ú¼Õj_annÎn·ÏÏÏ³5øî¡¬eyÊÕÖ`711599É¾Á ¬ ¬1q8uuu2#§26Õ_ÔÝÝ½oß>ig«SVÊPVPÖx<ÄÄDÅb³Ù¼^ïçßJèA´I²RV²²Þu²²RV²RÖWÖö¿vóÃ(+(+îLY2:Å1ÊÊÊPVPVÊJYÊÊJYMVÖí]3Ïé§/¿ýì~ï«7¬p%ÿ)ÿßðÓ(+(+F§Ç<ã>ÃÔö¡çpqmørmze¤ý±7à§PVPVÄä¦[?º6òø['øA ¬ ¬²²®÷²¦9.·º+MÿàºÖu,âEÓ¿ø @YAYqO>óµo¯º²ÒTøbç®Gøré±T @YAY±:+m~2ï5ÊPVPVPV²²RVÊPVPVÊJYPVÊJYAYÊÊÊPVPVÊJYÊÊJY)+e(+(+îDY·w|e¤Ý0ýÇ²òÚËáËoêäÇÊÊJYMkâ³É¼÷Î¾Ñ0ýï>å+	_~T<üãÙÊÊJYÍï.m¬RVPVPVÊJY)+@Y)+ee(+(+(+@YAY)+e¥¬e¥¬ ¬ ¬ ¬ee¥¬ëUãò±g]O¿¹ªIzÜæPVPVPVÊ£æíûxµÄµ¦ñÊÊÊJYqgHV)+(+(+ee(+(+(+@YAY)+e¥¬ ¬ ¬w¸¬ï_üèÚ¦k³ÓüêAY)+eÅý)ëKW°½ëàão0Í¤>Ý_=(+e¥¬¸?e5SVPVÊJYAY)+(+(+(+eee¥¬²²²RVPVÊÊÊÊJYAYAY)+e¥¬ ¬ ¬²²RVÊÊJYAYAYAY)+(+(+e¥¬¯ºòÑ)ÓOãÓ²RVÜu¯üÓiËOæ½fîIîã×.¾MYAY)+eîVW@æ²RV²²²k»¬yïÝÞu0Ó÷Ó-L_¾ÿÿo(+(+eeý]Y?º6â÷1ÝÚ$ÃýÇÞx²²RVPÖß·Cþ/yü­²²RVÊJY)+e(+e¥¬ ¬e¥¬²²²²RV²RVÊÊPVÊJYAY)+(+(+(+e(+e¥¬²²RVPV²RVÊÊâ÷ûm6[\²Ûíñ"ÊÊJY)+(kdv»½¾¾^fªªª222b¼²²RVÊÊY||üòò²ÌÁ¤¤¤è÷»ßêF'NØ¼y3Ï2 ¬²²~....â|ÄÊËË÷ß(--M.âYëEÿà¸ÄÃíûùìTåu5Å¢Í[­Ö/bk03¡¬õNJHHj¯ÌÇxe@YAY#s8uuu2#§v»=Æ(+ÊÊÇãILL´X,6Íëõ~þ­d/¢¬(+(ë]DYPVPVÊ²RV ¬ ¬@YAY)+ÊÊJY²²ee¥¬(+(+eÊJY)+PVPV ¬ ¬e¥¬²eeÊÊCY­VëÇ°QVÊºÌÌÌÈÕz«,K\Õä~É½³¿¸õûÛ¼y³)ïÚ!·|õßÿýß'BÕ$ôÑ^xÁw­®®îþàÌúû£?ú£oûÛ¦¼k%%%<ò)ïZ0Y»)ï]~~þ=	(+e¥¬²RVPVÊJY)+e¥¬ ¬²RVÊJYAY)+e¥¬²RVPVÊJY)+e¥¬ ¬÷<;_¹rÅwmppðßü¦Yq?úÑLy×|>_mm­)ïÚo~ó/òÞuww766PV(+uòûý6-...99Ùívày½Þ;vÈ=Ú¶mÜ;SÞÇÎÎNí p¦¹wYYYV«õ_übww·É~qr¶nÝªîÇã1Ç½NJJòdb¾?=PÖØíöúúz©ªªÊÈÈ0Á=zäGzeÆét>úè£æ»KKKò¯VVÓÜ»âââååeÐÃ?l²_|üØØÌÈ©ªÑz¿wòÿü¯ ?Ìoø=2ßÓ(k¬ðò=´¿¢þßOsØ²eùîãÊÊÊ´g4ÓÜ;ÖõÁ)ÿ+LLLÈªÿÖû½;pà@ Ð5üûéuEqqqçMÀçóeggì>Ê'%%E­´g4ÓÜ;ùæ+**ä!	O¿É~q~¿_îüÖäT¦¹wú²ß#?½²Fc±X´y«Õjû577g·ÛçççMvÓÓÓzzôÏh¦¹wrGÔÛlå¿ýâvîÜ©FäØÔÔTÓÜ;YÃïY^@Yo"!!!^m®ysÜ©¬¬¬ÉÉIóÝÇM72Ó½Óój|c¦_èÍ÷N_ÖðdÊ§PÖs8uuu×CÇ*A	îQww÷¾û¦§§M|õÏh¦¹w¹¹¹ê»ví2Ù/NÆ©r¿df``@Æ¯¦¹wú²ß#Sþé²ÞÇãILL´X,6Íëõà%%%Fuæ»úg4ÓÜ»ÙÙÙôôtÏ¥¤¤ýâ®^½*A'§2o§/kø=2å(+Ê(+ÊeÊeÊekÀÒÒÒÂÂw:ún``@V«èj7uóý¦M?äK=öîþ½mÚÖÖÖvì·d^fffòx(+p»D¥¢¢âzèãðÄJköõõ¥¨IåZêëëU´Ôú[¶lÙ½·,ojjåô%åååá_ettT.¯ÕÓÓbè¡åQDüGAîÙ³'|áþýûÃªÊ¬b'	TóFYe8«_s9DÍOOOËËÊÊ¢ß²ú8ñùùyoii1]]­Ý ¶Úàà WËðW©v­è_TÓÞÞ.+gggÇ^Ö#GöööÊr©;²1tI9ÚBN:eµZ´âJeùÖ­[oÚlYe¬éÑn¼ªªJNm6Z?ÊYàÊ¼ÅbÙ¹sgs|>Ò5ðx<²æ¹sçVúÆDøÖà¼¼¼ðØPPV V±ÊÊJí¬ÄOëÜM2;¢SAAÁJe­­­ÕAÕU³ú²ê·*Ëü¶mÛb/«º£GFI~Ä¿ácV%â+Ê(+¹1RÄUUs?¾´´¤/¥z1µ¸¸Ø04AgCCÌLOOë_j½iY÷îÝ«­¹÷îØË*_QVQîµ6¥¬êÒääd0enUÃk²JêÂ¯%£R¹bgg§¶dppPß¾~Ì*©SKy<XÊªª¬2ép8b/kØ·6fEEE±ï5²bãuaaAÛãWÿ:«Ì«466Ò[YY)Ë;::´ÝÝÝjã°6¨XV58ÖöºcÖÜÜ¾hôôÆø:kµ@YÏé7êFßÜÞÞ 2#5Ý"cÊââbõæTW#ÚÊ:66¦µê.UþPïÛÉ.¬jÌË[(+`¥¬RÄÔÔT-3>OÛV¬Þ¢jqh¨^4¶«wÉHt¥ÿ$"¾K5JYÕ[lß-ÊÜVYÒç-JYUÕÕår­ª¬999Ú§O½¬×CoÌÕnÊ@e~rr2Æ²ª§¤¤ðð(+pÊº°°¾a¥(emhh0,Û¹sgÄ£)©²^ÙÕ¾ÖÖÖÎÎN5å¾¨×JT;6G<zbÄ²úý~Yþè£òØ(+pÒ­[·îØ±CÛGfdh¸eËÂÂBý~=Âp0&­¬ccc2:ÌÏÏWoªýKGÜ!(âÐV355%>ôÐC±ßG¯×kø*á»)õ÷÷effFü?@:uG@YD|SML×Md©^½j×ÐÙ¦M.]xQMMMCaÊ(+Ê(+Ê(+ÊeÊeáþ±w*zýIEND®B`


KM time BY tumor_size
  /STATUS=status(1)
  /PRINT TABLE MEAN
  /PLOT SURVIVAL
  /TEST LOGRANK
  /COMPARE OVERALL POOLED.


Kaplan-Meier


附注	
创建的输出	19-JUN-2020 20:34:48	
注释		
输入	数据	E:\医学\肿瘤内科\孙佳春老师的肠癌统计数据\modified\data_modified 赋值.sav	
	活动的数据集	数据集1	
	过滤器	<none>	
	权重	<none>	
	拆分文件	<none>	
	工作数据文件中的 N 行	55	
缺失值处理	缺失的定义	用户自定义缺失值被视为缺失。	
	使用的个案	对于分析中的所有变量而言，统计量以带有有效数据的所有个案为基础。	
语法	KM time BY tumor_size
  /STATUS=status(1)
  /PRINT TABLE MEAN
  /PLOT SURVIVAL
  /TEST LOGRANK
  /COMPARE OVERALL POOLED.	
资源	处理器时间	00:00:00.25	
	已用时间	00:00:00.87	


[数据集1] E:\医学\肿瘤内科\孙佳春老师的肠癌统计数据\modified\data_modified 赋值.sav


个案处理摘要	
肿瘤大小（厘米）	总数	事件数	删失	
			N	百分比	
≤6.5cm	30	26	4	13.3%	
>6.5cm	25	23	2	8.0%	
整体	55	49	6	10.9%	


生存表	
肿瘤大小（厘米）	时间	状态	此时生存的累积比例	累积事件数	剩余个案数	
			估计	标准误			
≤6.5cm	1	3.000	死亡	.967	.033	1	29	
	2	4.000	死亡	.	.	2	28	
	3	4.000	死亡	.900	.055	3	27	
	4	4.000	删失	.	.	3	26	
	5	5.000	死亡	.865	.063	4	25	
	6	6.000	死亡	.831	.069	5	24	
	7	6.000	删失	.	.	5	23	
	8	7.000	死亡	.795	.075	6	22	
	9	8.000	死亡	.759	.080	7	21	
	10	9.000	死亡	.	.	8	20	
	11	9.000	死亡	.686	.087	9	19	
	12	12.000	死亡	.650	.090	10	18	
	13	13.000	死亡	.614	.092	11	17	
	14	14.000	删失	.	.	11	16	
	15	15.000	死亡	.	.	12	15	
	16	15.000	死亡	.	.	13	14	
	17	15.000	死亡	.499	.096	14	13	
	18	20.000	死亡	.	.	15	12	
	19	20.000	死亡	.422	.095	16	11	
	20	21.000	死亡	.384	.094	17	10	
	21	22.000	死亡	.345	.092	18	9	
	22	23.000	删失	.	.	18	8	
	23	24.000	死亡	.	.	19	7	
	24	24.000	死亡	.259	.087	20	6	
	25	31.000	死亡	.216	.082	21	5	
	26	36.000	死亡	.173	.076	22	4	
	27	39.000	死亡	.130	.068	23	3	
	28	57.000	死亡	.086	.058	24	2	
	29	58.000	死亡	.043	.042	25	1	
	30	87.000	死亡	.000	.000	26	0	
>6.5cm	1	1.000	死亡	.	.	1	24	
	2	1.000	死亡	.920	.054	2	23	
	3	1.000	删失	.	.	2	22	
	4	2.000	死亡	.878	.066	3	21	
	5	3.000	死亡	.	.	4	20	
	6	3.000	死亡	.795	.082	5	19	
	7	3.000	删失	.	.	5	18	
	8	6.000	死亡	.	.	6	17	
	9	6.000	死亡	.	.	7	16	
	10	6.000	死亡	.662	.098	8	15	
	11	7.000	死亡	.618	.101	9	14	
	12	8.000	死亡	.	.	10	13	
	13	8.000	死亡	.530	.104	11	12	
	14	10.000	死亡	.486	.104	12	11	
	15	11.000	死亡	.441	.104	13	10	
	16	12.000	死亡	.397	.102	14	9	
	17	14.000	死亡	.353	.100	15	8	
	18	15.000	死亡	.309	.097	16	7	
	19	16.000	死亡	.265	.092	17	6	
	20	20.000	死亡	.221	.087	18	5	
	21	23.000	死亡	.	.	19	4	
	22	23.000	死亡	.	.	20	3	
	23	23.000	死亡	.088	.060	21	2	
	24	26.000	死亡	.044	.043	22	1	
	25	29.000	死亡	.000	.000	23	0	


生存表的均值和中位数	
肿瘤大小（厘米）	均值a	中位数	
	估计	标准误	95% 置信区间	估计	标准误	95% 置信区间	
			下限	上限			下限	
≤6.5cm	23.125	4.051	15.184	31.066	15.000	3.486	8.168	
>6.5cm	12.024	1.770	8.555	15.492	10.000	2.359	5.375	
整体	17.983	2.422	13.235	22.731	14.000	1.724	10.620	

生存表的均值和中位数	
肿瘤大小（厘米）	中位数a	
	95% 置信区间	
	上限	
≤6.5cm	21.832	
>6.5cm	14.625	
整体	17.380	

a. 如果估计值已删失，那么它将限制为最长的生存时间。	


整体比较	
	卡方	df	Sig.	
Log Rank (Mantel-Cox)	6.361	1	.012	

为 肿瘤大小（厘米） 的不同水平检验生存分布等同性。	

#%W¯^ëïß¿__Êüü|Y(óÚ­æææ¼^¯ÿ½oYÔÓÓãsÑìì¬,w»ÝrÚÚÚªSe¦¬¬ß@YõKÆúí«>eÐ_y~~^ÛN«®³:55e·Û#""FFFü/u¹èêêj5¢ÍËËÊL»Úø¬~öìY9;44Ä/ ¬Àzúï´HB¥mÝ³g¯««S¿ÊEIIIþ[w¯_¿îÞ½Wû¡©©©ß7íîîö¹¦q¥ß2ÌÑ°äÉRtYn6cccµPPV`¡ªz#SÚ[5rÍüü|Õ<Í+Wd¹úÄûÐ¡CKý»§20íìì´´4kGGÏàX.jhhÓ9õx<r§Ý§ß @Yu7`-**òYNYý÷$³ÛíÚûµ>WÑ§Ì477«í·rZRR"§j_'UPmðªÞú+¨ÛºÝî¶¶¶ööv±U;	ù8PVàÉQãNýû¦>Å£ÊõÑµ9WÃ`Ûeù.~¦®®nppPDDDhWhjj%j[±ÚwIâªW=)«º+gÊ_"@Yõ5`hI¨Rù¿Ïªb977§¿UWWWFFFÀÁn°7>µ²ª"ÊTuQmOö)«èèè8~ü¸öÔºfmm­äV½Ì¾KeÖ#ï¢cVC® ×S#Ó©øÍæúE'N³Ú~¿ª*333ÿü>«¤Tf¢¢¢|Êªö`êééQÕÇXKKKå"5ÞUoÁò»(+°1²ÁÊ*¥jWa®OCY+++ãããõKµT«ñ«VVí:]õkBB¶eÜ_@Y]V«©Â,«Ú,%¾~ýú¹sçôiá©ÏU366¨©Ú¥RtýPV`#uttÔç£5+.«RQQÑÖÖ¦¿BÀ²ª¬Ô±Ô[³j³°Õ±dß@Yu¤¡¡!àQ|´³Á¡/«ººZ8qÂÿ§¨7eµQ<tpNNïI®ßÚÚ*áìÙ³úûéïï×Bò¯îvvv_%@Yuáqä3´UIöx<¯,#N´´´ÈÍýGÆÊøø¸TÜÿ¸`µ?Êe²ÊeÃgfQÖõbbbÂd2Ù`#ö½ ¬O§¬_øÂþe¥¬(+(+PVPVÊ²²RV ¬ ¬@YAY)+ÃÕív744455]¸paaaA-ÌÎÎñÚØÞÞ~éÒ¥Ð¯sss§NçöüùóÁ¾"LÅÅÅMddd¼ÉÔ75mèo^¢¬°îÊª$3éééÚÂß-èr¹µhíÚµKÍÔÖÖZ­VÿoFòz½ïÇß±cÇûâ<88(ù×Î=z4[É½½½>?ôäÉ>GÒÈÌÌ¢¬«¬6Mª#³ÉÉÉrZWWW^^®]³¾¾¾µµUÍËòææfu[Y®îáU~ôöíÛÏòõV9+cYÛÊª©¸ªÚßßæsYÍ;w:NÊJY`%eÍÏÏÈ¸SN«ªª´Putt¨²J#+**TY/]º$aåj3²¥ÇúN+r©*´rýúõ©©)íGKáÚÚÚôçeY;22¢_¨°6ì¿ätff&++Ëçæ¥¥¥R^¸p!à÷<÷Ý»w)+eYýËªÎªK%HrÓ§O«Q£^¼xQJ)ÅíììTWÓÞ©Uï_>|8Ä¬T-77WÜøø¸4~ÉåøøxõÓýË*R­ÎÔU+ëüü¼Ö5(¿ººZæå¹¹þ>ÂsSVÊ².»¬ÝÝÝv»]µ§§§¬¬L®/YdZZO¹yRRÚKÈtWF´ªÄÚÖàUî`äV².ú~û5ÄUFÞÚ¼<0©~ccc%V³ÙLY)+,¯¬RýÖ`Iö>ë¥KTY%QRbÊÎ?/ýpªAj°÷#å&rçúwjõ®"öõøñãË-ë¡CÔÖàÖE)))r*?Ýk°ºzõªüPmØÞ+?Ëd2QVÊË+k]]RóÚÚÚÚÛÛµ!`oo¯´Gfúúú$RÊþþ~Éasss«Tjzz:à~I2L¼~ýºÿ~¶ÚhØ§¬òCöÙ/Ë111]]]>Õ µ¢¢BVFÒZ¼õ#Qõv¬ú¡rÍ`£êûöQVÊË+«ôRTÛìS7j«öá Ú¾pû­¸  @jåú-±ê-ÏûËCñ²,IÞµk>2¤ñ´£µØ«1«ü¦ßgJn%«)××~èÈÈHbb¢Ëåòù)ÃÃÃðÐ)+e@Y8sæÚ¸º¬Ãm_b9=zô¨ÚÈ7¥±±1jûÜÊHWî0Ä¡'û©ÒÒÒØØXíS7òº»»õï¤ê×E3::*±×öC,<`mc¹gY¢)+epË*M-,,æÉðÎjµ(kMM)²JärsslÔÕÕµcÇ¼¼¼ññqý§êëëeºÜgejï¡K¼õ¨ÕUí*Ty1WoÌ¹<*Õfmt®P²­£üuã¿ÜívË_%ñññê­Ê`SÕétª¥2£>Zªdffê¯&ã<íË#jc;éÚËª¶Uûpd8ôæßÐd )Y!µÿnM¡éÿØ¸NYePðY%ÿ¬ÕÕÕj¾zCûþýûýzóÍ7Íf3ÿ-uk0(ë²>|x`` à³***JýãõzÕ 6##cgyfÅÏæ¿iøîÞG´)ÅvÃúYÇûü·@Y±ÁÞgø¬ð_Ó1«Õáå²;mü·@Ya²ê?ì@«ø>+e@Yað²FGG«]´åTæ)+ÊÊúXeµÙlê  rl'1Ê²².]VuÖápÄÄÄL&Åì¸ee]Mee¥¬@Y)+eÊÊJYPVPVÊ²®Ü/îü÷ìùÊí?~¶äÌÍÍeddÍæ;vø(llL<ÚIY)+µú_û¥ ½ý§ÛßMÌ»íÂÂÂÀÀÌÊYÉêÎ;õ×©¯¯W_ÊºÂ²¾rí¶<_*¸%ÓÉo¶ñÿÀú/«LÁ.1kÀ²çåå%%%ÕÖÖÊÙøøø¾¾¾÷ Yõù¾qÅ:tH¸»wïV_¤#«c·ÛeÉõë×ËËËeFÿE=u³õ¡qg÷=5IbùÿÀHeQ©dòèÑ£ÇkmmÕGDDDFFÊUû~:e×®]©©©rþþÿÿN8!U»jiiSemhhJLL»¬êOY7Yõ(+µ««ëøñã2Tê¿?Õd2UVVÊ´Sð®FGG÷ìÙópñ¸î>ß½*«£hëµiß¥¬À(ëþøÙß~ó¦42à$¯T!Ê*ÿÞgáÈÈÈ¥K¤ 2ôæÃÅÃ¶ëÇ¯ÁîMWJÀ²¡¬²XcÖO>¡g°éÓ¹ù`7ýwÛÚÚZSS#3êWÇì³5xppPYSSSeFF½W®ÎÎNmk0e¥¬A¶/K~~þ±GËò©©©´´4õfªÚ[X«£Ëå|ÊE¸ª=<(KâããÕ²²RV´¬ ¬ee¥¬@Y)+e¥¬(+(+e@YAY)+¬jYÇç>þÕ'ÓgÞ`·å@ü² ¬¾~8ø/òjv¦ûÿÓñ`·å@ü² ¬Ê*S°KÜ~Q+krrree¥×û!,â§¬e]yYûûû/^¼(cÓììlÇóñSVÊ²>NYùùy)«z²RV±¬-x+S§ê?ÉKY²Ê¥3÷Ô|__ÏñSÖU(ë~ûö55Éîkã"O9e°Ë*ç÷ÁnÛøQÛì>ÕÞg­ªªÒ¿ÏÊø)ë*upâ·¯6«IÊúw¾/Ó×gþÁýÊ`#n~²®ÕÖ`©¬a)+ÍVVPVÊ²²®ã²¾úne@YAYW­¬Ê`ãuâOe"i² ¬«£ú_ûe"iuýuhøAcÛ¤¬¯^ÿ¥~~×õq´¶¶644´··Ëªå/^õ¹rzzºþBuµÇ3r¹ì¤¬¶[eúÏÿ£1ë»·x-`Ô²>/½±Ûí333YYYåååõõõ.«³³óìÙ³ÚuÓéÓ§O3f5òÖ`¿Jhy-`°².,,¨±>/½ñ/«ü8¼êËYWW'SSS[;wNnEY)+¬ZY½ÿñ§ÿýTAý'y¥)Ø¥2:7ïÃÃÃyyyIIIêLOæKo,«¤½¬¬LÍ3f5fYþÝO)+#UÒ%<zôè±cÇ$iÚò'ð¥7£££999òÓåGK«V«U:ªUîPÜª<xPÍÈ5O:5??OYPVe`­Á]]]Ç¡ªLxý'ð¥7r³z½ÞcV-n·;;;Ûç_ÊJY`ÝU¹téT2Ð|¸f_zã³ß: ¿¾¬2º¯þVE²:<<¸Qª²ØÔeÕÈx±¦¦æáúÒ«W¯ª²æææª²ÊùUÆÍ.?QeÝ¨eýëÿ7í;p'~KY²¬ùùùÇ%ÃÇOäKo¼^oii©å:Îññq)«IçææN:¥]MÒ.R;ÛÞÞ~èÐ¡`WQÖõ[V÷ýw²ÞÊ×¾´UâJYl²n£ºÝî¦¦¦ÊÊÊéééììlðæææUw«³êIÚO<922BY7RYõ$®À*+(+eÊJY)+e°^ËÚ0òºL$²n¼²¦Øn¨	¿rívg×ë¤¬?üÖóëöf8?e]vYõ_óÜMrÊëñSÖ§VV=¶RVÆ(+â§¬À&-ëøÜÇ÷?8·¯D¦`Ê¤ÝIrrree¥~[+â§¬Àf,ëçÿï×»Î¹ýbÀiïÍ#2»T¦?R÷ÓßßñâEfgg<²RVl~ü­ÁÒ*)«z²RVuåeíëëó³r ~ÊJYPÖ599¹ªªJÿ>+â§¬e]Í9?e]eýÇïß®ýi¯ÖIYÛÆ2­ÛíMr ~ÊúXeµ½Üúêõ_òº`²e¥¬² ¬ ¬«[Ö£÷ÓÛÿõ±Æ¬ú)+ÊÊJYPVPVÊ²²µ¬=¡áee]²êQV² ¬ ¬ee¥¬@Y)ëàv»-ú¶öövýEê«ä"ÃAYPVPÖ¥Y­Öêêj)++KOO×_5<<,3rûTÊúõÛg¾ûN	e@Y±aÊ*ùTßJïõzò¹sçNõ-r*ó2»ëQ²µk÷tÿ#ã;ï|² ¬Ø0e.W###å	'§.K´´´|ïQyyy[·n¥¬(+(ëçL&6o6õ%&&ªoÄ&%%=É­ÁïþÇÓ®oÉ´÷æöÿÂ*ÓÇj¡~zî#.­¬/ÜéÅo·_û¡²>ÑÑÑ^¯Wmù0³k]Ö7?zKz)Óé<)­¨é'¿ùZ¨ßþ¶Ó[ï|èì¾'SEí;W^,PVÊúØl¶ªª*S«Õª¿HÆ©Gfzeüº>÷x«±@Y)ëSàp8bbbL&Åbq:?Å'ÙÝ»w%¨2ZS§¬(+(ë[ë²6¶¸ï¿CYPVPÖ'² ¬ ¬ee¥¬(+(+eÊJY)+e@YAY)+ÊÊJY²RVÊJYPVPÖ§QÖÛÚwÔôÞû÷yá@Y)+e]yYhYÄ6¶ðÂ²RVÊºò²ê].»CYPVÊJY)+ÊÊJYPVPVÊ²RVÊ²²RV² ¬ ¬² ¬ ¬ee¥¬(+(+eÊJY)+e@YAY)+ÊÊJYPVPVÊJYPVPVÊ²²RV²RV²RVÊ²²RV²e¥¬² ¬ ¬ee¥¬(+(+e¥¬(+(ë5¿Äb»zúYÇû¼²²UÖ%1¨@Y)+e¥¬(+(+e@YAY)+ÊÊJY)+ÊÊJYPVPVÊ²²­¬ß~û¥ïU½ÿ`P¦ÁßRVõ±¼únÙÞGãd² ¬ ¬«² ¬ ¬ee¥¬(+(+eæk·Sl7^*¸%ÓóY¯Nð*PVPVÊºò²~04îì¾§&I¬åU ¬ ¬uåeÕ1+e(+(+e¥¬(+(+e@YAY)+ÊÊJY)+ÊJY)+e@YAY)+ÊÊJY)+ÊJY)ëÚµ÷½.Ýáå ¬ ¬uuÊêì¾÷RÁ-^nÊÍXVyB<xð¤¦¦&¹èêÕ«ª¬Útüö7(+ÊJYWXÖ´´45ßÕÕ%gûúú´***6OY5×i×·VpÃÛ¿sñÐü­ÚaúeúÕ»#¼ô¨¬¢iQaa¡Ì>Z³677SÖðU¸ÿü¥7)«ÌÔÿ¬²²RÖÓoæÊMWÖ¢¢"YXXÐoõõõ333² ¬uy¤©v»=""âÂ^¯w||nkhn·Ûb±ÈmãããÛÛÛõÍÍÍeddÍæ;vtttlª²þúßïþó¯(+@Y±ËÚÐÐ 6Ëü¹sçd>//OrÎÍ­VkuuµÌ¥§§ë/*(((,,û¬îÜ¹Süä'?¹ø¨¬¬¬­[·¯¬z ¬Ø±£¤1&&¦µµU-ó#7QQQªÁ2ØÕ_$£XïåËÿêQ			2Þ¥¬(+SVµ×¡§ÝnÁådð*ge¡ÍfsébÀyu¶¤¤$22Rî³§§gSm¦¬eÅæ-«Uäiát:ÕM.ËápÈ°UrXWWúæ&I7Í>UVVÊL¿M)+ÊÍ²ÓÅåi!W­¬sssçÏyüEGG½^µ5Xæ.6¥¬(+YÖ±±1yB=ztrr²±±QíÍÔÔÔ¤.­©©³µµµ!îÁf³UUUÉZ­VýEê¶'99² ¬0xYûúú¢¢¢´³ÓÓÓòäøÒ¾¤¿Îõë×çççCÜÃá1LEmOV_µTZZV(+ÊÍ²5ø)¢¬(+VVÖ××ËÓ"%%²RVµ5¸µµUºÆívË»wïnÛ¶---ÍºHWû²RV²effFê>ûËÙÒÒRÊJYPVÊºøøxªÕGZ].vuuéËj·Û)+e@Y)ëÒÔçP+**ä9144ä3~¥¬e¥¬+ºÙ"õÁSÊJYPV<VYNgFF:ø¾ªï×ïÁÔÙÙIY)+ÊJYÃ°J8åtppP½ÃªmÖ®àr¹(+e@Y)ëÒÌf³Íf¸øÜ¹s£££###ký )+ÊuxxX5OYmiiQÍÈÈ ¬e¥¬Ë ÊªR*¹ùúúzýø5))²RV²kjjJ½ÏÚÛÛ+3ýýýúK=Ï>c(+Ê£µ¯¯O#à)+e@Y)ëÆ@YPVPVÊJYPVÊÜüüüìì¬·ÛíOøùAYPV­¬mmmò())ùØE² ¬xø8ÇTóÕÕÕú²Êp²RV².ÃÂÂ<!¢££?Þl6çåå<yRÎªõoÎ²î½y$÷ík>ÓËÝÿDYPVÊJ]]þÍív»ÅbakðôÚ`³ÿ$¹¥¬(+e]bS°M)k(+ÊJYÈjQQ~OYÕW£SVÊ²RÖpË:;;ÛÝÝ¶Hÿ>«ÌË©öè² ¬5óól¦¬(+V§¬!¶SVÊ²RVÊJYPV¬ã²ÎÎÎ®é~LeAÊº°°·oß¾¦¦¦dæÂ¹¹¹Úu°µ;e@Ya1ëøPe¥¬eÅ¦ÞLY×¢¬ßù]/Ü¸.wrvßã ¬ØÀeííí'Ýn÷y²>fYZû%®2r]ÖtòmÏg½Î@Y±Êª#B½¥jµZåÉ£½ÉZ]]-KRRR(ëãuedÀ*#]^°ÊTÖEZáüvÈÖ`Ê²RÖÞxË¸¸8Þg¥¬(+V¡¬ÍÍÍò´èêê:ºHÿÊª/«ör¿øðMÊ²RÖÀÛå9qêÔ)ý.~u«,/(( ¬=WÔW ¿ýçÞx² ¬5Ês¢µµU[âñxdÉÕ«WÙcÄuÚõ-Ê²RV_v»½´´T---ÚÂµqXÔRVÊ²RÖ°Þ^V»/IMÕ××È«Õ*ójDÛÛÛKY)+ÊJYCNJJÒvv¹Áe´º¦5¥¬ ¬0ø¾Áò(..ÖÎRVÊ²âqËªß_²RV«SÖáááêêêììlY"Í£¬e¥¬+,kcc£³YYYOæASVÊPV°¬Oe¥¬e¡ÊÚÕÕulõQõõõòD9þ<e¥¬(+e]¯×ë ¹¹9õ1ÖÊJYPVÊºGv||ÜívËS¤½½]ÎÎÏÏSVÊ²RÖåí»TUUÕÕÕe6þù(ü2fíííuá²²2ÊJYPVÊnYËËËÕLmmmýþýûûúúälvvöôô´Ï!)+e@Y)ëe­¬¬ÔÎªRSSÕ¯3335XY÷wüªiô1ÊPVPVß²ö÷÷=Väææª%5Ïïúã¶Ê¤¾²õó²öôô8Nikk1ë¡CdFõ²#÷ík ¬D²þ¥¬RPÕWµ5xûöí2£â*)+e@Y)ëJ¶ôõõÚívu1+e@Y)ëòª&ÏÅ"CÕººº'NÈÙ¨¨(9«ÃTSS#K¤¸² ¬5,mmmú³>ÇcRcV·ÛMY)+ÊJY7Ê¢¬'¿ÙöÁÐ¸Æ|Â@YAY)ëÊõ¾÷QíÆóY¯Ë$3µïðâPVl²ª#·¶¶ÊÓbfffddDm³õ)UO²JYÊ³opë"Ó¦¦&uP~·`)+e@Y)ëÒúûûÕåÔf³uww«Ü&''³5² ¬uyN§P+++óóóÕwÚÈ©ÝnÓãÇ7-ºzõªv	ÊJYPVÊúÚó-ÔwµÉ|ss³ÌoÛ¶mÏ=Y)+ÊJYÃ¥Þ[-**:sæÉdJHHùòòrõÁÖp6»ÝnÅ¯¾/Ýz²RV²ÂøegÃÀÀßôô´4R©rV_eá¹sçBßÕj­®®²²²ôôtÿ÷íÛ§=çï<J~Ùl¦¬ ¬ØðejCLµûj­6ÓâââÐ÷¥¸^¯766ÖçÒ+W®È XÎeddlñóÌ3ÏPVÊPVaÌ*e 655Ésbûöí2£>ÒªÄ/§N§3ôDDDÃÃÃ			êÓ±jÉïÿûûúõ¯½ÑÇ¬z®ì½yÄú#²«¬sssr*£R«ÌL&uQvv¶<QÒÒÒ¼í&B©¿HnÞÙÙ©:mà÷Yr¸N»¾EYÊM·PîÙ³Çår©ÃD¨åÚÌíõzÕÖ`ä=²RV²ÂøeUÔ7«#JkÕá#rrrä´££#ômm6[UUÌÈ©ÕjüÈ³RV²bUÂV\¼mÛ¶©©)9ÛÐÐPZZª!¬v÷yÉ499ìGLLb±hoÊú<É(ëãõrÙíox(+ÖcY½^o[[[BBÂìì¬ZÒ××'uTÇÐÈuxxxí4eGmSþo+@Y±Þ·?Eu¹$® ¬ ¬²²RV²RVÊ²²RVÊPVPVÊJYPVÊJY)+ÊÊJY)+@YAY)+e(+(+eÝXe½ÿ»ÉÛ¿ü-¯heeÝeó£·öÞ<òÜ/È$3-÷~±êeuvß©à¯heeÝeï?TÓ_^|m°²²®Ü·¯QV²²RÖuZÖÛSiõ¯xu(+(+e]9ý¯F(+(+e¥¬ee¥¬e¥¬Õ¨eøø_ÿûÊPVPVÊºú(+@YAY)+e(+(+e¥¬(+e¥¬ee¥¬ ¬ ¬²²RV²RV²²nâ²f½¿÷æÓ®oÉôÜ/|øñG ¬ ¬uåÞß1âR$öý ¬ ¬uuÈ²²RV²RVÊ²²RVÊPVPVÊJYÊJY)+e¥¬(+(+e¥¬ee¥¬ ¬ ¬² ¬ ¬W7²²RVÊPVPVÊJYPVÊJY)+ÊÊJY)+@YAY)kHoÉûZéUáL¿ù7ÊPVPVÊÊ?¿_fVÕ×¤SV²²RÖÕáqI/ëóY¯7¶¬Ö4ññ'üÊÊºyËÚygèk·/ÝY)Åvã¡q~eeÝ¼e]]2ü¥¬ee¥¬ ¬ ¬²²RV²RVÊJYÊÊJY)+@YAY)+e(+(+e¥¬ ¬ ¬²²RV²²RVÊ²RVÊJY)+@YAYáSÖ½7¼6ØbúÅoRV²²RÖ°¼ÿ`0ë­üÜ·¯ßþFø_àJYÊÊ§¶¹8Åv#ûrçr¿îk·Þ5Èï²RV_7o®àËÒe¤[Qû¿PVPVÊJYWd²²²RVÊJYÊuVV·Ûm±X"""âããÛÛÛõ9ÎûöÉEöì«QVÊPVPÖ¥Y­Öêêj)++KOO×_´k×®®®.±Ûí»wï¦¬ ¬ ¬KZXX¯×ìjrúòË/ÿ§Gõ«_A-ÿ-)+@YAY?']8¯çr¹Î=+3·nÝú_***Úºu+ÿ-)+@YAY?g2´y³Ùìééi«Õ:33ÃÖ`ÊPVPÖ¥EGG½^µ5Xæ.ÍÈÈcß`ÊPVPÖ°Øl¶ªª*Sê/êèèHMMv¸9e¥¬eeÃá1LÅét~þPd±±±[t(+e(+(ë£¬ ¬ ¬²RV²RVÊJY)+@YAYAYÊÊJY7oYSl7^*¸lz>ëõ¦ö~q ¬ ¬²åÞÝ÷BLWµ ¬ ¬²²¹ ¬²RVÊPVPVPV²²RVÊJYÊJY)+e¥¬eee(+(+e¥¬²RVÊPVPVÊJYÊÊJY)+@YAY)+e¥¬e¥¬²RV²²²²RVÊÊÊJY)ëSY×b»ñ|ÖëzU¸öC7O?PVÊJY)ëÓ7ññ'oôIÝÿTz§(+e¥¬«£±màree¥¬²²²²²RVPVPVÊJY)+(+(+e¥¬ ¬ ¬ ¬ ¬²RVÊÊÊJY)+(+(+e¥¬²²²RVÊJYÊJY)+eeeee¥¬ ¬ ¬uËº÷æk°éoïdùåEþ¡V½¬Ù;µ¯¾øøþM@Y)+e5S×ÓkÍõóÿÊ?ÔêºyPûYÐòoÊJY)ëfñþÁçÞxµs¹ìee¥¬²²²²²RVPVPVÊJY)+(+(+e¥¬ ¬ ¬ ¬ ¬²RVÊÊÊJY)+(+(+e¥¬²²²RVÊJYAYAY)+eeeee¥¬ ¬ ¬²RVPVPVÊºñËº÷æÜ·¯¾ýö÷^·¨Ë/qJÝ÷V6õ¾÷ee¥¬É©×CL¥ïUIzùZ±ªúwO~³í¥[L+ä.Åv²²RV6«ã¡ñç³^§¬ ¬²²RVÊÊÊJYAYAYAY)+(+e¥¬ee¥¬ ¬²²RVÊPVPVÊJYÊJY)+eee¥¬ ¬ ¬ ¬²RV²RVÊºêÜn·Åboooó"ÊJYAY)+(k`V«µººZfÊÊÊÒÓÓÃ¼²RVPVÊÊXTTÔÂÂÌx½ÞØØØÐýøÇ?þûG½ðÂ[·nåU²²RVPÖÏEDDxQqqñ¡G¥¤¤ÈE¼Ê<£¼÷#þðT?ø¤¢öÊÊÉdÒæÍfs±5PVÊº¢££½^¯Úä+óa^DYPVPÖÀl6[UUÌÈ©Õjó"Ê²²æp8bbbL&Åbq:?Å'YÀ(+ÊÊº(+ÊÊJY²RVÊ(+(+e@YAY)+PVPV ¬ ¬ee¥¬@Y)+eÊÊ² ¬²RV ¬ ¬@YAYÃ(«Ùl¾e¥¬ëÈää¤YÍ+e2"""ÌF$ë%kg6(~q÷·uëVC®Ú3V|ó/~ñD²ÄîÝ»_õUC®ZUUÕ¿üe£þâ¾ò¯üèG?2äªîÚµË«æõzedÖÜÜlÈµËÎÎ>pà!e¥¬²RVÊÊJY)+e¥¬²RVÊJY)+(+e¥¬²RVÊÊJY)+e¥¬õ)Wç;wîrÕ<Ï~ð£þâJKKß÷]C®Ëåª¬¬4äªýéOºvíÚàà !×®£££¶¶²@Y ¬ëÛí¶X,ñññíííX#§Ó¹oß>Y£=öÈÚr[[[µÀfíæææ222Ìfó;:::ö5Sëâp8±v±±±!^L÷_5,V«µººZfÊÊÊÒÓÓ°F»víêêê»Ý¾÷nã­ãüü¼üé Õ0kWPPPXX¸°° Ú¹s§Á~qQQQÃÃÃ2#§ªFíäïù[A_ÿ52ÞË(k¸ÿáåµìáâþú??!22ÒxëxåÊ¢¢"íÍ0k'Ã¾¾>£>9åoÑÑQSõwÃF_»ÃèËê¿FÆ~ye*"""à¼¸³gÏleÄ ¯VÚ+aÖN|IIü1$áééé1Ø/ÎívËªÉoMNåiµÓÕüòÊÉdÒæÍf³aÖkzzÚjµÎÌÌlÓÒÒ:;;õ¯hY;Yõ1þþ~ùëÁ`¿¸ÄÄD5"Ä&%%fíôeõ_#£¾¼².!::Úëõ>óÆX©ÑÑÑ±±1ã­ãGiíô^oôó½cíôeõ_#C¾¼².Íf³UUU=Vò°F©©©^Gý+aÖ.33SaÀãñ$''ì'ãTY/éííñ«aÖN_Vÿ52ä=PÖ¥9Éd±XN§Ö(66ÖgTg¼uÔ¿¢fí¦¦¦ÒÒÒd<000`°_ÜÝ»w%¨²vr*óY;Yý×Èÿõ@Y ¬PV@Y ¬PV(+ ¬PV(+ ¬PV(+ ¬XæççgggÕ¼Ýn×Hh½½½re¹~¡vWK?é·l	ø%_²<??mÿ¿mÙÒÔÔtòÏd^>ç@YÇÕÖÖ&Q)))y¸øux"Ø5»»»Ó©Iå999Zª««U´Ôõ###÷ïß/Ëëêêd¹ÏWzÉââbÿ244$ÉÏêììL_äÓ?088ðYxàÀÿò_¨þePV`8I 4êË*ÃYý5©ù	¹aQQQèV_'>33#ó>^¿~]»CíjG+Ëeø«¿T»Uèªinn+=6ü²?~ÜgaWW,ºó<(+I#::ºiÑùóçÍfs^^6@¶qXÇÅÅ-ÙlYe¬i[¤ÝyYYZ,u¯×+ge+ó&)11±~<¼Ãéúp8rÍK.`ÂkpVVÿûúúØPPV ±ÒÒRí¬ÄOëÜÃAÏ]¤SNNN°²VVVêÇ jÌª¿[Yõ[e~Ï=áUÝÏ'B$?à_ÿ1«ðeÜ)â²Êª¹§N×R½ZPPà34AgMMÌLLLèßj]²¬Ô®¹ÿþðË*?Q®600b­µAp8eUÆÇÇó(+°DVÞ³ô)«¤ÎÿV2*¶¶¶jK<,¹zõªÿýëÇ¬:µ·Ãá§¬ªÊ*6-ü²Ø½²1«ÈËË¯i·¬³³³Ú¿ú÷Ye^µ¤¶¶Ö'½¥¥¥²¼¥¥E[ØÑÑ¡6kÚeUcmï¡µ³fffÊÕäNoï³Ykø~£nè­ÁÍÍÍÑÑÑ*3RÓÄE2¦,((PNy5¢íííVÖááa­UkTVùk@n'D&¯²¬1k8l ¬¯e"&%%iq¹¶bõÑUÓCCõ¬Çãñ¹t¶«OÉH4Ø_?¥¢¬ê#¶>e«¬>Òç-DYUÕµ­­mYe=wîvÍ._ÖÌÕîÊÊüØØXeU%NHHàéPV`Ê:;;ë¿ÏþJ!ÊZSSã³pzz:111àÑTYÞÙÕFccckk«È³.êý`%ª=1`YÝn·,ß½7Ï²KÆÄÅÅíÛ·OÛGfdh«ß¯GøI+ëðð°³³³ÕjÂÿÑw8´ÕË¥Û·oN§ÏOñßM©§§§¨¨èôéÓÿçÏçÙPV ,?TÖóuËAêb¯ÚuôßlËòòòUTT9@Y ¬²@Y ¬²@Y ¬²@Y ¬PV@Y ¬PVàïÿÁÇþæ|cIEND®B`


KM time BY T_stage
  /STATUS=status(1)
  /PRINT TABLE MEAN
  /PLOT SURVIVAL
  /TEST LOGRANK
  /COMPARE OVERALL POOLED.


Kaplan-Meier


附注	
创建的输出	19-JUN-2020 20:44:02	
注释		
输入	数据	E:\医学\肿瘤内科\孙佳春老师的肠癌统计数据\modified\data_modified 赋值.sav	
	活动的数据集	数据集1	
	过滤器	<none>	
	权重	<none>	
	拆分文件	<none>	
	工作数据文件中的 N 行	55	
缺失值处理	缺失的定义	用户自定义缺失值被视为缺失。	
	使用的个案	对于分析中的所有变量而言，统计量以带有有效数据的所有个案为基础。	
语法	KM time BY T_stage
  /STATUS=status(1)
  /PRINT TABLE MEAN
  /PLOT SURVIVAL
  /TEST LOGRANK
  /COMPARE OVERALL POOLED.	
资源	处理器时间	00:00:00.37	
	已用时间	00:00:00.50	


[数据集1] E:\医学\肿瘤内科\孙佳春老师的肠癌统计数据\modified\data_modified 赋值.sav


个案处理摘要	
T分期	总数	事件数	删失	
			N	百分比	
T1-T3期	38	35	3	7.9%	
T4期	17	14	3	17.6%	
整体	55	49	6	10.9%	


生存表	
T分期	时间	状态	此时生存的累积比例	累积事件数	剩余个案数	
			估计	标准误			
T1-T3期	1	3.000	死亡	.	.	1	37	
	2	3.000	死亡	.947	.036	2	36	
	3	4.000	死亡	.921	.044	3	35	
	4	6.000	死亡	.895	.050	4	34	
	5	6.000	删失	.	.	4	33	
	6	7.000	死亡	.	.	5	32	
	7	7.000	死亡	.841	.060	6	31	
	8	8.000	死亡	.	.	7	30	
	9	8.000	死亡	.	.	8	29	
	10	8.000	死亡	.759	.070	9	28	
	11	9.000	死亡	.	.	10	27	
	12	9.000	死亡	.705	.075	11	26	
	13	10.000	死亡	.678	.077	12	25	
	14	12.000	死亡	.	.	13	24	
	15	12.000	死亡	.624	.080	14	23	
	16	13.000	死亡	.596	.081	15	22	
	17	14.000	死亡	.569	.081	16	21	
	18	14.000	删失	.	.	16	20	
	19	15.000	死亡	.	.	17	19	
	20	15.000	死亡	.	.	18	18	
	21	15.000	死亡	.484	.083	19	17	
	22	20.000	死亡	.	.	20	16	
	23	20.000	死亡	.	.	21	15	
	24	20.000	死亡	.399	.082	22	14	
	25	21.000	死亡	.370	.081	23	13	
	26	22.000	死亡	.342	.079	24	12	
	27	23.000	死亡	.	.	25	11	
	28	23.000	死亡	.	.	26	10	
	29	23.000	死亡	.256	.073	27	9	
	30	23.000	删失	.	.	27	8	
	31	24.000	死亡	.	.	28	7	
	32	24.000	死亡	.192	.067	29	6	
	33	29.000	死亡	.160	.063	30	5	
	34	31.000	死亡	.128	.058	31	4	
	35	36.000	死亡	.096	.052	32	3	
	36	39.000	死亡	.064	.043	33	2	
	37	58.000	死亡	.032	.031	34	1	
	38	87.000	死亡	.000	.000	35	0	
T4期	1	1.000	死亡	.	.	1	16	
	2	1.000	死亡	.882	.078	2	15	
	3	1.000	删失	.	.	2	14	
	4	2.000	死亡	.819	.095	3	13	
	5	3.000	死亡	.756	.106	4	12	
	6	3.000	删失	.	.	4	11	
	7	4.000	死亡	.688	.117	5	10	
	8	4.000	删失	.	.	5	9	
	9	5.000	死亡	.611	.126	6	8	
	10	6.000	死亡	.	.	7	7	
	11	6.000	死亡	.	.	8	6	
	12	6.000	死亡	.382	.131	9	5	
	13	11.000	死亡	.306	.125	10	4	
	14	15.000	死亡	.229	.115	11	3	
	15	16.000	死亡	.153	.099	12	2	
	16	26.000	死亡	.076	.073	13	1	
	17	57.000	死亡	.000	.000	14	0	


生存表的均值和中位数	
T分期	均值a	中位数	
	估计	标准误	95% 置信区间	估计	标准误	95% 置信区间	
			下限	上限			下限	上限	
T1-T3期	20.276	2.916	14.561	25.991	15.000	2.907	9.303	20.697	
T4期	12.014	4.116	3.947	20.081	6.000	.572	4.879	7.121	
整体	17.983	2.422	13.235	22.731	14.000	1.724	10.620	17.380	

a. 如果估计值已删失，那么它将限制为最长的生存时间。	


整体比较	
	卡方	df	Sig.	
Log Rank (Mantel-Cox)	4.614	1	.032	

为 T分期 的不同水平检验生存分布等同性。	

»q»ÝçÏ¹Ü´i¿2²«×ää¤äÊjµjI«¨¨XìÎúFJO>-÷ß±c¾eee²Pæµ¯æ÷û7ô÷÷Ý4;;+Ë½^¯ãTq8üÖÊ¬^2^Ôï_+ëÄÄþÎóóóÚ~ZuçÔÔÔ|Ó©©)§Ói6oß¾y«ÇãB744¨mii©ÔTfº»»ÕÎgð¹:::Ê/ ¬ÀjúsPiûf333Õ|SSºu±ñ«Ü´mÛ¶È½»gÏ¼¿LoÞ¼©Ó]»vÝ½nzíÚµ°ÊWú-ÃË=8 Eå%--Mµ»eVªª2õ¡±7X388(÷,++SÍÓ:uJËeX¼wïÞ-±Ô¿z*ÓÞÞ^ÉÍÍ¸öôôå¦¹¬­­KÏ'éééÚcJqùXuÖÊÊÊ°%ñ5òH¢ññq§Ó©½^vgÊL»Ú+ÕÕÕr©uRÕ¯ê¥_¹úZ¯×ÛÕÕÕÝÝ-1«ê á8Nøâ¨q§þuÓ°bÉÀQe,zëÚ«ÿÂÅöËò;wÞ½¦©©iddDÍfímmm²Dí+VÇ.IâªAÊªJÆÙccc2#!çPV`uX%Zª]!¯³ªXÎÍÍé¿ª¯¯/???ê`w±>µ²ª"ÊTuQíO+«èééÙ·oö¨uÏÆÆFÉ­zcÊ¬FÁ¨cÖH.Kî ÷K#sCTü,KsÈþýûåªvÜ¯ê¢ÊäÌÌÌý?½Î*)äää°²ª#úûûÕîbõ6Ö¹IwÕK°üîÊ¬ìbeRJµ»É07,É1Æ¬uuuú%UUUZªÕøU+«v®zÍ5++K[.ó²D¾_@Yµ]Ö°»©â,«Ú,%>öì#GôiáiØU3>>­©Ú­Rô°ãPV`-ull,ì­5+.«R[[ÛÕÕ¥¿CÔ²ª¬Ô¹ÔK³j·°Õ¹dß@YU¤¥¥%êY|³²²´«,B_V¹OCC,Ù¿äwQ/Êj'£zêàãÇËÔùäþò3èghhHÉ¿zØÙÙY~eVGiRØÐV%ÙçóE½³8Ã0ÓÑÑ!_92VT<ò¼`u<Êe²ÊeÃþð'¬D²®&ÉkÙb»ÊúdÊú¥/éÀZFY)+eÊÊ² ¬ ¬(+(+PVPVÊ²>¢¾¾¾9wîc(++ûÈLqqñøøøôôôùóç;CJJJûÈPV0fY7D£¿Ã=zeÆår©Äêuuuãß¨¨¨îØ±£²²rhhHû,ÂûöÑNÊëtÌõ[ø|¾ê§awXXX(,,ìééyõQÁ2®ÕÊõÃAY`ýÕf³MNNêG¨çÎ»U³ª²Ê0WíÎÍÍ¥(ëÕÕÕj?°Þ'>ôeeÌJY².QVeGGGçÃÔgÔÕÕÉ¨t||<êuzzúÈ#²eÝ ßrªQårÛ¶mjykkkä§îY¯]»¶wïÞ¶öSV ¬áfff´¡gcc£ÄUøUõÇçååÕ××>1+eÊý[ÔÖÖ¶··«yÃÑÑÑ¡¿u~~^Ò[SS399©ÞS]]-¥¬(ko1;;k·Ûµ«oi=vìÈ÷ööæææªÈ²eß&[Qïîîjnß¾]«¦"CXmÿp ¨¨¨ðx<÷Coå]7«º¬òMKKõz­V«ÙlÎÈÈß:e@YEÆ¦mmmÚÕ¹¹¹¼¼¼°]Á²jWe³¬]­¬¬Ô?VQY].WzzúboanhhP»þåW.3wîÜzØûï¿o±Xø³@YAYØ³gÏððpÔgUrr²Ú/Õ 6???òO=õÔÍßjùÁó^Ò¦ûÈéW=óg²b½ÎõYe6ÃæëUÊ¹ð¤ãJk×0ö(+PVÉ¤ÍK>÷ë¬eÁËÕÞ`§¬(+(ë#Õn·×××Ël6Ê²²®°¬êªËåJMM5LV«ÕívSVõ±£¬(+(+eÊJY)+PVPVÊ²&ÊáÃå²¼¼Üï÷ªNw8SSS²RVk¯¬¿ÛýÚ©÷¢NrSã/?ÖÕÓ6QOÏõþ>/;;[³ÝårÝDt÷îÝêãZ«ªªjkkÕgÉOÊJY¬É²æØ/MDÎÔþæä¯.ö§gz	ªú|¾¾¾äädµpaaaß¾ª¬Á`°­­íÄruïÞ½ä²RVkµ¬Ýô¶Ë/q]¬1NÏõþÌÌLíªª²2f¥¬À:-kv.vëüüüÓO?-N§¶P?fmiiéêê¢¬² ¬Ë³JGõgÕY***ØLY)+U]Öú_Üø¢·bµûýOÿÓëâ/kØñM1º«>glffF"ºgÏ5f----//aÌJY)+U]ÖßÏÍ61»Ø4qï÷163³ÿ/QcÖÍ7û|>q»Ý»víÒ`ª««¸ÎÍÍåççë² ¬ÀÚÛ¼¼ú#ÕëõfddÈh5''g||À¸±±Qfd¨Z]]ÝÒÒ%·ZbAY)+#5±d¨zóæMm¾··÷Á^èîîââbòIYWÖïy/Ç~áÕòË2x½¿ë°¬ ¬,ëð'ãîk·Ô$åïee¤²êQV² ¬ÑqÊJY¬ë²^ºñ¾Å¦OgïhGùFÿ~è´ÚUÎ°OY)+Êz_¶Q¯/ê$7½~ýäCô³nÝºU¿3ìSVÊ²¾´ØM¿çÕ1ÊzêÔ©ÊÊÊ°a²RVu%eõûýYYY2Hû.a²RVu%eÍÍÍUçv1fåû²0`YÝ¿ûÍOF~¾Ø»¬¹ïg±²FÓÄö)ë**kÔ©­ÓxìeýùÅb[ÂOgïÈ×Æx5r!gØ§¬«¢¬Qqba_ÌÞàåmÐ*+gØ§¬eMÎ°OY)+ÊÊJY²RVÊJY¬Ú²rPQÖuQÖ¿úoïüøgýl<î²æØ/*Êº.Êj­óÍ³¿f»Àðeÿ³qå¦îînKµü7Þ»s^^^0¤¬²X¥eúY7²©LKKóþ>/;;Ûl6gdd¸ûËÿl§Ó933SXXxîÜ¹ÉÉÉææfÇÓÛÛ«¿³<²ÛíC1f¥¬ÿª­¸µë_'y®ï=üK«LvXcÖ°·®¦§§Çþú[%¨­­­2Ó××¬.ë³q"ËÚÓÓ#wÖõèÑ£MMM²p×®]!G¯¢¬ë·¬g~â=é¸":âô¥¬W?4ü¯¡Å&ÙÂÄ¸U¾v±RîÙ³gxx8þ²j:::233µ«ñ6ÎeN;5Ï²ÓLY¬Â²Æhçb·ÎÏÏ?ýôÓ²P©-ó³qÆÆÆ?.£^¹§ÄU²j³Ù¤£ZY%«ê¬Oj¨úÂ/¨¹çÁå[SVÊJY¬ê½Á+(«"µKII:fñÙ8Né±Ü'ÆUË§×ë-**[³RÖóãóýÞ¡¬ÖPYÃoÑ]³Ù|?ÏÆ	ÀS§N©kenGGGäW²ê÷û%Òª®ë²vû]/¾û¤¬YÝßIf^-]Á£QV³nÞ¼ÙçóÉÛíÞµk6ÿ³qN>­ÊZRR¢Ê:666>>vç¡¡¡cÇ©ÈG¥¬)ë½Ïï|wDMÔ÷U¦7?tûàMÊ`ÕëõfddÈh5''Gr¨`çgãÈòÑÑQÝJU¤ÒãjwxiW»»»wïÞ=88HY¹7ø­öÎPV«ª¬OðìËúl	³·­­MÆ¸ÓÓÓEEEÃÃÃ²¼½½½¼¼¦¦¦äÔYõ<Ïnß¾MY)+eðEuíõï?~² ¬ ¬	+«²XeÍ»ò*¡¢¬VÖç/½Øí'gØ§¬eë¨àÈ3òßöA»Êö)+e@Y7f<[áÖ­[õ9Ã>e¥¬_ÖÙ?þþÎïMRÖ·þùÿ£¬§Nª¬¬[Èö)+e`ð²þdäç/½÷íÅ&)k[ÿnä.VV¿ß%ñ++gØ§¬W²7877WÛ!Æ3ìSVÊ²Æ[ÖÈc8Ã>e¥¬(ë#Á¹3ìSVÊ²&²¬a²®²Ö6^WS[÷0	,ëë×O&jËÉö)ë)kó*«¯_~¹ð"	,+(ëz,«ÆíÄÊJY)+e@YAY)+ÊÊJY²RVÊJYPVPVÊ²²>ñ²>é¥CïÊôâ»¯¸n(+ÊÊºrc÷Æ¥¦jÚ÷Þ_Ih)+ÊÊ%¡¬(+(+e@YAY)+ÊÊúè¼^¯Õj5ÍÝÝÝúzzzÒÓÓÕM.² ¬ ¬K³Ùl2ãp8òòòô7%''ûý~Ë´´4Ê²².Mò¹°°p?ôaù|î¹çÆÆÆî>DWæe¦¤¤dóÃd¹j)+ÊÊút1ê¼ÚQ$O8¹ôx<²¤££ã¿?¬´´tãÆeeÀd2ióESvv¶ú<zIì¶mÛØ²².-%%%ª½Á2çp² ¬ ¬ÑÙíöúúzKÍ¦¿IÆ©>OfdüJYPVPÖ¥¹ÔÔTÉdµZÝn÷%ô$»yó¦UF«r)óeeì(+ÊÊJY²RVÊJYPVPVÊ²²RV²RV² ¬ ¬_tY¿ùÞá¦¬(+(kbüGWþ÷¯ÿÊ²²>á²æØ/H¹ð¢Ã²RÖu]ÖÒë?,ùàLÏ_zI&é«LyïóËwïIMÕtàõ®Ö®a6(+e]×emýç·ßiI²úÍ÷Ë°U&_ÁCt¬(+eeoðúc)+ÊÊJYPVPÖÕTVÉêßþ² ¬ ¬GYPVPVÊ²²RV²RV² ¬ ¬ee¥¬l8PVÊJY)+ÊÊJYPVPVÊ²!ÊJY)+ÊÊJYPVPVÊ²²RVÊ²²>²~ÿ9ö/^IfØ ¬²>RYwï2Pe@Y)+eÔ²êQV²RVÊ²²RV² ¬ ¬Æ/«ï³¡õj²ò|WMço¶PV51eÝÑó­ÿìù¯eeMÌÞà·FÚK>8CYPVPVÊ²²®²~«ï¯½¬êdLúI¾Ó7Â ¬ ¬ë®¬õÖ§wµó1iSÑÉ^N,PV"DY)ëD=§ì@Y)+e¥¬(+(+e@YAY)+ÊÊJY)+ÊÊJYPVPVÊ²²RVÊ²RVÊJYPVPVÊ²²RV²RV² ¬ ¬ee¥¬e¥¬² ¬ ¬ee¥¬e¥¬5±e-«vçØ/hÓ¨ÿ.[²²RÖÄx¹ðâ'£¶2eÅº.«<!^xáÚÚÚä¦Ó§OSVÊ²RÖå577WÍ÷õõÉÕÁÁAí¦ÚÚZÊJYPVÊº¼²¶?tè6fmoo7dY¿ôRÍz5ù>¢¬(+(ëÊ|6¨eU+¡¥¬(+YÖÊÊJYXXÐïÍÍÍ333Æ+«^Ég(+Ê,Mu:f³ùØ±cÁ`0è_mÍëõZ­VùÚîînýMsssùùùåÙgíéé¡¬(+ÖEY[ZZÔáªª*?räÌJnãùrÍÖÐÐ 3#//OSyyyEE<dõ¹ç%¿øÅ/ÞxXaaáÆ)+ÊãujjjzzZÆÆÔÔÔÎÎNµ0Î·Ü$''«Ë`7--MbÃ¾'OüwËÊÊñ®aÊúÍ_¹¯Ý¸ÊtlÍ@Y±ËªZ2ôt:2¸Ü¹s§^åª,´Ûí±¿u^]­®®NJJÇìïï_÷ÿ«ìäÍ@Y±Ë*CÕÊÊJyZ¸Ýnu@Çãq¹l655ÅþrÉ¤Í[,°êêêdfhhHÆ¦ë¡¬¹¾Z~Í@Y±N`zã7äi!W­¬sssÅÅÅ2vPR¤`0¨öË|ØMg)+Êcu||÷îlmmUG3µµµ©[Ï?/Wc<Ýn¯¯¯¹´Ùlú=ª¾Öçómß¾² ¬0xYµ«ÓÓÓòäxúé§õ÷9öìüü|q¹©©&ÉjµªýÉjà«ÊÍÍÑjVVÖðð0e@Y±^ö?AeÑÊ*CÒææfyZäääPÖD¹ôî'u±¹(+ÖÝÞàÎÎNõî¯×+KnÞ¼¹iÓ¦ÜÜ*®:÷!e]·~´÷ð/ÙÜëqÌ:33#Ï	uÉÉÉ°ãåjMMe¥¬(+e]ÂÂÂBFFJÕÀê-­G.ûúúôeu:5N»÷Ô	?ëÿó¿lýuÿ§jålzÊãYÕûPkkkå91::6~¥¬+PÛxýåÂêL2ýùVdæçÿ8È¦ ¬X/Õ©ÕO)k¢è>é¸ÒÚ5Ì¦ ¬Xeu»Ýùùùêäûr©>?.##CSoo/e¥¬(+ewÀ*áËõ«¶gX»Çã¡¬e¥¬K³X,v»=êà#GÝ¾ûqÿÐF-ë½k"GYÊuQV¿ß¯>&¬¬.K®vtt¨ÄæççSÖGDYÊõu*«J©äVæõã×mÛ¶QVÊ²RÖxMMM©×YdfhhH«Ïç¬ÏÊ²ÂheçÄðð°ËåzÖÖVÊJYPVÊº6PV²RV².n~~~vvVÍ;Î/øùAYPV­¬]]]ò¨®®ù´ÊJYPVÜó'%%©ùYe8KY)+ÊJYaaaA)))m!ÅÅÅ¥´´ôÀrU¬²RV²Æ«©©IÿÁæN§Ójµ²7² ¬¸¿â3òËØ²RV	(«<*++õKÂÊª>²RV²Æ[ÖÙÙÙk×®åè_gy¹Ô>²RV²Æk~~~aa½ÁeEbÊco0e¥¬(+e¥¬eÅ*.ëìììc=² ¬0HYÒÓÓ·nÝÚÖÖv Df;TRR¢-Q'x|'c¢¬(+3fýÞTCY)+@Y±®÷SVÊ²"ae'Óé²RV²ÆE;GzIÕf³ÉãøñãÚ¬²$''²RV²Æuv)ÉÙLYPVÊºÒ/Þ°!==×Y)+Êµ½½]Côo¹9~ü8e¥¬(+e]Þ>ayN<xPáû¡nååååõÿ9ö/^´û¶×ß|@Y±öÊ*£RyNtvvjK|>,9ú4"p÷Þ'£åN2ÌýNi,²bÕétÖÔÔÈs¢££C[ØÓÓ£vkZÊúÅs_»õjùe6XeÅZ*kJJ:|IjÒÜÜ^.Kl6Ì«íÀÀe¥¬(+eÅï÷oÛ¶M;0Øãñh';Ñêc­)e¥¬eÁçDUUv²RVZVýñJ² ¬HLYý~CCCQQ,æQVÊ²RÖµµµUÍ¹ZXXøÅüÐ²,ëDY)+@Ya¨²öõõ#Äö°ææfy¢SVÊ²RÖe'Soc­®®¦¬e¥¬ËàóùôW×ë§Hww·§¬e¥¬Ë;v©¾¾¾¯¯Ïb±ÜÿÓYøeÌ:00 3êÂ²RV²Æ[ÖsçÎ©ÆÆÆæææ;vÊÕ¢¢¢ééé°SSVÊ²RÖ%ÊZWW§]U.íÚµK½ø:33CY)+ÊJYWXÖ¡¡¡YRRR¢PVÊ²RÖeµ¿¿ßívËLWWYwïÞ-3ª¯² ¬uÙeª¾ª½ÁÏ<óÌ¨¸ÊBÊJYPVÊº½Á·oß¬©©q:ê&	*cVÊ²RÖåUMV«UªMMMû÷ï«ÉÉÉrUéüùó²DKY)+ÊJYãÒÕÕ¥¿v>&5fõz½² ¬um ¬ ¬ ¬² ¬uêÌÀò´¹û¶Ú',W=JY)+ÊJYlpgÌLMMÉe[[:)ØK°² ¬uiCCCêÁri·Û¯]»¦r»ûvöSV².Ûíj]]]YYúL¹t:r¹oß¾¶Ó§Okç ¬e¥¬±¨=¿ûöõY­£££2ßÞÞ.ó6mÊÌÌdÌJYPVÊ/õÚjeeåáÃM&SVVÌ;wN½±5ÝÂ^¯×jµÍæõyéaÔ¸²Æ/k gÃðð°ßôô´4R©rU_eá#Gb?ÍfkhhÃyìñÖ­[µçÜÈÈÈÉ÷²X,²k¾¬ÒQm©_R­Õ²rYUUûqÕ7¦¥¥ÝzêÔ)kÏ¹üüüzê)Êµ¬9öµ×Õtõú§l¼ÊÕ>f²JPÛÚÚä9ñÌ3Ï´´´ÈzK«:¿ÝîØb6£Î¿ß¥Þ«|þùçwöÑG1fêÖ§wµ¬ªÄ²ñ(+VYçææäRF¥W9wîÉdR7É%77wÉÑ¾DH#õ7É÷ööªNó:ë£PeãPV¬#Ô233Óãñ¨ÓD¨å­­­ÚÌÅÕÞ`èze¥¬eñËª¨O8Wg4ÖªÓG?~zzb­Ýn¯¯¯¹´ÙlÑ2Æ¬ ¬X'e°UUUmÚ´ijjJ®¶´´ÔÔÔ¨s«ÃeÞd2MNN.ö.+55UîcµZµeÃd²Æ/k0ìêêÊÊÊUK¥ê³úýþÇ÷C?ñ²^-ñÝWjnÔ/wjýç·)+ÊJYW'^Ö·o]^AVy¾+=¦¬(+e¥¬áºí¸RV²RVÊ²²RÖÆë'W>¨­@YAY)ë#ilÌ±_x¹ð¢L2CÊÊJYFâJYÊÊJY)+@YAY)+e@Y)+e¥¬(+(+e¥¬ee¥¬e¥¬² ¬ ¬²²RV²²RÖ5QÖû¢½'WÊªÝL ¬ ¬õQ½Ó7ÒÚ5,ÄõÀë]lÑÊÊJYÃíÖ«åÙ¢²RV²²RVÊ²RVÊJYPVPVÊJYÊÊJY)+@YAY)+e@YAY)+e(+(+e¥¬ee¥¬ee¥¬(+(ë-ëó^ª¹Qç4ðÙ e(+(+e]ÔÈÄ¿ÄUið[#í ¬ ¬51J>8CYÊÊJY)+ÊJY)+e@YAY)+e(+(+e¥¬(+e¥¬ee¥¬ ¬ ¬²²RV²²E(+(+e¥¬ee¥¬e¥¬² ¬ ¬5^m¿uô"[4²²RÖÄøyëGÿ-@YAY)+e(+(+e]5eýèî´vËtìÍwsìÞ<ûk5Üø-[7²²RÖe§oä¤ãLûvHYí¯uÊ$3?>ßÏÖ ¬ ¬uåôÇ×6^­@YAY)+e(+(+e]e½zýÓ¢½ ¬ ¬5ñ(+@YAY)+e(+(+e¥¬(+e¥¬ee¥¬ ¬ ¬uWË^|÷õjúl*@YÊÊJYWîí[µ¬>é¥ïPV²²RÖÄÁ+e(+(+e¥¬(+e¥¬ee¥¬ ¬ ¬²²RVÊJYPVPVÊJYÊãÕëõZ­V³ÙÑÝÝ­¿ÉívoÝºUnÊÌÌ»QVÊPVPÖ¥Ùl¶q8yyyú6oÞÜ××'3N§sË-²uiÉÉÉ2ÓÒÒ»[RRöÚkö°¯ýë2¨¥¬ ¬ ¬H£Îëy<¹|ùòß=¬²²rãÆ²õÉ¤Í[,È;LOOÛl¶öSV²².-%%%ª½Á2vëØØX~~þøø8Ç?Ö²Ö5äØ/¼Ó7~%lÊÕUV»Ý^__/3r)cSýM===»vívÆørÊ¿»÷Éh`¹ûÚ-+ÛD²buÕår¥¦¦L&«Õêv»ü(¡'YZZÚÊúøÊº2WÆ¬eÅª+ë#¢¬ ¬ ¬² ¬²RVÊPVPVÊJYÊÊJY)+@YAY)+e@YAY)+e(+(+e¥¬ee¥¬Æ-ëßnzz¦÷>g[	PVPVÊº<·>½õlÃ²PâÊ¶ ¬ ¬51$® ¬ ¬kÃó^*ùàLÍú/fjé ¬ee5²ø¸ùËê!ÏweLYÊÊÄpÝöH+@YAYAYÊÊJY)+@YAY)+e¥¬eee(+(+e¥¬ee¥¬e¥¬	(kýBmãõÖ®áåNýÓþÙAYAY)+e÷·?½ú75WN:7©óÏÊÊJY)kbHV)+(+(+e¥¬ ¬ ¬ ¬ee¥¬ ¬ ¬²RV²RVÊÊPVPVþ,)+@YAY)ëjç½sý/Þ²ññÝß² ¬ ¬²6^Ï±_qúÃïyï¾~/ ¬ ¬²ÆåÆÇwb¯ÿåÂjAYAY)+eew1@Y)+ee(+(+(+@YAY)+e¥¬e¥¬²RV²²beeþÒK57êWÉtø«dZ=?Ï§·o]æÙÊJY)ëzôÙT`UÉeñÝW¯ñìe¥¬ìN·FÚK>8Ão²RVPVÊÊÊÊJYAYAY)+S×4c¿ðráÅ5=ýÊªÿ÷ÇÔ¼¬Î¥÷82(+e¥¬xB&~wïÑÀZê.*Ô|ÑÉÞÖ®a~³ ¬²Ù|Òq²²RVÊPVPVPV²²²RVPVPVPVÊPVPVPV²²²RVPVPVPVÊPVPVÊJYÊÊÊ¬²þMÍÚÆë8±Ônz²²RVPÖ+ëßþôªÎü¤¦¿Ý&ÿz²²òY7!cVé+ee¥¬ ¬²RVÊJYÊJY)+(+@Y)+ee¥¬ ¬ ¬ ¬ ¬²²²RVPVÊÊÊÊ¯×kµZÍfsFFFwww7QVPVÊJYAY£³Ùl2ãp8òòòâ¼²²RVÊÊ]rròÂÂÌÁ´´´Ø7ýìg?ûÎÃ^yå7²e¥¬õ³Ùu>êMUUU»#7±ÁZá½s]âÊ¿Ã£Ü½WÛx²²Fa2´yÅçMì`$²&RJJJ0T»|e>Î(+ÊÊÝn¯¯¯¹´ÙlqÞDYPVPÖèWjjªÉd²Z­n·ûÁzE½² ¬ ¬e@YAY)+PVÊJY²²ee¥¬(+(+eÊÊ² ¬ ¬(+e¥¬@YAY²²RV²RVÊ(+(keµX,·`-£¬u1«e¥L&Ùl¶¬¬Å øÅ­Ý_ÜÆ¹jO¬øË¿üå/!Êj[¶lyóÍ7¹jõõõ_ùÊWúûêW¿úÓþÔ«VQQ±yófC®Z0Y»!×®¨¨hçÎ²RVÊJY)+(+e¥¬²RVPVÊJY)+e¥¬ ¬²RVÊJY)+(+e¥¬²RVPÖ'H¶ÎW®äªù|¾ýèGFýÅÕÔÔ|øáÇSWWgÈUûãÿxæÌC®]OOOcc#!e²@YW!¯×kµZÍfsFFFww·ÖÈívoÝºUÖ(33SÖÎëØÙÙ©Î0k777o±XöÙýâdÒÓÓÕº¸c¬ÝÄÄDZZZñþô@Yãb³ÙdÆápäåå`6oÞÜ××'3N§sË-Æ[Çùùyù¯VVÃ¬]yyyEEÅÂÂDè¹ç3Ø/.99Ùï÷Ë­õµÿÈÿô§ùãm^@YãýmÙýÐñúÿ~CRRñÖñÔ©SÚÍ0k'ÃÁÁA£>9åÿccc2#êÿkíöìÙ3<<¬/käóÊº(³ÙuÞ<OAAÁÖQF<YYY²µÒ¶hY;ùá«««å?Cþþ~ýâ¼^¯¬üÖäRY;Y#×ÈÀPÖXL&6o±X³^ÓÓÓ6mffÆ`ëÛÛÛ«ß¢fídEÔÛläûÅegg«¹$vÛ¶mY;Y#×È¨PÖ%¤¤¤Áû¡Ý52oËÏÏ7Þ:nxÖNÿÃ«ñ~q£7c¬¾¬kdÈÍ(ëÒìvýýÐ¹dg5êééÙµk×ÄÄ×Q¿E3ÌÚ=zTaÀçómß¾Ý`¿8§ÊzÉÌÀÀ_³vú²F®!ÿô@Yær¹RSSM&Õju»ÝX£´´´°QñÖQ¿E3ÌÚMMMåææÊx.++kxxØ`¿¸7oJPeíäRæ³vú²F®!ÿô@Y ¬PV@Y ¬PV(+ ¬PV(+ ¬PV(+ ¬XæççgggÕ¼ÓéÔHlrgùýBí¡~ÒoØõC¾dyYYÙãýÛ°!''§­­íÀÈ¼,<tèÏ²ª««K¢R]]?ôqxb±^»v-7D¥Hº(_xüøq-N*ZêþIII;vìåMMM²<ì#½dIUUUäwäõööæõ/¬å1Dý,Ü¹sgäÂÝ»wG.Tÿ2(+°$PÍKõeá¬þ!j~bbB¾°²²2ö#«ù°[Ï=«= v7Ï'Åå2üÕßªUìoªioo;Ä_Öûö-ìëëåRw'eâ"ér¤¤¤´[,ÒÒRm¸ØÎaY¾d³õe±¦=DpÃ!V«UÝ?ÊUàÊ¼ÉdÊÎÎnoÏ=±GºaÜóÄý`"ropaaaäÙQPV ^±íªÄOëÜÃAÏíÑÇéøñãµ®®N?UcVýÃêËªß«,óñU=Îþýûc$?êßÈ1«õeÞ)â²Êª¹×R½Z^^64AçùóçefbbBÿRëeá´îØ±#þ²Êw»ÇXkmOYÕ­<aÊ,Õ°×,ÃÊ*©ü*ÊvvvjK|>,9útäãëÇ¬:u´Ëå§¬ªÊ*v»=þ²ÆØ½²1«(--ÿ¨ië·¬³³³Ú¿ú×Ye^µ¤±±1,½555²¼££C[ØÓÓ£vkÚ¨eUcíè¡Ç7f=zô¨ÜM¾iìôÆù:kµ@Yô;ucïnooOIIQfÈ²¼¼9UæÕv```±²úý~­U©¬ò¿õ¾<uêÔ²Æ¬ñ±²ábU¸mÛ6-3GÛW¬Þ¢juh¨^õù|a·>¦½ÁêA2]ìQß¥£¬ê-¶a?-Ê<RYÃ"¤Ï[²ª«1kWW×²ÊzäÈíÇ¿¬÷CoÌÕ*ÊüøøxeU%ÎÊÊâéPV e<)ìx¥e=þ|ØÂéééììì¨gSReeWû1Z[[;;;Õ@6uQ¯(QØõìQËêõzeù-[xnX4&==ëÖ­Ú<2#CÃ¤¤¤ýq="ìdLZYý~¿ÔjâÿÖQ:´Õ¹õgÝnwØw<L©¿¿¿²²òÐ¡CQÿ y¶KÔ7ÕÄõ|Ý°AFúàÅ8ªvýmØpîÜ¹¨7ÕÖÖÆ9@Y ¬²@Y ¬²@Y ¬²@Y ¬PV@Y ¬PVéÿ%	()£½TÔIEND®B`


KM time BY N_regional_lymph_nodes_metastasis
  /STATUS=status(1)
  /PRINT TABLE MEAN
  /PLOT SURVIVAL
  /TEST LOGRANK
  /COMPARE OVERALL POOLED.


Kaplan-Meier


附注	
创建的输出	19-JUN-2020 20:44:42	
注释		
输入	数据	E:\医学\肿瘤内科\孙佳春老师的肠癌统计数据\modified\data_modified 赋值.sav	
	活动的数据集	数据集1	
	过滤器	<none>	
	权重	<none>	
	拆分文件	<none>	
	工作数据文件中的 N 行	55	
缺失值处理	缺失的定义	用户自定义缺失值被视为缺失。	
	使用的个案	对于分析中的所有变量而言，统计量以带有有效数据的所有个案为基础。	
语法	KM time BY N_regional_lymph_nodes_metastasis
  /STATUS=status(1)
  /PRINT TABLE MEAN
  /PLOT SURVIVAL
  /TEST LOGRANK
  /COMPARE OVERALL POOLED.	
资源	处理器时间	00:00:00.25	
	已用时间	00:00:00.26	


[数据集1] E:\医学\肿瘤内科\孙佳春老师的肠癌统计数据\modified\data_modified 赋值.sav


个案处理摘要	
N分期	总数	事件数	删失	
			N	百分比	
无区域淋巴结转移	20	19	1	5.0%	
有区域淋巴结转移	35	30	5	14.3%	
整体	55	49	6	10.9%	


生存表	
N分期	时间	状态	此时生存的累积比例	累积事件数	剩余个案数	
			估计	标准误			
无区域淋巴结转移	1	1.000	删失	.	.	0	19	
	2	2.000	死亡	.947	.051	1	18	
	3	3.000	死亡	.895	.070	2	17	
	4	6.000	死亡	.	.	3	16	
	5	6.000	死亡	.789	.094	4	15	
	6	7.000	死亡	.	.	5	14	
	7	7.000	死亡	.684	.107	6	13	
	8	8.000	死亡	.632	.111	7	12	
	9	9.000	死亡	.579	.113	8	11	
	10	10.000	死亡	.526	.115	9	10	
	11	12.000	死亡	.	.	10	9	
	12	12.000	死亡	.421	.113	11	8	
	13	14.000	死亡	.368	.111	12	7	
	14	15.000	死亡	.	.	13	6	
	15	15.000	死亡	.263	.101	14	5	
	16	20.000	死亡	.211	.094	15	4	
	17	24.000	死亡	.158	.084	16	3	
	18	29.000	死亡	.105	.070	17	2	
	19	31.000	死亡	.053	.051	18	1	
	20	36.000	死亡	.000	.000	19	0	
有区域淋巴结转移	1	1.000	死亡	.	.	1	34	
	2	1.000	死亡	.943	.039	2	33	
	3	3.000	死亡	.	.	3	32	
	4	3.000	死亡	.886	.054	4	31	
	5	3.000	删失	.	.	4	30	
	6	4.000	死亡	.	.	5	29	
	7	4.000	死亡	.827	.064	6	28	
	8	4.000	删失	.	.	6	27	
	9	5.000	死亡	.796	.069	7	26	
	10	6.000	死亡	.	.	8	25	
	11	6.000	死亡	.735	.076	9	24	
	12	6.000	删失	.	.	9	23	
	13	8.000	死亡	.	.	10	22	
	14	8.000	死亡	.671	.082	11	21	
	15	9.000	死亡	.639	.084	12	20	
	16	11.000	死亡	.607	.086	13	19	
	17	13.000	死亡	.575	.087	14	18	
	18	14.000	删失	.	.	14	17	
	19	15.000	死亡	.	.	15	16	
	20	15.000	死亡	.507	.089	16	15	
	21	16.000	死亡	.474	.089	17	14	
	22	20.000	死亡	.	.	18	13	
	23	20.000	死亡	.406	.088	19	12	
	24	21.000	死亡	.372	.087	20	11	
	25	22.000	死亡	.338	.086	21	10	
	26	23.000	死亡	.	.	22	9	
	27	23.000	死亡	.	.	23	8	
	28	23.000	死亡	.237	.077	24	7	
	29	23.000	删失	.	.	24	6	
	30	24.000	死亡	.197	.074	25	5	
	31	26.000	死亡	.158	.069	26	4	
	32	39.000	死亡	.118	.062	27	3	
	33	57.000	死亡	.079	.052	28	2	
	34	58.000	死亡	.039	.038	29	1	
	35	87.000	死亡	.000	.000	30	0	


生存表的均值和中位数	
N分期	均值a	中位数	
	估计	标准误	95% 置信区间	估计	标准误	95% 置信区间	
			下限	上限			下限	
无区域淋巴结转移	14.000	2.237	9.615	18.385	12.000	2.152	7.782	
有区域淋巴结转移	20.733	3.730	13.423	28.043	16.000	3.687	8.774	
整体	17.983	2.422	13.235	22.731	14.000	1.724	10.620	

生存表的均值和中位数	
N分期	中位数a	
	95% 置信区间	
	上限	
无区域淋巴结转移	16.218	
有区域淋巴结转移	23.226	
整体	17.380	

a. 如果估计值已删失，那么它将限制为最长的生存时间。	


整体比较	
	卡方	df	Sig.	
Log Rank (Mantel-Cox)	1.583	1	.208	

为 N分期 的不同水平检验生存分布等同性。	

½ð(+°vµ··ÛKyöìÙõw÷wÛÏöôôì_â	Q[[«[¨¨¨½Ù]»vÎÎÎ+W®hfffF§>ú(¿2²k×ää¤råv»­¤]¸pa¹+Û©(èú»wï¶2//O5oÕÜÜ½µáááMKnÜ¸tÑìì¬wuué´¹¹Ù§j¦´´ß@YµKãEûöÕ ²NLLØ¯<??om§5WNHH¸o:55UUU322z©ßïW¡+++Í677W5ÕLkk«Ùøl~üøqâPV`-ý9-Q¨¬m³Û¶m3ó555æÒåÆ¯º(%%%tëî¥KB¯¯é­[·¬oºwïÞÅ¥×M»»»®©1®ú­a®FÃºæáÃUt-MLL´nÁl.@Y5DCUóB¦=´¶[úúútÍ¼¼<Ó<ËùóçµAñÞ·obiõTÓöövÍ¤¥¥)®mmmAc]TWW§Ó²²2öööêtëÖ­Ömª¸üÊ¬¹kaaaÐhÊº'ÑØØXUUõzmÐ5úÔLcc£Ù~«ÓââbLA­Á«yéWW0_ÛÕÕÕÒÒÒÚÚªë¬ÙI8Êû	²3î´¿nT,MÆ·¾Í¹ö/»±?õÔSKï¥©©©ÔëZb¶W«¸æ>¨¬æ¦4ÎÕBÎ/ ¬ÀÚ°*ZÕÞ%¡¯³XÎÍÍÙ¿ª££#333ì`w¹>­²"jjºh¶'UÚÚÚ<hÝ3c®Y]]­ÜÙw	 ¬ÀZXvÌÊçóéº¾N9#ÓøÅÆÆÖ.9tèÎZûý.LÎÌÌ,þêuV¥T3qqqAe50Ý¸qÃl.6oc-))ÑEf¼k^åwPV`d+«J©ZWÓ07(ÉÆ¬åååIIIö%EEEVªÍøÕ*«uE×¼æl-×¼èùeXßeºÙ))Ê²­Á*ñ¥K²²²ìiÔð4hÌjÛ¹s§¤ZªèAû3 ¬Àz*ëèèhÐ[kî¹¬FYYYKKýaËjv°2Çr2/ÍÍÂþc?iß@Y5¤®®.ìQ|­³Ë,Â^V]§²²RK:ú]Ì²ÖÁ(Â:8;;[W0ÇÒõu?n¿þþ~û1(s³³³³ü*Ê¬	÷Ó¤ ¡­IroooØ+kÄ´q¦¦&yèÈØWÅC¡ý¡PV(+PV(kþõ_ÿµÖ?"DY×	Ëåõl¹Ï]e8eýßøÿëe¥¬(+(+PVPVÊ²²RV ¬ ¬@YAY)+ÊºvtttôôôhæòåËa?×!//O×ÑÌéÓ§ÇÆÆ¦§§¯Ò¼$''g¹O ¬XVÝÃ %A×Ù¿»f|>I¬]KKË³Ï>áÂut÷îÝýýýÖÇ<x1+eUÖ;wÚ?¸0è>÷öö9rÄ><º'O¶µµiÞ|ê°ÆµVYÃ~1e¥¬àä²=v¹²z<ÉÉIûõòåËA7¢²ZcVSVsÍÖà´´4ÊJY`cU§)))£££¡e-..6ÛíácÇÛËÊ²eýuG;::¼^oPYË¦¦¦æ;Ð0W£Ò±±±°cÖééé¬¬,ÊJY`ãUTV³¯Y¢'á¡¡!ó2ªÔ«Õ××~OÐµ»»ûgiXÂÖ`Ê´¬###&¡÷yffÆzVWW+®öÝ4Tµï^QQQPPÀ²À.«yµ¼¼<ô>566ùÒÒÒ¦¦&û¥óóóJoIIÉää¤yONqq±*KYk¯òç²Þµ¬sssÉÉÉA÷yvvÖz	V?úÖ3gÎìéÓ§ÛÛÛÓÒÒÌ6dÊêØ²¦z¯òç²Þµ¬RSSc_¢Áh^^5ßÚÚªjîÚµËª¦¡!¬µx||üÂ~¿qé°¼ëæÔ1111tyWWÛíIJJÒ¯² ¬kÆ¦ömzzzÐ¦`=W« ÖÙ¾¾>=Ã[gí·@YïÏçÛºukØGÇã©¬¬4ÛëõÒÌ|Ð§7Þx#66² ¬ ¬Ù¿ÿÀÀ@ØGU\ÙÌ 633sSGyäÍÏþw5tÊú6þÔPV¬ã×YÃ>ªbbbæ?1kËÀ¹Òëü© ¬pZY].5¯|~l¯³RVÎ,k||| 0[5OYPVPÖû*«×ë­¨¨ÐN=Ï.ëÎo² ¬pfYÍYÏàr¹Ünwggç.ëök(+ÊõQV²e¥¬(+(+e°ËÚ×××âÄÖË4S^^~þüyëFí·yæÌ¦¦¦ÑÑQsvxxØ­PÍÑgëëëÛÛÛWt?õ%###f~~~>///è/¾ø¢yt,b­¬¬4GRÔ*X´CY)+Êz÷gà#/´~ñüëa']TýïØcÍgddÞTAAÁÌÌIÐÙÐ/<~ü¸®sòäIkùtìºº:ó©æº«àÅ[_nµyqéÐíßÂ~vllL×ß»w¯n_ÉïèèP5=OOO,..6õöÙgu-Ô=¡¬e½û3pª÷jOÿDØébÙÎýõ¡_¢`VVVèòË/+===sssfIzzºõÁ'ú´oß¾+û÷ï7_2ÍWÍÎÎúý~u.33SåÓÌòóóõMuimm­5DVCk­ïÞÝÝ=88hXiÔ©ª?55¥+è6u#ÖhëóùVDÝ59×êYß²RVõ.e]î¢øWûhÏPxL®¤¦¦F£LESVÍh¦¯¯Ou´yÅT°½½ÝÜ©£©¦¾DÁÖ¨qxxXAÕtýÂÂBs#rY5ÊÔu;§OVÈÍ ;h«NkôYUU¥ë¦tçMo_s[Õ7n¨ÊZM¶SVXÍ²Zý3/=jgÌº¸ôoj¤ÇãQ&ÃnVíTÁòòòS§Ni|m.2Å]»véz5Æµ§Qe=ö¬¼k¶îZUâóçÏhµÆ¬ê´¾©©ªfNN®©5²¾µ=ÿ³µ	úÊ+æNRVÊ«ÚÖÓÓ¡¬fO%o¹×Y5ÜlllÔ-h¤¨ÎÙ»¥Ñ¤nS5ÔRßÎúVûÕ¢°õ°Æ---füj-Ö,4oUÓlÖxôðáÃºTßôÖ­[ÖÀwÇºÚêue^÷D÷ÖlO¦¬ÀF/kÅËoÿS?XnPÖÖ7~ö§Ï_Z¨9rdrr2BYûûûKKK[[[5^´.©©©QÉ:èvTÁ'Nhh½«Â©yÕÕÕf¦ÊÊJó§¾ãøøøre«Íã^ºtIÕxT÷ÁËê¾YWVÔUnkgggÍÍê>XñÖ UWÖ7UVûúú4º5¯þz½^ûÅ²Ø¸eýÅÜü³ËM?ÿE'ÃÙÿ´¤¨¨HcJY­&²ªUÖEÇ³6«Ê*WõõõÊfÎ?oÞcnDãWµSQTPUÝ)«Â900¡¬ÖyÌæE«¦ºWj¶Ù¿IÎËË3cV-×-ë[wuuY#Úîîn]ªoª4`ÕÝPõi0e¥¬(ëjRÌfUY­·£²ÚÇ®a¨µõ×CáÖVó~Sõd[RR¢ 7jDkF½Ö»nÔ`û»n:OHJJR¹õíÒÓÓ5ÊT ÍVk³)X7bÊjX[EÕÜ¿¿nÖþÎ"­­oª+X7HY)+ÊºjÏÀfß`=7³³³³öì1UKII1c¾5,èÍ¦Zú«a¥õæòòr³ÏFf?©:Mb5Ðþ~½dkÝ[û[kTMë½@æÎM¾öcMè3¶6w#E¬Ý²¦z¯>ÿª¦Ïü~g÷üÙX_cVPÖµUÖÑ	ÕÔLoQhù³@YAYï¥¬a§³-ßåÏee]Tóïðg`=µ¿¿?ô¥ÓEÈOY)+Ê*7§ÞþÑÏ~6ûµ¯Ë3¯êìÝ»×ìÙÄù)+e@Y·_;p¬ûKa']ôüÍsÖ0Îì&''>ö¦8"?e¥¬(ë¿u¹þaô¯ô-TVL´43Líe]äü² ¬QµººÚêÙâÒCíÉáü² ¬+(kkk«pjµ-×0´¬²RV.kç?ÿèß]n´7>oirrR3Ñ#òSVÊ`ãõï~úòrÏ?ý@_´Ðþ¶ûx9"?e¥¬Ø|ìÓ¨4´¬²RVu¬Di´§&ãæ²RVëe¥¬(+(+e°1ÊÚÿîÏ	e¥¬°jeMõ^%T²ÀC(k___èGÙ8qÂzÌü¬sGÝ[ÍX;Fé[ÌÎÎ]9==µÞKY)+'Õ ¬±öÞhuSUUeîÿåË'''kkký~¿þ°Òçów"ý¡QÖUóWOüÖ×ê["Lïó¼`nÖ@0+++tùü¬Ð²êuMYµv555¦èæ.é§ú?e½_®ùTMÎ¹ÒëËMz¬+®</¸ÿ²¾ùãñÊÿÙ¿Ü¤gêkí£=Cá1¹5C£LÅøY7w-«îuEÆ¬¶¬òe~ùæ_E¸âJY¬²Zý3/=jgÌº¸a>ëFÖëÆÍa¡tß´Êê¨UVÝò¬²gÏ3£k9rÄúyRÖû241üÎíAMÿùÕ?Éð¿ðÆûojº9ÖGY¬£­Áj[OOO².nÏºÑ7ÕêúÆ¬V>»ººThûkÆYWGÚëúµ£¶_; )¹õYM¡¬ÖKY·TeuðgÝlúse³Û³½¬úÖÊ°ýuVCYÕúêßûªRÖHrÞºøÒ`£§¬ÖQY4¦´ÕjÒü¬SVØUÿèFÊª5Î9cþÐMQVÊ²þzS­Ù¬j/«õvöY7ÂêÎëûê.i­^óoÁÜÜúm§ý=µZ¬íd@Y)+G5Ê£õÜhÎÎÎÎîÙ³ÇT-%%Åù6ÚgÝh­UbóÖØéééS§NµãùùùÒjÚÿÕ°j­ÿ!¬ÃYPÖÕñÎíÁÑQV½¬X(ë]PV²pHYÓ¯?G¨(+eU+«VúÙßßúÒé"Gäçüe¾¬æ°FFFFAAWuöîÝkölâü² ¬ÑÕ¼OÔ¼ßæðáÃa¯Ãù9"?e°!Ê:ûË_|ðñå&=E¸ôÿÌÿß §Geµ¨¨ÈBÃ8³ÉÔ^ÖEÈÏù)+gõß=ðúgôáÒ¿üý¹±ººÚ>SEìÉáü²`kðö`jmmÕNM²¶åu#òsD~Ê²F¦ ©¦¬#òSV5Zö·ÍØÀ»Èù9"?e@Yï§¬F¥¡eåü²ØÐeþæ¹==ZÒhOM²#òsD~Ê²b]¢¬ee¥¬(+(+eÊJY7hY?ì/y»ÂL*kÿ3]¹UGYPVPÖëù°/´¬»Û>ý96e@YAYWgkðK9o]¤¬(+(+e@YAY×LY?ÝñÊ²²®>Ê²²RV² ¬ ¬+ÕÕÕåv»cbbìG^^óÖ­[ÍEöc4SVuY§²²R3¥¥¥éééöâââ>z>11² ¬ ¬w§|ÙAù|üñÇGGG>8PóÉÉÉyâNZ®A-e@YAY?¢.7·lÙ¢NÍï555íN¹¹¹7oþøËzðõ?óø5úÛ¿¿ÖÃóÊµRVËeÍÇÆÆÚ/Ú¹s§ù@]%6%%eílVP~í¨9Òáök¾ÒòPV¬²ÆÇÇ³5XóQgnYíþ¨.² ¬XCeõz½Ñ©Çã±_¤qjoo¯fzzz4~]e¦æK»ò>sòûR½Wy@YñËêóùÛíîììüè®,=ÈnÝº¥ j´ªSÍ¯Í²fw÷}whe@YñðËzzYsÞºøÒ`£§¬(+(+e@YAY)+ÊÊJY²RVÊJYPVPVÊ²²RV²RV² ¬ ¬ee¥¬@Y)+e¥¬(+(+e@YAY)+PVÊJYÃÉ¾qá»ïÖSVuu<ëûÜoþe@YAYWÇ§|ee¥¬(+(ëZ*ë±|1Ãÿ¦í×hÚýC¦_ï1?ÝñÜ_çÉe¥¬5Z×ßÿoÄ¯éÛ¾ë¿UÔhÚõÍÂ·+W,PVÊJYW,ì»nW²@Y)+e¥¬(+(ëC-ëÞµçÃ>Ê²²®>Ê²²RV² ¬ ¬(+e¥¬ee¥¬(+(+e@YAY)+e@YAY)+ÊÊJYPVPVÊJYPVÊJY)+ÊÊJYPVPVÇµ³û=Mß¾Þú'¾Ó<Y ¬²ÞÃÏ·<ÿª¦?Ìùæ'ÿþÏx²@Y)+e]³þ~Ýçy²@Y)+e¥¬(+(+e@YAY_Öí×¼4Øh¦ÁòÄ²RVÊzï¾÷ÝPó_sÞº¨É$'²RÖ×ÙýÞsù¯yÅ² ¬²RV² ¬ ¬(+e¥¬ee¥¬(+(+e@YAY)+e@YAY)+ÊÊJYPVPÖWÖoæò?6G:´¦37Î_þ§ïðl²RVÊºâ²vpÓ:4¿5)®_ç£ÑPVÊJYW^Ö°|#þÿ< ¬²FåßNõ^µ¦ÿÕöe@Y)+e]çJ¯×·PV²RVÊ²²RV² ¬D²Ú¾lÓ¦=öÑÐÐ (+e@Y)ëÊÊfæ;::t¶¯¯Ïº¨¬¬²RV²®¬¬Ò°äÂÏÈÈ°Æ¬² ¬²RV¯¬YXX°oÚÚÚÊZÖ7Þsûµa§Áò,PVlôÕÔªªª3gÎññqû«­uuu¹ÝnmRRRkk«ý¢¹¹¹ÌÌÌØØØO|âmmmN*ër~íè;·y(+6tYëêêÌá¢¢"Ígeei>77W¹æË=Oee¥fJKKÓÓÓíåçç_¸pA·£¬>þøãZòòË/¿x§'OnÞ¼² ¬pNY§¦¦¦§§5vTÍÂ(ßrg¬Ánbb¢ý"b¾çÎûOwJNNÖx² ¬pNYÍ^K¢¡gUUO=õ¯:«^¯7ò«açÍÙâââ-[¶è6oÜ¸ÁÖ`¢¬ªêaÑÙÙivhòûý>OÃVå°¦¦&ò»k>666è¢òòrÍô÷÷klJYPVl=^|ñE=,4xµÊ:77wúôiÍí*>>>­Áºh¹á,e@YáÌ²éñÌ3ÏLNNÖ××½Ì¥W®Ùêêê·àõz+**4£SÇc¿èÄækwíÚEYPV8¼¬qqqÖÙééi=8~ë·~Ë~K.ÍÏÏG¸Ïàr¹Ün·Ùl¾f7¨´´4V6TYuú·ï¼ÄÓ@Y±A·?DN-+i(+6nY5$­­­ÕÃ"55²úÒÅ×þlúªéË_ï ¬(+e´5¸¹¹Ù¼»¦««KKnÝºõè£¦¥¥yâcnØ²ÞèÑÕL©Þ«¯ü_9øW?)«¨Éyëâök4c¦NóÔPVl1ëÌÌæAûëlIIÉF.«Ý]ËúìëÇvµÚýC9°fÌ|å;/óÔPV8¼¬IIIÍ[Zý~¿N;::ìe­ªª¢¬QÕbß¬ñëK<õÎ³÷¡é11444~¥¬e¥¬÷ôeKÌO)ëjõ÷ß¤¬eÅF,kgggff¦9ø¾NÍçÇ%%%Ù÷`joo§¬+-«üìß§¬eÅ+«	§NÍ+¬Öaë~¿²ÞCY- ¬Ø(eõz½a·gee<è;MYPV8¤¬ÃÃÃæ£iÊêóùt¶©©É$633²RV²®)«I©r«ùÚÚZûø5%%²RV²FkjjÊ¼ÎÚÓÓ£þþ~û¥½½½ôCYPV8­¬zLø|¾°W¨¯¯§¬e¥¬ëe@YAY)+e@Y)ëòæççgggÍ|UUÕÇüø ¬(+VÖ=&5¸²RV÷sÜà-[¶ùÊÊJY5¥¬e¥¬+°°° D|||ÃÓ§OÇÆÆæææ>|XgÍÁú)+e@Y)k´jjjìl^UUåv»Ù¡¬õ-¡ÓÐðmÊPVPÖ6klJY£WÜy®ôzÐdrKYÊÊúoY-,,´/	*«ùhtÊâJYÊJ(ëGeíîîN[bUó:µ>²RV²Fk~~~aa­ÁeÅê5ÂÖ`ÊJYPVÊJY)+Ê5ÙÙÙºÓF(ë»ÿ2Ãÿâj¦§Æy(+XÖ­[·îØ±£¡¡áðÍ9sfË-999Ös°w0¦PVÕTqÕÈUÓökÞ¹=ÈÓ@YáÌ1ëÇð¦Êäé×RV²bm¦¬eÅªµ§§Gªªª ×Y)+e@Y)kT¬cDT=ÙÙÙÖ¬ZJY)+ÊJY£ÚÉ:Fz°C¶SV²ÞëoÚ´uëV^g¥¬(+V¡¬zXttt<³ÄþììlÊJYPVÊº²mÂzL9rÄ~áÅ¥nÕòüü|ÊJYPVÊºê1ÑÜÜl-éííÕ¶SV²®LUUUIIMMMÖÂ¶¶6³qØÔRVÊ²RÖ¨^^7»/©¦;ÔÖÖæççkÇãÑ¼ÑöôôPVÊ²RÖHSRR¬ý~¿u°CVhM7rY·_;ýô)_&OXeÅºÜ7X¢¢"ë,epeoÄá',²b½Õ¾¿e¥¬(+V§¬ÃÃÃ§NÒ5²RV²ÞcYëëëÍüøø¸Î<yòã¹Ó²,ëCDY)+@Yá¨²vtt<»Äs§ÚÚZ=PN>MY)+ÊJYW bnnÎ¼µ¸¸²RV²®@oo¯ýìøøxWW"­­­:;??OY)+ÊJYW¶ïREEEGGGllìâ¯Â¯1kOOfÌKKK)+e@Y)k´e½|ù²©®®®­­Ý½w__Î:ujzz:èÂ² ¬õ.e-//·ÎöîÝk^|¡¬e¥¬÷XÖþþþãÇkINNYBY)+ÊJYW7ntvvj¦¥¥EcÖûöiÆô²RV²®¸¬*¨é«ÙüØciÆÄU)+e@Y)ë½léëë+))©ªª2)¨Y)+ÊJYWV5=Ün·ª555ÒÙ¸¸85aºrå¨¸² ¬5*---ö³AÇc2cÖ®®.ÊJYPVÊº>PVÊPVPVÊz/]|=Õõ¹üWíÓgÿ¼õâ7»(+@Y±áÊjÜÜÜ¬ÅÌÌÌÈÈÙ&¬³'N ¬Ñxwh¼³û½ ©¬ú¦âJYÊ¸opóÍLMMé´¡¡Á?è%XÊº"«F® ¬Øpeíïï7GÖ©×ëíîî6¹Ýµk[)+ÊJYW¦³³S#Ôòòò¼¼<ó6:­ªªÒéÁXÇ ¬e¥¬=Ù´Ä|VëÐÐæ5ÿè£nÛ¶1ëÃ*ëî¶OëÔLCÃ<yë`k°ymµ°°ðØ±c.+99Yó/_6olf³pWWÛíIJJ2Ä¼KYWª÷Ãþ§_;ªa«¦í×¼]Á@Y±ÖË:>>®GÃÀÀßôô´©AªÎñ«feeE¾ÇSYY©ÒÒÒôôôÐwìØa=æ¯ßIß+66²F¦¬RV²b­Uµf÷%ÓZk ;99©Ó¢¢¢È·g¸@ 111èÒóçÏkl=æ2337xäG(+e(+0fUYÔ=&ì±ºº:Í·´ñë´³³3òÄÄÄáááäädóîX³ä_þå_>¸ÓO~òÆ¬ ¬pHYçæætªQ©âªË/»sÑ©S§ô@IKK»ëX_"j¤ý"y»é4¯³RV²bCìÁdÛ¶móûýæ0fy½õsÅÇÇ³5XówÜ¡;QVÊPV8¿¬ùssDCµÖ>";;[§mmm¿ÖëõVTThF§'ü=cÌJYÊRV­¨¨èÑGÒÙºººsa³»¯æ].×ääär·àóùt·Ûm½(ô £¬ ¬p~Y@KKKrròìì¬YÒ××§:cDX4f~pw²RV²Â["ÊJYÊÊJY)+ÊJY)+e@YAY)«UÖí×<ýÚQMáèüeeÝeMõ^-«¾i¦üÓ÷skN¿sÐL|åuÊ@YAY7VYßûÙm+«JlËÀjÝ²­ ¬ ¬®¬vçJ¯SV²²RVÊ²RVÊJYPVPVÊJYÊÊJY)+ÊJY)+e@YAY)+e(+(+e¥¬ee¥¬eeÝeÝ~í@ÉÛ/6jjöñPVPVÊz_¾úãÒ?ëk9o]<øúiüÊ3@YAY)ëêðø3ü/ðPVPVÊJYÊÊJY)+ÊJY)+e@YAY)+e(+(+e¥¬ee¥¬ee¥¬ ¬ ¬²²RV² ¬ ¬²²RV²RVÊ²²RVÊPVPVÊJYÊÊJY)+ÊÊJY)+@YAY)+e(+(+e¥¬(+e¥¬ee¥¬ ¬ ¬²²RVÊJYPVPVÊJYÊÊJY)+@YAY)ëªúò×;R½W?sòû+ömxþ«¯QV²²RÖ`ã·þîÐøJ's?ÛFYÊÊJYWGg÷Ïå¿JYÊÊJY)+@YAY)+e@YAY)+e(+(+e]µ²~oè|åPVPVÊº:e­¨ÙýC?(²²RVÊPVPVÊºfÊ:8ñSß_SÎ[·_; ¾iðö?4²²RÖµn°)Ãÿ&eU­4SØûM~hee¥¬÷¾5Ø¾opÉÛø¡²RV²²RÖµQÖ7Þ²²®ZYehb²²®ZY- ¬ ¬²²RV²RVÊ²²âßÊê½z®ôz4Ó§¿áKíÍ ¬ ¬uY|8Yß2åô¥ùÇ¾WÄ ¬pBY»ººÜnwLLLRRRkk«ý¢ÎÎÎ;vè¢mÛ¶éjõÁQV)+@Yá²z<ÊÊJÍ¦§§Û/zâ':::4SUUõäORVÊPVPÖ»[XXÐL HLLj[¶lÑé¿øÅw§ßû½ßÓ ?KÊPVPÖ¨açíü~ÿñãÇ5óê«¯þÍ7oÞÌåýuûµæ3p¬éÓ_È½ùu~8eÅ:+«Ëå²æcccC¯0==íñxfffØüà×t|åå«æs[­©äíÅ@Y±ÎÊÌÖ`Í]:::966Æ¾ÁTYõMMAí²bÝÕëõVTThF§Ú/jkkÛ»w¯ÚáË)+e(+(ë|>_BBËår»ÝÝ¥Ybbâ&ÊJYÊÊúÀQVÊPVPVÊJYPVÊJY7^Y¯¿ÿ£?½~@YAY±:eçöàÓ¯åPVPVPV²²RVÊPVPVÊJYPVPVPV²²RVÊPVPVÊº¾Êúß¯o°O×rûµßhi0½ö ãø?ÐI±çÊÊJY×W:¿tñõs¥×íÓ_üÍw5y÷~ïËM¿_õ®|.è³çÜ¤Ì¼]Á/²:Ö»Cãé~lßNY¥¬ ¬ ¬²RV²RVÊÊPVPVPV²²RVÊJYÊJY)+(+@YAYAYÊÊJY)+eee¥¬² ¬ ¬²²RVÊPVÊJYAYÊÊguûµO¿vt]OZs?þ<x@Y)+eÅÃ/ëSãïÜþ?øó·¾Æ²RV<ü²:ÃK9o]äçÊJY)+(+eeee¥¬ ¬ ¬u¾ê½vÒEü|(+(+(+V²Îòs ¬ ¬ ¬ ¬²RVÊÊÊJY)+e(+e¥¬ ¬²²²RVÊJYAYAY)+(+eeeeeýä+¬S3Nü	(+e¥¬ ¬÷®ë'ßÌÓ°UÓökW~& ¬²²®Å²²RVÊÊJYAYAYAY)+(+(+e¥¬²²RVPVPVPVÊÊÊJY)+eee¥¬²RV¬÷êáç[ËuEzÜÐ:@YAYAY)+õ¼ý~g÷+×²ê²bu(«²²RVPVPVPVÊÊÊJY)«ó|±û/·_;ðôkGîaúýWÓuJYAY)+(ë¯85þÎíA¦|#~Å²²RVPV¬Å1+(+eee¥¬²²²RVPVPVPVÊÊJY)+eee¥¬ ¬ ¬²²RVÊPVPVÊÊÊJY)+eee¥¬Xßeíêêr»Ý111III­­­Q^DY)+(+eeÏãñTVVj¦´´4===Ê(+ee¥¬ ¬áÅÅÅ-,,h&$&&F¾èÛßþöçïtôèÑÍ7ó,CYAY)+(ëGbbbÂÎ½¨¨¨hßRSSuÏ2Ð~èA~ÒSã%oWPVPÖ05åElà$²®¦øøø@ `6ùj>Ê(+ÊÊ×ë­¨¨ÐN=OQV5<Ïàr¹ÜnwggçGweéAö"Ê²²>@ee¥¬@Y)+eÊÊ² ¬ ¬(+(+PVPVÊ²²RV ¬²eeÊÊJYPVÊJY)+PVPV ¬ ¬Q566ö=XÏ(+e]C&''5f½W.+&&&Ö´^Z»Xâ·~q7oväª=²ä¿ü7ó7eu'|ò«_ýª#W­¢¢â·û·úûßùoë[.<ñÄ@  Ycc£#×îÔ©SO=õ!e¥¬²RVÊÊJY)+e¥¬²RVÊJY)+(+e¥¬²RVÊÊJY)+e¥¬õ!Ò³óõë×¹j½½½ßøÆ7ú+))ùñìÈUóûýååå_þò/^täÚµµµUWWPV(+uêêêr»Ý111III­­­X£ÎÎÎ;vh¶mÛ¦µsä:677[sÌÚÍÍÍeffÆÆÆ~âhkksØ/Nk´uëV³.>Ïk711áÉÄyz ¬Qñx<)--MOOwÀ=ñÄ©ªªzòÉ'·óóóú×Á*«cÖ.??ÿÂÐã?î°_\ðð°ftjj´Þ×Nÿèûa~C×ÈyO/ ¬ÑþÁë¹lqiEû¿Î°eËç­ãùóç­g4Ç¬5NpêÑÑQÍèÔüß°Þ×nÿþýö²®³^@YvÞü~ÿñãÇ¶ñ$''ëÙÊzFsÌÚéÎë!çÆûÅuuuiÕô[Ó©Y;YC×ÈÁO/ ¬¸k>66Ö1ë5==íñxfff¶iiiíííög4Ç¬VÄ¼Í¦¿¿_ÿ=8ì·sçN3"WbSRR³vö²®S^@Yï">>>,.m®Ñ¼3Vjtt433sllÌyë¸éNNZ;û7ã'ýâBGoÎX;YC×ÈO/ ¬wçõz+**U¤AÖ¨­­mïÞ½^Gû3cÖîÄæ½½½»vírØ/NãT­fzzz4~uÌÚÙËºFüÓe½;Ïàr¹Ünwgg§Ö(111hTç¼u´?£9fí¦¦¦ÒÒÒ4KNNpØ/îÖ­[ªÖN§wÌÚÙËºFüÓe²@Ye²@Y ¬²@Y ¬²@Y ¬²b5óUUUö¬§§GWÖØZ7u÷ý¦Ma?äKËóòòìßÛ¦M©©©EóZÁã ¬ÀýjiiQT>O»fwwwÚ"uQ_mÅ©²²ÒDËË-»wïÖò-úH/-)**ý.CCCºHß«½½=IPÿZÁàà`Ø´ð©§]¸oß¾Ðæ'²+À)f^i´UÃYû5ù	aaaaä[6'>33£ùººº K/]ºdÝ uµÞÞ^WË5üµ_jUäojillÔ?Y<´°££CËUw'e¢¢t©ñññKN>kÛ8¬å[·n½k³íeÕXÓ»ÄºñÒÒRºÝnsý@  ³àjÞåríÜ¹³vîÞþýû#tø|>]óìÙ³ËÝ1	Ý|òäÉÐ+÷õõ±¡ ¬@´±ë¬âguî®ÃAçYbSvvöre-//·AÍÕ~³ö²Ú·*k~Û¶mÑÕÜÎ¡C"$?ìßÐ1«öeßqEe5ÃÜ#GÌÏÏÛKi^LÍÏÏjÐyåÊÍLLLØ_j½kY÷ìÙc]s÷îÝÑUßQW°ÖÖ 8²KxÀ¸KV^³*«RúUê­%½½½ZRPPzûö1«Rgöòù|ÑÕTÙdÒëõF_Ö±ïmÌ*¹¹¹Ñï5²bãuvvÖÚã×þ:«æMKª««Ò[RR¢åMMMÖÂ¶¶6³qØÔ-«[=¸1ë't5ÓÈéòuÖ(k²±oÔ¼5¸±±1>>ÞdF5Ý¹DcÊüü|óæTÍmOOÏre¶ZõÊªÿÌûv"dòüùó+³F³ÇÊPV1%%ÅÊßï·¶·¦`.@[Í;4]î?°ïRPVóÛ ²÷UÖ Ùó¡¬&ÆfÌÚÒÒ²¢²feeY×<sæLôe]®uSALæÇÆÆ¢,«)qrr2²«PÖÙÙÙÐýöWPÖ+W®-Þ¹sgØ£)²½²kÝúúúææf3f]ÌëÁAJ4;6=zbØ²vuuiùO>Éc ¬À]¨1[·nÝ±cµ#f44Ü²eKNN¿	:UÖááaO:eÞTý·»CPØ¡­e||>öØcÑ¯cgggÐw	ÝMéÆaÿÐÂÓ§Oóh(+°oªêñºiFöàEØ«výmÚtùòå°E9@Y ¬²@Y ¬²@Y ¬²@Y ¬PV@Y ¬PVêÿ×D_Q¾®IEND®B`


KM time BY M_distant_metastasis
  /STATUS=status(1)
  /PRINT TABLE MEAN
  /PLOT SURVIVAL
  /TEST LOGRANK
  /COMPARE OVERALL POOLED.


Kaplan-Meier


附注	
创建的输出	19-JUN-2020 20:46:36	
注释		
输入	数据	E:\医学\肿瘤内科\孙佳春老师的肠癌统计数据\modified\data_modified 赋值.sav	
	活动的数据集	数据集1	
	过滤器	<none>	
	权重	<none>	
	拆分文件	<none>	
	工作数据文件中的 N 行	55	
缺失值处理	缺失的定义	用户自定义缺失值被视为缺失。	
	使用的个案	对于分析中的所有变量而言，统计量以带有有效数据的所有个案为基础。	
语法	KM time BY M_distant_metastasis
  /STATUS=status(1)
  /PRINT TABLE MEAN
  /PLOT SURVIVAL
  /TEST LOGRANK
  /COMPARE OVERALL POOLED.	
资源	处理器时间	00:00:00.17	
	已用时间	00:00:00.20	


[数据集1] E:\医学\肿瘤内科\孙佳春老师的肠癌统计数据\modified\data_modified 赋值.sav


个案处理摘要	
M分期	总数	事件数	删失	
			N	百分比	
无远处转移	36	30	6	16.7%	
有远处转移	19	19	0	0.0%	
整体	55	49	6	10.9%	


生存表	
M分期	时间	状态	此时生存的累积比例	累积事件数	剩余个案数	
			估计	标准误			
无远处转移	1	1.000	删失	.	.	0	35	
	2	2.000	死亡	.971	.028	1	34	
	3	3.000	死亡	.943	.039	2	33	
	4	3.000	删失	.	.	2	32	
	5	4.000	删失	.	.	2	31	
	6	6.000	死亡	.	.	3	30	
	7	6.000	死亡	.	.	4	29	
	8	6.000	死亡	.852	.061	5	28	
	9	6.000	删失	.	.	5	27	
	10	7.000	死亡	.	.	6	26	
	11	7.000	死亡	.789	.071	7	25	
	12	8.000	死亡	.	.	8	24	
	13	8.000	死亡	.725	.078	9	23	
	14	9.000	死亡	.	.	10	22	
	15	9.000	死亡	.662	.083	11	21	
	16	10.000	死亡	.631	.085	12	20	
	17	11.000	死亡	.599	.086	13	19	
	18	12.000	死亡	.	.	14	18	
	19	12.000	死亡	.536	.088	15	17	
	20	14.000	删失	.	.	15	16	
	21	15.000	死亡	.503	.089	16	15	
	22	20.000	死亡	.	.	17	14	
	23	20.000	死亡	.	.	18	13	
	24	20.000	死亡	.402	.088	19	12	
	25	21.000	死亡	.369	.087	20	11	
	26	22.000	死亡	.335	.085	21	10	
	27	23.000	死亡	.302	.083	22	9	
	28	23.000	删失	.	.	22	8	
	29	24.000	死亡	.264	.081	23	7	
	30	26.000	死亡	.226	.077	24	6	
	31	29.000	死亡	.189	.073	25	5	
	32	36.000	死亡	.151	.068	26	4	
	33	39.000	死亡	.113	.060	27	3	
	34	57.000	死亡	.075	.051	28	2	
	35	58.000	死亡	.038	.037	29	1	
	36	87.000	死亡	.000	.000	30	0	
有远处转移	1	1.000	死亡	.	.	1	18	
	2	1.000	死亡	.895	.070	2	17	
	3	3.000	死亡	.	.	3	16	
	4	3.000	死亡	.789	.094	4	15	
	5	4.000	死亡	.	.	5	14	
	6	4.000	死亡	.684	.107	6	13	
	7	5.000	死亡	.632	.111	7	12	
	8	6.000	死亡	.579	.113	8	11	
	9	8.000	死亡	.526	.115	9	10	
	10	13.000	死亡	.474	.115	10	9	
	11	14.000	死亡	.421	.113	11	8	
	12	15.000	死亡	.	.	12	7	
	13	15.000	死亡	.	.	13	6	
	14	15.000	死亡	.263	.101	14	5	
	15	16.000	死亡	.211	.094	15	4	
	16	23.000	死亡	.	.	16	3	
	17	23.000	死亡	.105	.070	17	2	
	18	24.000	死亡	.053	.051	18	1	
	19	31.000	死亡	.000	.000	19	0	


生存表的均值和中位数	
M分期	均值a	中位数	
	估计	标准误	95% 置信区间	估计	标准误	95% 置信区间	
			下限	上限			下限	
无远处转移	21.771	3.613	14.690	28.852	20.000	4.014	12.132	
有远处转移	11.789	2.043	7.786	15.793	13.000	5.804	1.625	
整体	17.983	2.422	13.235	22.731	14.000	1.724	10.620	

生存表的均值和中位数	
M分期	中位数a	
	95% 置信区间	
	上限	
无远处转移	27.868	
有远处转移	24.375	
整体	17.380	

a. 如果估计值已删失，那么它将限制为最长的生存时间。	


整体比较	
	卡方	df	Sig.	
Log Rank (Mantel-Cox)	4.799	1	.028	

为 M分期 的不同水平检验生存分布等同性。	

Þ¾[û¦û÷ï¿ïÝ´¯¯/à2Ç~Ë4WfÃrÍÌÌL)º¬7ÍÚ-¨ÍÅ(+Edªª^ÈÔ6ÌÖ`Çãk«æiÎ;'ëå4 ÞXê_=iOOÒÓÓ%®ÝÝÝc¹èÒ¥KrZ[[+§r¤Ý¦ß @Y¨°¬¤¬ÁMLLØívíõÚ+ËìSmmmjû­VVVÊ©Ú×IT¼ª~åêkÝnwgggWWÄXÎª#ü9PVàéQóNýë¦Å£ÊXõÖµ9WÿËm7õ÷î½ï/ÍÅGFFdMLLvÖÖVY£¶«$®ZqÕÏ eU7%óìññqHÈù%®	«DKBµß/øuVËýWõööfggì.÷Â§VVUDª.ªíÉeÝÝÝGÕ~5P×ljjÜªÙw	 ¬@4òù³s8r¹¾Jädî§âg6ý;&gµý~UU&çææîÿåuVI©âââÊªö`êïïWÕÛX«ªªä"5ßU/Áò»(+°1&²ËUJ)9Ô®&ÓÜ$³ÖÕÕ%''ë×TTTh©VóW­¬Úu$ºê5×m½e|!¿,²»¬WS;%EXVµ5XJ|áÂÜÜ}ez0gÕLLLìÚµKMRµK¥èû3 ¬ÀF*ëøøxÀ[kV]V¥¶¶¶³³SeU;X©c9©fÕfaþªc?Éß@Y(réÒ¥GñMIIÑÎ.w°Yå:²æØ±cÁßE½(«"ä¡O:%WPÇëwttÈÏ£¿¡¡!ý1($ÿêfçççùUÓ¤©­Jòàà`È+Ë3`ãíííòåÁ3cerrR*|	°Úe²@Ye²Fàü£6>"DY£ÅÔÔÉd²ÀF¶Üç.²®OY¿öµ¯ý62ÊJY)+PVPV ¬ ¬ee¥¬@YAY²²RVõêííAMMMÈj(..ëÈ °°pbbbvv¶±±±ÃïôéÓË e¥¬`¨²¦¦¦¶¶¶¬liiõ+<ØÓÓ#Ã¡«×ÙÙyäÈÒÒRéè=ÊËË´O*<zô(sVÊ¢¬òí$¢ú9¨wìØðc?~=¸¥¥¥üüüîîn«ÛÑÊò£)+ecµ¬¬¬¶¶V[#ãÃjµNOOëg¨5557%eÕæ¬ª¬2ÍU[ÓÓÓ)+eÍRÖÑÑÑmõ?Fee¥Ú¬wæÌ'NLNNêËÊ²eýó·ÛµkzétpppÇÚz!±looïxúäºº:NLL³ÎÎÎæææRVÊ±¬2+-((Pu<wî¶^WeF«^FU»;i»8&OÀµ¯¯ïðáÃ­~l¦¬°¹Ê:66¶mÛ¶gööíÛú9«~Ï&mêÙÔÔ$qÕï¾$SUý¾ÁõõõeeeÌY)+lÆ²Éaff¦ökðQ[[ÛÖÖ¦ÆÕÕÕíííúK%½UUUÓÓÓjÃ²Ì¥²²>påÚÞ6UY%2.//YÖùùyÍ¦ÍÉÉ	~KëÉ'ÕD¶°°°§§'==]mC¦¬õÏÒlùó°©Ê:11!c¯×Ës¬»ºº¤»wïÖª©ÈVÛ><99YZZêr¹Ô.Q¼ëfË*¿¹ÄÄÄàõn·Ûb±ÄÄÄ$''Ëï² ¬OÌMõiZXXÈÈÈØ,O¿RPí¬Çã'mí¬ÌóDYÃòQeµZÔöù¥ÊàîÝ»CûàÌf3e@YAY8xð`ÀÛ5qqqjËÏçSÚììì-AyæU?Tÿw	jðû_ºùS@Y±_gù¨	?9kKçðÙêëü© ¬0ZYM&6|>µ×Y)+Êc5>>Þçó©­Á2¦¬(+(ëcÕf³Õ××Ë@N­Vë.ë®þgÊ²ÂeUgGBBÉd²X,N§óIõ¥+(+ÊuÕ(+ÊÊJY²RVÊ²Øeõx<Aòòò´O¡éïïWªÚÓÓ£]áÔ©Súq¹ÕÕj<??¯>ÀUï­·ÞRoßPäË*,gÔ1åöµÏÌ¡¬´¬i¶ËµM7ÔróÖ]þì¬Y¿Ùõú¹k!¹¨ég·ôIÓÆYYY!o-;;û¾ÿcàûv/^lllTc)h~~¾vÑÄÄrÿþý­­­uuu½½½RM«Õ*+å³³³2/©¬¬Tõ=rä^ºt²nÆ²JJµ¬Jb%´üÙX÷²ÊÓÑÀÐTÈå|í¯Ïþ¿DÂ»ÜÊÌU?©ò>ZÞ¦¦&Y/3W©ãñãÇõ_+5mnnÖ¾KKKÃá¸é®Ë´Undaa¡¶¶V7SÖÍUV½³Õ×)+()ërýÂá¸ê'´M&j,sÐcÇIÞ¼^¯ê¥åúyyyrªê'µ§°°PUº«u±´´Tfv»~~^VÊ-K>ePRR"·?>>®æ¯r5¶ÊµT¼¬¬L~¶SVÊ`U¿Wò&ÓDuÈý¥KKKÚË¥ÁeVUUÉ ¡¡Áåri[­Vëèèè´4©¦´¶¼¼à5Ú%?I²³ÆÆÆÊJY)+WVÍ3gÊª¨pÞ¾[]ªÂ933£.U¯NOO8qBÿ:«æª±TSmgffJÅe+7¨½4»sçÎÚöôôÈuvïÞ-óZùvÚw¡¬lYëzó?üb¹%LY»>øüïÞ¸°RfÇ:)kee¥:«Â©6öJùT Ï;'Íà=¶¶6ªÊv~~~rrRÖÈ$Uû,t¤ÖÔÔH%«G½+×´Ùlú=)+e'XÖ?,,~95¿Ü2õÕÂ<ÎÍÿ¿52aÕUËDW¢8<<¬íE'W;|ø°~oá¬¬,õÊ¨Qg¹Yivqq±³ÎÍÍÈ»ÝnmFÛ××'JYÇÆÆdÂª¶*ËwQ¦¬º¬ÿðO·|ªÆW®ð÷`½¶¯Ýnïìì¾¬ê/CCCÙÙÙ2+­­­3¼páBWWWyy¹ÌVÛÛÛ½^ïÂÂK¯¶5ø¾ÿÍ¯êßö#UÖ^g+È´UËë¬õ÷¶×;Þ¹ð¡k3Q[Vµo°<7ª³2ÝÜ·o_¾_jjªL+e©'§R»£~GyþùçåÒ]ùÚÖÖVµ§zFæäädddHgggµ²JzõÏÆú7í©»Çãõþ¦DY)+ÌYAY)+ÊÊjè²¾üVéhKk»8 ¬ ¬ÍYõû³²ó0¨-ëÐÐPQQQðzÈOY)+ÊzÿÆÌÍ_5¸Üòùü]u5udA%++«¬¬L;,ðþýûµ=8"?e¥¬6Yå9êDßÛ!¹ègµ¢;NOOgff¹AÈOY×§¬NÞýrZn~AY¬cY»ègã¿ü¯ÊÕUM)ª­²#òSÖu.ë#QVQXVnæäähgeî¨U#òSVÊ²®¬¬]]]F5ÅT¤ÚÆ[#òSVÊÀøeuþö×?ùÉrKø²¦ð=­yRDDRVÈOY)+Í[Öÿùo?]îðóù»òµ+ËËËõ[õeåü²`kðie|vuuieåü² ¬«a·ÛÕ@&=Ç£ÎrD~ÊJYPVPVÊ²æ²ö¡¢¬Ö¬¬i¶Ë²RVX²z< yyyÚ±	£í³nÕ!»ººd í%ßB¾uÀ3226Ð;b)+e`²êò:QõY7v»nnN®_SS3==ÝÜÜ,]êëÐèp8NgDY£´¬ï´/·îPVqk°377w¹K£ç³nË*_(×ÔUæÜUtõË]¯¢¬Q]ÖýçN|>`Q¹¥¬BY?úd²á_[¤¬a.¯ÕO(iLÕX²tìØ1É[~ÖÍ#Ë*³pmÓ4sÖTÖ$®À*«~¯ÄI¦úÞ¾ÏºËË¬Wþ ßE²*ßE:ªUnJò,!WÿEØ·oÈ5¥÷a6hSVÊ­Ák¼5øÌ3eU¢ç³n$òê[,7gÕòév»¥ÐZ°³RVxzebÉNB¦¬ëõY7[þB]Yíx¬/kgg§dXÿ:«"?×ëérOU)+e`À²VTTÈU_V-ÑöY7eeeª¬`UÖññq¹²<yRm@V/SVÊO©¬v»]¦2ÐU½ã%Ú>ëFnS&Ð£££Gq:^¹Yù.òíô;$K¹Õ[zùÙ8 Ìe¥¬°â²FxtCµo°<7ª³2ÝÜ·o_¾_jjªL+£í³näG«·ÆÊÈdZmm.))Q[§zzÔYõä6333åîPVÊ«)+6"ÊJYPVPVÊ`s5ãúk²RVX³²¾tåÐJ!×sD~ÊJYPÖHËª,¨deeiÞ¿¿¶gGä§¬Ñ[ÖÌÂ_ýÀÍó()«:² z¿MfffkrD~Ê¥eµ½ÞñÎy^ðøeÿÓîþar¹EÊæÒÿ³ø%«*B2ST[eGä§¬Àf)ëF~rèÚw[¤¬a.ýçëeº©ðI¨´ªqD~Ê¥e=ÿ÷Ùêë²¤Ù.Ë"åïßþ%OÖwkðÿñ$j©Hµ·Gä§¬QWÖwßjéE²úwotÊ´Uó`Ë*Í8É ²rD~ÊíûSVÑ0gUÊËËõ[õeåü² ¬«/«ä³««K++Gä§¬Ñ^Vûÿö8ûîPVOº¬oÜ8»¢§G»Ý®2¡ìiâü5ÚËªGY<¹²b#¢¬ee¥¬(+(+eÊJY)+e@YAY)+ÊÊJYPVPVÊJYPVPVÊ²²RV²e¥¬² ¬Øeu»Ý%&&&99YpçûþÃ='%%©e@YAYÍjµ644È ºº:##CQ\×ë&&&RVõÑ$êü|>_@>_xáñññûþ¹±N>ýâÃd½Lj)+ÊÊút1äXm(ªÏökooÿ§mÝº² ¬ ¬L&ml6õíÚµKT¯$655­Á(+(ë£ÅÇÇû|>µ5XÆNg)+ÊÊÍf«¯¯Z­VýE2OÁÀÀÌ_×¥¬ù¿tåÐËï¿*YÖÚ¦jùy÷-,PVÊºGBBÉd²X,N§óÁâÝ¾[*³U9ñºuü«[÷FÔòÈ²Ö7¢²úZÉÕWòßãÉe¥¬Ï.«Þ#ËªqöÝ¸òd²RVÊJYPVPVÊ²²RV ¬²RV² ¬ ¬ee¥¬ee¥¬(+(+e@YAY)+PVÊJY)+ÊÊJYPVPVÊ²²RVÊ²²RV² ¬ ¬² ¬²RV² ¬ ¬(+e¥¬+*kírKç°ZF½÷xâ@Y)+e]Y?xûüµ³Õ×eQåe¥¬uõeÕ¸RV²RVÊ²²RV²e¥¬² ¬ ¬ee¥¬(+(+e¥¬(+(+e@YAY)+ÊÊJY²RVÊJYPVPVÊ²²FYc.µÜ¼EYPVPÖÇrôÚw³Ê"Íÿ¨² ¬ ¬kãÝ¶Ó§¬(+(+e@YAY)+ÊÊJY)+ÊÊJYPVPVÊ²²nª²W:%®Î¾;²|ø±g²RÖÇ*kó'ot¾VrU4ÛeAPVÊJY«¬ze¥¬² ¬ ¬ee¥¬(+(+e¥¬(+(+e@YAY)+ÊÊJY)+ÊJY)+e@YAY)+ÊÊºËzi¤ý¥+´¥å_yYÏV_¥¸ÒYwqg²RÖ@2y)lWþy÷­ÎaY$®otòl²RVÊúXeÕ8ûî¼Vrg²RVÊ²²RV¨¬òØ·o_fÖÖV¹¨¬¬²RV²®¬¬éééjÜÛÛ+g=vQmm-e¥¬(+e]YYE«_ii©³²²´9k[[e¥¬(+e¥¬eÅúµ¼¼KKKú-Ã¢¹¹ynn²RV²®4Õn·ÇÄÄ<yÒçóMNNê_mÏív[,ùÚäää®®.ýEÙÙÙf³ùùçïîî¦¬(+6EY/]º¤6WTTÈ877WÆEEEÛH¾Üjµ644È ºº:##CQIIIii©Üdõ^5?ýéOßzX~~þÖ­[7bY3ßèültR-S¿ýg²²>0333;;+sGIcBBBGGZá[nâââTe²¨¿Hf±ß³gÏþû¥¤¤È|wÃuàæi¶Ë¯ä¿'Z:yf(+(ëj¯%!SO»Ý.Ë½÷ÊäUÎÊJÍþË¥!Çêleeell¬Üf¿¶ë­¾NYÊJ(ë_ÉTµ¼¼N§SíÐär¹L[%/^ÿå&IÍæêêêd044$sSÊ²b³ìÁôÖ[oÉÃB&¯ZYe°SR°øøxÏ§¶Ë8à¢å¦³e1Ë:11!ÃOOO·´´¨½Z[[Õ¥r¶©©)Ì-Øl¶úúzÈ©ÕjÕ_§¾vppp÷îÝeÁËêñxâââ´³³³³òàxöÙgõ×¹páÂââbq8			&Éb±¨íÉjâ«vJOOÙjJJÊðð0e@Y±Y¶¯#Ê²Âhe)iss³<,ÒÒÒ6aYó?*þ_þ­ôU¿ì?WóSV²®xkpGGzwÛí5·oßÞ¶m[zzºÕOWûÐ¨euß½!sÖEâzôÚw)+ÊJYWcnnNêÓÓÓûËÙªª*5$Ç+Ëõ&e@Y)ë,--%''Ë¬Tí¬ÞÒêr¹ä´··W_V»ÝNY)+ÊJYM½µ¶¶V£££óWÊJYPVÊºª/óSo<¥¬eÅcÕétfgg«ïË©úü¸äädýL===² ¬5Ò	«SNGFFÔ+¬Úaí.²RV²>Ùl¶Ùl!·çææ=é² ¬0HY½^¯úh²:9ÛÞÞ®MY)+ÊJYW@U¥Tr+ãææfýü555²RV²FjffF½Î:00 ¡¡!ý¥OôCYPV­¬GÃÃÃ#äZZZ(ëJËz÷ËigßnÊÍXÖõeÔ²JV_+¹ÊÓ@YAY)+e@Yñxe]\Wc»ÝþeÑÊÚÙÙ)ÊÊJ'úQÖµ¸Òf»~ù_?óðÔPVl²Ê"66Vôeé,e]ýÊMTÖ¥¥%y@ÄÇÇ·úÍæ¢¢¢ÌÌL9«ÖOY)+ÊJY#uñâEýÛívÅÂÖ`)ëKW½;Ò¶åÞÌe(+6oYåÑ sSÊàæä­üO|~¥ôøÖ½ß?ü~VÊPVl²ÊC¡¼¼& ¬ê£Ñ7aYWíå÷_UeÕ£¬eÅ&*ëüü|___ºþuVË©öè² ¬5RKKKl¦¬(+Ö¦¬a¶SVÊ²RVÊJYPVDqYçççè~LeAÊº´´´sçÎÖÖÖL?<y266öôéÓÚu°'w0&Ê²Â8sÖ§ð¦Ê*Þ>-Ívùµ«+Z¾ó]çàæ	 ¬ØØ[)ë(ëðgÎ¾;+]jnHÂ(+6pYäÁa·Û^g¥¬YÖÕásèÊWVíê%U«Õ*S§Ni/²644È´´4ÊJYPVÊÑNLÚ1"¤pÁ;dk0e@Y)ëj¿xË¤¤$^g¥¬(+Ö ¬mmmò°èíí=ì§ËÍ©S§(+e@Y)ëÊ¶	Ëcâøñãúß÷t«¬/))¡¬e¥¬+ ³RyLttthkeMYY[)+ÊJYWÆn·WUUÉc¢½½][ÙÝÝ­6kZÊJYPVÊÑË«ñññj÷%©é.¿æææYcµZe¬f´5ÿájcÌ%qåóß~AYÊMTV¯×ªíìr¹´ÊlõÖÔÀeý=W¹¾tåRV²bî,í,e|2sÍr½IYÊÍ[VýþJ² ¬X²z½ÞY#Í£¬e¥¬«,kKKONNÊÙüüü§óCSVÊPV°¬ë²RV²ÂPeííí=âgXss³<P)+e@Y)ëø|¾àA,,,¨·±VVVRVÊ²RÖÔt»Ýòéêê³² ¬ueû.Õ××÷ööÍæû9¿ÌYd  ]MY)+ÊJY#-kMM455577ïÙ³ÇãñÈÙÙÙÙCSVÊ²RÖGµ®®N;«v¿¿zñunn²RV²®²¬CCC999²æôéÓje¥¬(+e]qYûûûN§:;;eÎzàÀ¨¾RVÊ²RÖUªúª¶?÷Üs2Pq² ¬u5[ÇÆÆ<OUUÝnWIP³RV²®¬jòh°X,2U½xñâ±cÇäl\Uajll5RJYPVÊÎÎNýÙã1©9«Ûí¦¬e¥¬e¥¬ee¥¬õ¥+$®²¼üþ«¿úüÊPVl²ª#wttÈÃbnnnllLm³yyyuÕîÍLIrôÚwßi£¬eÅfÙ7¸ÃO333rÚÚÚªÊð,e]µÓ§¬eÅf)ëÐÐ:b°Úl¶¾¾>ÛÝ»w³5² ¬ueN§ÌPëêêÕgÚÈ©ÝnÓ£G¶úiÇ ¬e¥¬á¨=Ûâ§>«uttTÆmmm2Þ¶mÛ;³RV²FJ½¶Z^^~âÄ	É"ãõÆÖH6»ÝnÅ¬>/=z²RV²ÂøeGÃðð°ßìì¬4R&©rVÍ_eennnø±Z­2¨®®ÎÈÈÞ÷xçÎÚcnddäúÃäÍfÊìÃ½i¶Ë¯ä¿'ÞýùM¼Ê¨.«tTbªÝTkµìôô´VTT¿¸¸85Áõù|;wN&ÁÚc.;;Kgy²ôÙè¤ZÎV_¯mºÁ@YísV)«µµµUÏ=÷Ü¥Kd ÞÒªÄ/§N§3üÄÄÄ¯×¢Þ«Öüîw¿»û°O?ý9ë#IV)+@Y±Êº°° §2+¸Ê ¦¦Æd2©äþÈÑ¾DH#õÉ÷ôô¨Nó:+e(+6ÅLjB¹cÇË¥¡Ö·´´h0^||¼ÏçS[eüÐô0ÊJYÊãUQp®h(­U8uêvwwÿZÍV__/9µZ­¡2æ¬ ¬Ø$e°UTTlÛ¶mffFÎ^ºt©ªªJCXíî+cÉ4==½Ü-8¹ÅbÑ^xQVÊPV¿¬>¯³³3%%e~~^­ñx<RGuÌY½^ïû¡)+e(+¸5xQVÊPVPVÊJYPVÊJY)+ÊÊJY)+@YAY)ë)kíòÙêë²¼þÚÝ/§y"(+(+e]½Os·¥sX-ØÏF'y"(+(+e]¯ä¿GYÊÊJY)+@YAY)+e@Y)+e¥¬(+(+e¥¬ee¥¬e¥¬² ¬ ¬²²RV²²RVÊ²²nº²xµµ¥sØÙwGOsg4²²RÖÇòÆ;ï¯¨ûµ«ÿþg2å ¬ ¬umÈUúÊ3@YAY)+e(+(+e¥¬(+e¥¬ee¥¬ ¬ ¬²²RV²þÕ¯>ø×ïêâ ¬ ¬umü¤åÓÃ'~Æ3@YAY)+e(+(+e¥¬(+e¥¬,kó'i¶ËÁËÆ~ÝÊÊJYWO¿opmÓYxv(+(+e¥¬ee¥¬e¥¬Õxe½ûåôµÿ²²®=ÊPVPVÊJYÊÊJY)+ÊJY)+e@YAY)+e(+(+e¥¬ee¥¬ee¥¬ ¬ ¬õ	õlõõÏF';ßã) ¬ ¬Fózß?¾tåÐËï¿*F§¼kþ-ÞýùÍ4ÛåWòßÓ/jOyee5/g&oÝQËßüòoåôé|_ýPVPVci+e(+(+e¥¬(+e¥¬²²RV²²RÖ(-ëG7>ÿ^Q7Oyee¥¬kã³ÑIÞuPVPVÊJYÊÊJY)+@YAY)+e@YAY)+e(+(+e¥¬ee¥¬Æ(kírKçpå½#<'²FäîÓo¿v¶úúrKÁÙI/Ïee¥¬l.(+(+e¥¬ee¥¬e¥¬²RV²²n/]9tâÃ·N|þñýçªo6PV²²nê²¶ßùÕ»#mk²H^û.e(+VV·Ûm±Xbbb»ººô9Î;wÊE;vì«QÖµåse¹Þ¤¬eÑÊjµZdP]]¡¿èÅ_ìííÝnß¾;e¥¬ee´¸¸¸¥¥%ø|¾ÄÄÄå®+§¯¿þú¿Ø·¿ýmÔògIYÊÊút1äXÏåråääÈàêÕ«ÿü°òòò­[·ògIYÊÊúÉdÒÆf³9ø³³³V«unn­Á ¬ ¬ïóùÔÖ`>>=11Á¾Á ¬ ¬±Ùlõõõ2Sê/êîîÞ¿¿´3ÌSÖõ-kírmÓµZ>üØËïõq9Éd±XNçÅÿ KLLÜ¢CY£­¬÷¾ZÃ¬ªHóee]guËº¶T_ù½²²RVÊJYÊÊÊPVPVÊJYÊÊJY)+e(+e¥¬² ¬ ¬²RV²RVÊJY)+@YAYAYÊÊJY)+@YAY)+e¥¬ee¥¬QRÖ4Ûå×J®.·¼ÿ^k×0¿8PVPVÊJY#rçóÎ¾;a+ZPVPVÊJYÙPVÊJY)+e(+(+(+@YAY)+e¥¬e¥¬²RV²²²²RVÊÊÊJY)+e(+(+e¥¬ee¥¬ ¬ ¬²RV²RVÊJY)+@YAYaÀ²Ö]H³]~%ÿ½½È]øouñðe¥¬²®¿©ß~õÙèäF_dÚýU×yø²RVÊJY±6Z:ÏVSVPVÊJYèú¿~éÊ!m©ùÍù7¡¬ ¬ ¬umTÝ¬ÊÊÊJY)+eee¥¬²²RVÊÊÊÊÊJYAYAY)+e¥¬ ¬ ¬²²²²²RVPVPVÊJY)+(+(+e¥¬Ðµàlvá©ß~Å¿	(+e¥¬«wåÚö=2ÐòoÊJY)+eÅÚ8[²²RVÊJYAYAYAYAY)+(+(+e¥¬²RVPVPVPVPVÊÊÊJY)+eee¥¬Q]Ö®Êr½¹&ËËï¿úùo¿à_²²²n^£S^Çk­ô­#ü«RVPVPV¬³RVÊÊÊÊJYAYAY)+e¥¬ ¬ ¬²B)®tJwXV·Üü²²RVÊ¿ªoþ$óÎ×J®²¬bº4ÛeÊÊJY)+°6>|%ÿ=ÊÊJY)+@Y)+e¥¬ ¬ ¬²²RVÊPVPVÊÊÊJYAYAYAY)+(+e¥¬ee¥¬ ¬²RVPVPVÊ·Ûm±Xbbb»ºº"¼²RVPVÊÊÕjmhhAuuuFFFQVÊÊJYAYC[ZZÏçKLLÑ~ô£ï=ìÕW_Ýºu+Ï2²²>rò¢KKKx*78þÕÿx¢&ïUÛt²²`2´±Ùlð"¶0ÊJY×R||¼ÏçS|eáEeeÍf³Õ××Ë@N­VkQV54Ã`2,Óé|ð£ød!/¢¬(+(ëDYPVPVÊ²RV ¬ ¬@YAY)+ÊÊJY²²ee¥¬(+(+eÊJY)+PVPV ¬ ¬e¥¬²eeÊÊAYÍfóØÈ(+e"ÓÓÓ2g5¯Éd1Ü/¹wfâ·qq[·n5ä]ÆoÕ_þõ¯QVØ¾û;ï¼cÈ»V__ÿo|Ã¨¿¸o~ó?üáy×JKK_|ñECÞ5Ï'3³¶¶6CÞ»½÷PVÊJY)+e¥¬ ¬²RVÊJYAY)+e¥¬²²RVÊJY)+e¥¬ ¬²RVÊJYAY×<;_¿~Ýwmppðûßÿ¾QqUUU|ò!ïËåª««3ä]ûÓþtþüùCÞ»îîî¦¦&BÊe²F!·Ûm±Xbbb»ººpNçÎ;åíØ±Cî!ïcGGv8ÃÜ»ììl³ÙüüóÏwwwì'÷())IÝÃa755æÉÄxz ¬±Z­2¨®®ÎÈÈ0À=zñÅe`·Û·oßn¼û¸¸¸(ÿuÐÊjWRRRZZº´´$záöóz½2SU£~ïäÿòýa~ïñ^@Y#ýç²ûþýõÿý4ØØXãÝÇsçÎkÏh¹w2­ñx<FpÊÿÆÇÇe §êÿýÞ<xpxxX_Öàdì§PÖeÅÄÄËåÊÉÉ1ØOJJ<[iÏh¹wòÃWVVÊ$<ýýýûÅ¹Ýn¹kò[SydæÞéË|üôÊÉdÒÆf³Ù0÷kvvÖjµÎÍÍì>¦§§÷ôôèÑsïä¨·ÙÉÿöÛµkKbSSSsïôe¾GFzeøøxÏwß¿¹FÆÆ¸SãããÙÙÙÆ»[f¤§ÿáÕüÆH¿¸àÙ1î¾¬Á÷ÈO/ ¬f³ÙêëëïûU$<Ü£îîîýû÷OMMø>êÑsïòòòÔwïÞm°_ÌSå~É```@æ¯¹wú²ß#Cþé²>ÃáHHH0LÅétà%&&ÌêwõÏh¹w333ééé2KII6Ø/îöíÛT¹wr*cÃÜ;Yï!ÿô@Y ¬PV@Y ¬PV(+ ¬PV(+ ¬PV(+ ¬óóójl·Ûõ$ÞÀÀ¾D¿R»©G?è·l	ù!_²¾¸¸øÉþ½mÙÖÚÚù2YYY<Ê<®ÎÎNJeeåÿÇáå®Ù××î§R$]/<uê§-uýØØØ=öÈú/Êúô5ÁßettT.ïÕÓÓáÐ¿122ò?²rïÞ½Á+8¼RýË ¬À&p@54êË*ÓYý5üÔxjjJ¾°¼¼<ü-«ñ¥K.½pávÚÕ¥¸²^¦¿úKµ¯ÿM5mmmråÈËzôèÑ½½½²^êÎã ¬@D$]RøøøV¿ÂÂB³Ù¤MÛ8,ëÙlYe®ióÓn¼ººZN-º¾Ïç³2Á±ÉdÚµkW³üx?Óàp8ägÎYîÁ[óóó¯ìñxØPPV R±ªª*í¬ÄOëÜ#§2;ì§Ó©S§+k]]~ªæ¬úÕU¿UYÆ;vì¼¬êv;&ù!7üÏY¯( ¬@èÆHWTV5Í=~üøââ¢¾êÅÔ©¡L:e055¥©õeÝ·ovÍ=öD^Vùrµááá0÷ZGRVuirr2²ÈjÀkeÔÌJå;::´5²¦¬¬,øöõsVIÚ[ÊápDRVUeIÍyYÃlÄ^ÝUE¾×4ÊÍ[Öùùym_ýë¬2V-ijjHoUU¬ooo×Vvww«ÃÚ¤6dYÕäXÛèÉÍYóòòäjòMÃ§7Â×Y#¬5Ê< ß¨~kp[[[||¼ÊÔtÌ)KJJÔSe¬f´ËÕëõj­zBeÿ¨÷íÉä¹sçV4gd-¦¬RÄÔÔT-3.KÛV¬Þ¢jrj¨^¸ô	mVïèrÿù.Õ0eUo±øiPVà±Ê!ÞÂUÅXÍY;;;WTÖÜÜ'O¼¬÷ýoÌÕn*ÊüÄÄDeU%NIIááPV`Ê:??¼SÀþJaÊÚØØ°rvvv×®]!¦¤ÊðÊ®öc´´´ttt¨l$÷E½p DµcsÈ£',«ÛíõÛ·oç±PVà¤1III;wîÔväLcccO>­ß¯GI+«×ëÙaAAzSMäß:äA!§¶ÉÉI¹ô¹çü>:Îï¼RyyyVVVÈÿÈÊÂÂB-e"òM5=^·l¤>xaöª¢?³-[jjjB^T[[áTe²Êe²Êe²Êe²@Ye²@Y@°ÿ«ñê!DÚ½IEND®B`


KM time BY surgery
  /STATUS=status(1)
  /PRINT TABLE MEAN
  /PLOT SURVIVAL
  /TEST LOGRANK
  /COMPARE OVERALL POOLED.


Kaplan-Meier


附注	
创建的输出	19-JUN-2020 20:47:11	
注释		
输入	数据	E:\医学\肿瘤内科\孙佳春老师的肠癌统计数据\modified\data_modified 赋值.sav	
	活动的数据集	数据集1	
	过滤器	<none>	
	权重	<none>	
	拆分文件	<none>	
	工作数据文件中的 N 行	55	
缺失值处理	缺失的定义	用户自定义缺失值被视为缺失。	
	使用的个案	对于分析中的所有变量而言，统计量以带有有效数据的所有个案为基础。	
语法	KM time BY surgery
  /STATUS=status(1)
  /PRINT TABLE MEAN
  /PLOT SURVIVAL
  /TEST LOGRANK
  /COMPARE OVERALL POOLED.	
资源	处理器时间	00:00:00.25	
	已用时间	00:00:00.28	


[数据集1] E:\医学\肿瘤内科\孙佳春老师的肠癌统计数据\modified\data_modified 赋值.sav


个案处理摘要	
是否接受了手术治疗	总数	事件数	删失	
			N	百分比	
接受过手术治疗	21	21	0	0.0%	
未接受手术治疗	34	28	6	17.6%	
整体	55	49	6	10.9%	


生存表	
是否接受了手术治疗	时间	状态	此时生存的累积比例	累积事件数	剩余个案数	
			估计	标准误			
接受过手术治疗	1	2.000	死亡	.952	.046	1	20	
	2	6.000	死亡	.	.	2	19	
	3	6.000	死亡	.857	.076	3	18	
	4	8.000	死亡	.810	.086	4	17	
	5	9.000	死亡	.762	.093	5	16	
	6	10.000	死亡	.714	.099	6	15	
	7	12.000	死亡	.	.	7	14	
	8	12.000	死亡	.619	.106	8	13	
	9	15.000	死亡	.571	.108	9	12	
	10	20.000	死亡	.	.	10	11	
	11	20.000	死亡	.	.	11	10	
	12	20.000	死亡	.429	.108	12	9	
	13	21.000	死亡	.381	.106	13	8	
	14	22.000	死亡	.333	.103	14	7	
	15	24.000	死亡	.286	.099	15	6	
	16	26.000	死亡	.238	.093	16	5	
	17	29.000	死亡	.190	.086	17	4	
	18	39.000	死亡	.143	.076	18	3	
	19	57.000	死亡	.095	.064	19	2	
	20	58.000	死亡	.048	.046	20	1	
	21	87.000	死亡	.000	.000	21	0	
未接受手术治疗	1	1.000	死亡	.	.	1	33	
	2	1.000	死亡	.941	.040	2	32	
	3	1.000	删失	.	.	2	31	
	4	3.000	死亡	.	.	3	30	
	5	3.000	死亡	.	.	4	29	
	6	3.000	死亡	.850	.062	5	28	
	7	3.000	删失	.	.	5	27	
	8	4.000	死亡	.	.	6	26	
	9	4.000	死亡	.787	.072	7	25	
	10	4.000	删失	.	.	7	24	
	11	5.000	死亡	.754	.076	8	23	
	12	6.000	死亡	.	.	9	22	
	13	6.000	死亡	.689	.082	10	21	
	14	6.000	删失	.	.	10	20	
	15	7.000	死亡	.	.	11	19	
	16	7.000	死亡	.620	.087	12	18	
	17	8.000	死亡	.	.	13	17	
	18	8.000	死亡	.551	.090	14	16	
	19	9.000	死亡	.517	.091	15	15	
	20	11.000	死亡	.482	.091	16	14	
	21	13.000	死亡	.448	.091	17	13	
	22	14.000	死亡	.413	.090	18	12	
	23	14.000	删失	.	.	18	11	
	24	15.000	死亡	.	.	19	10	
	25	15.000	死亡	.	.	20	9	
	26	15.000	死亡	.301	.086	21	8	
	27	16.000	死亡	.263	.083	22	7	
	28	23.000	死亡	.	.	23	6	
	29	23.000	死亡	.	.	24	5	
	30	23.000	死亡	.150	.068	25	4	
	31	23.000	删失	.	.	25	3	
	32	24.000	死亡	.100	.061	26	2	
	33	31.000	死亡	.050	.047	27	1	
	34	36.000	死亡	.000	.000	28	0	


生存表的均值和中位数	
是否接受了手术治疗	均值a	中位数	
	估计	标准误	95% 置信区间	估计	标准误	95% 置信区间	
			下限	上限			下限	
接受过手术治疗	23.952	4.553	15.028	32.876	20.000	3.780	12.592	
未接受手术治疗	13.235	1.820	9.667	16.803	11.000	4.405	2.366	
整体	17.983	2.422	13.235	22.731	14.000	1.724	10.620	

生存表的均值和中位数	
是否接受了手术治疗	中位数a	
	95% 置信区间	
	上限	
接受过手术治疗	27.408	
未接受手术治疗	19.634	
整体	17.380	

a. 如果估计值已删失，那么它将限制为最长的生存时间。	


整体比较	
	卡方	df	Sig.	
Log Rank (Mantel-Cox)	4.281	1	.039	

为 是否接受了手术治疗 的不同水平检验生存分布等同性。	

£ìÖÇÇtç»¼ä².ÙÿøaªûÆ×²dÌ^òÊÊJYW·ëÎ+EoñPVPVÊJYÊÊJY)+@YAY)+e@YAY)+e(+(+e¥¬ee¥¬e¥¬u­ÛyóðþäOÔ×Oðÿ|êó»Þûào´ñPVPÖh6xoHcÖUÞô7~ýVNßõñ`=EòûNú^§¬ee¥¬ ¬ ¬² ¬²RVÊPVPVPV²²RVÊPVPVÊJYPVPV,­¬©î¯½µÐô­¶×¾û6ÿeeEDe½ÿ3o×0SËÀÁø(+(+"*ë¢X]PVPVPV²²RVÊPVPVÊJYPVPVÊJYÊÊJY)+@YAY)+e@Y)+e¥¬ ¬ ¬ ¬ee¥¬ ¬ ¬Ë×ÙÙÜÚÚj¿ÈëõîÚµKíØ±CW£¬ ¬ ¬s¹)++KOO·_ôüóÏwtth¦ªªjÛ¶m²uqñññsssIJJZèj[¶lÑé«¯¾úöû¿ÿûÔòß²õKêbÈy;Ï©·Þzë¿=¬¸¸xóæÍü·¤¬eeýRLL5|	Ë599ÉÚ`ÊPVPÖÅ%$$ÌÌÌµÁw221::Ê¶Ák³¬©îááººÓàÐ]þp ¬ ¬p»ÝÑ©Æ¦öÚÚÚ8 vùqÊúË:<rOcÖWÞz<*~­úþp ¬ ¬ðx<[·nILLôz½_Þù'YRRÒ&ÊºÖÊú)«õ£¬ ¬ ¬²RV²RVÊJY)+@YAYAYÊÊJY)+eee¥¬²²RV²²RVÊPVPVÊJY)+@Y)+ezÿóãÿøÖQV²²RÖÕQ1P³÷§.ÊPVPVÊJYÊÊJY×wñ^é¾úî_ì¼yø¤ïu3µâ¥¬ee¥¬Ë/ë×o½jU3ßëûï ¬ ¬uùìÛÖRV²²RÖUÖT÷Çv õG4­÷ ¬²F§îÑÞÿòþ_­£²Þùä®·ëÎzÞ(»UXâåéÊJY)k[eõ-+ÿ ¬²RVPVPVPVPVÊÊÊJY)+eee¥¬²²²RVÊJYAYAY)+eee¥¬²²²²²RVPVPVÊJY)+@Y)+e¥¬ ¬ ¬ ¬ ¬²>¾²æ¿é£»þÕîvUÊÊÊºqýàöwÞ<üâÛ/¯Ê¤RW¥¬ ¬ ¬X+eSÖ¿Ñ S3Ýåß²RVPÖåëîþö¥w4lÕê¾¡¸òoÊJY)+(ëêP+(+e¥¬ ¬²²²RVÊJYAYAY)+(+eeee¥¬ ¬ ¬²RVPVPVÊJYAYAY)+ee¥¬ ¬ ¬ ¬²RVÊÊÊJYAY)+(+(+(+eee¥¬²²²RVÊÊÊJY)+VnçÍÃGßùæIßëKÔã7ý²Â©´×3ì[ê¤¸~X±¡þ¡¾ó½T÷¯eÿiÓgþ£N)+(+eÅÕVÖÀÝû7y»î(®²²bu(®YAY)+(+(+e¥¬²²²²RVPVÊJY)+(+(+eee¥¬ ¬ ¬ ¬²RV²²RVPVPVÊJY)+(+(+ee¥¬²²RVPVPVÊJY)+(+(+eÅZ/kgggbbblllrrrkkkQVÊÊJYAYCs¹)++KOOð"ÊJYAY)+(khñññsssIJJÑßýÝßëa/¿üòæÍy¡¬ ¬õK±±±!çC^tùòåKMMÕE¼ÊDÖ!Ïï¼Å¿"¸ÿZõ5k>...ÂX PVÊºfffÌ*_ÍGxe@YAYCs»ÝÑ©Ëåð"Ê²²æñx¶nÝèõz¿¼+óO²QVõ¢¬(+(+eÊJY)+PVPV ¬ ¬ee¥¬@YAY²²RV²e¥¬(+(+PVPVÊ²RVÊJY²²ee ¬qqqw`=£¬uÓ5n¹bbbbccã¢]â·~ÿp7oÊöÔ¼eÿøW¾ò"DY£Ä¶mÛ¾ûÝïFåC«¨¨øßùhýÃýîïþî÷¿ÿý¨|h.þùç£ò¡ÍÌÌhdÖØØ.''gß¾²RVÊJY)+(+e¥¬²RVPVÊJY)+e¥¬ ¬²RVÊJY)+(+e¥¬²RVPÖ'H¯Î·nÝÊÖÛÛû7ó7Ñú+--ýÙÏ~ÍçóGåCûõ¯éÒ%¿ß®­­­ºº²@Y ¬kPgggbbblllrrrkkk<"¯×»k×.=¢;vèÑEåclnn¶v5nzz:###..îÙgmkk²?ÑöíÛÍcñx<ÑñèîÝ»æÅ$úþë²FÄårUVVj¦¬¬,===ÑóÏ?ßÑÑ¡ªªªmÛ¶EßcÕ[«¬Qóè.077§=÷ÜsQöÒNMÖû£Óû½W°ïæ7øEßË(k¤ÿáõZö`~EûÛÏè°eËèçÏ/..¶^Ñ¢æÑiXÓ××­ON½WÑNÍûõþè:400`/kð#îPÖÅÆÆ>/333Ê£F<)))zµ²^Ñ¢æÑéÎèÍÂÓÝÝe¸ÎÎN=4ýÕtªgfÔ<:YQ¿¼²cÍÇÅÅEÍãp¹QöÓÒÒÚÛÛí¯hQóèô@Ì×lúûûõî!Êþp»wï6#r%vÏ=Qóèìe~DÑúòÊºó«k4jdd$##ctt4úã¦EÓ£³ßy3¾¦?è-:½¬Á(*_^@Yçv»+**Ìï«H¼(xDmmm¸wï^?Fû+ZÔ<ºÓ§O=ôöö¾ðÂQöÓ8UK3===¿FÍ£³5øEå=PÖÅy<­[·ÆÄÄ$&&z½Þ(xDIIIQ]ô=Fû+ZÔ<ºñññ´´4çRRR¢ìwûömUN§Gg/kð#Êÿz ¬PV(+ ¬PV(+PV(+PV(+PV¬³³³SSSf¾ªªÊ~ ðzzzteý¡uS?é7my/-/,,|´ÿß6mJMMmhh8þ×Â'Oò|(+°R---JIIÉùÃáÉB×ìêêJgR¤.êóòò¬8UVVhëoÙ²eïÞ½Z^SS£åCziÉåËËàà .ÒïjooOçè£åaøýþo´pß¾Á<¼ÐüË ¬ÀpJ WíeÕpÖ~Í¹yfþÞ½úÁâââð·l'>99©ùºº:Ç¥W®nÐºZoo¯«åþÚ/µ~*ü/µ466êÊõèÑ£Z®ºó<(+¥KåHHHhWPP`Z9¬åÛ·o_´Ùö²j¬égÝxYYNÍõgfftVÇÄÄìÞ½»vîÞ¡CÂt<®yîÜ¹î¯ÎÎÎ¾r__+ÊDJ+--µÎ*~Vçj<wd=Nyyyµ¼¼Ü>5cVûÍÚËj_«¬ù;vD^Vs;Çü+~Ç¬FÈOPV tcTÄ%ÕsO81;;k/¥ù0µ¨¨È14Ô óúõë¹wïý£ÖEËºÿ~ë÷î¼¬úºÚÀÀ@Gm#)«¹499'@YE²êøÌÒQV¥.ø§4*Õ677[KzµäâÅÁ·o³*ufk)ÇIYMM&Ýnwäe³ycV)((|«i·¬SSSÖ¿öÏY5oZR]]íHoii©755YÛÚÚÌÊakP²¬fplm=ôèÆ¬§OÖÕôKÃ§7ÂÏY#¬5Ê|É¾R7üÚàÆÆÆÕt÷<)ÌS5oF´===uhhÈjÕ#*«ÞïíÉäùóç4fd-pSVqÏ=Vf|>µ®Ø|5ÅÔ4äÐÐ|ÛÛÛë¸ô­6ßÒHt¡w!¿¥¦¬æ+¶²+*«#Bö¼)«±³¶´´,©¬YYYÖ5Ï9yYÌ1×º)ùÑÑÑËjJÂÓ ¬À*ujj*x;&ÇöJaÊzýúuÇÂÝ»wÜ)«ã]ënÔ××777l$Å|ìØQ¢Ù°9äÞCµ³³SË·mÛÆs ¬À"ÔíÛ·ïÚµËÚG3nÙ²%??ß¾]8vÆduhhH£Ãó¥ÈuÈBm-@@>óÌ3?F¯×ëø-Á)uww<y2äû-ÌÍÍåÙPV "!¿TÑóuÓ& íÁ³Uíúo¶iÓÕ«WC^tíÚµÂ(+PV(+PV(+PV(+Ê(+Êý?>¬'>Ê=IEND®B`


KM time BY chemotherapy
  /STATUS=status(1)
  /PRINT TABLE MEAN
  /PLOT SURVIVAL
  /TEST LOGRANK
  /COMPARE OVERALL POOLED.


Kaplan-Meier


附注	
创建的输出	19-JUN-2020 20:47:49	
注释		
输入	数据	E:\医学\肿瘤内科\孙佳春老师的肠癌统计数据\modified\data_modified 赋值.sav	
	活动的数据集	数据集1	
	过滤器	<none>	
	权重	<none>	
	拆分文件	<none>	
	工作数据文件中的 N 行	55	
缺失值处理	缺失的定义	用户自定义缺失值被视为缺失。	
	使用的个案	对于分析中的所有变量而言，统计量以带有有效数据的所有个案为基础。	
语法	KM time BY chemotherapy
  /STATUS=status(1)
  /PRINT TABLE MEAN
  /PLOT SURVIVAL
  /TEST LOGRANK
  /COMPARE OVERALL POOLED.	
资源	处理器时间	00:00:00.25	
	已用时间	00:00:00.25	


[数据集1] E:\医学\肿瘤内科\孙佳春老师的肠癌统计数据\modified\data_modified 赋值.sav


个案处理摘要	
是否接受了化疗	总数	事件数	删失	
			N	百分比	
接受过化学治疗	30	26	4	13.3%	
未接受化学治疗	25	23	2	8.0%	
整体	55	49	6	10.9%	


生存表	
是否接受了化疗	时间	状态	此时生存的累积比例	累积事件数	剩余个案数	
			估计	标准误			
接受过化学治疗	1	1.000	删失	.	.	0	29	
	2	3.000	死亡	.966	.034	1	28	
	3	3.000	删失	.	.	1	27	
	4	4.000	死亡	.930	.048	2	26	
	5	6.000	死亡	.894	.058	3	25	
	6	7.000	死亡	.858	.066	4	24	
	7	8.000	死亡	.	.	5	23	
	8	8.000	死亡	.787	.077	6	22	
	9	9.000	死亡	.	.	7	21	
	10	9.000	死亡	.715	.085	8	20	
	11	10.000	死亡	.679	.088	9	19	
	12	11.000	死亡	.644	.090	10	18	
	13	12.000	死亡	.608	.092	11	17	
	14	13.000	死亡	.572	.093	12	16	
	15	14.000	死亡	.536	.094	13	15	
	16	14.000	删失	.	.	13	14	
	17	15.000	死亡	.	.	14	13	
	18	15.000	死亡	.	.	15	12	
	19	15.000	死亡	.421	.095	16	11	
	20	16.000	死亡	.383	.093	17	10	
	21	20.000	死亡	.	.	18	9	
	22	20.000	死亡	.307	.089	19	8	
	23	21.000	死亡	.268	.086	20	7	
	24	22.000	死亡	.230	.082	21	6	
	25	23.000	死亡	.192	.076	22	5	
	26	23.000	删失	.	.	22	4	
	27	36.000	死亡	.144	.071	23	3	
	28	57.000	死亡	.096	.061	24	2	
	29	58.000	死亡	.048	.046	25	1	
	30	87.000	死亡	.000	.000	26	0	
未接受化学治疗	1	1.000	死亡	.	.	1	24	
	2	1.000	死亡	.920	.054	2	23	
	3	2.000	死亡	.880	.065	3	22	
	4	3.000	死亡	.	.	4	21	
	5	3.000	死亡	.800	.080	5	20	
	6	4.000	死亡	.760	.085	6	19	
	7	4.000	删失	.	.	6	18	
	8	5.000	死亡	.718	.091	7	17	
	9	6.000	死亡	.	.	8	16	
	10	6.000	死亡	.	.	9	15	
	11	6.000	死亡	.591	.100	10	14	
	12	6.000	删失	.	.	10	13	
	13	7.000	死亡	.546	.102	11	12	
	14	8.000	死亡	.500	.103	12	11	
	15	12.000	死亡	.455	.103	13	10	
	16	15.000	死亡	.409	.102	14	9	
	17	20.000	死亡	.364	.101	15	8	
	18	23.000	死亡	.	.	16	7	
	19	23.000	死亡	.273	.094	17	6	
	20	24.000	死亡	.	.	18	5	
	21	24.000	死亡	.182	.082	19	4	
	22	26.000	死亡	.136	.073	20	3	
	23	29.000	死亡	.091	.061	21	2	
	24	31.000	死亡	.045	.044	22	1	
	25	39.000	死亡	.000	.000	23	0	


生存表的均值和中位数	
是否接受了化疗	均值a	中位数	
	估计	标准误	95% 置信区间	估计	标准误	95% 置信区间	
			下限	上限			下限	
接受过化学治疗	21.870	4.142	13.751	29.988	15.000	1.255	12.540	
未接受化学治疗	14.308	2.359	9.684	18.932	12.000	5.110	1.984	
整体	17.983	2.422	13.235	22.731	14.000	1.724	10.620	

生存表的均值和中位数	
是否接受了化疗	中位数a	
	95% 置信区间	
	上限	
接受过化学治疗	17.460	
未接受化学治疗	22.016	
整体	17.380	

a. 如果估计值已删失，那么它将限制为最长的生存时间。	


整体比较	
	卡方	df	Sig.	
Log Rank (Mantel-Cox)	1.351	1	.245	

为 是否接受了化疗 的不同水平检验生存分布等同性。	

>~ñññúPV5àPO1³g¬¿¿_©KKKKNNVN:eíÆA233÷påÊûVÄYoo¯¦«jkË[ÅÏl¥;ôx<ª»®¸¸X?Wmvl¯«¨ÅVÔìàÛ"i¨jí¶Âêº³7BÞÐeÕ?å¶mÛ¾ªô»1ëîõÒ_.øøãî÷öÛoGEEQV|m0(ëo<xP|UEGGuúú;Åjõ÷Î&?=öØ²_ÍGÿNA~úAëü·@Y±Î>gøªtLþcÖóÅ7ëùo²"ÊaM+äsVÊ²"|Êc6­Ö¹¦)+ÊÊº¢²º³Î;î*u×7ÿ² ¬Ï²n·ëÖ­±±±í×cËºãú!Ê²"LÊºlee¥¬@Y)+eÊÊºò²þÙÿáßëÿí¬¯²æååY»­/++Ó[eÀÅ´Ûí®®®NKKKLL´nÒÓÓãØù~mm­ÏçsÜÒM¦§§Í±]tÏSSSfC],--µ®ooo·.fdd9z¹ÿ(kÕõrÓ×®üÿöyY½Úòò·tUÕ÷?°/lvm¯@655åççk¢¸¸ØTÍ©ªªº÷®688xëÖ-û[«ã9özYXXhî-++Ëÿë>ÍquvíÚ¥s¥ÑqcÇuttLLL¨Ùª»æ8P­#®8pÀLèÝ3e¥¬°Ò²&º®uö<]*ýñù¿Ç,6<<¬ü<xPUY[ZZFFFº»»;»×@V)U¢ÌArÔH¯×k_ ¾¾¾¯¯Ï[NNNAAÆ¦Öð×eZeU­újB·RMu®*kÈ«kZçÊ§wuuõööê1BÏ/|ÇÒ@]ÝCee¥#çY×qYÐúA]s¯Nz)>þÅU§²ÿÕÉÿ°¬]õ@q5Ó&ZÊ¦]½zUíT5­«é¢r¥¥§§kæáÃM8u®ÂÍÎÎ*u&çÐÐ¹[GäÔ¿/~ñæÞLYì;¸5^AË:uJ?«µµõøñã%%%ú¹;wî4O!--M?´¹¹¹ÉFs4È¦¬ë²¬¾á;_|S'³S~â`8Ö~Y­e¿IêøÐôÈ#°ÚUTT¤abnn®§àiùcÇa¥®ò/ëåË­c©ª©©©ú)Öº^í?BchûÇ½ª¸²ªÅôHìÀ³VJk¦ÆÙIOuz¥¥¥öÞQÖu¿m0e°öË:66VVV¶wï^3ÚS233MÛ4ÕOÅòx<ª©A¤nÒÛÛôèQÇ2	sOYµ¼Þ­²j´ºk×.ÿ¤ÚsÞÒÒRUUem!eÂÙÑÑa­bF«¢pöõõéZUY%¾xñ¢Uk==`ÖSVx²÷Îg¿±Ø)HY[ÞþÙ¼rÝL+?VöN:5¿p,tóyª®ÕUê±ÿv¿öþyáóùÔ<ÇLøÚG«û÷ïohhÐÎ].Ëe¬ÏYM¡Uk-¦»µò¬«ìa¦¬ë¸¬¾á³ÏJY<Ô²þjzöÑ©ÅN£÷~äÍprêÿùgohh¨°°ðêÕ«Ê¤ÿÒd822Òÿë4²jø«Û76?âîû¶Án·Û.ikcc³ÂÙQVûæÁ&½5ÜÊjGY<ÂµÁ¡Ó8Ïþ!èéÓ§Ï9£9O(ÛÚÚKJJ4¦Ô¸S×Î.0«^Ï;§ø!£Æ ãÃZ«ö²:Vù:h¬|âÄ	SVý,Ó×'OZeUÎ5øÖþ&èëë+(( ¬MYëëëÏ=«áccccii©YÓ«bÂ´´´h¾5¾Tÿª««].B;<<¬Ä¯ÁhÈ«ûQÒ?®¦¤¤dffZÇªÖÖÍÕæ»wï¶¯4VóóóÓÓÓý¤FÀæTPkÐ[k¾É£÷vëáOvÞ	e¥¬ðy² ¬²RV ¬ ¬ÀF+kJJJÀV¸Ë~k#&öÈOY)+õZÖÛãw~|¯k±ÓÏ¦>¶/ltß¾:¯¯¯?ú´ÿ×r½ËþyöÈOY)+õ^V½GïøjÀ®zåöyÇoß±¬Ö.ûÙ#?e]²z:>ò?üÉï>².vÕ÷~øÝ¯ò/ëÊwÙ?Ïù)ëj5õæòn8NÊ­µûCxäeU4555fÏÖsÙ»ìgüuµÊÇÂ°Æ¬V&5<zô¨f_xå»ìgü²XeõüâÇßèûÎb§àeMzûË5ÒÒÒIÍ¿Z»ìgü5¤²æ¾I§;þêÛw¿GY¬©²þã?w±wÂM¬Û:¶®««ÓJfVÞ¹ò]ö³G~Êº´º¾ñZ_NiÞWWÊ`M­Ñôôtkk«Y§jÕLcG¥t~5vÙ?Ïù)ëQr¥¬ÖcYRSSýg®pýf&ä§¬ÀF,+(+e@YAY)+eððËÚóá=BEY)+¬ZY]×e¥¬ðhÊºÂCÙôôô8öÊT[[ëÿÝµs¬ÞÞÞ¦¦&=ÈMXßß=ú´cáÅÿCY)+Êº¨ÊFo­GµßaEEcçøkêX7fÏ%%%ccc555^¯·­­ÍúrI»i|Àï×RÖ5TÖ¿*ºYZuûÃþú>åÀ£-ëjÊ¦¾¾¾¯¯Ï[NNNAAÆ¦ÖðwMëÆ¿¬­­­úqö²8qBÐ¾/åÌÌLGË)ë(ëßó½Ü_Èz]'3`%eç½ïõ,vÒ;Lku[ó®¸òCÙ(u&§Ù³I=rkíX7KU÷iÙ³®õ²ÚQV¼¬ÖrÙ²Qð´¼ÙkºÊ¿¬kêX7Ê¿þ2¨««ÓóRUu]?Î*«n«§q³ýhðæø?zöNY)+Ö;­üP6]JJ£¬kíX7úAzxfè¼ØÕÊ§ÏçËÎÎvÿ²RVuQ«u(Ç*Sÿ<í±n6ý¹h¶¶UX(ØöÏYeUw®f?Ô¡*e¥¬Â§¬öì­äP6ö²ªRj°ãáù5v¬üü|SÖÜÜ=÷ááaGY5>6jèZûqà)ëC/kÝOßØqýuÒEÊ`uå²ÑÅsçÎ)~fÈ¨1¨"çø°víëFótGñx<###*«Èôô´ýGëÏû÷n[ZZ8ÐÝÝMY?²Úiðª!,eðÈËúÞWx(%Ö|F;Ývüøqu4%%%33Óú8víëF77ÛLé'fggkyÍohh0ëÀõþ0_fµóz½©©©þß¥¬À(+Ö#ÊJYPVPVÊ`c5åæK²RVXµ²î¸~hoí~»ì·6bbü²Øeµ¾Kjö7T__¯Æø-gÙ»ìgü²ØPeµ¾!ã_ÕÚe?ä§¬SY­Sê+Í¼Yx ²NýúWÿjd±ÊäÚÿ3û¾Iúuå»ìgüõs+«ÅÓñÑKy7x³ð@eýFßw½õ¥ÅN*kkÿ¡ïÛVB´ö>oßëÐJvÙ?Ïù)+e°¡Ö[Tf=êØûüÊwÙÏù)+e°±ÊjÔ,HKKS&­meçWoýì²RV¨¬jU]]&T2³òVìÈï²=òSVÊ`CuzzºµµÕ¬SµÊjJ¦±£2¿»ìgü²Xïeåöùe¼I¦¦¦úÏ.ûÍLöÈOY)+õ]V¬G² ¬ ¬ee¥¬@Y)+e¥¬(+(+e@YAY)+ÊÊJY)+ÊÊJYPVPVÊ²²RV ¬²RVë±¬>/666222>>Þ~AimmÝ¶m¹Ê¾kfÊ²².*99¹¢¢BÅÅÅ)))ö«¢££æGYPVPÖ¥)æ~333|>õÔSæP:×´&rss¾ækPûð^îYïý7?üOê«N/wüe@Y±ÖËª.6+·lÙ¢Î½^¯æ466þõýÎ9³yóæ÷r÷|[cVsÚqýe@Y±ÖËaMGEEÙ¯Úµk9Ú»»÷îG²6Ø² ¬Xe1k5âp² ¬ ¬¹òòrMè<99Ù~Æ©]]]èììÔø² ¬ ¬Ks»Ý[·nõx<=ÙÝ»wTVu®éõUÖD×µ²^×I?lïãe¥¬ëÃÚ,ë½¿ü°Ä²Ï·Õ5÷òÆ²RVÊºü²Ú/¾IYPVÊJY)+ÊÊJYPVPVÊ²RVÊ²²RV² ¬ ¬² ¬ ¬hüèTÿÓÜ|² ¬ ¬«3f­¯!÷ÝKee¥¬(+(+e@YAYÃ²¬7þcÊ²²®>Ê²²RV² ¬ ¬² ¬ ¬ee¥¬(+(+eÊJY)+e@YAY)+ÊÊJYPVPÖZÖ×Ý)7§ÎOº)+ÊÊº|]ôXYUbÚ õÒ[Ï©^Õé/þ¶w²RÖ`rß½¼¬·º5f5§D×5ÞAPVÊJYWTV;Ê²RVÊJYPVPVÊ²²RV²RV² ¬ ¬ee¥¬e¥¬² ¬ ¬ee¥¬@Y)+e]qYK«nÓZ?àÝe¥¬uEe-¯yÏdõ¥¼/d½Î»	ÊJY)ëÊjñt|¤¸òn²RVÊJYPVPVÊ²²RV²RV² ¬ ¬ee¥¬e¥¬² ¬ ¬ee¥¬(+(+e]AY]×êÍ©àSÞYÊÊJY_ÖûG¾zé­óÅ7u2å ¬ ¬uùeµS+@Ye¥¬ee¥¬(+(+e@YAY)+e@YAY)+ÊÊJYPVPVÊJYPVÊäf6íÛ·/ÕO½®ÊÏÏ§¬e¥¬VÖ¤¤$3ÝÞÞ®ÝÝÝÖU¥¥¥² ¬õÁÊ*õ.^¼¨é´´4kÌÚÐÐ@Y)+ÊJY)ëçWÖ³ÅÕÓñ9Ý»÷KÞeÊ^ÖMÌÍÍÙ×KMMÍää$e®¼æ½ÔW_Ê»¡S¢ëÚý#¼ËÛ`5µ²²222òÔ©S333###öO[óù|±±±ºm|||KKýªéééôôô¨¨¨'|²µµ5j÷BÖë ¬Øèe­­­5+/_¾¬éÌÌLM9sF¹åæÉÉÉ(..NII±_wñâEÝ²úÔSOiÎw¿ûÝÓ÷ËÊÊÚ¼yó#üÿpüG§ÿá'W)+ÊU+ëøøøÄÄÆJãÖ­[ÌÌ¿rm¬Án\ý*bßóçÏÿëû%$$h¼ûÿ?üwú_ÜþÊ²bÕÊj¶Z=+++5¸Ü»w¯¯º¨.+øÍÕÅÓæbaaá-[t·nÝZk)+ÊU.«ªzYx<³A×ëu»Ý¶*ÕÕÕÁoaMGEE9®*++ÓDOOÆ¦kª¬ý£|Ú§ÓÝø/iÞWßþù;:Ýî¦¬(+Va¦Ó§Oëe¡Á«UÖééé'OjÚ±Q¿³6XÓ«Î>ò²&½õåçÞ|Q§×éÐrD'MPV+*ëðð°^«««3[3Õ××k¯^½ªUUUAîÁårkBçÉÉÉö«N8anÛÕÕõì³Ï®ým)+Êµ»»;::Úº811¡Ç¾ðû2W®r'n·ëÖ­±±±f²øÍ 4ZMHHèíí¥¬(+6ÊÚàG² ¬·²jHZSS£Ebbâ,ëöÝ¦¬(+VgmpSSùvÏçÓ»wï>þøãIIIÉLqÍ¾Ãµ¬veÅ*Y'''õ0;sl¬EEE5Dÿ!ãWFy»(+6PYçææâãã5*5¯´z½^···ÛËZYYIYCtô¿6*®¹&º®é·²bcYÍ÷PKKKõèïïw_)ëJhäúRÞÞnÊ¸6ØìÚÐ|ñ²RV+*«ÇãIOO7;ß×¹9~|¼¦¶¶6ÊJYPVÊêUáÔy__ùÕZ3l-àõz)+e@Y)ëÒ¢¢¢WÀ5ÀCCCûASVaRÖshGYÝn·.666Ä¦§§SVÊ²RÖ`ÊjRªÜjº¦¦Æ>~Ý½7e¥¬(+eÕøø¸ùµ³³S===ök»ººê+² ¬·²vwwë5ÑÛÛëv».PWW·qÊZt§Üê~úÆJîêÞþé¾ÒÂÛ@Y±Ëúh­©²ß©0YMó¾úÜ/®ä®¾S÷þáãßçí ¬ ¬º¬÷ Wq¥¬(+e]ÙÙÙ©©)3]YYù9¿>(+Êp+kss³^[@Y]Ö¿-'ÑuÍÿôíº÷yë(+6JYõØ²e®¨¨°UÃYÊº<ömÏß¬kîå­ ¬ØeÓ"&&¦~ÁÉ'£¢¢Î9ªfgý² ¬5TÕÕÕöWVVÆÆÆ²6² ¬_öù56¥¬«^ÖÑ_Ü»óÁÇ ¬ØXeÕK¡  À>ÇQVshtÊºB ¬Ø@eêèèHZ`ÿUÓ:·NY)+ÊJYC5;;;77ÇÚ`Ê²buÊdm0e¥¬(+e¥¬eÅ.ëÔÔÔCÝ² ¬²ÎÍÍmÛ¶mçÎõõõ©4qêÔ©-[¶äææZsÌÎ"ÞÎ(+Êð³~_ª¡¬ ¬ØÐk)«UÖ×)®¡óE-OYÊÊêÔÙÙ©Gee¥ãsÖXÖOÆGËOÏ¿õg¯õ5PV²²Î[û0©&''ëÅcÈZQQ¡9­¬$÷ÝK ¬D²~¶µÎgvm0e@Y±ÒµÁzYlÛ¶ÏY)+ÊU(kCC^íííØ¿rCY)+ÊJYl°^Ç³ï@x~áÐ­GY)+ÊJYF¥zM455Ysººº4'??µÁe¥¬¦²²²¨¨H¯ÆÆFkfkk«Y9lj)+e@Y)kH¯ÆÄÄÍTÓ]jjjòòò4'99YÓfDÛÙÙIY)+ÊJYØ½·µa°×ëµvv¨ÑêC­)e@YæÛë5qùòeë"e¥¬(+VZVûöJ² ¬X²TTTdggkGY)+ÊJYYÖºº:3=22¢YYYÏ¦¬(+Â°¬e@YVemoo?² ù~555z¡<y².é+ïþuÑò>í[ìû¦×ßã­ ¬ØeñßÄôô´ùkaa!e]Ò×Þ+ÞqýÐso¾¸ØI×þeó·yë(+6DY»ººìGFF|>^"---º8;;KYWî?ÖæRV²b£U¯òòòööö¨¨¨ùßì_cÖÎÎNMSVÊ²RÖPËZRRb&ªªªjjjöìÙÓÝÝ­ÙÙÙ]SVÊ²RÖ%ÊZVVf]4.íß¿ß|ø:99IY)+ÊJYYÖÍÉÍÍ5s(+e@Y)ëõÖ­[GÍÍÍ³8p@¦¯² ¬õËª¾µÁO<ñ&L²RV².gmðàà`wwwQQQee¥¹JAeÌJYPVÊú`UÓ«!66VCÕêêê£Gêbtt´.Ý0]½zUsTJYPVÊææfûEÇþÌÕçóQVÊ²RÖõ² ¬ ¬õÁyí«ÏÍ!ëu]×ú>åm ¬Û²=755ée199988hÖ	ëâ'(ëªøÊóË:¯Ø?¢Ós_ª×9oCeE8oÜ´@ããã:¯¯¯7;åw|KYÍ~W[)+@YÎeíéé1Ö¹Ëåêèè0¹öÙgYLYPVÊú`<F¨eeegÏ5Ç´Ñyee¥ÎþùúùùùÖ~$(ëòd½sö¹7_,ºS®Ó¿+Î»Øñ?Íô§º¾Á@Y±Êj>88¸i9Vk¿¦4ýøãoß¾1ë½ñÑ+¡5ÍûªzÌ@Y±ÎÖÏV? éóÅÖPVû|¾ØØØÈÈÈøøxs¼tó!îF.«]èkÝ^Å7,²b=uddD¯ÞÞ^¿	5RT]4ãWÍÌÌÌ~'ÉÉÉ(..NIIñßöxçÎÖk®¯¯ïæýô³¢¢¢(+e(+ÖYÕQki6_2­µ²ccc:¿|ùrðû6Ü¸¸8Çµ. ØzÍ¥§§oòóØcQVÊPVÃUeUPëëëõxâ'jkk5a¾ÒjvÄ¯sÇüN"##NËÀÀ@BBùv¬óË_þòãû½ÿþûY)+@Y&eÖ¹F¥«&JJJ"""ÌUÙÙÙz¡$%%-y'ÖMD´_¥·µµNó9+e(+6ÄLf@¹ûv¯×kvaæ×ÕÕY.&&fffÆ¬Öôè~²á_VÃáÜìÑP­5»ÈÉÉÑykkkðÛºòòrMè<999ð#cÌJYÊRVíòåË?þøøø¸.ÖÖÖÍ5166¶Ø=¸Ýî­[·jØØXëCYÇ²RV²"üË:33ÓÜÜ055eætww«fYÞÞheMt]îKõæ¸7ÁOôÄïeòPV¬ËµÁÐF+ëG?ûÔôfÉÓ×ÿíw¿Ì@YAY)ëêøÖÍ?¬¥¬ee¥¬ ¬ ¬² ¬ ¬ee¥¬ ¬ ¬õQuÇõCiÞW;ýçö??sûoù(+(+eÉþéß]uz;Ý)W(+(+e§ã£ònY4²RV²²RVÊ²RVÊJYPVPVPV²²RVÊ²RVÊJYPVPVÊJYÊÊJY)+@YAY)+e@YAY)+ÊÊJY)+@YAY)+e@Y)+e¥¬(+(+e¥¬ee¥¬ ¬ ¬² ¬ ¬²²RV²²RÖGYÖD×5ÅU§²^¯oé¥¬ee¥¬Ë7ú«9)®¥U·)+@YAY)ëêPV)+@YAY)+e(+(+e¥¬(+(+e@YAY)+e(+(+e¥¬(+e¥¬eee(+(+e¥¬ee¥¬ee¥¬uÇõCEwÊÍéæÏÌ?@YAY)ëòËÚ7úÏVVMbù(+(+eµ¬öãÞ:0å ¬ ¬5$ýZÇ½Qb?ì¡¬ee¥¬«CcVÊPVPVÊJYÊÊJY)+ÊJY)+e@YAY)+e(+(+e¥¬(+e¥¬eee(+(+e¥¬ee¥¬ee¥¬ ¬ ¬²²®®k©¯4¿wãK_i¹xåG ¬ ¬uEnuÞVÝV+@YAY)ëêPr¥¬ee¥¬ ¬ ¬² ¬²RV ¬ ¬²²RVk±¬>/666222>>¾¥¥Å~ÇãÙ¹s§®Ú¾»£¬ ¬ ¬KKNN®¨¨ÐDqqqJJýª§~º½½]Ï<óe¥¬ee]ZttôÜÜ&fffâââ[lË-:ùåÿÕýþàþ@Zþ[.ÃkwÿÙ÷)+@YneUNÛy½ÞMÜ¸qãîWPP°yófþ[.ÃwêÞ?|²aWÖk:**ÊäääÉÉIÖSV²².-&&fffÆ¬Ö´ãÚ¡¡¡ôôôááa¶^-?lï;_|S§¤Ì$º®¹^nÒéÐéüã«cæ?ªÓW/½¥ÇÆ/u¥Wyy¹&t®±©ýªÖÖÖýû÷«AnNYÔû?ù¸®¹W§¿üºGeýÚéô§W¾ùß^ÿfþ£:½õziÕm~A ¬ ¬+åv»·nÝëñx>(/²¸¸¸M6uu­µmUÊÊÊúèQÖÕ*kî»>ø´Ïqê ¬eeEHz?.¯yÏLûî÷vôÜ/ÚOfe(+(+VÐæ² ¬ ¬²RVPVPVÊJY)+@YAYAYÊÊJY)+@YAY)+e¥¬e¥¬¿ÕùIwÒ[_¦¬eeÅêøàÓ>öPVPVPV²²RVÊJYAYAY)+e¥¬eeÅ.ëùâöýiphW(+e¥¬õ¡íw]×^Èz=¼Oz_½ô¯.PVÊJY)+VG]s¯æü;²RVÊºAËºãú¡¢;åANß¾û=þ¡(+(+(+BòÉøHð¬æ¾Iéå²²²ÕÅ²RVÊÊÊJY)+(+(+e¥¬ ¬²²²RVÊJYÊJY)+(+eeee¥¬ ¬ ¬²RVPVPVÊJYAYAYAYAY)+(+(+e¥¬²RVPVPVÊJYAYW»¬®k¥U·Íéý|Ì¿	(+e¥¬ ¬Ëwç­¬*±-ÿ& ¬²â·eÝqýP÷UÇI¹ýÙ/~Î¿ÏÎß¤¬ ¬²â·îýòÐëRn]þ(+(+(+VÆ¬²²²²RVPVPVÊJY)+(+(+e¥¬ ¬ ¬²²RVPVPVPVÊÊÊJY)+eee¥¬ ¬²²²RVÊJYAYAY)+eee¥¬²²²²RVPVPVÊJY)+@Y)+eEPx#Å=èUý4to8þ¡Îz]×8­äDYAY)ëF³*®:_ÝÓëîóÏãÃþ²^§¬ ¬Ë§¬RVPVPVÊÊÊJY)+eee¥¬ ¬ ¬ ¬²²²RVÊJYAYAY)+(+(+(+ee¥¬ ¬ ¬ ¬²²²²RVPVÊJYAY)+(+eee¥¬²²²RV¬§²ú|¾ØØØÈÈÈøøø¯¢¬²²¡âââ¯¢¬²²=77§¸¸¸àWë[ßúòý^|ñÅÍ7ó.CYAY)+(ëg"##N¼êòåËî¨«xÙZÜo|t#Þ+­ºMYAY°¦£¢¢B¼µÁÂ	e¥¬«)&&fffÆ¬òÕtWQV50ËU^^®	'''xe@YAYs»Ý[·nõx<=YÀ«(+ÊÊúQV²e¥¬(+(+PVPVÊ²²RV ¬ ¬@YAY)+ÊÊJY²RVÊ(+(+e@Y)+e¥¬@YAY²²PÖ¨¨¨`=£¬uÓ5j¹""""##£Â]Tâ·~q7oË§öØeßüw÷weÏ<óÌ×¾öµ°|jååå¿÷¿®¿¸ßÿýßÿæ7¿OíâÅO?ýtX>µÌÂòÙeggïÝ»²RVÊJY)+ee¥¬²RVÊÊJY)+e¥¬²RVÊJY)+ee¥¬²RVÊÊúéÝùæÍaùÔººº¾þõ¯ë/®¨¨è½÷ÞË§æõzËÊÊÂò©ýú×¿¾téR___X>»ÖÖÖªª*BÊe²®A>/666222>>¾¥¥%ÇãÙ¹s§ÑöíÛõìÂò9655Y;g7==õäO¶¶¶Ù/NÏhÛ¶mæ¹¸Ýîðxv£££qqqAÞLÂï¿(kH+**4Q\Ïèé§noo×Deeå3Ï<~ÏqvvV:XegwñâÅ¹¹9Eè©§³_ôÀÀ&tnj´Þþ>ÐßöÝüú?£ðeõ?¼ÞËæ¶W´ÿù¶lÙ~ÏñÂÖ;ZØ<;kº»»ÃõÅ©¿4¡sówÃzvìííµÕÿ÷Û(ë¢"##N¯×fÏQ#½[YïhaóìôàõÇÂsëÖ­0ûÅù|>=5ýÖt®WfØ<;YýQ¿½²aMGEEÍóHNN³çÔÖÖfGg§'b¾fÓÓÓ£¿Âì·k×.3"WbwïÞ6ÏÎ^Vÿg®o/ ¬K_X]£éðxRCCCéééÃÃÃá÷7Ý/ýÁñM8ýâüGoáñììeõFaùöÊº4ËU^^>¿°¯"òÂàµ¶¶îß¿tt4£ý-lÝ'ÌºººöÙ0ûÅiªç¥ÎÎN_ÃæÙÙËêÿÂò¿(ëÒÜn÷Ö­[#""bcc=O<£¸¸8Ç¨.ü£ý-lÝøøxRRÆs			½½½aö»÷®ªg§sMÍ³³Õÿå=PV(+PV(+Ê(+Ê(+Ê(+ÖÙÙÙ©©)3]YYi?HpZX7±Ï´îjéý¦Mò¥ùgÏ¸ÿß6mJLL¬¯¯OýMkfZZ¯²+ÕÜÜ¬¨Î/O[²££#iIº¨æääXqª¨¨0Ñ2ËoÙ²eÏ=_]]­ùCziÎåËýJ¿®ÒÏjkkKYàè£åAôõõüCA3÷îÝë?óÀþ3Í¿Ê<ÀN	4ÓJ£½¬ÎÚ[`¦GGGuÃà÷l'>99©éÚÚZÇµW®îÐZ¬««KÅÕ|í×Z·þC-Z8###ô²>ÿüóííí¯ºó:(+¥Kå©_pòäÉ¨¨¨3gÎXÄÅVkþ¶mÛl¶½¬kºXw^\¬óØØX³üÌÌ.j«é]»vÕ,ÐÃ;xð`ð®ÛíÖçÎ[ìÿÚà¬¬,ÿ»»»YQPV TÊXQQuQñ³:·äpPã¹ÃìqÊÉÉY¬¬eeeö1¨³ÚïÖ^VûZeMoß¾=ô²û9zôhäë?f5~¢²£">PYÍ0÷Ø±c³³³öRSóòòCC:¯^½ªÑÑQûG­Kuß¾Ööì	½¬úZ¬··7È³¶Á¡ÕÏ ¬ÀYu|fé(«Rç+JuÃ¦¦&kNWWæäççûß¿ÌªÔ­¥Ünw(e5U6t¹5ÈJìåYåÌ3¡o5²bãujjÊÚâ×þ9«¦MKªªªé-**ÒüÆÆFkfkk«Y9ljÕ­­ÞõÄZL?4xzCü5ÄZ ¬Àgì+u¯nhh1QMw-Ð2//Ï|9UÓfDÛÙÙ¹XY¬V=¤²ê¯ó½ ¼páÂYCÙbeUEÜ½·¯×k­+6_M15844Ávuu9®HkÍ74]ì/ßRRVó[Ç£@YÕ!ÞÕÄØY¨¬Ö§N½¬ó_ÌµîÊÁd~xx8Ä²'$$ðò(+°eòßÉ±½R²^½zÕ1sbbb×®]÷¦dÊêød×zuuuMMMf Ês1;vh6l¸÷Äeõù|ÿÌ3ÏðÚ(+°5fÛ¶m;wî´6äÑ[¶lÉÍÍµo×#1YeÐè0;;Û|©&ôp C[ËÈÈ®â'BÇñSü7SºuëVAAAZZZÀ¿4óäÉ¼ZÊ$àjBz½nÚ¤¤=xA¶ª]CÿÍ6m*))	xUiiiCaÊ(+Ê(+Ê(+ÊeÊeþþ?µÇp¹îàIEND®B`


KM time BY chemotherapy
  /STRATA=surgery
  /STATUS=status(1)
  /PRINT TABLE MEAN
  /PLOT SURVIVAL
  /TEST LOGRANK
  /COMPARE OVERALL STRATA.


Kaplan-Meier


附注	
创建的输出	20-JUN-2020 13:09:08	
注释		
输入	数据	E:\医学\肿瘤内科\孙佳春老师的肠癌统计数据\modified\data_modified 赋值.sav	
	活动的数据集	数据集1	
	过滤器	<none>	
	权重	<none>	
	拆分文件	<none>	
	工作数据文件中的 N 行	55	
缺失值处理	缺失的定义	用户自定义缺失值被视为缺失。	
	使用的个案	对于分析中的所有变量而言，统计量以带有有效数据的所有个案为基础。	
语法	KM time BY chemotherapy
  /STRATA=surgery
  /STATUS=status(1)
  /PRINT TABLE MEAN
  /PLOT SURVIVAL
  /TEST LOGRANK
  /COMPARE OVERALL STRATA.	
资源	处理器时间	00:00:00.31	
	已用时间	00:00:00.65	


[数据集1] E:\医学\肿瘤内科\孙佳春老师的肠癌统计数据\modified\data_modified 赋值.sav


个案处理摘要	
是否接受了手术治疗	是否接受了化疗	总数	事件数	删失	
				N	百分比	
接受过手术治疗	接受过化学治疗	10	10	0	0.0%	
	未接受化学治疗	11	11	0	0.0%	
	整体	21	21	0	0.0%	
未接受手术治疗	接受过化学治疗	20	16	4	20.0%	
	未接受化学治疗	14	12	2	14.3%	
	整体	34	28	6	17.6%	
整体	整体	55	49	6	10.9%	


生存表	
是否接受了手术治疗	是否接受了化疗	时间	状态	此时生存的累积比例	累积事件数	剩余个案数	
				估计	标准误			
接受过手术治疗	接受过化学治疗	1	9.000	死亡	.900	.095	1	9	
		2	10.000	死亡	.800	.126	2	8	
		3	12.000	死亡	.700	.145	3	7	
		4	20.000	死亡	.	.	4	6	
		5	20.000	死亡	.500	.158	5	5	
		6	21.000	死亡	.400	.155	6	4	
		7	22.000	死亡	.300	.145	7	3	
		8	57.000	死亡	.200	.126	8	2	
		9	58.000	死亡	.100	.095	9	1	
		10	87.000	死亡	.000	.000	10	0	
	未接受化学治疗	1	2.000	死亡	.909	.087	1	10	
		2	6.000	死亡	.	.	2	9	
		3	6.000	死亡	.727	.134	3	8	
		4	8.000	死亡	.636	.145	4	7	
		5	12.000	死亡	.545	.150	5	6	
		6	15.000	死亡	.455	.150	6	5	
		7	20.000	死亡	.364	.145	7	4	
		8	24.000	死亡	.273	.134	8	3	
		9	26.000	死亡	.182	.116	9	2	
		10	29.000	死亡	.091	.087	10	1	
		11	39.000	死亡	.000	.000	11	0	
未接受手术治疗	接受过化学治疗	1	1.000	删失	.	.	0	19	
		2	3.000	死亡	.947	.051	1	18	
		3	3.000	删失	.	.	1	17	
		4	4.000	死亡	.892	.072	2	16	
		5	6.000	死亡	.836	.087	3	15	
		6	7.000	死亡	.780	.097	4	14	
		7	8.000	死亡	.	.	5	13	
		8	8.000	死亡	.669	.111	6	12	
		9	9.000	死亡	.613	.115	7	11	
		10	11.000	死亡	.557	.117	8	10	
		11	13.000	死亡	.502	.118	9	9	
		12	14.000	死亡	.446	.117	10	8	
		13	14.000	删失	.	.	10	7	
		14	15.000	死亡	.	.	11	6	
		15	15.000	死亡	.	.	12	5	
		16	15.000	死亡	.255	.107	13	4	
		17	16.000	死亡	.191	.097	14	3	
		18	23.000	死亡	.127	.083	15	2	
		19	23.000	删失	.	.	15	1	
		20	36.000	死亡	.000	.000	16	0	
	未接受化学治疗	1	1.000	死亡	.	.	1	13	
		2	1.000	死亡	.857	.094	2	12	
		3	3.000	死亡	.	.	3	11	
		4	3.000	死亡	.714	.121	4	10	
		5	4.000	死亡	.643	.128	5	9	
		6	4.000	删失	.	.	5	8	
		7	5.000	死亡	.563	.135	6	7	
		8	6.000	死亡	.482	.138	7	6	
		9	6.000	删失	.	.	7	5	
		10	7.000	死亡	.386	.140	8	4	
		11	23.000	死亡	.	.	9	3	
		12	23.000	死亡	.193	.119	10	2	
		13	24.000	死亡	.096	.091	11	1	
		14	31.000	死亡	.000	.000	12	0	


生存表的均值和中位数	
是否接受了手术治疗	是否接受了化疗	均值a	中位数	
		估计	标准误	95% 置信区间	估计	
				下限	上限		
接受过手术治疗	接受过化学治疗	31.600	8.331	15.271	47.929	20.000	
	未接受化学治疗	17.000	3.495	10.149	23.851	15.000	
	整体	23.952	4.553	15.028	32.876	20.000	
未接受手术治疗	接受过化学治疗	14.552	2.389	9.870	19.233	14.000	
	未接受化学治疗	12.155	3.172	5.939	18.372	6.000	
	整体	13.235	1.820	9.667	16.803	11.000	
整体	整体	17.983	2.422	13.235	22.731	14.000	

生存表的均值和中位数	
是否接受了手术治疗	是否接受了化疗	中位数a	
		标准误	95% 置信区间	
			下限	上限	
接受过手术治疗	接受过化学治疗	4.743	10.703	29.297	
	未接受化学治疗	6.606	2.053	27.947	
	整体	3.780	12.592	27.408	
未接受手术治疗	接受过化学治疗	3.154	7.817	20.183	
	未接受化学治疗	1.556	2.951	9.049	
	整体	4.405	2.366	19.634	
整体	整体	1.724	10.620	17.380	

a. 如果估计值已删失，那么它将限制为最长的生存时间。	


整体比较	
是否接受了手术治疗	卡方	df	Sig.	
接受过手术治疗	Log Rank (Mantel-Cox)	1.971	1	.160	
未接受手术治疗	Log Rank (Mantel-Cox)	.598	1	.439	

为 是否接受了化疗 的不同水平检验生存分布等同性。	


分层: surgery = 接受过手术治疗


úú?aüýj4zçwÞa><ï¼3³ov®üYYYYYYY>¯¿®û®^½º¥åsrrvüf¬¯¯Ûõß¾['u²­­Ms&&&øM!+Ñî­¯¯Ï)åÝ»w·$ë«¯¾ê9??ï<922r.7¤­¡®®.tµ'N0~¿¿¡¡AËËË:>pà¿2"d%Ú½-,,+ÇcI»ÿ~¤FÅòòr-òäI§wîÜÑLMÛK­®®ÐµMOOï6<<ì:keeEóuÜÕÕeÇ©¨®®æ·F¬D»7ÛW]²>yòÄ¹ðÚÚÝNkNKKÛÆ...666&&&ÎÌÌ;00 ¡ëëëÍööíÛÒT===fã³!¼°°P'§¦¦ø%!+Ñnús&¨ì¶Ù£Géææfsn¤ñ«ÎÊÎÎÝºûðáÃÐå50üø±½Ò3gÎl_7r-©1®üÖ0W£a-yéÒ%®ùIIIééévfs1!+Ñ.JCUóB¦Ú([mcccZòÎ;Æ<Û½÷4_Ç.¼Ï=+,¯j`Ú××§ÜÜÚÛÛëë¬ÖÖV×ÔÔèxttTÇvß ²íºkEEkN,²îI477×ØØh_¯u-¬Ñ§&:::Ìö[WVVêØìëdµWóÒ¯0ìîîîééÆ:ivñv²½¸Ì¸Óùº©K,c®Ì[_Ìæ#m7ÖüS§NmßKÓÜÜ<99©9vöövÍ1ÛÍ¾KÂÕknd5«Ò8vvV_"²í®«ÐTg¾Îj°]u^ª¿¿¿   ì`7ÒVV#¢ ÆE³=Ù%«êíí½pá½fÂ,ÙÔÔ$nÍÀì»D¬D»±@°°cÖÐ|>Ðò:rGæ3ø%%%µ»xñ¢NÚý~ÉåååÏ^g¥HIIqÉjö`6ÍÛX«ªªtï`ùÝ!+ÑÞÈFURC»¹.£Ykkk333s<x`©6ãW+«]Fè×²²ì|Mk.È/Yö¶¬®ÅÌNI1Êj¶Kâ9iÔðÔ5fµÍÍÍ?~ÜRí¹Ýµ?3!+Ñ^uvvÖõÖmËjª©©éîîv.VV³ù,'óÒ¬Ù,¬á¯ùì'Mðû"BV¢]TkkkØOñÍÊÊ²'#XSV-S__¯9/^½ó¢¬ý0°ªÌç=iù®®.ÝÂÂBçzÆÇÇA!þÍjWVVøU!+Ñ®èYLrmÉ£££aÖÓµ±«ÎÎN]<tdlâ¡!ÍþPD¬DDDÈJDD¬DDD¬DDDÈJDD¬DDD¬DDDÈJDD¬DDDÈJDDDÈJDD¬DDDÈJDDDÈJDD¬DDDÈJDQÿêöí;tèsÏçëììÜÒJÖ××jjj´¶W_Õuîììl¤vttè"Qp]Ëðð°¹ÄÄÄè¯®®j±Ë/ëRYZZÒ]3uÁ±±±ï»V¢åx,²Ñ/d=yòdèÌS§N]^ÃÖÖÖªªª[·nÝ¼y³¸¸XÎ¥¤¤ó;æúªó¶¶6Í5Lµ··ë¬Ç»æwwwëR---ZóíÛ·÷+,,|øðaSSÎ]\r¿ïëëÓ6·*ìbºñ:wffÆyß8°¥auuµ.599içDú²w"d%ÿäÖñãÇ5±²²L%''?^µµµ2£®®.t ©±æþþ~,--ë·Çã$ëÂÂ"×@Ó¬YÇ[Fë"åååÖtBBB¤¡íÈÈ±ÆëÐò:k||<Ùú¡yé§TVVfÆÇæd~~¾NNMMñè"d%z¹2BySRR"J:´¼¼¬á -¹¹¹®K¢ÎJOO×F¢^MÜ¿?ìuMLL<yòdttôl0k67CÒH75TÖùùùXãöö466JñH2xk1:Y__o°k6f!+Q¬éI¶°°ðW^1Û9Þ¸pÎ1Ó_üâí`N'322Ù8é·ÎÕ=zÔù§sU_þòÍ-qí4ªùføè¼Tha9qYM¨iaa,åª¹¹YÇGi	¦éG-¢EEEfÆma*tÌº5xllÌ¬Þ½W]¸pÁÔèP'«ªªìò½½½Y³ÍÍÐÔÎ1·P7ÛZkïæÏÍÍ;â¼æ5×÷îÙè¤æùÌ`^GæJ¯_¿Î_!+;=G»d²O±Èª ð:×søðaçòÌ,ls«ºråkÃ¬ÙT«ÿBoL,²¬@àX0cF®:¶TØÝd¹NÖÔÔDùÑé_-sðàA³¼l3+1s4rÕtWWWtY¥nR1kèòÎÈÖ9qâD¤hðmGÞ7nÜ0?¨k×®iZÄê`¶[¾ñÙv`scü~ÿÊÊk·nÝÒY>ä/ÈñÌ¼Þf^í^-±ÈzóæM=¹]Z¢QJ#K=[³Íò¯éàN¹ÎËË»*s®sO¬¬,ÍzjdÐ0ÝÌ1;"Ù1º3Þ6ÝoV#ZñiO~ Ì]$««­¾Îª»pîÜ9]äÌ3¡Ãn~³R÷Ýãñhåa·ë_gíIýdîNEEs±¾¾¾ðÚ*!+QÄÁ ¨¨H8G'±ÈêÜ×ôW^1ÍI³7]ÞëLs"­Êw0áêþ+ÚÊU0Å]Ca<þ¼s1×þDbþt°Ð¡íÿÛ~ú¦êäÙ³gQVóOhæåÒÎÎN³ÀùÒi¤Û&bÍÆ¸®¤ÿlë÷¨á¬Y¿k%®²=Æ1W«vEV'E±,ïÌîÂºªòòr3üµÝÚÚÚíÉªñÙ¼¬DÄLk¢²²Rs4àkù¬;wîhpY«q¶sÌK·Å"«k¨¬ve§pÎ×YÍsãCßßb·ßõ»KKKô6[§5â4Ë»n¿sÓ®Ü¼ÎêzkûN¬DÿÜÐÐsíF§fÎD³8pÀ¼ß#¬F®Uéº	ë²®¶ö÷ÜÜ­Ñª ²wÇùÂmØ·ºíÂíÛ·£,:¼4fÝûÖ`óÚ³Ù²m÷ûýódöþÕà;ÎÑ¼yUÑ£Bf³0²²ÅyÕ°¡§`M'%%9¥ÔpMÉË/FÕ.óòò4@æõ?»a»¸¸Xkëêê2»³FZÕÆg»VÉ	<û=uîl¶ ØÌÔæ"ev2»hEJ¨¬f ³·m¤íº[ÕÜfë´ÕêzñXRVUU9ßW]VyPèDVBV¢h	B×ÖÔ«W¯³ìFTÄ.«âØ4t.c^t¦dYzzì>Ê`Û²êÆhÜ©TPPà|·I¤44Ôÿú/ä_übO(´Ãtç[ÿOV²©vK²V­fi½1¯RëþÚµ£¿óuÖîîîPD]/¾"+!+Ñæé	º´´TÏ³æp5:´*h &e>Zc¯è²n·y:tHéùÚóí¹)j<§s5u¾¡%ÒGéééQ>p«­Á./³³³Å­Ù+'ìçîj0ùàÆr«©©©=ÙCØ¼ ªÌOMM5£U³Z³MummMsô/s§â-ÉªìÚpíRsã³ømkksþ^ÌÛsc³·ù:?ÈY	Y^tfÃ²ÙH322¶±*i¤ñpr6ÐöÖZÕ¥KÌÝ<³ã«#>Í¶ëÖÖV³@WWWGGÐûªþÒú·Ã9Ô3[ÎWªËê?­_Ë+¯¼¢ÿ3Bßká7fGyK®þé	iÖÈé3¡L£££®jï©ãÜ¯Ø¬Ó¹W°îNQQQôG¬D;yÕUôgü!¥ÑmØ¶ÙN«qü¨®®Ö?wïÞ½Ò3gÎ|?@wu.^¼¸¥Ki*å³>C9>ãÂYMMý|"d%zîi8uãÆÈ²mô4¼ÓN<öcYY÷L?ÿùÏûö~ ¬»%ós'ör¼qYw¬¿ôK¿ô÷DD9dEVd%"BVBV""d%dEV"BVBVd%"BVBV""d%dEV"BÖ­ÖÙÙi¿¼o#øu~~¿?úE@wwwUUÕéÓ§u¼½ëíííµß:ìõz7]~qqñæÍ¡ß¤¬ÈJD´²À®Ïjhh¸uës±±±öVWW;)5ß,PÍùùùæíb===º¢ÚÚÚ¦¦&IÜÑÑ!¥ó;ºÂ%¼óòòÖÖÖ·P7chhÈLGùö¤òòrËsss+++Íô¹sç"0²"+ÑeywîÜ±tÝ½×õµðbÌ788º]VëÊ%%%³³³ö»èåèêêªsá©©)#."PÍLYççç¹.eïß¿¯Ûà2Xµ'­¬bÛLhaóm[ýÊBdEV"¢íÈªQ£óà¥KVÛÜÜÜo¾yöìÙÐM©ÌYU__ûz½^¹ëZòÔ©S933£µiFÎ±oØ¤uØ¯Ë½wïF.YÅê¥KtÍÌ«W¯s5==ívëF¬ÈJDôÇ¬v )«4Ä4tµ¶¶Xç2FÖÉÉIMhÌzýúõÊ`:©Ëu¬qç¹sç´¤®NÄêXK-½®Ñ¶ÜÜ:_kÓíÉÏÏwÊ*/_¾ìë¬µ`øòòòG¹ÆÊÈ¬DDÏ$kÿøø¸=9::ê<iÉ¬¨¨0&ÙmªRVCX1é¸¸ØîµtáÂ;j´UUUIÐ°·DkmKKK¡g½öÚka/¢µI÷ÂÂB;'##ãÚµk¡KgR£d±ºGG«ÈJD´Keí]ìñãÇöRÙÙÙÎh¼«cS5ÜÌÉÉinn6k¨­­dvW¯^5ÊjÍZk¬óù|B=ÒM+«®tpppaaÁÊª+XW'ïµrçÐV7éáÃ¼Î¬DD;)k 0i<·é>MMMFA,ÍÄÝ»wíðTOffKKÙ½HÚÍ¿.]QV¶ÙéÓ§OÉ(×iÌjvÒué¸²²R£mç½0yu¬q­FØÎ¡­®Ý¾$¬ÈJD´[O<iö0²CÌÆÆÆË/»ÞÙòæoº.xýúõÞÞÞÐ×YÍ.Ä¤i¤èzÉÓ)b½&nÞ¼¹éÛ^7Õüsàúÿ µµ5ô¶!ëè:_¿uÇÙÓÓ¬DôrÊ:44ãuK²ËËËuí:v¾IÆYGGÇÙ³g&o[Vç¾Nvº¸¸Øþ [kÆèÈúLù|¾°*¯×kþWª®®ÎËËCV"z	e]]]-++3û"=zôèèÑ£Q>H²º^=þ¼SVR´6;L×E¤dÕõìºêºººªªªYKJJ¤ìãÇucåùùy¨5k,éMNN:eÕzVÏvøða³7Î.ò#ZdµsçÎMLLT¥¤¤ÿ¡ô/Ôê^út_ûÚ×öïß¿íGó·àûýî?=Tw¿ÛÖ=±S©éOxâ ¢mÈª¡0s2655%ê®¢±|ºqãýUTTäZÃÃ/--	3=ëêéTgj»ç­ð3ûCi~¿¿³³Sª9÷Ëvçèze³k]]DFz­DEßIÏóöc+¬ëÎÝ	Yc»îpªÄÄD×ô½÷¾üt_úÒtÖ¶ÍBô×[o¹_úî¯¿ñ%oU¿·#üw+OD´í­Á¬;&kBBNJJza[¿=Ùqëý·wjmÂYYuWÈj^ÊÖ±¦õdÍÏÏ7[íué+usYÍIÏàñx"½YY	Yw2d%"d%dEV""dEVdEV"BVBVd%"d%dEV"BVBVdEV"Úe²ÙO·¯­­Õ³eØÅ´Ïçknn¾råJNN½Èøø¸ë3ú[[[]×¥tÕÕUó°®´æóþ¬©©±ç...ö÷÷ÛQ¾äÿÞ½È¬ÈJD;&ë>ÿOÿyàí¿	ÐYË+?w.o>_@vuuUUUk¢ººÚ	ªæ455=~üX¤ÍÌÌ;]]ß«S__ïPÉËÊÊJ³¶ë×¯Þf­Ó|ýÎñãÇ7Pëú:Ë/---Élé®9®¤²ýbö³gÏ	ýÓ 5#+²=¬õ1þo¿û]ßtØCNþ;?Y1KÎÍÍsçÎÉQÉÚÓÓ3???66æúL|dE©2ß¥##´··ONNZØJKK+**46µÃ_;Ê´²JG«¯&t)iªc©¬!¯.®iO3 Ðm0Bo?È|»ZCcc£sÆ¬È¬D´c²êéke5h=&Éd§´F®ÆEq%Ã4óüùóNK¸µµ5Qg³³³fµ.ääß«¯¾jÖfdv~^^ØÏÎñºZ~qqñÆº®ÞÞÞ«W¯>zô¨£££³³S×ìØ1s®¢+íîîîr¤9d#+²"+½8YírjjÊ%A]/¾ùæ°:ÅªªªÒ0ñÖ­[2OàiùË/a¥ÎõÁö+W%å¥Kt-v[®.®!¯ÊÎ«ÐÚùr¯«ZL·ÄùMyv£´fj­´yõWëÔ¨©©q~/²"+Ñsuaa¡¶¶öÔ©Sf´'mjÀ'±ü~¿ Dê"/^t½x©!£ÈËËsÉªåõôkeÕhõøñã¡ß¥êä¼§§§©©Éî!eàfF«JpNNNê,ïß¿oµÖÑfk0²"+Å*ëßÿÃÿ»ñ­þÿxïûa9ùïDUçNNÿÌLËÞ76_n^Ou¥suVNN<Ýï×y2ô%Os2Ïµ¾ÎÑê3g:::4¡ãüü|eii©Í2öuV#´´ÖbZ­åYg9aFVd%"Ú|Ìú?>ú»ñ'?ýYÄ7¥üüÃ§þS(³³³b2tLiNLL;KVe°sças9÷öù|vH	i»³±ÙàìÕ¹°¡YYhç·o)ó/Þ¼yóöíÛãz²¯¯Ïëõ>zôHcJ;uîZ0³éõîÝ»ÂÏ5r®k­NY]|ÍE ®4V¾víU×e|-))±²s¾5¡ÿ	&''+**Y>YÛÛÛïÜ¹£ácgggMMÙÒ+±póóó===oÇò¯¹¹9??_ÐÎÍÍXó6yµvõêU9WTTd_Ööâ²¹0Xvv¶s£±P,///((½5HP;hnmm5ïäÑÓ»½yæÝ°+AVd%"z²²"+!+!+²"+!+!+²²²"+!ëÆÊ§ÿørøô>Á¼¼¼°»Ô>ãáØÝù®dEV"Ú²þÉÿúú»o¼ñýö ³«syû)§OÖqûÍ7<ãálð]7È¬D´×eÕ!Ò¹ÂÕ%«ïiè§4ìÔáð]7È¬DôÉêÜ`ëóì_³ÁwÝ +²ÑË#«Xrf¯hii1ydGÛþ2¾ëYv¿¬þÓ§ßùÉ÷þÛì_=ÈÎ(²¾þîÿûÿÎnÖPòâÅÌ¹ð³ßu¬[UÑªëb<~<¬Dôì²®|úw?ü£ß«ø¾åÿ»¿ôdXùÑþ,à­%Ø+WÄ¤`³ówêËpø®d5I;«2X#+=ï­Á[MVµµµiB·Ê9|ö/Ãá»nõ¹tëý·v¬«««½½½fªÕH¦±£(ÝØ/ÃÙà»nõóõ÷þ ÿkeß®æÀ3½1«íÒ¥K¡3ñËpÌL¾ëY?Y¥©pÕÈUüwx!¢,+!k¼ÉêYYYYY	YYYY	YYYYY	YYYYY	YYY	YYY	YYY	YYYYYY	YYYY	YYYYY	YYYYY	Y^*Y=ObbbfffOOó¬ÞÞÞsÏçCV"BVBÖÍóz½õõõ¨®®ÎËËs2==­	§§§#+!+!ëæÏõõuMÕ5­ût¿ñ¿±ÿþ=$kMÓC~ôYugJLL;m6'''ë§ãÍùã?þcïÓå+_ÙC²Öµ|Êª¸mëàY	Yw¦;ä<ëøñãcccØììì8Ø¶·ªßCV"d!dÝ±RSSÙ¬é³ÈJDÈJÈ¾üüüºº:MèØëõ:ÏÒ8uttT###¿"+!+!ëæù|¾´´´Çã÷ûqS²ÇTVu¬id%"d%dî!+!+!+²"+!+²"+²²²"+!+!+²²²"+²²²"+!+!+²²²"+²²"+²"+!+!+²²²"+²!+²"+²"+!+!+²²²"+!+!+²"+!+!+²²²"+!+!+²"+!+²"kÄ®ÿàÎëï¾qeàëÎÃoöÿÛ?üd%"dEVdÝr2épª>¬®ÈJDÈ¬Èº3	WYYY	YYYYY	YYY	Yu²þîÛßÿêõïÔ4ýpÇí=M¬¬/¬ïM=V¿Vö=Í²²¾t²>§üC?®<a!+!+²"+²²"+²²²"+!+!+²"+²²"+²²"+²"+!+!+²"+²²"+²!+²"+²"+!+!+²²²"+²!+!+²"+!+!+²²²"+²!+!+²"+!+²"+²²²"+²!+!+²"+²²"+²²²>Ïþúoðú»oÄ~hû_ßEV"d%dEÖéÖûo²YY¬z@>úRHííí:«¼¼YY·&knn®îïï×É±±1VMM²"+!+²nMVÕìþýû¾rå³vtt +²²"+²"+!+~²VTThbÝ¹eXµ´´,//#+²²"ëÖ©7nÜóóóÎW[YYuµ¶¶Â<ÐtQQ¦oß¾-nYYuË-...--I¸êêê´´´®®.33Æ·Üz<w333zzg­®®$%%½öÚk½½½È¬DÈJ/¬f¯%U__ßØØxèÐ¡S§Niðªýâ^¯WKjB0çåå9Ï*++ÓÊ5ö«Z-²"+²ÒK!«D¬¨¨ÐÃÂï÷|>­ÃÃÃÍÍÍÑ/b¶ôôtçYÅº^¬ýÖ·¾õ/.;;[ã]dEV"d¥¸ÚéæÍzXh|ie]]]-))Ñ´kohr1ì´9YYY¬«Ö?ÿó?/yº¢¢¢ýû÷#+²!+Å¬sssz@?~aa¡­­ÍìÍÔÞÞnÎmhhÐÉ¦¦¦(kHHH°ÓIII®³jkk51>>ÅÖ`d%BVsYÇÆÆRRRìÉ¥¥%=8^yåç2>[²ÔÔÔ@ `¶kÚuV¤á,²"+²R<o~òóóëêê4¡c¯×ë<ëÚµkf¼;::zâÄ	dEV"d¥øUãÑ=&rrr¶w­>/---!!Áãñ ÌµÁ·îäææj´511¬ÈJ¬Ï²uuu·ÖjÎãÇ8 ½Á¸æ_È¬DÈJq5f]^^ÖÂ|:ÄÂÂkg`¬ªªBVd%"dEÖMZ__ÏÌÌÔ¨ÔìýkÞÏ:00 ãþþ~§¬È¬D¬ÈºyfÞ= ¦¦¦WdEV"BVÚÎïÕ|®¡ÙYIV¿ß_PP`>y_ÇæËã2330õõõ!+²²"k¬VÁ©ãÉÉIó«Ý2l@Vd%"dEÖÍKJJÊÏÏ»¸¨¨hvvvffæÜhdEV"d¥xuzzÚ|Æ¯KVÏ§ØdEV"BVdÝBFVC©¸ÕtKKsü¬ÈJDÈ¬±¶¸¸h^gÑÄøø¸óÜÑÑÑçýpAVd%BV+YÇÆÆôðù|ahkkCVd%"dEÖ=SÈú÷¿UõaÝGLF9|¼8¬DÈJÈ¬1õÖôú»oüÚ_ýv¤ÎÝÆ Yöª¬kkk+++fº±±ñ?>â@Öç´¹Yöª¬ÝÝÝzLTVVj:=²"+!+m_V= Ít½SVgYYu­¯¯ëÚ¬¤¤$))éöíÛ.]ÒIóyýÈ¬D¬ÈkÍÍÍÎï6ollôx<lFV"BVÚØö·Èil¬ÈJDÈJ; «Î9.YÍ·£#+²²"k¬²®¬¬ås¾ÎªiÛ/EGVd%"dEÖX[[[[__gk0²²ÒÎÈek0²"+!+²"+²²Ò.ueeå¹îÇ¬ÈJ¬'²®¯¯gdd;v¬½½ýR0MÜ¸q#99ùÖ­[vù°ç÷aLÈ¬DÈJñ3foªAVd%BVz©·#ë®5'ÿ·ªßÓáwßþ~ßS<y!+í1YGFFôàhllt½Î¬¬?ùx¡­Â¾zý;5M?äÉYi·Ëj?#Â¼¤êõzõà(--µ/²Ö××kNNN²¾xYUd%BVÚ²®³Â~Ø![Y·áû222xYYidíèèÐÃ¢¿¿ÿ|0ç[nJKKYYukÛõ¸|ù²ó7_ÝªùeeeÈ¬D¬Èº4*Õc¢««ËÎÕòòr¶#+!+²n­ÆÆÆªª*=&:;;íÌÞÞ^³qØjYY5¦WSSSÍîKÒôx°²²2Íñz½6#ÚdEV"BVdÖôôtvv¶Ý1x``À~Ø¡F«ÏUSdEV"d¥8ß7XØÈ¬D¬ô¬²:÷WBVd%"d¥uzzº¾¾¾¸¸Xsd²"+!+²nSÖ¶¶63=??¯×¯_17YY)eýd½1|O¸úf¶zýxYö¬ýýýoó>]KK%%%%Èº#=úváûÿþÊÀ×·t8Ùû¿öW¿¬DÈJIÖ@ ú)«««æ=¬Èú9¦1«|EV"d¥½$ëèè¨óäüüüàà ===:¹¶¶¬ÈJDÈ¬[Ûq©®®®¿¿?))iã³à×uddDæÓ«««YY5VY=zd&ZZZN<966¦ÅÅÅKKK®ÏFVd%"dEÖMd­­­µ'ÍKgÎ1/¾.//#+²²"ë6e/,,Ô[·n9È¬D¬ÈºeYý~¿&º»»5f=ö¬&¯È¬D¬ÈºeY%¨ñÕl>xð &®¬ÈJDÈ¬ÛÙ<33366VUUÕØØhÎ¨YY·FGCÕæææ/êdJJNajhhÐ¬ÈJDÈ¬1ÕÝÝí<éú<&3fDVd%"dEÖ=²"+²²"+²²"kÌwuuéa±¼¼<33c6ëäµk×YYuË»wÓÄââ¢ÛÛÛÍçò»^EVd%"dEÖÍ7¬ãüüü¡¡!Ãí'böx<æKr¶Yâ_V¿ß¯jmmí;wÌ×Úè¸±±QÇ.V^^n?J"l^¯·¾¾^ºx^^^èÖæcÇ!+²!+½²o>ÙÌ|]ëÔÔ¦;::4àÀ£GF_IJJy»Öî:÷Þ½ö1÷o|ã_<.¢ñ.ÊÿÎ×Ê¾§ÃW¯gfö	?"d¥]º5Ø¼¶*ü®^½¥éG,cÙ,,ÃN«ééi­Ðìeætww?xº»wïîß¿?Ëè²NMâú±9ØMÍóó!BVÚ²ÎÏÏëÑ011a^]ZZòx<¤ê¤¿jfQQQôÈc;m¾AÝÛ××gÖÃÖàgÕÆ¬ÈJ¬´e£v#³û±Ödt¬aeôõ¤¦¦³5XÓ®!+²!+ÅùU²Ôööv=&<ØÚÚª	óVóYü:öûýÑW¢5ÔÕÕiBÇ^¯7ü-cÌ¬DÈJ/¬«««:Ö¨T¸jâÑ£GvÓnqq±(¹¹¹®Äçó¥¥¥éÇ2ìz!+²!+½,0Ý=:00`>&ÂÌokk³ß1÷%BV+YMæKÎÍ'ÊZóñ¥¥¥:îííEVd%"dEÖ-ÀöàÁ,..êdkkkUUùáà?h:!!aaaYY£º»»³²²VVVÌ±±1Çc>#Â¦1ëôô4cVd%"dEÖ½²"+²²"+²²"+²îAY_÷oOv¸ÿºâAíÈ;¡óG?çF¬¬È±>¼þ;·ÞÛuøWz£dà¾k¦®ú°²²"ë»5X¬"+²²"+²!+!+²"+!+²"+²²²²!+!+²"+²²"+²²²"+²!+!+²"+²²"+²²"+²"+!+!+!+²²"+²²"+²"+!+!+!+²²"+²!+!+²"+!+!+²"+²²"+²!+!+²"+!+²"k|ÿÎ¥ßéþZÙ÷¯üé=9<~øµÍ#<	YYã§ÿÖ?ôc×á÷¾ÿPÐùvüðVõw*ý<	YYã<¶¿°Úº'+?BVdEVd%d%d%d%dEVBVBVdEVd%d%dEVd%d%d%d%dEVBVBVdEVd%d%dEÖ½!ëëï¾a|uZ';ùá +!+!+m¹÷þöoBY½2ðõ_û«ßæ¬¬¬´3ùf+?d%d%d%dEVBVBVdÝë½b~ÈJÈJÈJ;ÓGLò:+²²²²"+!+!+²"+²²²"+²²²²²"+!+!+²"+²²²"+²²²²²"+!+!+²"+²²²"+²"+²"+²"+!+!+!+!+²²²"+²"+!+!+²"+!+!+!+!+²²²"+²"+!+!+²"+!+!+²"+²"+²²Ò^uppÐãñ$&&ffföôô8ÏòûýÇÓYGÕbÈ¬ÈJÈJÈºy^¯·¾¾^ÕÕÕyyyÎ³>Üßß¯ÆÆÆ#G +²"+!+!ëæ¥¤¤¬¯¯k"¤§§GZ,99YÇW¯^MIZþ,uÉÿÎW¯GMüeÿ$?BVdÝáäbØigfÓñ·®¶¶6))?KdÝ+=ù»þhjÞßê´üLYuÓ ÓNËÈÐ¼^ïòò2[5Îz«ú=d%dEÖ/555­Áv;;;[PP077Ç¾ÁÈ¬¬¬1_WW§	klê<«··÷Ì3²3ÊÅYõ©|>_ZZZBBÇãñûý¿¸)ÁYzzú>GÈ¬ÈJÈJÈúÜCÖç*ëëï¾qëý·#¾ñþ·¾ùA5?(d%d%d¥údñÉ·';¢ª>¬½ü Ø¬¬¬È¬ÈJÈJÈ¬ÈJÈJÈ¬ÈJÈ¬¬¬¬ÈJÈJÈ¬È¬ÈJÈ¬ÈJÈ¬¬¬¬ÈJÈJÈ¬È¬¬¬È¬¬¬¬¬ÈJÈJÈ¬È¬¬¬È¬¬¬È¬¬ÈJÈJÈJÈ¬¬¬È¬ÈJÈJÈ¬È¬ÈJÈJÈJÈ¬¬¬È¬ÈJÈJÈ¬ÈJÈJÈ¬ÈJÈ¬¬¬È¬ÈJÈJÈ¬È¬ÈJÈ¬ÈJÈ¬¬¬¬ÈJÈJÈ¬È¬¬¬È¬´¥~ïúsòßùêõïpØÆá+ÿ]ÇÈJÈ¬ÈJÿÜü'?ýÑÔ<íüC?®ÈJÈ¬ÈJ´3	WÆ¬¬È²¾þîW¾þbRüÛüØ	Y	Y5nûéÏ~êxaáZõa?vBVBVd¥I¬"+!+!+²²²"+²"+!+!+²²²²"+!+²²²"+²"+!+!+²²²²"+!+²²²²"+!+!+²²²²"+!+²"+!+²²"+!+!+²"+²²²"+!+!+!+²²"+!+!+!+²²²"+!+!+!+²²"+²"+!+!+²²²"+²"+!+!+²²²²"+!+²²²"+²"+!+!+²²²²"+!+²²²²"+!+!+²²²²"+Å¬'11133³§§'Æ³Y	Y5|^¯·¾¾^ÕÕÕyyyÑÏÒãïã§ûðÃxAVBVd%dýE)))ëëëéééÑÏ*((ØÒ¾ðe^ª?¬GV²MM¬¬áKLL;ö¬ýèGýtY_>Y|2ûÓ9~d¬¬aJHH°Ó22Æ³xâ)dEÖ,555M¾ñ,d%"d%d_~~~]]&tìõzc<YY	YÃçóùÒÒÒ<ßïÿÅM	>ÈÂ¬D¬¬Ï1d%"d%dEV""dEVd%"BVBV""d%dEV"BVBVd%"BVBV""d%dEV"BVBVd%"BVdEV""d%d%"BVBVd%"dEVdÝU²&%%ýh/¬ÈºZXXÐ5i»%$$$&&&Åcº_ºwIq¿¸½ûÛ¿µ/ÛöÅù5N:räÈ7¿ùÍ¸¼kuuu¿ò+¿¯¿¸_ýÕ_ý?ù¸¼k÷ïß?|øpµ@  YGGG»âââS§N	!+²"+²"+²²"+²"+²"+!+²"+²"+²²"+²"+²"+²²"+²"+²"+!ëçßï½¸¼k£££ø¯¿¸ªªª>ø .ïÚÀÀ@mmmµO?ýôí·ßË×ÛÛÛÔÔ$¬DDDÈJDD¬»°ÁÁAÇÙÓÓ÷Èï÷;vL÷èèÑ£ºwqy»ººìÀÅÍ½[]]-((HJJzíµ×zãì§aîÏç÷äÉôôô(O&ñ÷§GÈS^¯·¾¾^ÕÕÕyyyqp>Üßß¯ÆÆÆ#GÄß[Ó¿VÖ¸¹weee÷ïß___B³_Êôô´&tl4Úë÷NÿèçÇüÞ£øz!dõ^ÏeÁýÿ~ÆGÉÉÉñwïÝ»WQQaÑâæÞiX366¯Ný¯0;;«	ÿöú½;wîÜÄÄSÖÐßO/¬KLL;ÆÙÔ'++KÏVö-nîn|ee¥þ<ÃÃÃqöÔ]ÓoMÇzdÆÍ½sÊzâøé5Z			v:)))nî×ÒÒ×ë]^^³ûÛ××ç|F§;bÞf3>>®ÿâìwüøq3"±ÙÙÙqsï²Þ£xz!dÝ¤ÔÔÔ@ °Üéø¸S³³³sssñw÷=]<Ý;ç7ãxúÅÞâãÞ9e½GqùôBÈºyùùùuuuÁÏ*Ò /îQooï3g<yÇ÷Ñù7÷îÚµkæFGGO8g¿8Su¿4122¢ñkÜÜ;§¬¡÷(.ÿôY7Ïçó¥¥¥%$$x<¿ß÷(==Ý5ª¿ûè|F·¸¸«ñÖÄÄDýâ?~,Puït¬é¸¹wNYCïQé²!+²²!+²!+!+²!+!+²!+!+íÖÖÖVVVÌtcc£óD¢722¢uçL»ªÍôûöý/Í¿sçÎóýÛ·/''§½½ýÒgiZ3¯ÂãYµîîn¡RYY¹ü:<iÉ¡¡¡Ü`"¹¨ZêëëZfùäää'Oj~ss³æ»¾ÒKs<xz-SSS:K×Õ××ÌåËò(MNNýGA3O::óìÙ³¡3ÍOh8h¦E£SVgK®3ÓO<Ñ+**¢¯Ù|øòò²¦[[[]ç>|øÐ®Ð.6::*q5_Ã_ç¹öRÑ¯ÔÖÑÑ¡cõÂ®ýýý/Ýy!+QL.ÉÚ¬¤¤$))éöíÛviã°ægddlj¶SV5óÙWWWëØãñåNj«éãÇ·ÓÍ;wî®+Ï§%ïÞ½é©Ð­Á×¯_]xllÅDÈJkb¬ªªÊ~Ö¹MÏæÄ©´´4¬µµµÎ1¨³:WëÕ¹UYÓG]V³/F!?ìßÐ1«)ì+ÊD¬Dá[Õs/_¾¼¶¶æÒ¼ZVVæjÐÙÐÐ 'O8_jÝTÖÓ§OÛ%O<»¬ºF-611å^ÛAp,²s333yÀ!+Ñ&¬º^³tÉ*êB/¥Q©.ØÕÕeçjNyyyèúcVQgöòù|±ÈjT6LæççÇ.kØÛ³ªÛ·oÇ¾×4!+½¼²®¬¬Ø=~¯³jÚXÒÔÔä¢·ªªJó;;;íÌÞÞ^³qØjÃÊjÇvï¡ç7f½víÓF§7Æ×YcÔè97êFßÜÑÑj¦ÇiLYVVfÞªi3¢$ëôô´µê9ÉªÿÌûv¢0yïÞ½-YcÙcÈ]Y%bvv¶ef``Àn+6oM1`GGG]ç>§­ÁæAFúO"ì»T£ÈjÞbëºµD¬DÏ$«!'oQd51kww÷d-**²KÞ¸q#vY7oÌµ«reQV#qVV"d%ÚYWVVB÷crí¯EÖ×Ì¥¥¥ãÇý4%#«ë]3ÚÚÚºººÌ@6ûb^vP¢Ù±9ì§'uppPó9ÂcY6IÆddd;vÌîÈ£	oÝºåÜ¯G¹>ÉÊ:==­Ñaqq±ySMìWv °C[Ûüü¼Î=xð`ì÷Ñï÷»®%t7¥ááá+W®ý?@3KJJx´!+QLSML×û4te¯Ú]ôg¶oß£GÂUSSãPYYYYYYYYBûÿ©ã8±¥îóIEND®B`


COXREG time
  /STATUS=status(1)
  /PATTERN BY T_stage
  /CONTRAST (tumor_size)=Indicator(1)
  /CONTRAST (T_stage)=Indicator(1)
  /CONTRAST (surgery)=Indicator(1)
  /CONTRAST (M_distant_metastasis)=Indicator(1)
  /CONTRAST (sex)=Indicator(1)
  /CONTRAST (smoke)=Indicator(1)
  /CONTRAST (chemotherapy)=Indicator(1)
  /CONTRAST (age)=Indicator(1)
  /CONTRAST (N_regional_lymph_nodes_metastasis)=Indicator(1)
  /METHOD=ENTER sex age smoke tumor_size T_stage N_regional_lymph_nodes_metastasis M_distant_metastasis surgery chemotherapy
  /PLOT SURVIVAL
  /PRINT=CI(95)
  /CRITERIA=PIN(.05) POUT(.10) ITERATE(20).


Cox 回归


附注	
创建的输出	20-JUN-2020 13:54:58	
注释		
输入	数据	E:\医学\肿瘤内科\孙佳春老师的肠癌统计数据\modified\data_modified 赋值.sav	
	活动的数据集	数据集1	
	过滤器	<none>	
	权重	<none>	
	拆分文件	<none>	
	工作数据文件中的 N 行	55	
缺失值处理	对缺失的定义	用户定义的丢失值作为丢失对待。	
语法	COXREG time
  /STATUS=status(1)
  /PATTERN BY T_stage
  /CONTRAST (tumor_size)=Indicator(1)
  /CONTRAST (T_stage)=Indicator(1)
  /CONTRAST (surgery)=Indicator(1)
  /CONTRAST (M_distant_metastasis)=Indicator(1)
  /CONTRAST (sex)=Indicator(1)
  /CONTRAST (smoke)=Indicator(1)
  /CONTRAST (chemotherapy)=Indicator(1)
  /CONTRAST (age)=Indicator(1)
  /CONTRAST (N_regional_lymph_nodes_metastasis)=Indicator(1)
  /METHOD=ENTER sex age smoke tumor_size T_stage N_regional_lymph_nodes_metastasis M_distant_metastasis surgery chemotherapy
  /PLOT SURVIVAL
  /PRINT=CI(95)
  /CRITERIA=PIN(.05) POUT(.10) ITERATE(20).	
资源	处理器时间	00:00:00.34	
	已用时间	00:00:00.52	


[数据集1] E:\医学\肿瘤内科\孙佳春老师的肠癌统计数据\modified\data_modified 赋值.sav


案例处理摘要	
	N	百分比	
分析中可用的案例	事件a	49	89.1%	
	删失	6	10.9%	
	合计	55	100.0%	
删除的案例	带有缺失值的案例	0	0.0%	
	带有负时间的案例	0	0.0%	
	层中的最早事件之前删失的案例	0	0.0%	
	合计	0	0.0%	
合计	55	100.0%	

a. 因变量: 生存时间（月）	


分类变量编码a,c,d,e,f,g,h,i,j	
	频率	(1)	
sexb	0=女性	13	0	
	1=男性	42	1	
ageb	0=≤65岁	24	0	
	1=>65岁	31	1	
smokeb	0=无吸烟史	25	0	
	1=有吸烟史	30	1	
tumor_sizeb	0=≤6.5cm	30	0	
	1=>6.5cm	25	1	
T_stageb	0=T1-T3期	38	0	
	1=T4期	17	1	
N_regional_lymph_nodes_metastasisb	0=无区域淋巴结转移	20	0	
	1=有区域淋巴结转移	35	1	
M_distant_metastasisb	0=无远处转移	36	0	
	1=有远处转移	19	1	
surgeryb	0=接受过手术治疗	21	0	
	1=未接受手术治疗	34	1	
chemotherapyb	0=接受过化学治疗	30	0	
	1=未接受化学治疗	25	1	

a. 分类变量: sex (性别)	
b. 示性参数编码	
c. 分类变量: age (年龄)	
d. 分类变量: smoke (是否有吸烟史)	
e. 分类变量: tumor_size (肿瘤大小（厘米）)	
f. 分类变量: T_stage (T分期)	
g. 分类变量: N_regional_lymph_nodes_metastasis (N分期)	
h. 分类变量: M_distant_metastasis (M分期)	
i. 分类变量: surgery (是否接受了手术治疗)	
j. 分类变量: chemotherapy (是否接受了化疗)	


块 0:起始块


模型系数的综合测试	
-2 倍对数似然值	
297.164	


块 1:方法 = 输入


模型系数的综合测试a	
-2 倍对数似然值	整体 (得分)	从上一步骤开始更改	从上一块开始更改	
	卡方	df	Sig.	卡方	df	Sig.	卡方	
280.571	17.287	9	.044	16.593	9	.055	16.593	

模型系数的综合测试a	
从上一块开始更改	
df	Sig.	
9	.055	

a. 起始块编号 1. 方法 = 输入	


方程中的变量	
	B	SE	Wald	df	Sig.	Exp(B)	
							
sex	-.255	.492	.268	1	.605	.775	
age	-.252	.346	.528	1	.467	.777	
smoke	.531	.443	1.435	1	.231	1.700	
tumor_size	.210	.378	.309	1	.578	1.234	
T_stage	.836	.394	4.500	1	.034	2.307	
N_regional_lymph_nodes_metastasis	-.472	.324	2.119	1	.145	.624	
M_distant_metastasis	.284	.498	.326	1	.568	1.329	
surgery	.733	.480	2.326	1	.127	2.081	
chemotherapy	.376	.353	1.131	1	.288	1.456	

方程中的变量	
	95.0% CI 用于 Exp(B)	
	下部	上部	
sex	.296	2.032	
age	.394	1.533	
smoke	.713	4.051	
tumor_size	.588	2.588	
T_stage	1.066	4.996	
N_regional_lymph_nodes_metastasis	.330	1.178	
M_distant_metastasis	.501	3.524	
surgery	.812	5.334	
chemotherapy	.728	2.911	


协变量均值和模式值	
	均值	模式	
		1	2	
sex	.764	.764	.764	
age	.564	.564	.564	
smoke	.545	.545	.545	
tumor_size	.455	.455	.455	
T_stage	.309	.000	1.000	
N_regional_lymph_nodes_metastasis	.636	.636	.636	
M_distant_metastasis	.345	.345	.345	
surgery	.618	.618	.618	
chemotherapy	.455	.455	.455	

a]]ÝòåËK~õë×çS¾·Dºå¯¾új|ÃÝ»wÇòµk×ªü:iésÏ=×/,1õL?:ûÖ®]ÿª(n~³jIVÇÆÆJæÜ%ÜÔÔ4¿ìíÛ·cc¢úåÄMíêêJ78~©Hi,;w.ýYÓï¿Tÿ)È*TÝ-J©ï½÷^Æô(Õ(>¦º?(ÇÕÒÛ8ûí·³köõõÅt¶âÒÆòöïßÛ´Á¸aï¾ûn|IT!ffirÉD3mUÎÎFcÍÆc9f`¢ôÛÍ7/>úè£43+ÏdÚÒ0"~øÑ¯ÓÇl9Æ0m5Í¿<g7oÞUÙst«âÓò#Gªl@kzfPþ~£òëÇ46.¤~<g*¹fzcóàôyÜ%bKKKùà¬ÂÒÞ@U6§ïìÕÍL4#íL½Ä:tëÖ­xXÏ?ÇuLi¯*~Pä09%¿d¶Øßß_r;ó³Õô¢R±ß¤¯yðÕæÜ;wT$ý:ÙæÓü«ùMÖ±|øðá½ÏOFÓL=ã«oÎÄ5#öi@2i+BÉëXóÚk¯¥ß(Å4¦¤çÏO»zEYKô¤¢Óóxr§ÃÃÃùW£kÜdz8NYÍffY9¢1©zÿý÷ó×O!IóÎÓì-~¥kööö¦ùYÖÑüÔmÏ=å7,e5Â²kÎ=JVÕk×®¥9wºÑ4WË~i&ÿ1-ÉÙöíÛ³Ä¦hþ×)Jd5¦Âñ³âÙÀøøøtÕÌû²Z¾ëP<ÅaÉ^£-¹rÌ;|µuH¦ÍÚéiAöÂsº~z¹7®=ká=wî8Î¦]k¼ «P!«)$ioÚ¤àiwÜl6»Ñªx­2¿5í6´vvvÞ»w/¿ãkùÄ·$NÕ³zãÆü­Í"ÛI6¹L³ÕÕüþMg«iMÚÐ^S	bIVãw9uêTþK¾ÿýï¼IlØ°¡¼iÆ­´ä1eL+¶±§­¸ýò¿cz?+áêÕ«i;vvü/¶4DY³Ü¦ÛK,Ä_Áÿ²Õ4¹Ó´Í0Õ¥©©©­­-%*ÖÇ¤'*E" ³cþA¿âþ¨)Biâ´¶WyMÉ.KILªâ,0!½E5¢¦¡YjÉjcccaÃå7xVße)ÿ ßõÕ«WÌzÓú(VüÜW§¿¶-âÿ)1ý]·n]Åiît/vfYM9L3û4Û¶m+ÿ¥ÒNÝù¥³kÆ5Z^ôµ³²ÕlÞYòÀÓ+p7æö0Êï6{VüãOÕèh±XÌæéÝ )Ãåoc-ÉjõÀÙÙÈ^L¾ãìÐÐÐ£fµ<riº6®æWæmTß¸ÚÛÛ¤-Þ1%UÚþÖ[oåw(KQLÌ6Ñ¤=¹J~©´ËRz-<»¤	wÚlfºÓí¡²3ÏVÓl)Í´²]s³	Pù~¤I*n<^§¦VÙô÷÷§ÝÊãý%Ü¸Ñ¸Ué]+ie@þeÅ8^.«3n.ggg«fhÆ¬O·n-YLÆdWg%=®2[=pà@~×èì¦¦N§§)%ÏRqÓë¬ùãFÅr¬/ô¬Âl²ãq9ÿÉ#M0¹eJþý!Ùl5íV?:¥´Ù9­9eÔ½<E÷­¶ÿªÎÎÎtÔM6EÔG§L7×VËúV²ÓríY-¹ZÚ©Æ¬¦ç@áúò]_jº)øÍ7.]Æ<»4nÆßdªe5íò²1ÉK§Õ3vóIàõõõÓ¥èÁÔ&Çéæ|IÄ IùCïÞ½ù5iÛfWW×#ÍVcZ³Êç½õ6Äõ×®]³fÍwÞygãÆ1ß¶m[Ú»çAÙûV³CÄL:ÿïÙgqÌK²zãÆwÑÌ:«ÉþýûÏ=¿BÅ¬¦=ªÒôrlÚÏrÒóìéÈ*ÔÕ+WÆ4.?¹IG!îà>Ô¤ÞÄd(í^áGð.¤NDÏÒKåÇ;,YSÊµaóæÍiscccùïlß¾=¥1ûþ[·nMýÈ^C=uêTüù7Æd»¾¾¾ôM¢¬%ÈO£Kv§JÐ³½jcÇU<°Tö^Þ*GÈg5®öÊ~ë­·Ê%½?UùMk§ùzþ.q²mìÙüìhú¶ù<Y²)OTùä)Ö<yòðáÃÏ=÷Ütï»0§EÄ4.í8§%o/ù²÷ÅVÞíÙ³§â®È¿-_'H%ÚÔãáááW¹fÉ&écyÊ-	/?øCÔ·â±±@V@VYYY<îÝ»w~«þû¿ÿû)Éêçþ½ïïÀoÉ~ðþç~z²ú»¿û»ÿ~KþøÿXV@VeYUdUV@V@VeYUdUV@VeYUdUV@V@VeYUdUV@VeY­dll¬¥¥¥|ýàà`sss]]][[Û¹sçdYAooïÂ?½½½½««+:;;W¯^W¯^xØéÓ§eYýÒë¯¿~ùòåYmhh4]»vm¡LLgç|/_ÿ­­­î7<YýògWÊjô²dùöíÛ7644ô¸g«1!ÊºßðdgµX,fËõõõ¿­×Ve§!«i#p,Ë*²:û¬vtt<x0â´½½]VÕÙd5ííímjj*ÍÍÍýýý²¬>ñUdUVUY@VeYÕ²úÂ/|såÊw#du6zzzÒñÇ2@Vçròj0²*«Èª¬ «²¬ÊêÌYöÙgOçôôô¸WÈª¬ÎÆ¯~õ«ÖÖÖå_yá¬ÊêøäO¼ß@VeUVUY@VeYUY@VeYUdUVUYUdUVUY@VeUVUY@VeYUdUVeYý¶duþüùä|úé§îd²*«³-­_ÄÆYw2YÕ¹¼Ú& «²*«Èª¬ «²¬Êª¬ «²¬Ê*²*«Èª¬Ê*²*«Èª¬ «²Z5«Ba_ÎÇì> «²:×®][¹rå¯¬X±Â÷dUVçlòj0¬Êª¬ «²¬Ê*²*«² «²úfµP(,ÿÊ/¾§î²*«³7ïtÎ¾û¾Í·Yýü	mÂ²*«²¬Ê*²*«Èª¬Ê*¬Êª¬ «²¬Êª¬Èª¬Ê*²*«Èª¬ «²*«²*«²¬Ê*²*«Èêã088ØÜÜ×ÖÖvîÜ¹üE===.LõööÊ*²:ööö®®®Xèììzuþ¢ÑÑÑXÓXÿÍÃþã?þCVÕÿßÎÉÉÉXHíÌ<ÿüó7nÜ8åXX»vm¡LLgeYýBD±ârÚ><oÞ¼gÄÿüÏÿüýò¿4[@V¿T,³åúúúüEK.I]¶l×VÕ466NLL¤À±DVVÕ:::<qÚÞÞ¿(f¨ÃÃÃ±0443WY@VgÐÛÛÛÔÔT,ûûû¿¼)/nÌ+W¢¦1OÓXUdÕá d@VeUVUY@V¿+Y-ûr>þøc÷HYÕÙ¸víÚÊ+×|eÅQY÷HYÕ¹¼Ú& «²*«Èª¬ «²¬Êª¬Èª¬Ê*²*«Èª¬ «²*«Èª¬ «²¬Êª¬Èª¬Ê*²*«Èêw5«Bá'9ý×í «²:¿þõ¯óM]³f_UY³É«mÂ²*«² «²*«Èª¬ «²*«²*«²¬Ê*²*«Èª¬Ê*¬Êª¬ «²¬Êª¬Èª¬Ê*²*«Èª¬ «²*«²*«²¬Ê*²*«² «²*«Èª¬ «²¬Êª¬Èª¬~²Z(Z¿òû¿ÿû?øÁÜ_dUVgãÖ­[ä>:Êêþ «²:7WÛdUVe@VeUVUY@VeUVdUVeYUdUVUYUYUY@VeYUYUYUdUVøvgµP(¬X±bM'NÄE;vìÕYgÕÇ¯|³ºjÕª´Ü××gGFF²öïß/«³ããW¾£Y'¦|øá±¼víÚl¶zòäIY«É«mÂ²*«² «Õ;wÆÂääd~p8zôè;wdUVdõDP»»»ëêêÞï½H]þÖêãkÛÚÚÎ;¿èÞ½ëÖ­«¯¯î¹çzzzdUVþ¬;v,mÞµkW,oØ°!·nÝ­­åËÛÛÛ»ººb¡³³sõêÕù¶oßþáÆ÷¦>ÿüó²*«OVoß¾=>>>66]ljj:sæLZYã»kRcÛÒÒ¿(æ¯%SÞ?ÿó?ÿ_?~Ìt¿#Y_ö¹7<mYM»)tvwwÇ´rùòå1m³±²£££úG+.§³»wï7o^|Ï/¦7ðt=ìç?ÿyýwáÏöé§æßÆâ¬3ÀÓÕ¤îÜ¹3âûûûÓL½½½1a9r¤úÅl9YrÑbáÒ¥KK,ùo.'«OaVÃüãxikÕ÷îmÞ¼9KöB*×ØØ8116ÇrÉEÓMdeUVÂ¬Þ¼y3Üßxã[·n?~<í¾tâÄté¡CâìáÃ«|ÆB¶··ç/Ú¸qcúÚááá^zIVeàiÎêÈÈHCCCvv||<èï÷~/>úèþýûU¾IoooSSS±XlnnNÓ7í÷´jÕª§.Y²äòåË²*«OùlÕ'ØÈ*¬Êª¬ð­Ìêýû÷=ñ¯¼ò¬~ÃYõ6V§'«###gÎIïO5W®?þªU«Ú§¤Ü¦#ËêãÈª·±<m³Õ;wîÄz:¸Ò­[·JÞTg÷ìÙ#«ßLeÀÕÉÉÉ¶¶¶¦wÑ¤#BÄi___>«ÝÝÝ²*«²:týû÷Çúµk×Jf®²*«²:Ë1OÇmUYÕÙgµ¿¿ÝºuéÃjâ4Òj[[[~¥óçÏËª¬ÈjMSÕ¨f^½z5½ªmÎ®000 «² «3¨¯¯ïèè¨¸áwÃ7nÜ¸~ýº÷­Ê*¬Îltt4[IVãì©S§R_×­['«² «ö¾ÕÔÑhm,=z4?s]¶l¬Ê*¬ÖäöíÛéµÕ¡¡¡X¸téRþÒáááôq4²*«²ZÓ!ãýòåË½½½¯püøqYUYõ	6²À7Õû÷ïß½7-www?ÖM¾²*«OyVÏ=è»wïå)²*«²:ûÎ7/-wuuå³YYUYÏ±GóÆÆÆS6oÞ_¿uëÖ5kÖÄÙt¸`YýÆ²ZÂ<aY=räHþãT»»»m6yÕY8f¥²*«²úu³>½<¿¦$«éYeUVdµ¦¬Þ½÷Â«¦ä_[å8Í>UVe@Vkßêää¤À² «s|%YUYUYàIÈêÝ»wëK²Z%«ÞÆðÄdurrráÂ/>qâÄ)±ðÞïÍ7ïý÷ßÏÖ¤ôÇw¸%Y5yxJf«ßÀûgdUV¾+YõÁp²ÀãÊêÐÐP<vwww¼¶*«² «3Ë^FmooÇî-[¶d/¬vuuÅW^yEVe@VgÞk);ÄØØXù±mUYå§®.Ðk«² «_7«'OGí¾¾¾7¦äß]³eËYýdÕÛX¬¦O2ûí·óNÅë·oß.«&¯²Z«Æcô3g²5ÃÃÃ±fÇ6Ë*¬>ÚÑ÷ìÙÑ§NÊVöôô¤mÂÙtVVe@Vg~Iµ±±1í¯)]:åèÑ£Û·o5ííí±æ²CCC²*«²:­ÑÑÑeËe»dÇ2yêcM©¬Ê*ÀS8[Í¿»f×®]ÙYYUYýZYÍï $«² «sÕÑÑÑ®®®M6Å±±1YUYMV?u.Î¾ûî»²$«²êádYEVô¬öõõ­Òþ°£GÆ÷æÍeUVdµVåQºwï^:ÄîÝ»eUVdµVÃÃÃ%ìsçÎÅÙû÷ïËª¬Èê#ì|ðàÁ¾¾¾úúúìSkb¶:44éû²*«²ZSV÷îÝ>|ôèÑ_~ydd$ÎnÚ´i||¼ä(ü²*«²Z-«ÈÎ¦=^õÕôë;wdUVdu6Y½téÒúõëcÍûï¿ÖÈª¬Èê£eõâÅýýý±pöìÙ­¾öÚk±â*«² «ÕÈgkÚÜÚÚ©¬±RVe@Vy#ðõë×GFFöìÙÓÝÝ.­Ê*¬Öjll,czäÈ·Þz+Î644ÄÙt ¥CÅÈ­¬Ê*¬ÎììÙ³ù³%G³ÕÁÁAYUYu¨YàÌj:<á3gâa:æ¦×¯_OsÖ8»qãFYUY´]ÎLÛ·oÇé'ÒaK¶Ëª¬Èê.]º[§.­é¥lUYýýý17=pàÀ¶mÛÒ!õã´»»;Nß|óÍSvìØ½«UVe@Vgø°Õë×¯¦¤»víZ,<y2çÏ¿hÑ"³UYÕ¤×SwîÜùÎ;ïÅ%KÄòÞ½Ó;mjÙ<88ØÜÜ×ÖÖ>¥µDzáVVeà)Ïj$-/_¾Ê7>>éiM3×X¹aÃêß¤½½½««+:;;W¯^]¾§ñâÅeUVò¬FD³ÉeÚ_)E.ÂÞºu+NwíÚUýû444¤©íÄÄDKKKÉ¥|ðAL³¬þô§?]ü°ã>óÌ3þ¨²ðÄÏV#«QÓ'NÄctkkë±cÇb!½u58Nûûû«ºººËatttÉ%é]°iÍ/~ñ>ì/þâ/êëëýQeàÏê½÷â4æ£QÖXØ»wo±XLmÚ´)¸W­Z5ã7É¾$D óÅ?>EÚF`YxÊ³PiÑ¢EéXiýñãÇóp^EccãÄÄDÚË%ÇÈÕ¹ÍjkÊ|+²¤ÏUM,Ð¦cDlÙ²%NzzªmGGÇÁc!NÛÛÛ§;ÙêÜúäa²ð­ÈêØØØ®]»æÏûöí8ìØ±=ö¤ã§c¹X,ÞºukºïÐÛÛÛÔÔ×innÎ^-é¨¬~WðÛÌêÄÄÄÙ³g,Yr÷îÝ´fdd$ÒÙêèè¨ÃAÈ*¬ú`8Y@VUYUYUYUYUd@Veõqduß×ðÙgC@Ve/­rÍlE?ùäcÈª¬2Z[[eUYEVdUVe@VeUVdUVUYEVdUVe@VeUVdUVUYUYUYUYUd@VUYUYUYUYUd@VeUVdUVe@VeYUd@VeUVdUVe@VeYUYUYUYUYEVdÇ¦P(¬X±bÍWV®íÚ5ÃÈª¬2üñ¾¨¬É+ «²ÊÜ°MUYEVdUVe@VeUVdUVUYEVdUVDñçØ·oßéø@VeÙX¾|ù/¾¸ü+ÞÆÈª¬2gldUVUYEVdUVe@VeYUd@VeõIT(~òä?9î³Ï>3,¬Ê*³ñÎ;ïüÙýYö!çÞÆÈª¬2gldUVUYEVdUVe@VeYdUVUYUYUYUYdUVUYEVdUVe@VeYdUVU§!«ÍÍÍuuummmçÎË_Ôßß¿xñâ¸hÑ¢Eq5YUYAWWW,tvv®^½:ÑøÃ¾¾¾Xèîî^°`¬Ê*¬Î ¡¡arr2&&&ZZZ¦»Ú¼yóRzßzØüÉ<óÌ3þ¨² «_¨«««¸700°~ýúXØ·o_ÇÃþôOÿTVe@V¿T,³åúúúò+···ß¹sÇF`YÕ466NLL¤À±é7Ö­[wóæMË*¬Î¬££ãàÁ±§1+Í_ÔÓÓóê«¯y¬ÈjMzÅbsssÿ7¥ðÅiii)äÈª¬ÈªÃA «¬Ê*A¡P?~ëWâì§~jXYUf#"úIN$ÖäUYenØ&Èª¬"«²¬Èª¬Ê*¬Ê*²Èª¬"«²¬Èª¬Êª¬²*«È*¬"«²*«² «²¬²*«È*¬"«²*«² «²Ê×T(V¬X±f+W®¼páQdUV©ÉÇ¼ozÝÓ§O%@Ve9°|ùrYdUVU@VeYUYUYUdUYEVdYUYýîjmm-­µköôô4@VeÊ®òIÍ^xáS[@Ve¹a1 «²¬²*«È*¬"«²*«È* «²¬²*«<^|ñÅûöUyNÜU «²J­³Õ¸'T9X¹, «²ÊEWVYUdUYEVYUd@VeYdUVU@VeYU¾»f<XÄ#ùì³Ï) «|§g«UñHBÚ²s`Í5²È*È* «²¬²*«È* «²²È*È* «²¬²*«|w­ZµjùòåûjöïÿþïdUV¡²¿ýÛ¿²®©cG¬Ê*Ì[AVedUU@VeYdUVAVYoÐ£¾ç©÷_üÂ½YU¥Gz7ÎSï^hmmu¯@Ve§O¹»q@VeUU@VAVeÖ¬Ö¯Ä®Ê"«sopp°¹¹¹®®®­­íÜ¹s5^$«ðÄÿÖOröíÛ÷â/duµ··wuuÅBggçêÕ««_tôèÑÿó°ýèGõõõþ¨ðN^óDimm½råï·:«±011ÑÒÒRý¢¿üË¿|ñaððÌ3Ïø£Âç×¿þõi4¿üå/oÝºåÞû­Îj]]]ÅåêÙ¬VP,³åúúú/UdµÆÆÆ´¥7k¼HVÕ:::<qÚÞÞ^ãE²¬VÐÛÛÛÔÔT,ûûû¿¼)ÂtÉ*²êpÈª¬¬¬Ê*²*«Èª¬¬Ê*²*«Èª¬¬¬Ê*²*«Èª¬¬æ²z~KþèþèéÉêo~ógy¦nNïß+uÔæw~çwBâ~÷.ãàßÐ¿áSöo³»_ýêWOIV¿ÿû¿öÙgC-þçþ§P(üÓ?ý¡¨Å+~ô£ZüÝßý]SSq¨Å7âßð_ÿõ_E-þðÿpË-ßÌÏUYUYUYUYUYUYUYUYUYEVeUVeYUYUYUYUYUYUYýæýÍßüq¨Å½÷þê¯þê¿þë¿E->üÿøÆ¡ÿöoÿöóÿÜ8ÔâÎ;ñoq5µøøol& « « «ßBÍÍÍuuummmçÎ3 õ÷÷/^¼8FiÑ¢E1bÆmFgÎ)îcÕÝ»woÝºuõõõÏ=÷±ª.háÂipzWEccc---Uá÷ Éêööö®®®Xèììzµ©è?üa___,tww/X°À¸UwÿþýxeÕXMgûöí~øáäädãùç7VÕ544ÆB¦r®ñl#ydÿzèq¬~qOÿêXÈ?Ça:óæÍ3nÕðÁ;wîÌþ·Õtbº022âÿ±FñÌ#í£§éYá*ñúë¯_¾|9Õò!zÜ&«êêê*.SÑÀÀÀúõë[1X²dIüßfÿÛÆªÊßîÝ»ãZDââÅÆªºÁÁÁ«¸_Åiü'®iÃËjù=îAÕÅb1[®¯¯7 U···ß¹sÇ¸U±jÕªóçÏçÿ·Uÿ¾ÄÂ¥Kâ¹±ªnéÒ¥ir]¶láª%«åCô¸MV466NLL¤±l@¦sãÆuëÖÝ¼yÓ¸Íø/g¬ªÿ÷ÌUõÉ½ázÔ¬Ñã4YÐÑÑqðàÁXÓzzz^õÕ±±1ã6ÿmc57>|8_zé%cU]ÌPc L¾&f®«¬Ñã4YýbÏ±¦¦¦b±ØÜÜÜßßo@*jii)·Úÿ·Õtnß¾½jÕªx-Y²äòåËÆªº+W®DMc¸â4W-Y-¢Ç=h²²²²È*È*È*È* « « «¬¬¬Sîß¿÷îÝ´ÜÝÝÿTêâÊñ%ùÙ·ù¿ºP¨øqW±~Û¶m÷¥PxåWN8±æ+±+×®]ëþ¬_ËÙ³g£(»wï~0õÉzaºk^¸paÕÔ¡b|á-[²2uuu¥b¥ëÏ7ïå_õGõ%nkvíÚUþS®]»ÅÏ:þüê)%ñ+	yW¯^­ø,!V._¾¼|åk¯½V¾2È*PëÔ-ú£ù¬ÆD6ÍÉ)iyll,¾pçÎÕ¿súÐï;wîÄò±cÇJ.ýè£²o]mxx8rëcâ¿4ûªê?4sòäÉ¸òúõëkÏêo¾Y²²¯¯/ÖGÚÝOU`fÑ­ÈFccã)7o®¯¯ßºuk65np¬_¸páÁÎg5fS²oÞÙÙ§ÍÍÍéúq6¦¶±.]ztJÜ¼×_½ú·Dooog?ûÙt7,o~÷ÝwË¯<22bû0²Ô$¶gÏìl/ÜÁÉ½1%_¦-[¶LÕägi¶ÿ¶ù¬æ7&Çò¢EjÏjú>o½õVÞWÜÞ[>[M*¾²TLäð²&¸o¿ýöýû÷óL/ nß¾½dRÓÍCÅÂØØXþåÕ³ºbÅì/¿üríYW»|ùrß:þÖÕti[[;²TkjÉë%YÎUÌGãÏ9­5;vì(ÿþùÙjt.íÕÛÛ[KVSS#;::jÏjm×³­­[·Ö¾4È*|G³z÷îÝlÿÞük«±BrøðáîîÙ³'Ö:u*[ÙÓÓ¶	gÓÙYMÓâlw¡Ç7[Ý¸qc~hõîÖøÚj©Y¾ß[#ðÉ'Sc"¥K§ÄlrûöíéM¨±æ²CCCÓeutt4ÕcÊj<HoÑ©ÒÈ>øàf«µì¢²<(NÕÈá²eË²ÆdÓ»PRJ+NÓË®ÃÃÃ%>¦ÀéÍ?1îiDÅw£VÉjz+mÉ­YfÕåÛV%«©Äi¶zöìÙGÊê²k¾÷ÞµgõÁÔp³oU"5þæÍ5f5exÉ%îÈ*ðu³z÷îÝòJvPªÕC¬_ºtiÅã%¥¬¼ÝãÇ9s&Makù]ÒkÀ%ÇAL»1W<8bÅ¬Æú¸o «@5.^¼8Ûs'bR8oÞ¼÷ß?¿#O(9ÜRÕÑÑÑnÚ´)½¦ö]q ÚÌç¶¶¶Öþ;ö÷÷üòý.^¼¸sçÎµk×V|+7oÞìÞ¬3«øþþ!;ækWeÚoÑãH¡°wïÞíß¿¿ÆI0È* « « « «¬¬¬²²²²È*È*È*þf]iðgÝYIEND®B`


Dëz3/Ä¬jÐ<½¬vttÈ4údÕ?rjÔÆUýúwoï(«÷ne:CÏl6«&¯¿þºÕöòUQTK'£çG_¾·ª¿éUµËÒõë×ÕVb5à.**«ÔHW½íÊódx$¨jÅ­#+è-3´]^åB5Jó©Ve¥/×Ê J½´ä,8«³Ö÷òI²ªwp7ÝP²*,-Ôn&­j_ëªê´¹ú¼Q­«ÞgÕ7J`)ø_ «À3³JÔG8Ô%²ÒzIMüÇãQi²ÖÖÖª7w	¾®_XV;Èl¿BÿÞªþú±¦~¿ßyeÕçfj/¤³ª6Ë~àÀýßJþ!fÛÿkhh(11Q-²v­ä|Î¥È*ð¬³êÓKÜ¦  @^%--->;ø¨½~¢¢¢ÔÝJn7«e^YÕ¿oªmè)÷£!õÒK/ÍùÇñÉªüY|>E³à¬*%%%Ú_	Uõ·U/ÔÛ±jk°¼RGwÌ «ÀÈjRRWÜÔö¾Q8PÜW;£JJ¿×ÖÖ¦~dÇÏ¹´£ûúWí¬¤íU«Èr<BoBBvv¶#Bè³*·QS8ÜWÏµ#N<,°«#:Éíå1dffêïGíz­mÁV<¥AVç¦ßá3Uoæåææ~Ò¹ý²ÛæIä3¨U=îììxck<R¦¡¡A~ÜL¬½DûüAê«vÈ* «UÈ*du<ËÏÞÝ «Ï<_xáËVTT"«K(«_ýêWÿËÖæÍÉ*Y%«@VAV¬¬Udd¬Y%«dÈ*È*ÕG3_¾ÔÑÑ!3çÎø¥yyyrÉÎÎ;þ|ãÙ¾dÕ9¿©wÇêåU_õÇ÷¾÷½S§NID7mÚTPPÐÝÝ­[_JJE$«°G«¿÷¾³³sïÞ½ú©Ï¦§§³²²e^®hµ¬ün]UX¡YµZ­###ú±é¹sç|n#YÕF«*«2ÀUSSS)"Y²ú¹ÂÂBµùWïøñãû÷ï×yÑ*Y%«ÈêYR6444>Î>£´´TÆ£CCCG«ccc «d¬ «_ðx<ê­S9MJJR×ÖÖJVý7ëG«ííí»víªÁF`²JVÕÇkÎ)«~%¤ê÷NKK+++;ú4£U²JVÕJJJêëëÕ|qqqCCþÚ©©)énQQÑÈÈúøMaa¡$¬U²¬ú°ÙlÚÙÌÌLÿ®=zTa³³³[ZZRSSÕ¦c²JVÉ*²úØæß¼¼<Ç#óMMMÌ7jÉTdðªmµè©S§×£¼ò#dUþùcbbü/w»Ý%<<<..NdYNF¥uuuÚÙÉÉÉ´´4-À²Ê|jg»ººde«-((Ðß_VåÉ;Û'ËËËÕòÌÞÞ^×ã.@VUÕ/ìØ±£§§'`V###Õ¯×«³éééþÁáìÍöß¯¿¸Søï¬Âï­ÌªôÒg~tttðq5Z%«È*UÉ¤ÍÍæ§ýÞ*Y@Vaä¬FEEy½^µXæÉ*²²ºð¬Úl¶²²2S«ÕJVUÕdUçYtt´Éd²X,N§¬ « «OY@VAVÉ*U²jô¬(¾*Óþ¦­ÖÑÃuddõ²*5éPþ%)+ÿÕUÍþýûå4??¿¿¿¿««KYzqqñèè(ù$«sl²ÊÿêNVËnîÏù_o¼â?ý»´üõO´ý=õÔ³w=àí;;;ÕÁØÕ£nÛ¶MKëÙ³gKJJÔ·ÈeffÒN²JV,¿¬JSËÿwG·Çú¨µÛ¾ºÙ>[ä¸ëo/5U_nÓÖÖ©.NIIQYõz½uuuÇ³»ví¢d¬X~YQ©4àU÷=qø£Ù2ä¸ëo¯ihh×ÎªiVVÊ*£U²:¬þÏæÛÉ¶mºvó3þÛX¦YÎÙ®zñÅåB»Ý®]¨­VWW;²JVCÍªÞ¡üKÎöOùo`ådUê«­VVV:uÀd¬XºY½zcHê8Ûl«-«ÿ$×UDWuØøø¸tÇj´ßÛÛËh¬UK7«ÿò/ÿOÆA&¹ÁlkÂñÿ»X£ÕW_µ³³SfNçÖ­[µ]JKKGFF¤¬ú!,È*Y°Ì6ÏomþYu»Ýqqq2NMNNÒv'®¨¨¤VWW½^i­ÖWU²À°Y]H½sç6ßÒÒ¢æ²³³i'YÕt×;2¥óÜoþ¬ « «÷Éï®µ¸dÊ¹qæèõdYY]ôÖKYÉ*²²JVÕ¹qè|²JV¬Ü¬þúaçÏ6]ñ¸µzýµÿhæØÚYOVÉ*ÕWÞHýä­túO~ý£õw>¶6÷;áôrè|²JV¬è¬îoW¬¯º÷OÃÝ Y=yòdAAÏ:¬UduÞYíïïOHHá©OV9t>Y>YÝua×_~(qiÏÿÿ,¯¬¦¦¦ª8­rè|²úì²zëö=­ª)ÙVÃÿO)«¿ý?¿ìý¯³Më/îÕ ï­úïÄÄ¡óÉêsÈª<G¥¬2e]Ë»ý W]NV<¯¬ÊôûüÑö~8øq÷Vý/äÐùdõfU:*eUôµuÀEV<ßÀó[ÏUOViVõÒ]ïUË+«Áqè|²JVUU²d¬U²¬¬Udd¬ «d¬®Ì¬(¾*Óþ¦­º±u²²ºð¬Ö5õÔ:>å_²².@VAVUUÆ¬¬UU²¬¬UddÕ¨YÝþFzU¦÷GX/ « «Ìêµ½æÚ+8ÙVÃ÷ « «ÏªÞ¡üKdYY%«È*È*Y@VAVÉ*U²JVÉ*²²JVUU²¬¬U « «dYY%«È*È*Y²JVÉ*Y@VAVÉ*²²JVUU²dd¬ « «Ï"«)WÞq§6UdduqUdd¬ « «dYY%«@VAVÕ=G'¯Ö:zdºx¥²²ºð¬Uý^¡SÊzøDK²­²²ºð¬jîöïÎºÀYY%«È*È*Y@VAVÉ*U²úT¸ÝnÅ×ÔÔ¤¿ª¹¹966V]5Ûs¬ « «bµZËËËe¦¸¸8--MUddd¿ÌÈiLLÌ=xÜ­[·È*²²ú§vNOOË×ëUíÔ¬]»vppPfäTæe&===ÌgÉ*²²ú9bÀyµ8""BÂ)§.K.¹ûöÇøáVUÕ/L&mÞl6ë¯JLLìêêRMJJâ½UdduQQQ^¯Wmù²dYYÀf³ÉZ­VýU2Bíìì¹Udduòd6LÅét~ñPÂ>0wîÜÊ8UNe~ég5ÙVãlÿTM=wXY «duY:Y½wäot(ÿLÒ×wÏ¬U²,«E·ÊÔÔÔßäµÅWYY «d¬ÎªøV¹jêöËû²®åUddu|Ð[sãY@VAVÉ*²²JVUU²d¬U²¬¬Udd¬ « «d¬ « «dYY]"Y­îmXqçöËûd÷½dYY] xxûA¯R®¼Ù:à"«È*Èê"Hw½CVUU²¬¬Udd¬Y%«d¬ « «dYY5LVm5RVÞ=s¥çî+d¬Õfµ¯ÿUMÒWgû§¬8U²JVU½CùÈ*²JVÉ*Y@VAVÉ*²²JVUU²JVUU²¬¬Udd¬Y%«d¬ « «dYY%«È*È*Y%«È*Èê3Èê#d[6±@VÉ*Y]xV5wûwg]`¬U²JVUU²¬¬Udd¬UddõYe5ÙVãlÿTMrµ	²JVÉê³zïþÈÞþèPþ%¤¯ï¹ÂÚY%«duYÕ«uô(¾ÊÚY%«d¬ « «dYY%«È*È*Y%«È*Y%«Ï*«Ûß¨S5Ý»?Â « «duY½vó³wÏ«LêÃ¬¬Y²²JVU=¾ «d¬U²¬¬Udd¬ « «d¬ «d¬UddõÙûnkÆú;·_Þ÷¯/¥ýYË²¬¬.ÜàÃ¡Ûze1«¬ « «@Ê*cV²¬¬Udd¬ «0XVÃÂÂ¶lÙ²ÇO]]úôi²JVU²:¬¦¦¦ªù¶¶69ÛÕÕ¥]URRBVÉ*²JVçUQ7ãÔ©S2®VëëëÉjp8m5ÚÄ* « «duáYÕÜíÞuU@V±Ò³ZPP 3ÓÓÓúÂ¢ªªj||¬Ud¬ÎÕn·=zÔëõJêôï°çv»-ül\þªÉÉÉ³Ù¼fÍææf²¬ÂøY­®®VÛÏ=+óùÜÜ(?nµZËËËe¦¸¸8--MU~~þ©S§ä~¤©k×®%«È*ÕÑÑÑ±±1Ç#]nllTøéÈÈH`æÆÄÄè¯ñ«Ï÷­·ÞúW[½zµtÉ*²dUí¦$dÐi·ÛeX)O¶ÊY¹Ðf³ÿqbÀyu¶°°0""Bîóúõëf>ÀSþ¸ÿüçf³¬ «0HVeZPP u:j&Ë%O°J+++ÿ¸ÉdÒæ%>WÊLwwwBBá7÷õ?H¶ÕHYeÿô¯±ºÈ*Vâ.K?üá¥¦2lÕ²:99-ó>!ùòz½j#°ÌûÛ@ÖYUe1«L%7ß+ºÊê «XYYvîÚµkdd¤¶¶Ví¾TWW§®=þ¼­¨¨r6­¬¬LfäÔjµê¯:xð úÙÎÎÎ7®¬jj='É*@V±²ÚÕÕ©¾øâúÛ¼ÿþûSSSAîDLÑÑÑ&Éb±¨ÍÈjÈ«öJMMqjBBBOOY@V±"6?GdYY%«dY%«LMMUUU%''U²¬báï­666ªÏ§ºÝn¹äÎ;«W¯NMMµÎP¹UG&«óÍj²­FÊ*Ó»g®ÜífÕU¬Ñêøø¸´SÄçC5r¶¨¨hduýÅôÖ«ÉïæÜ[_ÿ)«¤¯|!+@Vað¬NOOÇÅÅÉxTFÂårÉi[[>«v»%duðáÐÛíïåÜ8#Ó·>~-ëZÞbÝ3ßsU¬Ñª:CII´³¯¯ÏgäºÒ²ª'£U+Y@VÉêül:nY%«È*U§Ó¡¾¬FNÕ7­ÆÅÅéwYjii!«dY%«!U¥rÚÛÛ«ÞUÕ6k7p¹¬ «duf³Ùf³ÜðàÀÁÁÁ§ý É*²#dµ¿¿_OVåÉ!gT_322È*Y@VÉj¨TVUG¥µ2_UU¥¹&%%U²¬Õª÷V;::d¦»»[mgg§ú:²JVU²:·®®.	gOOÏlOÚÚZ²JVU²ºUdÊêÔÔÔÄÄ·ÛíOu/Y@Vað¬:Iiaa¡ÌÇÌ «dYÅÂ^¡æËËËõY,Y%«È*YÕôô´d5**ªnFvv¶ÙlÎÍÍÝ³gU&«dY%«!©¬¬ÔªÝn·X,l&«È*xL`U²¬âI³ª¾½OVÕ²U²¬Õ²:11ÑÞÞ:CÿÞªÌË©ö=¬d¬ «d5$SSSÓÓÓl&«È*!«A6U²¬U²JVU,É¬NLL<ÕÈ*²#duzz:66vÃuuufÈÌÑ£G#""rrr´KÔ!ÞáÈ*²VÁçgÈ*YÈ*VîF`²JVU,NV;::ÂÂÂìv»Ï«d¬ «dunÚ ÔÛ¨V«U²zìØ1íÕòòr¹$99¬Ud¬Î½×v Çã,C6Ud¬.èÃÂbccyo¬ «xÒ¬Ö××KVÛÚÚvÍÐºæØ±cd¬ «du%÷îÕøÑÌW±Êåùùùdõ	í9âH¶Õhë ¬ÂÈYñ¨ä³±±Q»¤³³S.9úôJÞråÍÖî/ÊÝÞíÞuu@VaØ¬Úíö¢¢"hCCvass³Ú&¬gWZV%¥Û/ïKw½#Óú;å,Y@VÉêÜo©FEE©ý$¥3ªªªòóóå«Õ*ój,ÛÑÑ±¢²ª'e%«È*YCRR¶°ËåÒe(ãÔ§R²¬ÂÈKDÏ=«%«dYÅeU¿Y%«È*!«ýýýåååK<Y%«È*Y]HVkkkµÎÉÙ¬¬¬gó É*²£eõ9ZiYM¶ÕÊ¿Ê´ç£¬êïYadd¬Îêßèw¶Êt¢øj^¡@V±²ÚÖÖö½ÖÇUUUeggÕÅÍjèj=RVVXYÅrÊª×ëõ?Òää¤:Daa!Y%«È*YUgg§OäÜn·µ©©IÎNMMU²¬ÕyìÖÖÖf6ù­52ZíèèuÌýââb²JVU²RVÏ;§f***ªªª6mÚÔÕÕ%g><66æs~²JVU²,«¥¥¥ÚYµ§ÒÖ­[Õ®ãããd¬ «du!YíîîÎÌÌKrrrÔ%d¬ «du~Y½~ýºÓéÃ!£ÕmÛ¶É+Y%«È*Y_V%*®j#ð+¯¼"3ª¬r!Y%«È*Y÷Fà®®®¢¢"»Ý®®2Z%«È*YÇãjZ,¤VVV¾þúër622RÎª-?^.Ü®ä¬þ«?è­¿ý W¦^ÏoÉ*²JVgåp8ôg¸¤F«n·%gõ-÷¿ÿæÇ¾ýò¾oüÚú;É*²JV®¥UV%®dY%«³R'lllQ©MÔUÎ<x¬Ud¬Îo¥Æ23::*§uuuê°À>ÛÉ*Y@VÉêº»»ÕaåÔf³µ··«ÖnÜ¸ÀdY%«óàt:elZZZ§©/§v»]NSRRêf>ZûT+Y%«È*Yú²Õ°êKâúúúd¾¾¾^æW¯^Ïh¬ «d5$êýÔýû÷L¦?wîú¤M([Ýn·Åb	SßÒêC½qKVÉ*@Vað¬JÒ$x===ª|cccHÊY5r8üN¬Vkyy¹Ì§¥¥ùïi¼aÃ²JV²gU"ª.ÕþJ*rÚvddDNÏ=ü~"##ÕÐÖëõÆÄÄø{òäIþjYýñ¼áq2Æ]µjÕrÉêú;Ó]ïÈråÍs¿ù;²¬Õ?¬JMëêê$¯¼òJuuµÌ¨®ªCË©Óé~'áááçEBBú¬º¤¦¦æÇ;räÙl^ÿsþáá'¿»Ö:à)çÆ£×OUd¬þÉää¤ÊxTÊ*3çÎ3LêªÃKSSSç¼íGRüxKK´6ëÐ[/e%«È*YJÆÇÇ»u,uymm­þÎòz½j#°Ì?öGVÉ*@Vað¬*êUÕ%´êÇÓæææà?k³ÙÊÊÊdFN­VkàGÆh¬d+!«çìÙ³«W¯³ÕÕÕEEEêøÀjç^7L###³Ý<¢££å6E#Ö§£duaY=|¢ånß°>ü+/¬béfÕëõ:	uIWW¤QB#£Õþþþ§÷ Éêl>nëM¶ÕìÎº Ì8Û?eåU,ÀÏYÅ¡üKd « «d¬dd¬ÕoüªiðáYÈ*È*Y] ÷½Y×òdÀ*Óú;[ « «du¤»Þ!«Y%Bd¬Udd¬ « «duÁvg]²TÜ©¢®µ@VAVÉêÂÕ5õ¨¦(¾l«a-UU²ºîöËÈµ@VAVÉ*YÈ*È*Y%«È*Y%«dYY%«d « «d¬dd¬.»¬n£Nûnó÷GX£dd¬.ÐÀ GÿÝæï¹Â « «duÔ:zN_eUU²JV²²JVÉ*²JVÉêSõý¶¿*ºU&eÉï&YÈ*È*Y]¸tJYeÌråÍõwU¬¬ÕEpûAïöËûÈ*@VAVÉ*Y@VAVÉ*²²JVÉ*@VAVÉ*Y@VÉ*Y%«È*È*Y%«YY]¹Y]qgÎ32½Ýþ^ÅÝÿAV²²JVèáV÷6|Ð[/Sºë)+YÈ*È*Y]RV³U¬¬U²¬¬Udd¬U¬¬U²¬U²JVUÕeÕoüú°LÈ*@VAVÉê¹ïÝÌº§±þâÎÖYÈ*È*Y]é®wÈ*@VAVÉ*Y@VÉ*Y]1YÝþFªéÞýÖnYY%«t½sàÝ3WdÀ*S²­ÆÙþ)k7¬¬ÕEp(ÿYÈ*È*Y%«YY%«dY%«d¬ « «d¬dd¬U¬¬U²¬¬Udd¬U¬¬U²¬U²JVUU²JV²²JVÉ*@VAVÉ*Y@VÉ*Y%«È*È*Y%«YY%«d «d¬U²¬¬ÕÞÊm®uôÜíöîÝaUU²:?GþÃåmûêvg]ÐOÉ¶wÏUU²ºdüz¢ø*«<¬b9eÕív[,ððð¸¸¸¦¦&ýUN§sÃrU||¼Ü¬U¬¬ÎÁjµËLqqqZZþªW_µ­­MfìvûºuëÈ*YÈ*Èê"##§§§eÆëõÆÄÄÌv³Þ×÷Ýï~wÕªUd¬ddõsáááçõWff¦Ìüò¿´=îµ×^#«d « «_0LÚ¼Ùlö¿ÁØØÕjg#0YÈ*Èê¢¢¢¼^¯Ú,ó>×fdd±'0YÈ*ÈêÜl6[YYÌÈ©JõW577oÝºÕãñùñÕí÷IYnÉô_nWU¬¬ÎJLÑÑÑ&Éb±8Î/JØç&&&&LgÅfµº·A55çÆõwU¬¬>-+!«Ûzeäú,³ºçÃÙþiénß0ëD¬¬Õ¹I5wg]8i¶£dd¬² « «d¬dd¬Ud¬U²JV²²jô¬®¿¸SØ&©©¿¬dd¬ö`ÔS|«<Ä¦Ê¸6ëZYÈ*È*Y]ôÖçÜ8CV²²JVÉ*@VAVÉ*Y@VAVÉ*YÈ*È*YþYM¶ÕøÑð­ÜæÃ'Zøûdd¬ÎÏÀ ÇÿàûÒÚmûêøûdd¬.»Ã»³.ð÷È*È*Y%«YY%«d « «d¬ «d¬U²UU²JV²²JVÉ*@VAVÉ*Y@VÉ*Y%«d « « «YY%«Ìj²­Æÿ ¡Lß?ädd¬Õ?ôüàí|¿Ê¤bÌ?È*È*Y%«@ÊJVAVAVÉêÒÍêú;Ó]ïÈ´ýò¾ÎûÝd « «duµ¸Ô´©ùûrJV²²JVXÉ*@VAVÉ*YÈ*È*Y%«d «d¬Õ÷ÛþªèVzÕï&YÈ*È*Y]¸tJYeÌråÍõwU¬¬ÕEpûAïöËûÈ*@VAVÉ*YÈ*È*Y%«d «d¬5«ë/îü ·^MW÷k²UU²º@÷Gßn/çÆ¾õñkY×òÎcÛu!ÙV#§³MrmÇ­ßñ²²JV¢¥v,þAÏÝ¾á Ó#³ « «d¬² «d¬U²UUU¬¬U²JVAVAVÉ*Y%«YYYÈ*È*Y%«YY%«d¬d¬U²JV²²²UU²úU÷6¬¿¸sûå2ÉÌõ¡Î%þwg]ØõJõ$Kqëö=~ «d¬ÍÃ?<¼ý WM)WÞlp-ñü¿¹'£Õå>É+ÆÜ «d¬ë¥Uc`S6È*Y%«ddddd¬¬¬U²JVAVAVÉ*YYYYÅÿ¸ýò¾+oæÜ8£ÞnïÞzþ8dddó#CU)¨Ï$CX)+²²²E°ìÃ´,¼Ûè¹Û7,Ó§=à²JVÉêÊêú;¶³ø	>Ñ²m_Ýî¬Ûß¨K¶ÕðY%«du¥èõüÖË0[V%®ü@VÉ*Y[É*È*È*[í?~´þâÎ²²²EpûAïöËûø;UUU,>OÿlCØ9§«¿ûõÊj²­ÆßÉó'ÏïUU_n·Ûb±ÇÅÅ555xY5ã¦»Þ)ºUdffy=?xû£åþõ±ÏqR/JÈ*Èª/«ÕZ^^.3ÅÅÅiiiÁ¯ªªªúëÇ:tÈl6Ó$¨ýÇ7U³¿/é]ÖÓ7?þóÿø%jWêÓ|_k¦ õo¯ý¬¬ú¯×üª÷ÞïûÆ7¾±jÕ*´tÜïjp-÷éèõ2åÜ8#§ßmÍò±`¦àÓÖ2®Þ¾EVAV~Y%«Éd2ióf³9Ä«È*²²@TT×ëU[ze>Ä«È*²²Íf+++9µZ­!^EVUÕäÉm2,Óéüâ¡ÍvY@VAV²¬¬U «d¬YY²²JVUU²d¬U « «dYY%«@VÉ*Y²²dd¬ « «dÈ*Y]YeëÛßþ6Y%«KÅV­Z¾P&é^7"Y.YºpúÊW¾bÔEã9¹26øäOY5¯ýëEEEÃoÞ¼Ù&¯ëÃÂÂ<!îk_ûÚ¯~õ+C.Zffæw¾óC.Ãáçäøø8!Y%«d¬U²²JVÉ*Y%«dd¬U²JVÉ*È*Y%«d¬U²²JVÉ*Y%«ddõùÅ/~qíÚ5C.ZSSÝn7ä¢öÙg?ýéO'&&¹t?ûÙÏnÞ¼iÈEkll¬¬¬4ä¢õõõÉsÒëõ¬ô¬@V «KÛí¶X,áááqqqMMMX"§Ó¹aÃY¢øøxY:ã-ccccXXñþù&''322Ìfó5k´t²8±±±jAÔÁù°h'&&&ÈÄxëÕX­Öòòr)..NKK3À½úê«mmm2c·Û×­[g°eZV´hùùù§N­]»ÖHKÙßß/3rªR´ÜM^Èíyp·nYõ?¼¬ÈdÆëõê_CDDÁñäÉÚêÌH&c®®.C>9åUÂàà ÌÈ©zÅ°ÜmÇ===ú¬ú/±×- «³8o.+33ÓHË(cYUi«3#ýóÉã/,,WBë×¯iéÜn·,ü«É©<-³hú¬ú/×- «ÁL&mÞl6f¹ÆÆÆ¬V«úüa155µ¥¥E¿:3Ò?,Kii©ÌtwwË«#-]bb¢K_³hú¬ú/Q×- «sR2S7ÆBfddlÃg°>ýãWÃ,ÿ¸Í¦ÏªÿrÝ²:7ÍVVV&3r*Ã;,QssóÖ­[õÇ2Þ2j«3#-ÚÁ+**d¦³³sãÆFZ:¡ÊBÉLGG³hú¬ú/ñþß¬¤µµ5::Úd2Y,§Ói%ñÒoµÕmtt455Us			===FZº;wîHMeÑäTæ³hú¬ú/ñþß¬@V «¬@V «U@V «U@V «UÈ*ðMMMMLL¨y»Ý®ÿà:::äÆò#úµ»ûIð«»äò¼¼¼§ûÿ-,,99¹®®nÏd^.LOOçùUà8)Jaaá£o¸³Ý²½½=uêDQ~ðØ±cZÊËËU±Ôí#""6mÚ$WVVÊå>_Ô%=Öÿ·ôõõÉUò»ZZZÒføÄÏ'äAôöö| nÞ¼ÙÿÂmÛ¶ù_¨þ2È*êÐMú§æ¥ú¬Ê@VËéjÞãñÈ¿gõÝàããã2_]]ísíûï¿¯Ý¡v³ÎÎNÉ­_ýµÚOÿ¥úúz¹qfffèYMIIñ¹°­­M.´ó<È*07éd#**ªnFvv¶ÙlÎÍÍÕ³mËcccç¶>«2Ê´ÍÐî¼¸¸XN-º½×ë³2´yÉX5CÞ;q´¶¶Ê-?>Ûþ³²²üoÜÕÕÅöa¬!ig¥|ZäæÊHn×;6[VKKKõ£O5ZÕß­>«úÉ2zVÕý¼þúëAzp¯ÿhU	ø.2²äp^YUÜ½÷NMMé3©Þ@ÍÏÏ÷Êpóüùó2ãñxôo¯ÎÕ-[¶h·Ü´iSèYß(7ëéé	²ÔÚð7¬ªkãââxÂdÖT÷)²*óÿ)Ê666jtvvÊ%§Oö¿ýhU:§vjmm%«*Éª6-ô¬Ùv½°ÑªÈÍÍid+4«Úþ½ú÷Ve^¤¢¢Â§»EEEryCCvass³Ú&¬gfUµÝÞhõàÁr3ù¥Á»â«!¦Y>§ß|#pTTj¤4q&óóóÕPe^e;::fËj¿ª§Uy) >¢¤'O×h5]´UàÏ`t¶¬J´Æ¸m±úJiÀA¡zÛµ³³ÓçÚ§´XøGÆ ³½øiÔ YU¥õy´È*°ð¬úHß¶ YU%V£UÃ1¯¬8p@»åÑ£GCÏê£àjwåC5~hh(Ä¬ª'$$ðôÈ*ð¤YðßqÉg¥ Y=þ¼Ïccc¤²êón®ö0jkkÕ6eQïûQíÆðà³êv»åòuëÖñÜÈ*&66vÃÚ;2#Âý<ÂçpKZVûûûeøðaõùÐuÀ=j5ÃÃÃrí+¯¼ú2:Nßâ¿_Òõë×ÒÓÓ¾³³³y¶d[ÀÏÏô|±£¾vAö¡]BÿÍÂÂÎ;ðªÁÈ*dUÈ*dUÈ*d²È*d²È*d²ÀJðÿ,S°ÕW IEND®B`
